# From Divergent Guidelines to Delphi-Ready Items: Tutorial for Large Language Model–Assisted Guideline Harmonization Using Penile Cancer as a Worked Example

**DOI:** 10.2196/96886

**Published:** 2026-10-20

**Authors:** Maximilian Haas, Emily Rinderknecht, Matthias May, Maximilian Burger, Christian Gilfrich, Anne Herrmann, Sabine Brookman-May, Christoph Alexander Eckl, Udo Kruschwitz, Samy Ateia, Gregor Donabauer

**Affiliations:** 1 Department for Urology, University Hospital Regensburg, Caritas Krankenhaus St. Josef, Landshuter Str. 65, Regensburg, Bavaria, 93053, Germany, 49 941782 ext 3506; 2 Department of Urology, University Hospital Augsburg, Augsburg, Bavaria, Germany; 3 Department of Urology, Barmherzige Brüder Klinikum St. Elisabeth in Straubing, Straubing, Bavaria, Germany; 4 Department of Epidemiology and Preventive Medicine, Medical Sociology, University of Regensburg, Regensburg, Bavaria, Germany; 5 Department of Urology, Ludwig-Maximilians-Universität München, Munich, Bavaria, Germany; 6 Faculty of Information Science, University of Regensburg, Regensburg, Germany

**Keywords:** clinical practice guidelines, Delphi method, artificial intelligence, large language models, evidence harmonization, reproducibility, consensus methodology, rare diseases, white area analysis

## Abstract

**Background:**

Clinical practice guidelines are central instruments of evidence-based care; yet, in many domains, particularly rare or complex diseases, multiple guidelines coexist and frequently offer conflicting advice. Existing strategies for resolving such divergences are often labor-intensive, opaque, and insufficiently reproducible.

**Objective:**

This tutorial aimed to develop and demonstrate a reproducible workflow for transforming divergent clinical guideline recommendations and literature-derived white areas into human-reviewed Delphi-ready candidate items.

**Methods:**

Following a step-by-step tutorial structure, we demonstrate the workflow using penile cancer as a worked example. The process comprises manual selection of clinical guidelines, a systematic PubMed search for literature-derived candidate issues potentially unaddressed by the selected guidelines, selection of 3 complementary large language models (LLMs), and execution of predefined prompts, multimodal recommendation extraction, semantic harmonization, and item-level guideline stance classification. The evaluative process combines a 3-LLM ensemble consistency check with an independent pre-Delphi appraisal by 4 penile cancer experts using a predefined 5-point suitability scale and acceptance threshold.

**Results:**

In the worked example in the field of penile cancer as a high-stakes use case, the workflow extracted 157 clinical recommendations from 5 guidelines and consolidated them into 68 harmonized guideline-derived items. Stance classification identified 15 uniformly supported items and 53 divergent, ambiguous, or incompletely addressed items. In parallel, literature processing generated 95 harmonized white-area items, of which 9 were classified as true white-area items not addressed by the included guidelines. The final methodological output was a set of 62 human-reviewed Delphi-ready candidate items for subsequent clinical consensus voting.

**Conclusions:**

This methodology provides a scalable, transparent, and transferable approach for identifying and structuring interguideline inconsistencies and evidence gaps. By combining LLM-assisted analysis with independent external expert appraisal, it supports the reproducible transformation of heterogeneous guideline content into Delphi-ready candidate items.

## Introduction

Clinical practice guidelines are central instruments in the promotion of evidence-based care. By synthesizing complex bodies of research into coherent clinical pathways, they serve not only to standardize treatment approaches but also to enhance quality, safety, and accountability in health care delivery [1]. Across a wide range of disciplines, guidelines function as reference points for therapeutic decisions, reimbursement policies, and medicolegal standards. Yet in many fields multiple guidelines coexist, often published by distinct professional societies, health authorities, or international consortia. Particularly in rare or complex diseases, these parallel recommendations frequently diverge in scope, terminology, and the strength or direction of their guidance, creating substantial ambiguity for clinicians at the point of care [2,3].

Such inconsistencies are not merely editorial or semantic in nature. They can reflect fundamental disagreements in the interpretation of evidence, differences in methodological rigor, or varying thresholds for consensus among expert panels. For the practicing clinician, reconciling these contradictions imposes a considerable burden, both intellectually and operationally. Where no structured process exists to address interguideline divergence, treatment decisions become susceptible to variability, and the standard of care risks fragmentation. Beyond editorial inconsistency, unresolved guideline divergence represents a structural failure of implementation, whereby uncertainty at the evidence and synthesis level is systematically propagated into clinical practice and patient care.

This issue is especially acute in rare oncologic entities such as penile cancer, where high-quality data remain limited, and clinical recommendations are often based on retrospective analyses, small patient cohorts, or expert opinion [4-10]. In these cases, discrepancies between national and international guidelines are not only common but also difficult to resolve through conventional means. Unstructured reconciliation efforts, including ad hoc side-by-side comparisons and narrative expert reviews, can be labor-intensive, difficult to scale, and variably documented [11]. Furthermore, they offer little methodological guidance for other groups facing similar challenges in different disease domains. As the volume and complexity of medical evidence continue to expand, traditional guideline development processes increasingly struggle to reconcile heterogeneity, temporal lag, and contextual interpretation. Without reproducible mechanisms to harmonize conflicting recommendations, guideline ecosystems risk becoming progressively fragmented, thereby undermining consistency, equity, and trust in evidence-based care.

In response to this gap, we developed a reproducible and transferable methodology that identifies and transforms heterogeneous guideline evidence into human-reviewed Delphi-ready candidate items.

Our approach enables the systematic extraction of clinical recommendations from multiple guidelines, the identification of areas of agreement and conflict, and the transformation of these findings into clearly defined, Delphi-ready candidate items. The workflow incorporates multimodal content analysis across textual, tabular, and visual elements, allowing for a comprehensive mapping of the guideline landscape. Divergent positions are semantically unified and transformed into Delphi-ready candidate items that will subsequently undergo expert evaluation in an iterative Delphi consensus process.

In addition to capturing areas of interguideline contradiction, a structured and up-to-date PubMed search was conducted to account for newly emerging evidence that is not yet incorporated into current guidelines.

To demonstrate the feasibility of this approach, we applied it to the field of penile cancer. While clinically specific, the methodological blueprint was designed to be domain-independent and adaptable across medical specialties.

This methodological framework was designed as a reproducible and adaptable workflow with structured human oversight. Its attention to context and human involvement reflects considerations also addressed in implementation science frameworks such as the Consolidated Framework for Implementation Research (CFIR) and the Exploration, Preparation, Implementation, and Sustainment (EPIS) framework [12,13]. We draw on this literature to situate the methodology conceptually, while this study focuses on methodological development and its application in a worked clinical example. The resulting approach offers a scalable tool for professional societies, academic networks, and policy developers seeking to resolve guideline inconsistencies and strengthen the coherence and credibility of clinical recommendations.

## Methods

### Tutorial Aim, Scope, and Intended Use

This tutorial provides a reproducible and transferable guide for transforming divergent or absent recommendations across multiple clinical practice guidelines into externally appraised Delphi-ready candidate items (Figure 1). Rather than testing a clinical intervention or establishing disease-specific consensus, it explains the methodological decisions, required inputs, human-oversight procedures, and documentation standards needed to implement the preparatory workflow. Penile cancer serves as a worked example because of the well-documented heterogeneity among available international guideline recommendations in this field [2,3]. However, the methodology was designed to be independent of any particular disease or specialty. The tutorial ends with the generation and independent external appraisal of candidate items suitable for subsequent formal consensus evaluation. Formal Delphi voting, disease-specific consensus formation, and the development of clinical recommendations are outside its scope. The intended output is a transparent and scalable methodological template that can be adapted by research groups across different medical specialties.

**Figure 1.**
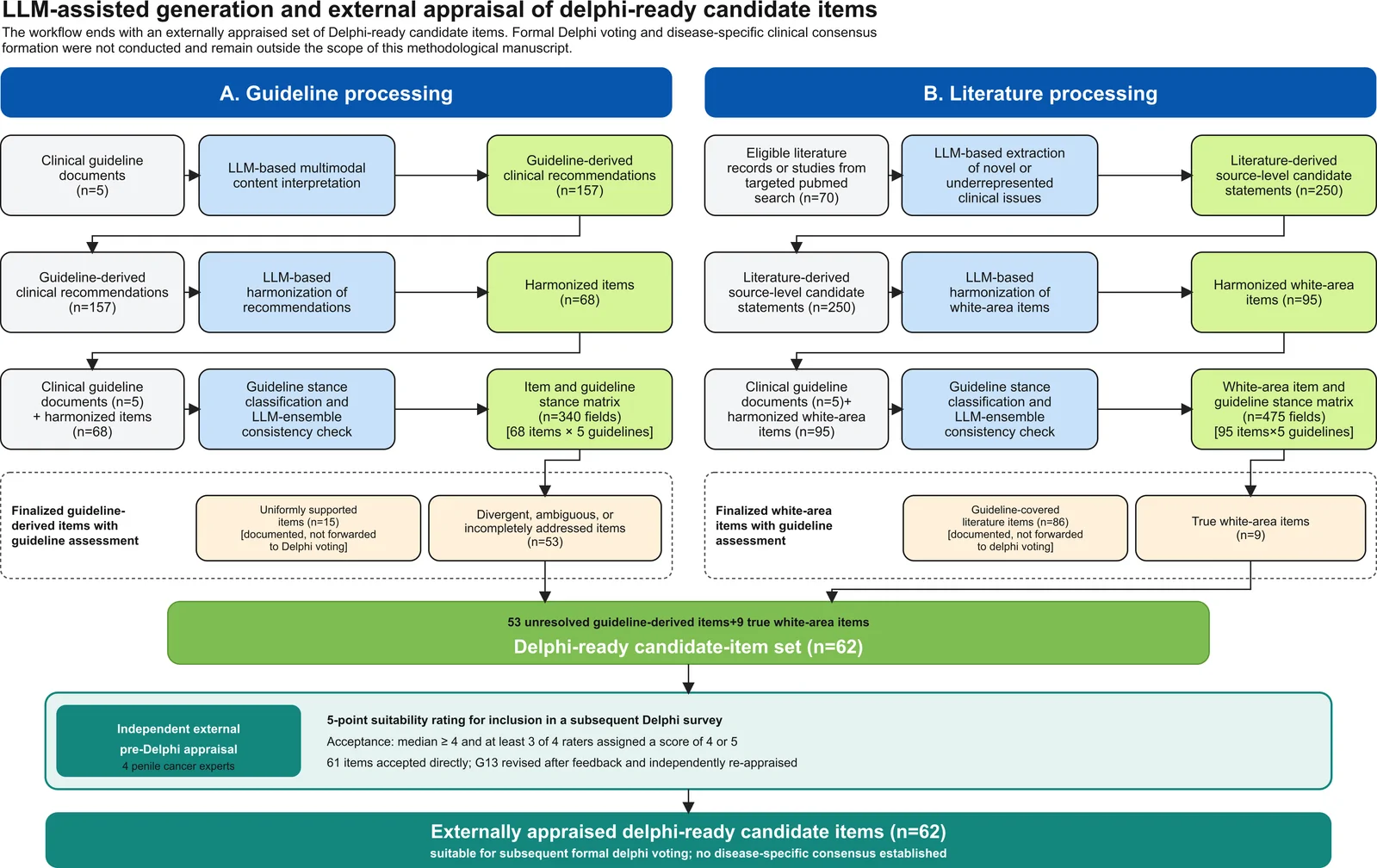
LLM-Assisted generation and external appraisal of Delphi-Ready candidate items. The workflow comprised two parallel processing arms. Guideline processing extracted 157 clinical recommendations from five guidelines and consolidated them into 68 harmonized items, of which 53 divergent, ambiguous, or incompletely addressed items were advanced. In parallel, 70 eligible literature records or studies yielded 250 source-level literature-derived candidate statements, which were consolidated into 95 harmonized white-area items. Of these, 9 were not addressed by any of the five included guidelines and were therefore classified as true white-area items. The resulting set of 62 candidate items underwent independent external pre-Delphi appraisal by four penile cancer experts. Sixty-one items met the predefined acceptability criteria directly; one item was revised following expert feedback and met the criteria upon reappraisal. The final output comprised 62 externally appraised Delphi-ready candidate items suitable for subsequent formal Delphi voting. The external appraisal assessed item suitability and did not constitute Delphi voting or disease-specific clinical consensus formation.

### Ethical Considerations

The study was approved by the Ethics Committee of the University of Regensburg (approval number: 25-4115-101).

### Overview of the Methodological Workflow

The methodology presented in this tutorial is organized into the following sequential stages, each of which is described in detail in the corresponding subsection below:

1. Selection and acquisition of guidelines: Relevant clinical guidelines were manually identified, assessed against predefined eligibility criteria, and obtained in their complete original format.
2. Identification and processing of white areas: A structured PubMed search was conducted to identify recent clinical evidence and potential issues not addressed by the selected guidelines.
3. Large language model (LLM) selection: Three complementary LLMs were selected for defined methodological roles, with their identifiers, configurations, prompts, and execution procedures documented for reproducibility.
4. Multimodal preparation and AI-based content interpretation: Complete guideline documents and eligible literature records were processed to extract source-level clinical recommendations and candidate statements.
5. Harmonization and semantic consolidation of recommendations: Semantically overlapping source statements were consolidated into nonredundant harmonized items while preserving their original clinical meaning and source attribution.
6. Matrix construction and guideline stance classification: Each harmonized item was compared across all included guidelines and classified as supported, differently addressed, or not addressed by each guideline.
7. LLM Ensemble consistency check: Three LLMs independently reviewed the item-level stance classifications to identify potentially unstable or incorrect classifications requiring reassessment.
8. Transformation of content into Delphi-compatible items: Unresolved guideline-derived items and literature-derived true white-area items were converted into a standardized format suitable for subsequent Delphi voting.
9. External expert appraisal of Delphi-ready candidate items: Four independent penile cancer experts assessed each candidate item for its suitability for inclusion in a subsequent Delphi survey using predefined criteria.
10. AI-Assisted revision of failed items: Items not meeting the predefined acceptance criteria were revised using the documented expert feedback and original source material and were subsequently reappraised.

Together, these stages describe the complete preparatory workflow from source selection to the generation and independent external appraisal of Delphi-ready candidate items. The subsequent sections provide a detailed description of the respective stages.

### Selection and Acquisition of Guidelines

Five comprehensive clinical guidelines were included in the analysis: the American Urological Association Update Series on Treatment of Early Stage Squamous Cell Carcinoma of the Penis [14], the German S3-Guideline on Diagnostics, Therapy, and Follow-up of Penile Cancer [15], the 2025 edition of the European Association of Urology–American Society of Clinical Oncology Collaborative Guideline on Penile Cancer, presented at the European Association of Urology Annual Congress Madrid 2025 [16], the European Society for Medical Oncology–European Reference Network for Rare Adult Solid Cancer Clinical Practice Guideline for Penile Cancer [7], and the National Comprehensive Cancer Network Clinical Practice Guidelines in Oncology: Penile Cancer, Version 2.2025 [17]. These were identified through targeted searches of professional society repositories, guideline databases, and international clearinghouses. Eligibility criteria required that the guideline be written in English or German, available in full-text format, and formally endorsed by a national or international professional organization. Each document was required to contain clinical recommendations relevant to the diagnosis, staging, treatment, or follow-up of the selected disease entity. All textual components, as well as nontextual elements such as flowcharts, tables, and visual contents, were included. No sections were excluded from the analytical process. For each issuing organization, the most recent publicly available guideline version at the predefined study cutoff date was included. Recommendations were not weighted according to publication year because the analysis was designed as a cross-sectional comparison of the guideline positions concurrently available to clinicians, rather than as an assessment of their relative validity or methodological authority. Publication years were retained in the guideline identifiers throughout the comparative matrices to preserve temporal transparency.

### Identification and Processing of White Areas

To complement the manually selected guidelines, a systematic PubMed search was conducted to identify recent publications containing clinical evidence that could reveal potentially relevant issues not addressed in the selected guidelines. Records retrieved through this search were treated as potential sources of white-area items and were not assumed to represent gaps in guideline coverage in themselves. A literature-derived item was designated a true white area only after its content had been extracted and harmonized and its final stance had been classified as “not addressed” across all 5 included guidelines.

The search was performed on November 7, 2025, using the following complete executable query: ((“penile cancer” OR “penile neoplasm” OR “cancer of the penis” OR “squamous cell carcinoma of the penis” OR “penile squamous cell carcinoma”) AND (“2020/11/07”[Date - Publication] : “2025/11/07”[Date - Publication]) AND (randomized controlled trial[Publication Type] OR review [Publication Type] OR meta-analysis[Publication Type]). The query incorporated a 5-year publication-date window (November 7, 2020, to November 7, 2025) and PubMed study-type filters for randomized controlled trial, review, and meta-analysis. The PubMed review category was used as defined by the database and was not restricted to systematic reviews. All records retrieved by the search were manually screened for eligibility by MH before any LLM-assisted processing. Titles and abstracts were assessed against the explicit eligibility and exclusion criteria, with full-text assessment when eligibility could not be determined conclusively from the bibliographic information. Records were eligible when they (1) focused primarily on penile cancer or penile squamous cell carcinoma or (2) reported separately identifiable penile cancer–specific evidence. Records were excluded when they (1) did not meet the previously defined publication-type criteria or (2) mentioned penile cancer only incidentally or did not provide separately interpretable penile cancer–specific evidence (Textbox 1).

Following LLM selection and prompt specification, each eligible publication was processed individually using the procedures described in “Multimodal Preparation and AI-Based Content Interpretation” section. The unit of extraction was an individual source-level clinical candidate statement rather than the publication itself. Accordingly, a single publication could contribute no candidate statement, one candidate statement, or multiple statements when it addressed distinct clinical decisions, patient populations, or interventions. The extracted statements were consolidated across publications according to the procedures described in “Harmonization and Semantic Consolidation of Recommendations” section, with semantically equivalent statements merged and clinically distinct statements retained separately. Each unique literature-derived item was subsequently compared with all 5 guidelines using the procedures described in “Matrix Construction and Guideline Stance Classification” and “LLM Ensemble Consistency Check” section. All contributing publications, harmonization outputs, and item-level stance classifications were retained in Sections 6-8 in Multimedia Appendix 1 to ensure complete source traceability.

Textbox 1. **Literature Search Strategy for Newly Emerging Evidence**

- Database and search date: PubMed (November 7, 2025).
- Complete executable search string: ((“penile cancer” OR “penile neoplasm” OR “cancer of the penis” OR “squamous cell carcinoma of the penis” OR “penile squamous cell carcinoma”) AND (“2020/11/07”[Date - Publication] : “2025/11/07”[Date - Publication]) AND (randomized controlled trial[Publication Type] OR review[Publication Type] OR meta-analysis[Publication Type])).
- Filters incorporated into the search string: Publication date from November 7, 2020, to November 7, 2025; article types randomized controlled trial, review, and meta-analysis.
·Screening procedure: All retrieved records were manually screened for eligibility by MH before any LLM-assisted processing. Titles and abstracts were assessed against the explicit criteria, with full-text assessment when eligibility could not be determined conclusively.
- Eligibility: Records were eligible when they focused primarily on penile cancer or penile squamous cell carcinoma, or contained separately identifiable penile cancer–specific evidence.
- Exclusion: Comments or responses; records mentioning penile cancer only incidentally or without separately interpretable penile cancer-specific evidence.

### LLM Selection

LLMs were used exclusively as methodological support tools to facilitate semantic structuring, extraction, and classification of guideline content. They were not used to generate clinical recommendations autonomously, nor to adjudicate clinical correctness. All model-generated outputs served as preparatory material. Following completion of the computational pipeline, the final candidate-item set underwent independent external expert appraisal for suitability for subsequent Delphi voting. Most computational steps were performed using a primary multimodal LLM; the use of 3 distinct LLMs was prespecified specifically for the ensemble consistency check. In the current implementation, three distinct LLMs were prespecified for the ensemble consistency check because 3 represents the smallest odd-numbered ensemble permitting a majority decision, without ties, on whether an existing stance classification should be retained or flagged for reassessment. Models from different providers were selected pragmatically to reduce dependence on any single model family and to increase diversity in model output behavior. Provider diversity was not assumed to guarantee independence of training data, architectures, or model-specific biases. Candidate models were evaluated based on their performance on the Humanity’s Last Exam (HLE) and Graduate-Level Problem Solving Assessment (GPQA) benchmarks, which assess expert-level knowledge of LLMs and therefore provide a reproducible foundation for comparing and selecting models for the framework [18,19].

The final model lineup consisted of Google’s Gemini 2.5 Pro (API model identifier: Gemini-2.5-Pro), OpenAI’s GPT-5.1 (API request identifier: GPT-5.1; corresponding dated snapshot: GPT-5.1-2025-11-13), and xAI’s Grok 4 (API request alias: Grok-4; canonical model identifier: Grok-4‐0709). Model selection and all API runs were performed on November 13, 2025. The exact request strings, model configurations, prompts, source code, timestamps, and archived outputs are publicly available in the version-controlled GitHub (GitHub, Inc) repository, providing model-level traceability and enabling independent auditing and methodological replication.

Gemini 2.5 Pro was used for most steps of the methodology, as we found it to be particularly effective at interpreting visual content, such as flowcharts, directly from guideline PDF files. GPT-5.1 and Grok 4 were used alongside Gemini 2.5 Pro in the ensemble consistency check described below.

### Multimodal Preparation and AI-Based Content Interpretation

Each guideline document was processed using a multimodal input framework to ensure that both textual and visual information were retained. The complete text of each document was extracted while maintaining its internal formatting, structural hierarchy, and contextual relationship. In parallel, the original, full-length guideline PDFs were provided as an auxiliary input to support the multimodal LLM’s interpretation of visual content.

Subsequently, the LLM was instructed to analyze the entire multimodal input and to extract every clinical recommendation, whether explicitly stated or implicitly embedded within figures or conditional pathways. The extraction prompt instructed Gemini 2.5 Pro to assign each recommendation to its principal aspect of care and provided representative clinical areas as nonexhaustive examples rather than as a fixed taxonomy. Because the resulting labels varied across the 5 guideline-specific outputs, MH, MM, and ER reviewed and standardized them by consensus into 7 overarching clinical domains for the guideline-derived recommendations: diagnosis and staging, primary treatment, lymph node management, recurrent disease, systemic therapy, palliative and supportive care, and follow-up and survivorship. Individual recommendations were assigned according to the principal clinical decision point they addressed, while more specific subcategory labels were retained where clinically informative. Each structured recommendation remained linked to its respective guideline source. The exact extraction prompt used with Gemini 2.5 Pro and the complete model-generated recommendation tables for each of the 5 included guidelines are provided in Section 1 in Multimedia Appendix 1. The corresponding API model identifier, execution parameters, timestamps, and archived outputs are available in the version-controlled public repository specified in the Data Availability section. The primary users of the AI-generated outputs were the research team responsible for guideline mapping and item preparation. Expert panel members did not directly interact with the language models; instead, they evaluated standardized candidate items derived from the preparatory AI-assisted workflow.

### Harmonization and Semantic Consolidation of Recommendations

After extraction, the clinical recommendations were grouped thematically and assessed for semantic equivalence by using an LLM. Statements with identical or near-identical clinical meaning but differing in formulation were consolidated into harmonized items. This process preserved the specific intent of each guideline while eliminating redundancy and terminological inconsistency. This process yielded a consolidated list of clinically relevant recommendations suitable for comparative evaluation and consensus rating (Section 2 in Multimedia Appendix 1).

### Matrix Construction and Guideline Stance Classification

A comparative matrix was constructed to evaluate how each individual guideline addressed each harmonized clinical item. The matrix was structured such that each row represented one unified recommendation, while each column corresponded to a single guideline. The aim was to determine, for every item, whether a given guideline (1) explicitly supported the recommendation, (2) provided a different or conflicting recommendation, or (3) did not address the item at all. This analysis was performed using a controlled prompting procedure in which the LLM was asked to review the full multimodal content of each guideline and to classify its stance with regard to each individual item. For each cell in the matrix, the model was required to provide a concise justification based on the original document, including direct textual references or summaries of relevant content (the detailed prompt and output classification are depicted in Section 3 in Multimedia Appendix 1).

### LLM Ensemble Consistency Check

To identify potentially unstable stance classifications, all model-generated classifications were subjected to an independent consistency check by an ensemble of three distinct LLMs, each developed by a different commercial model provider to reduce dependence on any single model family and increase diversity in model output behavior, as specified above. The use of an ensemble-based consistency framework was motivated by prior work demonstrating improved robustness and error detection in complex natural language processing (NLP) tasks [20]. The LLMs independently assessed the stance attribution and corresponding justifications. When at least two models assessed a classification as incorrect, the original classification was revised (Sections 4-5 in Multimedia Appendix 1). The 3-model ensemble served as a consistency check to flag potentially unstable stance classifications for reassessment. It was not treated as a validation reference standard. For reproducibility, all prompts, model versions, and run timestamps were archived in a version-controlled repository. Model outputs were logged with unique run identifiers, enabling full traceability of each classification and harmonization step. This allows independent teams to replicate the pipeline using either the same model versions or functionally equivalent successors.

### Transformation of Content Into Delphi-Compatible Items

The consolidated set of clinical items, which originated from both the harmonized recommendations extracted from existing guidelines and the evidence synthesized from white areas, was systematically converted into a format suitable for formal Delphi consensus procedures [21]. Each item was articulated in precise and declarative language, accompanied by its original source and a concise, evidence-based rationale. When relevant, the item also included a structured summary of observed discrepancies between the various guideline positions. When the guideline-derived and literature-derived items were combined, the 7 overarching guideline-derived domains were used as the initial organizational framework but did not constitute a closed taxonomy for the final candidate-item set. Literature-derived true white-area items were categorized according to the same principal-clinical-decision rule. Additional category labels were introduced when these items addressed topics not adequately represented by the guideline-derived domains, including general principles or management, cancer prevention, complication management, perioperative or postoperative care, and screening. MH, MM, and ER reviewed the resulting category framework and the final item-to-category assignments and finalized them by consensus. The complete item-level categorization is reported in Sections 9 and 11 in Multimedia Appendix 1.

### External Expert Appraisal of Delphi-Ready Candidate Items

After completion of the LLM-assisted extraction, harmonization, and stance classification workflow, the final candidate-item set underwent an independent external pre-Delphi appraisal. This appraisal was designed to assess whether the generated items were appropriate for inclusion in a subsequent Delphi consensus survey. It was not designed as Delphi voting, did not ask experts to agree or disagree with the clinical content of the items, and did not establish disease-specific clinical consensus.

External experts were selected according to predefined eligibility criteria to ensure domain expertise and independence from the item-generation workflow. Eligible experts had to be clinically or academically active in penile cancer and meet at least two of the following criteria: (1) at least 10 PubMed-indexed publications on penile cancer or penile squamous cell carcinoma; (2) participation in national or international guideline, consensus, or systematic review work related to penile cancer; (3) clinical activity at a recognized referral center or high-volume unit for penile cancer care; or (4) principal investigator, coprincipal investigator, or steering-group participation in penile cancer studies, registries, or multicenter research initiatives. Experts were not eligible if they were coauthors of this study, had participated in the LLM-assisted extraction, harmonization, stance classification, or candidate-item generation workflow, or had contributed to the drafting of the paper. Experts were instructed to complete the appraisal independently and were asked to disclose potential conflicts of interest before participation. A purposive recruitment approach was used to identify independent experts who met the predefined eligibility criteria. Seven penile cancer experts meeting the predefined domain-expertise criteria were approached individually by email through the authors’ professional networks: CG contacted one expert, MM contacted 5, and MB contacted one. One invited expert did not return an appraisal. Of the 6 returned forms, 5 were complete. The incomplete form was not evaluable because the planned item-level analysis required ratings for all 62 candidate items. One of the 5 complete appraisals was returned by an expert who, before initiation of the pre-Delphi appraisal, had already agreed to serve as a voting member in the subsequent disease-specific Delphi study. Owing to an administrative oversight, this expert had inadvertently also received the invitation to participate in the pre-Delphi appraisal and returned the completed form before the oversight was recognized. To preserve the methodological separation between pre-Delphi item appraisal and downstream clinical consensus voting, this appraisal was not included in the analytic pre-Delphi dataset. The remaining 4 complete appraisals constituted the final analytic panel.

Characteristics of the four included experts were summarized descriptively in Section 9 in Multimedia Appendix 1, including specialty, country or region, number of PubMed-indexed penile cancer publications, guideline or consensus involvement, referral-center activity, study or registry involvement, and declared conflicts of interest. External experts who agreed to disclosure were named in the Acknowledgments to allow transparent appraisal of domain expertise. Individual item-level ratings were reported only under pseudonymized labels (Experts 1 to 4) and were not linked to named individuals in the study or supplementary data. Expert identities were not used for scoring or weighting, and all ratings contributed equally to the analysis.

For each item, experts answered a single 5-point Likert-scale question: “Is this item appropriate for inclusion in a subsequent Delphi consensus survey?” Response options ranged from 1 (strongly disagree) to 5 (strongly agree). If experts selected 1, 2, or 3, they were asked to provide a brief reason using predefined categories and optional free text. The predefined categories were unclear wording, limited clinical relevance, redundancy with another item, insufficient derivation from guideline divergence or white-area evidence, need for merging with another item, and other (Section 9 in Multimedia Appendix 1).

Items were considered externally acceptable for subsequent Delphi inclusion when the median rating was ≥4 and at least 3 of 4 experts rated the item as 4 or 5. Items rated 4 or 5 by all 4 experts were classified as unanimously supported for Delphi inclusion. Items not meeting the predefined threshold, or items receiving two or more ratings of 1‐3, were flagged for wording revision, merging, reclassification, or internal adjudication before subsequent Delphi voting.

Interrater concordance was assessed across the four experts’ initial ratings of all 62 candidate items using Kendall’s coefficient of concordance, with correction for tied ranks. The repeat appraisal of the revised item G13 was not included in this analysis. Analyses were performed using IBM SPSS Statistics, version 31 (IBM Corp). Each individual rating of ≤3 underwent a substantive review addressing the underlying criticism. Based on this assessment, any potential operational consequences were identified, justified, and documented. If an item failed to meet the predefined acceptability criteria, it underwent structured reassessment and AI-assisted revision before being resubmitted to all 4 external experts.

### AI-Assisted Revision of Failed Items

Items that did not meet the predefined criteria for Delphi inclusion were revised through a structured LLM-assisted revision workflow that operationalized the experts’ qualitative feedback into a reproducible revision process.

For each item requiring revision, the LLM received four predefined inputs: (1) the original candidate item together with its assigned aspect of care; (2) the complete source document from which the recommendation had originally been extracted; (3) the complete cross-guideline stance matrix for that recommendation, including all supporting or conflicting positions and their justifications; and (4) the structured critique provided by the external expert reviewer explaining the reason for the low rating.

Importantly, the stance information from other guidelines was supplied solely to provide contextual information explaining why the item had been selected for review. The model was explicitly instructed not to revise the recommendation in order to maximize agreement across guidelines but instead to remain faithful to the original source document. Revision prompts emphasized preservation of the underlying clinical meaning unless the reviewer had identified a genuine inconsistency between the extracted item and its source document.

For every revised item, the model additionally generated a structured explanation documenting how the reviewer critique had been addressed, whether the clinical meaning had changed, and whether the requested revision was supported by the source document. This created a transparent audit trail for each revision before the item was resubmitted for independent expert reappraisal. The AI-generated revision was reviewed and approved by the author team before resubmission to the external experts. The complete prompts, model outputs, and revision logs are provided in Sections 10-11 in Multimedia Appendix 1 and the version-controlled public repository. Practical prerequisites for adapting this workflow to other clinical specialties are summarized in Textbox 2.

Textbox 2. **Transferability guidelines.**

For researchers aiming to replicate this methodology in other specialties, several prerequisites should be met:

- Availability of full-text clinical guidelines with structured recommendations.
- Language alignment, including translation workflows where necessary.
- Access to high-capacity LLMs capable of multimodal processing and prompt chaining.
- Technical infrastructure for iterative AI ensemble application, data security, and storage.

### Using Delphi-Ready Candidate Items in a Downstream Consensus Process

This tutorial ends with the generation and external appraisal of Delphi-ready candidate items. Formal Delphi voting, disease-specific consensus formation, and clinical interpretation of voting results were not conducted or analyzed as part of this tutorial. However, because the generated output is intended for subsequent consensus work, the candidate items can be transferred into a downstream Delphi process. In such a process, the panel composition, eligibility criteria, conflict-of-interest management, number of voting rounds, stopping rules, and consensus thresholds should be defined prospectively before the first voting round. The use of a 9-point scale aligns with established practice in consensus research and is well established in Delphi methodology [21,22]. It may be used to preserve granularity of expert agreement, with scores 1 to 3 indicating disagreement, 4 to 6 uncertainty or partial agreement, and 7 to 9 agreement. Items not reaching predefined consensus thresholds should be returned in a subsequent round with anonymized group-level feedback and, where necessary, wording clarification that preserves the original clinical meaning. The downstream Delphi study and its voting results should be reported separately according to established Delphi reporting standards. A concrete template for the downstream Delphi module, illustrated using the current penile cancer worked example and adaptable to other fields when following this tutorial, is provided in Section 12 and Table S1 in Multimedia Appendix 1.

### Transparency Reporting of AI-Assisted Components (Chatbot Assessment Reporting Tool)

Because the framework relies on generative AI systems for semantic extraction, classification, and preparatory synthesis of clinical guideline content, reporting of the AI-assisted components was guided by the Chatbot Assessment Reporting Tool (CHART), as proposed in *BMJ Medicine* [23]. CHART was applied to explicitly document model identifiers, intended use, prompt and query strategies, performance evaluation, human oversight, and AI-specific limitations. Items not applicable to a classical chatbot health advice study were transparently marked as partial or not applicable. The completed CHART checklist is provided in Textbox 3, Table S2 in Multimedia Appendix 1.

Textbox 3. **Optional downstream Delphi implementation module.**

For researchers proceeding from Delphi-ready candidate items to formal consensus formation, the following elements should be prospectively defined:

- Panel composition: Independent, multidisciplinary expert panel selected based on documented expertise, publication record, and prior consensus or guideline involvement; where relevant, complemented by a validation panel including patient advocates to ensure clarity and patient-centeredness.
- Conflict-of-interest management: Structured disclosure and management of relevant conflicts of interest prior to panel participation.
- Language alignment, including translation workflows where necessary.
- Voting procedure: Anonymous, digital, multi-round voting to minimize social desirability bias and dominance effects.
- Rating scale: 9-point Likert scale with predefined interpretive zones, with granular anchor definitions provided to panelists prior to the first round.
- Consensus definition: Prospectively defined consensus threshold (eg, ≥75% of ratings within the same 3-point zone), established before the first voting round.
- Inter-round feedback: Anonymized, group-level feedback (eg, response distribution, interquartile range, thematic comments) provided for items not reaching consensus, with wording clarification where necessary to preserve original clinical meaning.
- Stopping rules: Predefined criteria for response stability (eg, <5% change in the primary interpretive zone between rounds) and convergence (eg, IQR ≤2), alongside criteria for flagging polarized items for reformulation.
- Reporting: Separate publication of the Delphi study and its voting results, conducted in accordance with established Delphi reporting standards.

## Results

### Overview of Included Guidelines and Extracted Content

Five high-impact clinical guidelines on the management of penile cancer were included in the analysis. These documents were published between 2020 and 2025 and originated from leading international societies, including European, American, German, and multidisciplinary consortia. Collectively, the documents comprised 259 pages of content, which were fully processed using the multimodal extraction pipeline. From this material, a total of 157 distinct clinical recommendations were identified.

### Thematic Harmonization and Construction of the Recommendation Matrix

The 157 extracted recommendations were assigned to 7 overarching clinical domains of penile cancer management: (1) diagnosis and staging, (2) primary treatment, (3) lymph node management, (4) recurrent disease, (5) systemic therapy, (6) palliative and supportive care, and (7) follow-up and survivorship. Through deduplication and semantic consolidation, the corpus was reduced by 89 (56.7%) redundant or semantically overlapping source-level entries, yielding 68 (43.3%) unique harmonized guideline-derived items. These 68 items formed the rows of the comparative matrix, which captured how each guideline addressed each item (Sections 2 and 3 in Multimedia Appendix 1).

Of the 68 harmonized items, 15 (22%) were supported uniformly across all 5 guidelines and were therefore not advanced as unresolved questions for subsequent Delphi evaluation. The remaining 53 (78%) items showed divergence or incomplete coverage, meaning that at least one guideline provided a different recommendation or did not address the item. These 53 items were retained as guideline-derived candidate items. The matrix enabled explicit mapping of agreement and disagreement patterns and revealed recurring areas of divergence, particularly regarding indications for inguinal lymph node dissection, imaging protocols, and adjuvant treatment strategies. Complete source-level extraction tables, harmonization prompts and outputs, source attribution for the harmonized items, and item-level stance classifications are provided in Sections 1-3 in Multimedia Appendix 1 and the public version–controlled repository.

### Ensemble Consistency Check of Classification and Model Concordance

The initial guideline classification for each item was conducted using the primary LLM (Gemini-2.5-Pro). This classification was then reviewed by an ensemble of 3 LLMs to detect potential misinterpretations. In 91.5% (311 fields) of the matrix entries, all 3 models confirmed the previous classifications with consistent justifications. In the remaining 8.5% (29 fields), discrepancies were observed. For these entries, a corrective reassessment was performed by one of the models that identified a misinterpretation. The ensemble step was used to identify potential inconsistencies in stance attribution. It was not treated as a validation reference standard.

### Identification of White Areas

The literature search yielded 141 records. Two comments or responses were excluded based on publication type, and a further 69 (49%) publications did not meet the thematic eligibility criteria defined above, leaving 70 (50%) for analysis [24-93]. Because the unit of extraction was the individual clinical recommendation rather than the publication, a study could contribute multiple distinct recommendations. The 70 eligible studies [24-93] yielded 250 source-level candidate statements, which were consolidated into 95 unique literature-derived items, with the contributing source publications documented for each item (Section 6 in Multimedia Appendix 1).

Each of these 95 items was evaluated against all five included guidelines, resulting in 475 item–guideline classification cells. Ensemble review identified no need for correction in 440 cells (92.6%) and resulted in revision of 35 cells (7.4%; Sections 7-8 in Multimedia Appendix 1). Following this consistency check, 9 of 95 (9.5%) literature-derived items were classified as true white areas because they were not addressed by any of the five guidelines. The remaining 86 (90.5%) items were addressed by at least one guideline and were therefore not advanced as white-area candidate items. Each item was phrased in alignment with the existing matrix format and annotated with bibliographic references and concise explanatory notes. Importantly, the identification of guideline-independent white areas demonstrated that gaps in guidance were not random but systematically aligned with emerging evidence and clinically relevant decision points.

### Finalization of the Delphi Item Set

The harmonized guideline-derived items (n=53) and the white-area literature-derived items (n=9) were combined to form a unified set of 62 candidate items for expert consensus. Each item was accompanied by its origin, its current status across guidelines, and, where applicable, a summary of the evidence base extracted from the literature. The candidate items were organized into clinical decision-making categories according to the two-stage procedure described in the Methods section. The final categorization retained the seven clinical domains established for the guideline-derived recommendations and their clinically informative subdivisions and incorporated additional categories required for the literature-derived true white-area items. The category assigned to each item is reported in Sections 9 and 11 in Multimedia Appendix 1. This structure was intended to provide a coherent clinical sequence for subsequent expert assessment.

The analytical process yielded a comprehensive matrix of clinical recommendations, clarified the patterns of interguideline variability, and highlighted areas of missing guidance. The final product of this preparatory phase was a structured, evidence-informed, and thematically organized item set that is suitable for rigorous consensus assessment using a multiround Delphi approach. Application of the framework revealed that full concordance across major international guidelines was rare, with most clinical domains characterized by structured but persistent patterns of partial agreement. Most clinical domains exhibited partial agreement, with recurrent patterns of divergence clustering around nodal management, imaging strategies, and adjuvant treatment indications. All reproducibility materials, including full prompt sets, model parameters, and classification logs, are provided in Sections 1-8 in Multimedia Appendix 1.

### External Expert Appraisal of Candidate-Item Suitability

Of the 62 candidate items, 61 (98.4%) met the predefined external acceptability criterion at the initial appraisal. Expert 1 rated 59 items as 5 and 3 items as 4; Expert 2 rated 41 items as 5, 15 as 4, 5 as 3, and 1 as 2; Expert 3 rated 60 items as 4 and 2 items as 3; and Expert 4 rated 42 items as 5 and 20 items as 4. Kendall W indicated limited concordance in the relative differentiation of item suitability across the 4 raters (W=0.314, *χ*²_61_=76.57; *P*=.09). Nevertheless, 55 of 62 (88.7%) items were rated 4 or 5 by all four experts, indicating broad agreement at the prespecified suitability threshold. Six (9.7%) items received a rating of 3 or lower from a single rater but remained above the predefined threshold; 5 concerns related to wording clarity and one to insufficient derivation from white-area evidence. One item (G13) failed the initial threshold because 2 raters assigned a score of 3 for unclear wording. After structured revision and repeat appraisal, G13 met the acceptability criterion, resulting in a final set of 62 externally appraised Delphi-ready candidate items. A detailed description of the revision process, together with the final list of revised candidate items considered suitable for the Delphi process, is provided in Sections 9-11 in Multimedia Appendix 1.

These findings address different properties of the appraisal: threshold-based agreement reflects whether candidate items met the prespecified gatekeeping criterion for Delphi suitability, whereas Kendall W reflects consistency in the relative differentiation of items across the full 5-point scale. The appraisal assessed suitability for Delphi inclusion only and should not be interpreted as validation of the items’ clinical content or as disease-specific clinical consensus.

## Discussion

### Principal Findings

The divergence of international clinical guidelines has become an increasingly recognized challenge, particularly in diseases that lack robust, harmonized evidence or suffer from regionally fragmented care structures [2-5,8-10]. These discrepancies are not merely academic inconsistencies; they carry significant implications for patient safety, therapeutic equity, and the credibility of evidence-based medicine. In this context, the methodology presented in this paper offers a structured, transparent, and scalable approach for identifying, contextualizing, and structuring divergent recommendations across major guideline bodies. By uniting systematic literature screening, semantic decomposition, AI-supported item formulation, and structured pre-Delphi expert appraisal, we provide a methodological framework for generating Delphi-ready candidate items across a wide range of clinical domains that can subsequently undergo formal consensus evaluation.

Established structured approaches address complementary but distinct stages of guideline work. Appraisal of Guidelines for Research and Evaluation II supports the appraisal of methodological quality and reporting transparency [94], Grading of Recommendations Assessment, Development and Evaluation provides structured workflows for evidence assessment and recommendation development [95], and Guidelines International Network resources facilitate guideline development, adaptation, and collaboration [96]. These approaches strengthen the quality and governance of guideline development but are not primarily designed for the systematic item-level reconciliation of recommendations across multiple heterogeneous guideline documents. This framework addresses this specific preparatory task and is intended to complement, rather than replace, these established approaches. It does not assess overall guideline quality, grade the certainty of evidence, or substitute for systematic review and expert judgment. Instead, it produces a structured and traceable representation of interguideline agreement, disagreement, and noncoverage that can inform subsequent evidence appraisal, guideline adaptation, and formal consensus procedures.

Compared with conventional manual guideline comparison and narrative expert synthesis, the proposed framework primarily differs in how the preparatory workload is organized. Manual approaches remain indispensable for nuanced clinical interpretation but require repeated extraction, terminology normalization, cross-document mapping, and documentation of source provenance, particularly when multiple lengthy and structurally heterogeneous guidelines are assessed at item level. Rather than replacing expert judgment, the proposed framework redistributes this workload: LLMs support high-volume semantic processing, while researchers and independent clinical experts retain responsibility for source verification, correction of model outputs, item appraisal, and all clinical judgments. The distinctive methodological value of this architecture lies in its standardized representation of heterogeneous guideline content, multimodal processing capacity, semantic consolidation of differently worded recommendations, explicit mapping of agreement, disagreement, and noncoverage, item-level source traceability, and modular human-in-the-loop design. Applying the same structured comparison to recent literature additionally enables the identification of potential white areas not addressed by any included guideline. Together, these features provide a compelling foundation for the scalable, auditable, and transferable preparation of unresolved clinical questions at a time when the volume, velocity, and heterogeneity of clinical evidence increasingly challenge conventional guideline revision cycles. However, because this study did not include a parallel comparison with manual reconciliation or established guideline-development tools, these advantages should be interpreted as functional and architectural strengths supported by the demonstrated feasibility of the workflow, rather than as evidence of superiority in accuracy, processing time, personnel requirements, or cost.

Within the proposed framework, the Delphi process is intended to function not merely as a consensus-generating technique but as an epistemic adjudication layer that translates probabilistic evidence synthesis into clinically interpretable normative statements. Every preparatory step, from construction of the guideline matrix to the formulation and external appraisal of Delphi-ready candidate items, was designed to be methodologically auditable and suitable for interdisciplinary application. This role is expected to be particularly important in contexts where guideline divergence reflects interpretive uncertainty rather than evidentiary absence. In fragmented evidence ecosystems, the subsequent Delphi process provides a structured bridge between probabilistic AI-assisted evidence synthesis and independent human expert judgment, enabling consensus to be established on a transparent and reproducible foundation.

One of the defining innovations of this approach lies in the integration of LLMs during the preparatory phase. State-of-the-art models with transparent selection criteria were used to facilitate the semantic structuring of clinical statements based on curated thematic domains in clinical guidelines. Importantly, the LLMs were not tasked with adjudicating clinical truth but served as an instrument to accelerate and standardize the formulation of votable items. The applied methodology, however, extends well beyond this initial step. To account for relevant emerging studies and topics not yet incorporated into existing guidelines, a structured PubMed literature search constituted an additional core component of the approach. Recommendations derived from this process were first subjected to a structured, scalable LLM-based review.

To address the risk of relying solely on cross-model agreement, we added an external expert appraisal of the final candidate-item set. This step provided an independent human appraisal of the suitability of the final candidate-item set for subsequent Delphi voting. However, it should not be interpreted as clinical validation of the recommendations or as a substitute for formal Delphi consensus. The appraisal assessed whether items were sufficiently appropriate to be submitted to a Delphi panel; it did not determine the clinical answer to those items. The Delphi procedure itself will represent a distinct methodological component. Experts designated for participation in the planned downstream Delphi consensus process were not included in the analytic pre-Delphi appraisal panel, thereby preserving the methodological separation between candidate-item gatekeeping and subsequent clinical consensus voting.

Importantly, the relevance of the proposed workflow extends beyond penile cancer. Rather than representing a disease-specific solution, it provides a generalizable methodological framework for transforming dispersed narrative recommendations into structured candidate items that can subsequently be refined through independent expert consensus procedures. Such an approach may be applicable across clinical domains in which recommendations remain sparse, weakly graded, or structurally noncommittal because of limited or heterogeneous evidence. Typical examples include rare diseases, uncommon clinical scenarios, or highly specialized subspecialty questions in which expert interpretation necessarily complements the available evidence.

By systematically converting narrative expert judgment into transparent, reproducible, and votable candidate items, the workflow establishes a reproducible methodological pathway from heterogeneous guideline content and emerging evidence to structured candidate items for subsequent expert adjudication. Its principal contribution therefore lies not in harmonizing the recommendations themselves, but in providing a scalable framework that enables future expert panels to generate harmonized consensus statements in areas characterized by interpretive uncertainty, weak evidence, or parallel guideline ecosystems. In contrast, in clinical fields supported by large, consistently graded randomized evidence and broadly concordant international guidelines, the incremental value of such a Delphi-based harmonization framework is likely to be more limited.

This observation already highlights an inherent limitation of the approach; beyond this, several additional limitations warrant discussion. The use of AI models in a methodological pipeline of this kind is inherently shaped by the underlying architecture, training data, and versioning of the model.

Hence, the model selection strategy for this methodology followed a systematic and qualitatively grounded rationale. Models were selected from different commercial providers as a pragmatic diversification strategy. Because proprietary training data and architectural details are not fully disclosed, provider diversity cannot be assumed to ensure statistical independence or eliminate correlated model biases. Model selection was further based on performance on the HLE and GPQA benchmarks, which emphasize deep conceptual reasoning and complex problem-solving rather than surface-level linguistic competence [18,19].

By integrating provider diversity with benchmark-based selection criteria, the methodology aims to balance robustness, reasoning quality, and complementary model perspectives. Consequently, the model selection process is grounded in measurable expert-level performance and strengthens the conceptual rigor of the proposed framework across a wide range of application domains. The LLMs used in the current study, however, still reflect a specific point in time within a rapidly evolving technological landscape. For instance, we used Google’s Gemini-2.5-Pro, OpenAI’s GPT-5.1, and xAI’s Grok-4. Successor models may offer improved domain specificity, better contextual comprehension, or more advanced capacity for nuanced evidence synthesis. Consequently, while our findings are valid within the scope of the current framework, future applications may benefit from refined prompt engineering, enhanced fine-tuning procedures, or even domain-specific LLMs designed exclusively for medical consensus work.

In this context, the pipeline’s modular architecture constitutes a strategic advantage for future-proofing, as it allows for seamless substitution or upgrading of individual model components without altering the overall methodological structure.

### Limitations

Some observed divergences may reflect asynchronous guideline updating cycles or differences in evidence cutoff dates rather than persistent substantive disagreement between professional organizations. The divergence classification should therefore be interpreted descriptively as representing differences among the guideline versions available at the study cutoff date, without implying the superiority of either the older or the more recent recommendation.

Although the LLM outputs were treated as preanalytic material, the risk for automation bias remains an issue that requires attention [97,98]. We sought to minimize the likelihood of any practically relevant bias by deliberately separating LLM-based item generation from independent external expert appraisal, thereby ensuring that only human-reviewed candidate items are advanced to the planned Delphi consensus process.

While the final candidate-item set underwent independent external expert appraisal, the stance-classification matrices were not fully reannotated against an independent dual-expert reference standard. The LLM ensemble step should therefore be interpreted as a consistency check rather than as formal validation of classification accuracy. Future studies should include sampled or full reannotation of stance-classification cells by at least 2 independent human experts. Disagreements should be resolved through adjudication to establish a human reference standard, and agreement among the human annotators and between the human reference and the LLM classifications should be quantified using appropriate interrater reliability statistics.

The external pre-Delphi appraisal was characterized by a pronounced ceiling effect and restricted scale use by three of the four raters. The concentration of high ratings is compatible with the appraisal’s gatekeeping purpose but limits its discriminatory resolution across candidate items. Accordingly, the high overall acceptance rate should not be interpreted as evidence of item validity. The limited Kendall W similarly indicates restricted concordance in the relative differentiation of items across the rating scale. In addition, all four experts were urologists or uro-oncologists working in Germany, limiting both disciplinary and geographic diversity. Future applications should therefore consider broader multidisciplinary and international expert panels.

Furthermore, particular care was taken to ensure full transparency and traceability of the process. Every step of the workflow was disclosed, not only to document what was generated by the LLM, but also to make explicit how these outputs came into being. This approach aligns with established principles of responsible AI deployment in knowledge production [99,100].

In addition, certain model-specific limitations regarding input modality had to be accommodated. While Google’s Gemini-2.5-Pro model supports extended context windows of up to 1,000,000 tokens, thereby enabling full integration of raw PDF documents as byte streams, this was not feasible with GPT-5.1, which is currently limited to a context length of 400,000 tokens. In instances of very long guideline files, the raw PDF content was truncated, potentially reducing the comprehensiveness of guideline interpretation for this specific model. In our experiments, this occurred only for the guideline document from the German society; all other guidelines fell within the 400,000-token context window of GPT-5.1 and were therefore not affected. Moreover, the discarded content was limited to the document’s bibliography, meaning all substantive content was preserved.

While LLMs were used to enhance scalability and transparency, their outputs remain inherently dependent on model architecture, training data, and versioning. Consequently, the framework emphasizes modularity, allowing individual components to be updated without altering the underlying methodological logic.

Finally, although the LLM-based components of the framework are broadly applicable to guideline documents in other areas, the prompts used at each step rely on domain-specific formulations that require adaptation to the particular use case by qualified medical professionals.

Despite these constraints, the methodology offers several forward-facing opportunities for the evolution of guideline development. As generative AI models become increasingly capable of ingesting, comparing, and synthesizing vast bodies of heterogeneous clinical data, the vision of real-time, adaptive, and continuously updated guidelines becomes conceivable. In such a future, guideline development may no longer follow a static, multiyear revision cycle but instead operate as a dynamic, modular architecture that integrates expert consensus, patient preferences, and emerging evidence through AI-moderated pipelines.

In this prospective paradigm, Delphi-based consensus could serve as the adjudication core of an AI-augmented guideline engine. AI could continuously scan the literature, identify areas of ambiguity or divergence, and formulate candidate items. These items would then be subjected to real-time review by curated expert panels, whose feedback loops would be re-integrated into the system for iterative refinement.

At its core, the framework presented here does not claim to resolve the challenges of fragmented guideline ecosystems in a definitive manner. Rather, it offers a reproducible, auditable, and extensible approach that may serve as a methodological cornerstone for future consensus-building efforts. By openly describing our process and detailing each procedural element, we aim to enable other working groups across disciplines to replicate, adapt, and refine this model within their own clinical realities. As medicine continues to grapple with the dual pressures of evidence inflation and epistemic fragmentation, structured methodologies such as the one proposed here will be essential for maintaining coherence, transparency, and trust in medical knowledge. This methodology is directly applicable to guideline panels, professional societies, and health technology assessment bodies seeking to reconcile divergent recommendations systematically. It provides a reproducible, Delphi-ready matrix of candidate items as a starting point for expert adjudication. The approach is not intended to replace evidence grading systems or systematic reviews, but to complement them by offering a transparent mechanism for harmonization.

### Conclusion

The methodology presented in this study provides a structured and transferable approach for identifying, mapping, and organizing inconsistencies across clinical guidelines and areas of missing guidance. By combining systematic guideline mapping, white-area identification, LLM-assisted item generation, and independent external expert appraisal, the framework enables the reproducible transformation of heterogeneous guideline content into Delphi-ready candidate items.

While this framework was developed in the context of a rare urologic malignancy, its principles are broadly applicable to any field where fragmented recommendations impede the development of unified care strategies. The integration of LLMs not as clinical arbiters but as tools for semantic structuring and preparatory synthesis represents a pragmatic and forward-looking enhancement of classical methodology.

We invite research groups across specialties to adopt, refine, and expand upon this framework. Its modular architecture allows for tailored implementation, and its transparent design ensures methodological reproducibility. In an era of accelerating knowledge production and increasing complexity, structured consensus frameworks such as this will be essential to uphold clarity, comparability, and trust in evidence-based guidance.

## Supplementary material

10.2196/96886 Multimedia Appendix 1 Methods and outputs for the penile cancer worked example, including prompts, recommendation extraction and harmonization, guideline stance classification, external expert appraisal, item revisions, final Delphi-ready candidate items, planned Delphi voting procedures, and the reporting checklist.

## Abbreviations

CFIR: Consolidated Framework for Implementation Research
CHART: Chatbot Assessment Reporting Tool
EPIS: Exploration, Preparation, Implementation, Sustainment framework
GPQA: Graduate-Level Problem Solving Assessment
HLE: Humanity’s Last Exam
LLM: large language model
NLP: natural language processing
xAI: explainable AI
