# Supplementary material for: From Divergent Guidelines to Delphi-Ready Items: Tutorial for Large Language Model–Assisted Guideline Harmonization Using Penile Cancer as a Worked Example
Source: J Med Internet Res. 2026 Oct 20;28:e96886. doi: 10.2196/96886 (PMC13683995; doi:10.2196/96886)
Supplement: Supplementary file 1 [file jmir-v28-e96886-s001.docx]

From Divergent Guidelines to Delphi-Ready Items: A Tutorial for LLM-Assisted Guideline Harmonization Using Penile Cancer as a Worked Example

Table of Contents

[Section 1: Extraction of Recommendations From Each Guideline	3](#_Toc238648690)

[Prompt	3](#_Toc238648691)

[Output Tables with Extracted Recommendations From Each Guideline	4](#_Toc238648692)

[Section 2: Unique and Unified Treatment Recommendations	24](#_Toc238648693)

[Prompt	24](#_Toc238648694)

[Unique and Unified Treatment Recommendations	25](#_Toc238648695)

[Section 3: Stance classification	35](#_Toc238648696)

[Prompt	35](#_Toc238648697)

[Output Classification	36](#_Toc238648698)

[Section 4: Model Consistency Check	99](#_Toc238648699)

[Prompt	99](#_Toc238648700)

[Output Table:	99](#_Toc238648701)

[Section 5: Corrected Stance Classification	100](#_Toc238648702)

[Prompt	100](#_Toc238648703)

[Output Table:	100](#_Toc238648704)

[Section 6: Recommendation-Extraction From Current Literature	101](#_Toc238648705)

[Prompt: Single Paper Extraction	101](#_Toc238648706)

[Prompt: Unique Recommendation List	101](#_Toc238648707)

[Unique Recommendation List – Penile Cancer	102](#_Toc238648708)

[Section 7: Stance Analysis Of The Guidelines Toward Literature-Derived Recommendations	118](#_Toc238648709)

[Prompt	118](#_Toc238648710)

[Output Table on Stance Classification	119](#_Toc238648711)

[Section 8: Assessment of Stance Classification of Guidelines Toward Literature Recommendations	247](#_Toc238648712)

[Prompt	247](#_Toc238648713)

[Output Table	247](#_Toc238648714)

[Section 9: External Expert Appraisal of Delphi-Ready Candidate Items	248](#_Toc238648715)

[Table of Initial Candidate Items for the Delphi Process Prior to External Expert Appraisal, Derived From Divergent Guideline Recommendations (G01–G53) and the Supplementary Literature Review (W01–W09)	248](#_Toc238648716)

[Characteristics of the Four External Expert Raters	255](#_Toc238648717)

[Rating Framework and Assessment Criteria for Delphi-Ready Candidate Items	256](#_Toc238648718)

[External Assessment of the Delphi-Ready Candidate Items by Four Independent Expert Raters, Including the Median and Interquartile Range.	257](#_Toc238648719)

[Delphi-Ready Candidate Items Rated ≤3 By At Least One Expert Rater Regarding Their Suitability For Inclusion In The Delphi Process, Together With The Corresponding Reason For The Rating	260](#_Toc238648720)

[Substantive Appraisal and Resulting Actions for Candidate Items Receiving Ratings ≤3	261](#_Toc238648721)

[Section 10: AI-Assisted Revision of Failed Items	265](#_Toc238648722)

[Prompt	265](#_Toc238648723)

[Output Table Containing the Revised Failed Items	266](#_Toc238648724)

[Section 11: Repeat Expert Ratings of Failed Items and Final Delphi-Ready Candidate Item List	266](#_Toc238648725)

[Repeat Expert Ratings of Failed Items	266](#_Toc238648726)

[Final Delphi-Ready Candidate Item List After External Expert Appraisal and AI-Assisted Revision of Failed Items	267](#_Toc238648727)

[Section 12: Planned Downstream Delphi Voting Procedure	274](#_Toc238648728)

[Table S1	275](#_Toc238648729)

[Table S2	277](#_Toc238648730)

[References	281](#_Toc238648731)

# Section 1: Extraction of Recommendations From Each Guideline

## Prompt

The following prompt was used to instruct the large language model (LLM) Google’s Gemini-2.5-Pro to systematically extract recommendations from each clinical guideline:

From the provided guideline on penile cancer, identify and extract all explicit treatment recommendations. For each recommendation, ensure the following elements are included and clearly presented:

1. Aspect of Care: Categorize according to the relevant clinical area (e.g., diagnosis, primary treatment, lymph node management, systemic therapy, follow-up care).

2. Specific Clinical Indications:

- Stage-specific details: Clearly specify applicable T-stages (e.g., PeIN, Ta, Tis, T1, T2, etc.) or relevant classification (e.g., grade, invasive vs. non-invasive).
- Grading information: Include tumor grade where mentioned (e.g., G1/G2/G3).
- Anatomical specifics: Clarify the anatomical location (e.g., prepuce, glans, shaft).

3. Treatment Modality with Precise Conditions:

- Include precise treatment modality (e.g., laser therapy, wide local excision, glansectomy) along with qualification clauses (e.g., "if clinically feasible", "if facilities available", "not recommended after failed treatment").
- Indications of treatment limitations: Clearly state any limitations (e.g., "off-label use", "not to be repeated after failure").

4. Additional Clinical Clarifications:

- For recommendations involving lymph node management, explicitly clarify if they refer to palpable or non-palpable nodes.
- For topical therapies or surveillance, specify applicable stages (e.g., PeIN/Tis/Ta), and include any mandatory control assessments (e.g., biopsy after treatment).
- For follow-up, explicitly state if it applies after curative treatment and specify follow-up frequency and duration.

## Output Tables with Extracted Recommendations From Each Guideline

**AUA 2020: Treatment of Early Stage Squamous Cell Carcinoma of the Penis**

| Aspect of Care | Recommendation |
| --- | --- |
| Diagnosis and Staging | Definitive diagnosis must be achieved through tissue sampling/excision. A thorough physical examination is required to assess lesion size, location, and involvement of the corporal body or urethra. |
| Diagnosis and Staging | Inguinal lymph nodes must be palpated for lymphadenopathy. Imaging (e.g., CT, MRI, Doppler US) is often required to determine the extent of tumor invasion and assess lymph nodes, as physical examination alone can result in false-negative findings. |
| Diagnosis and Staging | Computerized tomography (CT) of the chest, abdomen, and pelvis is typically performed to evaluate for metastatic spread. The scan should extend through the mid-thigh to ensure the groin is included in the interpretation. |
| Primary Treatment (General Principles) | For early stage (Tis, Ta, T1), low-grade (G1, G2), low volume disease with favorable histopathology, organ-preserving techniques should be considered. |
| Primary Treatment (General Principles) | The goal of surgical treatment should be R0 surgical excision (negative margins). A 2 cm surgical margin is no longer considered applicable; a 5 mm margin for G1/G2 tumors and a 10 mm margin for G3 tumors is suggested based on histological spread. |
| Primary Treatment (Topical Therapy) | Topical treatments (e.g., 5-fluorouracil, imiquimod), cryotherapy, and photodynamic therapy can be considered as first-line options for non-invasive and early-stage penile malignancies (carcinoma in situ, Ta, T1). |
| Primary Treatment (Topical Therapy) | 5-fluorouracil (5-FU) is widely accepted for early-stage penile condylomas and carcinoma in situ (Tis). |
| Primary Treatment (Topical Therapy) | Topical therapy is ineffective for more advanced stages of penile cancer and should only be prescribed after an early and accurate diagnosis is established. |
| Primary Treatment (Laser Therapy) | Laser therapy (e.g., CO2, YAG) can be used as monotherapy or with local excision for early-stage (Tis, Ta, T1) non-bulky tumors. |
| Primary Treatment (Laser Therapy) | A surgical margin of 5 mm is considered oncologically safe for laser therapy. |
| Primary Treatment (Surgical - Organ Preserving) | Circumcision is indicated and can be curative for low-grade, low-stage (Tis, Ta) preputial disease in uncircumcised men. For tumors near the coronal sulcus, the margin must be extended. |
| Primary Treatment (Surgical - Organ Preserving) | Mohs micrographic surgery can be considered for disease with favorable histopathology when the lesion is located on the distal penis or glans. |
| Primary Treatment (Surgical - Organ Preserving) | Glansectomy (partial or total) and glans resurfacing are options for tumors located on the glans penis. |
| Primary Treatment (Surgical - Aggressive) | Partial or total penectomy are acceptable options for patients with higher stage tumors, unfavorable histopathology, or recurrent disease, or for patients who would not be suitable surgical candidates in the event of recurrence. |
| Treatment of Recurrent Disease | For a recurrent lesion at a new site, treatment options include topical therapy, laser therapy, surgical excision, Mohs surgery, and glans-specific procedures (glans resurfacing, glansectomy) if applicable. |
| Treatment of Recurrent Disease | For a recurrent lesion at the same site, options include laser therapy and surgical excision. For high-risk recurrent lesions or if follow-up is unreliable, partial or radical penectomy is reserved. |
| Radiation Therapy | Radiation therapy may be considered for patients with T1-T2 disease who are unfit for surgery and are willing to accept the associated late-onset side effects. Surgery remains the preferred approach due to higher success rates. |
| Lymph Node Management | Following circumcision, patients with moderately or poorly differentiated tumors (grades 2 and 3) will require inguinal node dissection. |
| Follow-up Care | Close postoperative follow-up is crucial after all organ-sparing treatments, particularly circumcision. |
| Follow-up Care | After laser therapy, close follow-up is recommended, especially during the first 6 months when the majority of local recurrences develop. |
| Follow-up Care | Following any penile-preserving technique, close follow-up for at least 5 years is required to detect and re-treat potential recurrences. |
| Follow-up Care | Post-laser therapy follow-up schedules vary by laser type: • CO2 Laser: After 2 months, then every 3 months for 3 years, then biannually. • Nd:YAG Laser: Every 3 months for 2 years, then biannually. |

**Extracted recommendations from DGU 2020: S3-Leitlinie Diagnostik, Therapie und Nachsorge des Peniskarzinoms**

| Aspect of Care | Recommendation |
| --- | --- |
| Diagnosis & Staging | For unclear penile lesions, early histological diagnosis should be obtained. |
|  | For primary staging, cross-sectional and hybrid imaging methods should only be used for T3/T4 tumors or in cases of positive lymph node status. |
|  | For superficial, non-invasive penile carcinomas (Carcinoma in situ), a fluorescence-diagnostic technique can be used to better determine the extent of the lesion. |
| Treatment of Primary Tumor (General) | Patients with penile cancer should receive organ-preserving therapy depending on the tumor stage and differentiation grade. |
|  | Treatment of superficial and non-invasive penile carcinomas should only be performed after a conclusive histological diagnosis has been established. |
| Treatment of Primary Tumor (Non-Invasive/Superficial: PeIN, Tis, Ta, Precancerous) | For superficial, non-invasive penile carcinomas, an organ- and function-preserving curative treatment should be pursued. |
|  | Topical Therapy: Therapy with 5-fluorouracil or imiquimod can be used. Patients must be informed about limited efficacy and off-label use. This treatment should not be repeated after failure. |
|  | Laser Therapy: CO2 laser can be used for penile intraepithelial neoplasia (PeIN) and precancerous lesions. Nd:YAG laser with deep coagulation can be used for Ta/T1 tumors. |
|  | Control Biopsy: After topical medical therapy or laser therapy, a control biopsy must be performed to verify local tumor control. |
|  | Glansectomy: Complete surgical de-epithelialization of the glans ("glans resurfacing") can be used for Carcinoma in situ (CIS). |
| Treatment of Primary Tumor (Invasive: T1-T4) | Tumors confined to prepuce: Should be treated by circumcision. |
|  | Small T1/T2 tumors of the glans: Local excision should be performed. The non-diseased prepuce can be preserved. |
|  | T1/T2 tumors: Glansectomy should be offered, depending on tumor location and extent. If glansectomy is not possible and surgery is desired, a partial penectomy should be performed. |
|  | T1/T2 tumors (Non-surgical): Local radiotherapy can be performed as an organ-sparing therapy. Patients must be informed that percutaneous radiotherapy has a lower local control rate than surgery. When using radiotherapy or topical therapy, a circumcision should be performed. |
|  | T3 tumors (invasion of corpora cavernosa): A partial penectomy should be performed. For extensive tumors, a radical penectomy may be necessary. Alternatively, local radiotherapy (preferably brachytherapy) can be performed, though local control is lower than with penectomy. |
|  | T4 tumors: An extensive surgical resection or local radiotherapy should be performed. Neoadjuvant chemotherapy or radio-chemotherapy should be considered. |
|  | Surgical Margins: For organ-sparing surgery, the margin should be at least 1-few millimeters. In case of a positive surgical margin, a re-excision should be performed. |
| Treatment of Local Recurrence | After organ-sparing surgery: Recurrence should be treated according to the same principles as primary tumors. |
|  | After radiotherapy: Recurrence should be treated with surgical salvage therapy. |
|  | After topical or laser therapy: Recurrence should be treated with a different treatment modality. |
| Lymph Node Management (Clinically Negative Nodes, cN0) | Low-risk (pTa and pT1aG1): Operative lymph node staging can be omitted in favor of clinical surveillance only. |
|  | Intermediate/High-risk (≥pT1b): Clinically non-palpable inguinal lymph nodes should be invasively staged via either dynamic sentinel lymph node biopsy (DSNB) or modified inguinal lymphadenectomy. |
|  | Intraoperative Management (for cN0): Frozen section analysis should not be performed on tissue from a modified inguinal lymphadenectomy or DSNB. |
| Lymph Node Management (Clinically Positive Nodes, cN1/cN2/cN3) | Palpable nodes (cN1/cN2): Palpably enlarged inguinal lymph nodes should be histologically confirmed by operative removal. If metastasis is proven, a radical inguinal lymphadenectomy should be performed. |
|  | Fixed nodes (cN3) with good performance status (ECOG < 2): Neoadjuvant chemotherapy should be administered. |
|  | Primarily resectable fixed nodes: Neoadjuvant chemotherapy should be offered to reduce tumor burden and postoperative morbidity. |
|  | Intraoperative Management (for cN+): If intraoperatively suspicious nodes are removed, frozen section analysis should be performed. If positive, an ipsilateral radical lymphadenectomy should be performed in the same session. |
|  | Pelvic Lymphadenectomy: Ipsilateral pelvic lymph node dissection should be performed in patients with ≥2 involved inguinal lymph nodes or with extracapsular extension in at least one lymph node. |
| Systemic Therapy (Adjuvant) | pN1: Adjuvant chemotherapy can be considered for patients with an aggressive tumor (≥ G2) and/or a large metastasis (≥ 3 cm). |
|  | pN2/pN3: Adjuvant chemotherapy can be administered. |
|  | Adjuvant Regimens: The recommended regimen is 4 cycles of Cisplatin/Paclitaxel/5-Fluorouracil. Alternatively, 4 cycles of Cisplatin/Ifosfamide/Paclitaxel can be used. |
| Systemic Therapy (Neoadjuvant) | Regimens: Recommended polychemotherapy regimens are Paclitaxel/Ifosfamide/Cisplatin (TIP) or Cisplatin/Paclitaxel/5-Fluorouracil (TPF). |
|  | Consolidation: If neoadjuvant chemotherapy leads to a response, surgery should be performed after a maximum of 4 cycles. |
| Management of Metastatic Disease | General Principle: Patients with metastatic disease or requiring multimodal therapy should be discussed in an interdisciplinary tumor board. |
|  | First-Line Chemotherapy: In patients with good performance status (ECOG < 2), combination chemotherapy should be administered. It should be cisplatin-based if comorbidities allow. The recommended regimen is Cisplatin/Ifosfamide/Paclitaxel. Alternatively, Cisplatin/5-Fluorouracil can be used. Bleomycin-containing regimens should not be used. |
|  | Second-Line Chemotherapy: Paclitaxel can be used as monotherapy. |
| Management of Recurrent/Progressive Disease | Regional lymph node recurrence after surveillance: Should be treated according to the same principles as primary lymph node metastases. |
|  | Regional lymph node recurrence after radical lymphadenectomy: Should be treated with neoadjuvant chemotherapy and surgery after histological confirmation. |
|  | Regional lymph node recurrence after false-negative staging: Should be histologically confirmed and treated with surgery, plus neoadjuvant or adjuvant therapy. |
| Adjuvant & Palliative Radiotherapy | Adjuvant radiotherapy after lymph node dissection cannot be generally recommended but can be considered for patients at high risk of recurrence (e.g., high number of metastases, extracapsular extension). |
| Supportive & Palliative Care | For chronic lymphedema, a complex decongestive therapy (lymphatic drainage, compression therapy, and skin care) should be performed. |
| Follow-up Care | General: Regular, risk-stratified follow-up should be performed to detect recurrences and functional disorders. Patients should be taught self-examination for early detection. |
|  | Follow-up for Primary Tumor (Clinical Exam): For organ preservation, every 3 months for years 1-2, then every 6 months for years 3-5. For total penectomy, every 3 months for years 1-2, then annually for years 3-5. |
|  | Follow-up for Inguinal Lymph Nodes (Clinical Exam +/- Imaging): For pNx or pN1, every 3 months for years 1-2, then every 6 months for years 3-5 (optional imaging for pN1). For pN0, every 3 months for years 1-2, then annually for years 3-5. |

**Extracted recommendations from EAU-ASCO Collaborative Guidelines on Penile Cancer**

| Aspect of Care | Recommendation |
| --- | --- |
| Diagnosis & Staging | Primary Tumour Biopsy: Obtain a pre-treatment biopsy of the primary lesion when malignancy is not clinically obvious, or when non-surgical treatment (e.g., topical agents, laser, radiotherapy) is planned. |
| Diagnosis & Staging | Primary Tumour Imaging: Perform magnetic resonance imaging (MRI) of the penis/primary tumour (artificial erection not mandatory) when there is uncertainty regarding corporal invasion (T3) and/or the feasibility of organ-sparing surgery. If MRI is not available, offer ultrasound (US) as an alternative. |
| Diagnosis & Staging | Lymph Node Physical Exam: Perform a physical examination of both groins. Record the number, laterality, and characteristics of any palpable/suspicious inguinal nodes. |
| Diagnosis & Staging | Lymph Node Staging (Clinically Node-Negative, cN0): • High-Risk (T1b or higher): Offer surgical lymph node (LN) staging. • Intermediate-Risk (T1a G2): Discuss surveillance as an alternative to surgical staging with patients who are willing to comply with strict follow-up. |
| Diagnosis & Staging | Surgical Staging Method (cN0): • Primary Option: Offer dynamic sentinel node biopsy (DSNB). • Alternative: If DSNB is not available, referral is not feasible, or if preferred by the patient after being well informed, offer inguinal lymph node dissection (ILND) (open or video-endoscopic). • Pre-DSNB: If DSNB is planned, perform inguinal US first, with fine needle aspiration cytology (FNAC) of sonographically abnormal LNs. |
| Diagnosis & Staging | Lymph Node Staging (Clinically Node-Positive, cN+): • Biopsy: If there is a palpable/suspicious node at physical examination (cN+), obtain an (image-guided) biopsy to confirm nodal metastasis before initiating treatment. • Systemic Staging: In cN+ patients, stage the pelvis and exclude distant metastases with ¹⁸F-fluoro-2-deoxy-D-glucose positron emission tomography (¹⁸FDG-PET) computed tomography (CT) or CT of the chest and abdomen before initiating treatment. |
| Primary Tumour Treatment | Shared Decision Making: Offer a balanced and individualised discussion on the benefits and harms of possible treatment options with the goal of shared decision making. Inform patients of the higher risk of local recurrence when using organ-sparing treatments compared to amputative surgery. |
| Primary Tumour Treatment | Topical Therapy (Superficial Disease): • Treatment: Offer topical therapy with 5-fluorouracil or imiquimod to patients with biopsy-confirmed penile intra-epithelial neoplasia (PeIN). • Follow-up: Clinically assess treatment effects after a treatment-free interval and, in cases of doubt, perform a biopsy. If topical treatment fails, it should not be repeated. |
| Primary Tumour Treatment | Laser Ablation (Superficial/Early Invasive): Offer laser ablation using CO₂ or Nd:YAG laser to patients with biopsy-confirmed PeIN, Ta, or T1 lesions. |
| Primary Tumour Treatment | Organ-Sparing Surgery (Superficial/Early Invasive): • Indication: Offer organ-sparing surgery (circumcision, wide local excision, glansectomy, glans resurfacing) and reconstructive techniques to patients with lesions confined to the glans and prepuce (PeIN, Ta, T1–T2) who are willing to comply with strict follow-up. • Intra-operative Assessment: Perform intra-operative frozen section analysis of resection margins in cases of doubt on the completeness of resection. |
| Primary Tumour Treatment | Organ-Sparing Salvage Surgery: Offer salvage organ-sparing surgery to patients with small local recurrences that do not involve the corpora cavernosa. |
| Primary Tumour Treatment | Radiotherapy (Early Invasive): Offer radiotherapy to selected patients with biopsy-confirmed T1 or T2 lesions. |
| Primary Tumour Treatment | Amputative Surgery (Locally Advanced): • Partial Penectomy: Offer partial penectomy (with or without reconstruction) to patients with invasion of the corpora cavernosa (T3) and those not willing to undergo or comply with follow-up for organ-sparing surgery. • Total Penectomy: Offer total penectomy with perineal urethrostomy to patients with large invasive tumours not amenable to partial amputation. |
| Primary Tumour Treatment | Amputative Surgery for Recurrence: Offer amputative surgery to patients with large local recurrences or involvement of the corpora cavernosa. |
| Primary Tumour Treatment | Multimodal Therapy (Advanced/Unresectable): Offer induction chemotherapy followed by surgery to responders, or chemo-radiotherapy, to patients with non-resectable advanced primary lesions, or to patients with locally advanced disease who refuse surgical management. |
| Lymph Node Management | Inguinal Dissection (cN1 Disease): For patients with cN1 disease (palpable mobile unilateral inguinal node), offer either ipsilateral fascial-sparing inguinal lymph node dissection (ILND) OR open radical ILND, sparing the saphenous vein if possible. |
| Lymph Node Management | Inguinal Dissection (cN2 Disease): For patients with cN2 disease (palpable mobile multiple or bilateral inguinal nodes), offer ipsilateral open radical ILND, sparing the saphenous vein if possible. |
| Lymph Node Management | Minimally-Invasive ILND (cN1-cN2): Offer minimally-invasive ILND to patients with cN1–2 disease only as part of a clinical trial. |
| Lymph Node Management | Surgical Timing (cN1-cN2): Complete surgical inguinal and pelvic nodal management within three months of diagnosis (unless the patient has undergone prior neoadjuvant chemotherapy). |
| Lymph Node Management | Prophylactic Pelvic Lymphadenectomy (pPLND): Offer open or minimally-invasive prophylactic ipsilateral pelvic lymphadenectomy if: • Three or more inguinal nodes are involved on one side on pathological examination, OR • Extranodal extension is reported on pathological examination. |
| Lymph Node Management | Surgery after Chemotherapy (cN3 Disease): • For Responders: Offer surgery to patients responding to neoadjuvant chemotherapy (NAC) in whom resection is feasible. • For Non-Progressed: Offer surgery to patients who have not progressed during NAC, but resection is feasible. |
| Lymph Node Management | Contraindication (cN3 Disease): Do not offer video endoscopic inguinal lymphadenectomy for cN3 disease (fixed inguinal mass or pelvic lymphadenopathy). |
| Systemic Therapy | Neoadjuvant Chemotherapy (N-staged): • cN3 Disease: Offer neoadjuvant chemotherapy (NAC) using a cisplatin- and taxane-based combination to chemotherapy-fit patients with pelvic LN involvement or extensive inguinal involvement (cN3), in preference to up-front surgery. • cN2 Disease: Offer chemotherapy as an alternative to upfront surgery for selected patients with bulky mobile inguinal nodes or bilateral disease (cN2) who are candidates for cisplatin and taxane-based chemotherapy. |
| Systemic Therapy | Adjuvant Chemotherapy (pN3 Disease): Have a balanced discussion of risks and benefits of adjuvant chemotherapy with high-risk patients with surgically resected disease, in particular, with those with pathological pelvic LN involvement (pN3). |
| Systemic Therapy | Adjuvant Radiotherapy (pN2/N3 Disease): Offer adjuvant radiotherapy (with or without chemo sensitisation) to patients with pN2/N3 disease, including those who received prior neoadjuvant chemotherapy. |
| Systemic Therapy | Definitive Radiotherapy (Surgery Refused/Contraindicated): Offer definitive radiotherapy (with or without chemo sensitisation) to patients who are unwilling or unable to undergo surgery. |
| Systemic Therapy | Radiotherapy (cN3, Chemo Ineligible): Offer radiotherapy (with or without chemo sensitisation) to cN3 patients who are not candidates for multi-agent chemotherapy. |
| Advanced & Palliative Care | First-Line Systemic Therapy: Offer patients with distant metastatic disease platinum-based chemotherapy as the preferred approach to first-line palliative systemic therapy. |
| Advanced & Palliative Care | Contraindicated Chemotherapy Agent: Do not offer bleomycin because of the pulmonary toxicity risk. |
| Advanced & Palliative Care | Progressive Disease: Offer patients with progressive disease under platinum chemotherapy the opportunity to enroll in clinical trials, including experimental therapies within phase I or basket trials. |
| Advanced & Palliative Care | Palliative Radiotherapy: Offer radiotherapy for symptom control (palliation) in advanced disease. |
| Follow-up & Survivorship | Multidisciplinary Care: Deliver penile cancer care as part of an extended multi-disciplinary team comprising specialists in urology, nursing, pathology, radiology, oncology, lymphoedema therapy, psychology, and more. |
| Follow-up & Survivorship | Follow-up Schedule: Follow-up men after penile cancer treatment, initially three-monthly for two years, then less frequently, to assess for recurrent disease and offer patient support. At discharge, recommend self-examination with easy access back to the clinic as local recurrence can occur late. |
| Follow-up & Survivorship | Psychosocial Support: Discuss the psychological impact of penile cancer and its treatments with the patient and offer psychological support and counselling services. |
| Follow-up & Survivorship | Functional Impact Discussion: Discuss the negative impact of treatments for the primary tumour on penile appearance, sensation, urinary, and sexual function so that the patient is better prepared for the challenges they may face. |
| Follow-up & Survivorship | Lymphoedema Management: Discuss the potential impact of lymphoedema as a consequence of inguinal and pelvic lymph node treatment. Assess patients for it at follow-up and refer to lymphoedema therapists early. |

**Extracted recommendations from ESMO EURACAN Clinical Practice Guideline for diagnosis, treatment and follow-up**

| Aspect of Care | Recommendation |
| --- | --- |
| Diagnosis & Staging | Primary Tumour Assessment: Clinical assessment should record size, morphology, and relationship to adjacent structures. In cases of uncertainty, MRI or penile ultrasound (US) combined with an intracavernosal injection of prostaglandin E1 can be useful. |
| Diagnosis & Staging | Distant Metastasis Assessment: CT of the chest, abdomen, and pelvis is advised in all cases. For patients with high-risk disease, MRI or CT fusion PET can be used. |
| Primary Tumour Management | Penile Intraepithelial Neoplasia (PeIN):• Initial Treatment: Circumcision is recommended for any biopsy-proven PeIN located on the glans or prepuce.• Residual Disease: Following circumcision, treat residual PeIN with topical agents (e.g., 5-FU, imiquimod) or CO2 laser ablation. • Treatment Failure: If topical treatment fails, wide local excision or glans resurfacing should be considered. Response to topical therapy should be assessed clinically or with a repeat biopsy. |
| Primary Tumour Management | Ta-T1 Disease:• Surgical Options: Wide local excision of lesions on the glans or a glans resurfacing procedure is recommended, with reconstruction using a split-thickness skin graft (SSG) or penile shaft skin.• Specialised Surgery: Mohs micrographic surgery may be performed for low-grade T1 lesions, but only if the appropriate clinical set-up, including a pathologist, is available.• Radiotherapy Alternative: Brachytherapy is an alternative to surgery for pT1 disease; patients should be referred to specialist centres for multidisciplinary consideration. |
| Primary Tumour Management | T2-T4 Disease: • T2 on Glans: Glansectomy with or without distal urethrectomy and SSG reconstruction is recommended. • T3-T4 (Corpus Cavernosum Invasion): Partial or total penectomy with perineal urethrostomy is recommended. • Radiotherapy Alternative (T1-T2): In selected patients with low-volume T1-T2 disease, brachytherapy is an option in specialist centres. |
| Primary Tumour Management | Radiotherapy (RT) for Primary Disease: • General: Physicians in specialist centres may recommend LDR or HDR brachytherapy, especially if surgery is not an option. • External Beam RT (EBRT): A typical course is 66-74 Gy in 2 Gy fractions, five times per week. • Brachytherapy (HDR): For intact tumours, the most frequently proposed schedule is 42-45 Gy in 12-14 fractions. The usual dose can also be 35 Gy in nine fractions over 5 days. • Brachytherapy (LDR/PDR): The usual dose is 60-65 Gy delivered continuously (LDR) or in hourly pulses (PDR) over 5 days. |
| Regional Lymph Node Management | cN0 (Non-palpable) Disease: • Low-Risk (pTa/pTis and pT1G1): Clinical surveillance of inguinal nodes is recommended. • Intermediate/High-Risk (pT1G2, pT1-4G3/G4 or lymphovascular invasion): Dynamic sentinel lymph node biopsy (DSLNB) should be performed. If positive, proceed to radical inguinal lymphadenectomy. |
| Regional Lymph Node Management | cN1-2 (Palpable) Disease: Radical inguinal lymphadenectomy is recommended to remove superficial and deep inguinal lymph nodes, with preservation of the long saphenous vein and fascia lata where possible. |
| Regional Lymph Node Management | cN3 (Bulky/Ulcerated) Disease: Neoadjuvant chemotherapy (ChT) with triplet regimens (e.g., TIP or TPF) should be discussed for eligible patients with fixed nodes. Responders should undergo ipsilateral radical inguinal lymphadenectomy, with or without pelvic lymphadenectomy. |
| Regional Lymph Node Management | Pelvic Lymph Node Dissection (PLND): Unilateral PLND is recommended for patients with two or more ipsilateral inguinal metastases, a metastasis with a diameter of ≥30 mm, or extranodal extension. |
| Regional Lymph Node Management | Salvage Inguinal Lymph Node Dissection (ILND): Salvage ILND with myocutaneous flap reconstruction may be recommended for recurrent inguinal disease. It should be part of a multimodal treatment plan that includes neoadjuvant or adjuvant ChT. |
| Systemic Therapy | Locally Advanced Disease (Adjuvant ChT): For patients with pN2 and pN3 disease following lymph node dissection (LND), adjuvant ChT should be considered. |
| Systemic Therapy | Locally Advanced Disease (Adjuvant RT): For patients with pN3 disease, consider adjuvant RT with at least 50 Gy in 2 Gy fractions (or a biological equivalent dose in 1.8 Gy fractions) in combination with adjuvant ChT. Higher doses up to 66 Gy have also been recommended. |
| Systemic Therapy | Metastatic Disease (First-Line): Treatment options include systemic ChT with platinum-based combination regimens (e.g., cisplatin-5FU, carboplatin-paclitaxel, TIP, or TPF), depending on patient comorbidities, fitness, and ECOG PS. Clinical trial enrolment is strongly recommended. |
| Systemic Therapy | Metastatic Disease (Subsequent Lines): For patients with no response or disease progression, consider second-line systemic ChT, RT for local control, best supportive care, or a clinical trial. |
| Systemic Therapy | Metastatic Disease (Molecularly Selected): • Biomarker-selected clinical trials should be considered where available. • In selected patients whose tumours have a high TMB, MSI-H, or dMMR, the use of anti-PD-L1 immunotherapy can be considered. |
| Recurrent Disease Management | Local Recurrence: • Non-invasive: Salvage penile-sparing options can be considered. • Invasive: Invasion of the corpora cavernosa warrants partial or total penectomy. |
| Recurrent Disease Management | Regional Recurrence: For recurrences in the inguinal and pelvic lymph nodes, consider systemic ChT, EBRT, surgery, or a combination thereof. |
| Follow-up Care | Frequency & Duration: Close follow-up every 3-4 months is required for the first 2 years following primary surgery to detect local recurrence. |
| Follow-up Care | Procedures: Follow-up should include clinical examination and imaging. Imaging may consist of US of the inguinal LNs for patients post-DSLNB or regular CT surveillance for patients post-radical inguinal lymphadenectomy for pN+ disease. |
| Follow-up Care | Supportive Care: Regular follow-up should provide psychological support and address sexual and urinary dysfunction, as well as lymphoedema-related complications. |

**Extracted recommendations from NCCN Clinical Practice Guidelines in Oncology (NCCN Guidelines): Penile Cancer**

| Aspect of Care | Recommendation |
| --- | --- |
| Primary Treatment | For Penile Intraepithelial Neoplasia (PeIN) or Ta Disease: • Treatment options include: Topical therapy, Wide local excision, Laser therapy (category 2B), Complete glansectomy (category 2B), or Mohs surgery in select cases (category 2B). |
| Primary Treatment | For Pathologic Stage pT1a: • Treatment options include: Wide local excision, Partial penectomy, Glansectomy in select cases, Mohs surgery in select cases (category 2B), Laser therapy (category 2B), or Radiation Therapy (RT) (category 2B). |
| Primary Treatment | For Pathologic Stage pT1b: • Treatment options include: Wide local excision, Partial penectomy, Total penectomy, RT (category 2B), or Chemo/RT (category 3). |
| Primary Treatment | For T2 or Greater: • Treatment options include: Partial penectomy, Total penectomy, RT (category 2B), or Chemo/RT (category 3). |
| Primary Treatment (Topical) | For PeIN or Ta disease: • Imiquimod 5% cream: Apply at night three times per week for 4–16 weeks. • 5-FU cream 5%: Apply twice daily for 2–6 weeks. |
| Primary Treatment (Surgical) | For high-grade primary tumors: Partial penectomy is standard, provided a functional stump with negative margins can be achieved. If not possible, a total penectomy should be performed. • For tumors with invasion into the corpora cavernosum: Partial or total penectomy is necessary. • Intraoperative frozen sections are recommended to determine negative margins. |
| Primary Treatment (Surgical - Organ-Sparing) | For Wide Local Excision (Stage PeIN, Ta, T1): Complete excision with wide negative margin is needed. If margins are positive, re-resection may be considered. • For tumors of the shaft: May be treated with wide local excision with or without circumcision. • For tumors of the distal prepuce: Circumcision alone may be reasonable. |
| Primary Treatment (Surgical - Organ-Sparing) | For Glansectomy (Stage PeIN, Ta, T1 on glans/prepuce): • For PeIN or Ta disease, a complete glansectomy is a category 2B recommendation. • For T1 G1-2 disease, glansectomy is not recommended unless required to ensure complete tumor eradication with negative margins. |
| Primary Treatment (Surgical - Organ-Sparing) | For Mohs Micrographic Surgery (Category 2B): An alternative to wide local excision in select cases. It may be preferable for a small superficial lesion on the proximal shaft to avoid total penectomy. |
| Primary Treatment (Radiotherapy) | For T1–2, N0 tumors <4 cm: After circumcision, options include: Brachytherapy alone (category 2B, performed with interstitial implant), EBRT (total dose 65–70 Gy), or EBRT with concurrent chemotherapy (category 3). |
| Primary Treatment (Radiotherapy) | For T1–2, N0 tumors ≥4 cm: After circumcision, options include: EBRT with concurrent chemotherapy (total dose 65–70 Gy) or Brachytherapy alone (category 2B) in select cases with careful post-treatment surveillance. |
| Primary Treatment (Radiotherapy) | For T3–4 or N+ (surgically unresectable): After circumcision, EBRT with concurrent chemotherapy (total dose 60-70 Gy) to the whole penile shaft, pelvic lymph nodes, and bilateral inguinal lymph nodes. |
| Lymph Node Management (Non-Palpable Nodes) | For Low-Risk Primary Lesion (PeIN, Ta, T1a): Surveillance of the inguinal lymph nodes is recommended. |
| Lymph Node Management (Non-Palpable Nodes) | For Intermediate/High-Risk Primary Lesion (T1b, or any T2 or greater): • Treatment: Bilateral inguinal lymph node dissection (ILND) or Bilateral dynamic sentinel node biopsy (DSNB). • Additional Clarifications: DSNB is recommended only if the treating physician has experience. If DSNB is positive, ILND is recommended. Prophylactic EBRT (category 2B) to inguinal nodes can be considered for patients who are not surgical candidates or decline surgery. |
| Lymph Node Management (Palpable, Non-Bulky Nodes) | For Unilateral Mobile Lymph Node(s) <4 cm: • Low-Risk Primary: Percutaneous lymph node biopsy. If negative, options are excisional biopsy or surveillance. • High-Risk Primary or Positive Biopsy: Bilateral ILND is recommended. Alternatively, consider neoadjuvant TIP chemotherapy followed by ILND. |
| Lymph Node Management (Palpable, Bulky Nodes) | For Unilateral Mobile Lymph Nodes ≥4 cm: • After a positive percutaneous biopsy, the preferred treatment is Neoadjuvant TIP chemotherapy followed by ILND (consider PLND). • For patients not eligible for TIP, ILND (preferred, consider PLND) is an option. |
| Lymph Node Management (Palpable, Bulky Nodes) | For Unilateral Fixed or Bilateral (Fixed or Mobile) Lymph Nodes: • After a positive percutaneous biopsy, neoadjuvant TIP chemotherapy is recommended. • If response to chemo: ILND (preferred) and PLND (preferred). • If no response: Proceed to management for metastatic/progressive disease. • If not eligible for neoadjuvant chemo: ILND (preferred) and PLND (preferred) or RT or Chemo/RT. |
| Lymph Node Management (Enlarged Pelvic Nodes) | If percutaneous biopsy is positive and patient is a surgical candidate: Neoadjuvant TIP chemotherapy. If there is a stable or clinical response, consolidation surgery (bilateral superficial and deep ILND and unilateral/bilateral PLND) is recommended. • If percutaneous biopsy is positive and patient is a non-surgical candidate: Chemo/RT. |
| Lymph Node Management (Surgical Principles) | Pelvic Lymph Node Dissection (PLND): • Should be considered for patients with ≥2 positive inguinal nodes on the ipsilateral ILND site or with extranodal extension. • A bilateral PLND should be considered for patients with ≥4 total positive inguinal nodes. |
| Adjuvant Therapy (Post-Lymphadenectomy) | For pN2-3 disease: Consider Pelvic lymph node dissection (PLND) ± adjuvant RT/chemotherapy (Cat 2B), OR Chemo/RT (Cat 2B), OR Chemotherapy (Cat 2B). |
| Adjuvant Therapy (Post-Lymphadenectomy) | After neoadjuvant chemo and ILND with ≥2 positive nodes or extranodal extension: Adjuvant chemotherapy (if not already given) and/or adjuvant RT (if pelvic nodes are positive) or Chemo/RT (category 2B). |
| Adjuvant Therapy (Post-Lymphadenectomy) | For high-risk features (pelvic node metastases, extranodal extension, bilateral inguinal nodes, ≥4 cm tumor in nodes): If not given preoperatively, 4 courses of TIP chemotherapy is reasonable. Adjuvant EBRT or Chemo/RT can also be considered. |
| Adjuvant Therapy (Radiotherapy) | For positive margins after penectomy: Postsurgical EBRT (45–60 Gy). If gross disease remains, treat as T3-4/N+. • For positive inguinal/pelvic lymph nodes: Recommended for palpable bulky nodes. Consider for pN2-3 disease (Cat 2B) or local recurrence. Involves EBRT to nodes (45–50.4 Gy) with a boost to gross disease (65–70 Gy). |
| Systemic Therapy (Metastatic/Recurrent) | First-Line Therapy: • Preferred: TIP (paclitaxel, ifosfamide, cisplatin). • Other Recommended: 5-FU + cisplatin; 5-FU + cisplatin + pembrolizumab followed by pembrolizumab maintenance; 5-FU + carboplatin + pembrolizumab followed by pembrolizumab maintenance. • Not Recommended: Bleomycin-containing regimens. |
| Systemic Therapy (Metastatic/Recurrent) | Subsequent-Line Therapy: • Preferred: Clinical trial. OR Pembrolizumab for unresectable/metastatic MSI-H/dMMR or TMB-H (≥10 mut/Mb) tumors that have progressed after prior treatment. • Useful in Certain Circumstances: Paclitaxel or Cetuximab. • Other: Any tumor-agnostic therapies based on NGS results. |
| Management of Recurrence | For recurrence of penile lesion after initial penile-sparing treatment: Treat according to the new recurrence stage (per primary treatment guidelines). |
| Management of Recurrence | For local recurrence in inguinal region with no prior RT/lymphadenectomy: • Single, mobile, <4 cm node: Percutaneous biopsy. If positive, perform ILND. • Fixed node, ≥4 cm node, or cN2/N3 disease: Manage as palpable bulky primary disease. |
| Management of Metastatic Disease | After initial systemic chemotherapy: • If complete/partial response or stable disease: Consolidation surgery (bilateral superficial and deep ILND and unilateral/bilateral PLND). • If no response/disease progression: Consider subsequent-line systemic therapy, RT for local control, clinical trial, or best supportive care. |
| Palliative Care | For palliative intent: Consider a palliative RT dose of 30 Gy in 10 fractions. |
| Follow-up Care (Surveillance) | After Topical/Local Therapy: Clinical exam every 3 mo (Years 1-2), every 6 mo (Years 3-5), every 12 mo (Years 5-10). |
| Follow-up Care (Surveillance) | After Partial/Radical Penectomy: Clinical exam every 6 mo (Years 1-2), every 12 mo (Years 3-10). |
| Follow-up Care (Surveillance) | After Node Treatment (pN0, N1): Clinical exam, CT abdomen/pelvis, and chest radiograph every 6 mo (Years 1-2), then every 12 mo (Years 3-4). |
| Follow-up Care (Surveillance) | After Node Treatment (pN2, N3): Clinical exam and CT of chest/abdomen/pelvis every 3 mo (Year 1), then every 6 mo (Years 2-4). |
| Follow-up Care (Surveillance) | Imaging for abnormal findings: If a clinical exam is abnormal, or for patients with obesity or prior inguinal surgery, consider ultrasound, CT, or MRI of the inguinal region. |

# Section 2: Unique and Unified Treatment Recommendations

## Prompt

Prompt used to instruct the LLM Google’s Gemini-2.5-Pro to consolidate previously extracted recommendations from all five guidelines into harmonized items.

You are given a list of treatment recommendations for penile cancer. Your task is to identify and remove duplicates — that is, recommendations that convey the same clinical advice or intent, even if worded differently.

However, do not merge or group recommendations that represent distinct treatments, approaches, or clinical decisions, even if they appear under a shared heading or are closely related.

If multiple options are listed under a broader category (e.g., organ-sparing surgery), treat each bullet or treatment modality as a potentially distinct recommendation unless two entries are clearly semantically equivalent.

When removing duplicates, always retain the most comprehensive, specific, or medically precise version. But do not combine multiple unique recommendations into a single, summarized entry.

When in doubt, err on the side of treating treatments as separate unless they are clearly interchangeable.

When duplicates are merged, list all the sources that provided this unified recommendation in the "Sources" column.

If a recommendation is unique (not merged), the "Sources" column should list only the source where it appeared.

If multiple recommendations are merged, the "Sources" column should contain all relevant sources, separated by semicolons.

## Unique and Unified Treatment Recommendations

| Aspect of Care | Recommendation | Sources |
| --- | --- | --- |
| Diagnosis & Staging | Definitive diagnosis must be achieved through tissue sampling/excision. A pre-treatment biopsy of the primary lesion is required, especially when malignancy is not clinically obvious or when non-surgical treatment (e.g., topical agents, laser, radiotherapy) is planned. | AUA2020; DGU2020; EAU-ASCO2025 |
| Diagnosis & Staging | A thorough physical examination of the primary lesion is required to assess and record its size, location, morphology, and involvement of adjacent structures like the corporal body or urethra. | AUA2020; ESMO2024 |
| Diagnosis & Staging | A physical examination of both groins must be performed to palpate for lymphadenopathy. The number, laterality, and characteristics (e.g., mobile, fixed) of any palpable or suspicious inguinal nodes should be recorded. | AUA2020; EAU-ASCO2025 |
| Diagnosis & Staging | Perform magnetic resonance imaging (MRI) or penile ultrasound (US) of the primary tumor when there is uncertainty regarding corporal invasion (T3) and/or the feasibility of organ-sparing surgery. | AUA2020; EAU-ASCO2025; ESMO2024 |
| Diagnosis & Staging | Computerized tomography (CT) of the chest, abdomen, and pelvis is performed to evaluate for distant metastatic spread. For high-risk or node-positive disease, ¹⁸F-FDG-PET/CT can also be used. | AUA2020; EAU-ASCO2025; ESMO2024 |
| Diagnosis & Staging | Cross-sectional imaging (CT, PET/CT) for primary staging should generally be reserved for T3/T4 tumors or in cases of positive lymph node status. | DGU2020 |
| Diagnosis & Staging | For superficial, non-invasive penile carcinomas (Carcinoma in situ), a fluorescence-diagnostic technique can be used to better determine the extent of the lesion. | DGU2020 |
| Diagnosis & Staging | For clinically node-negative (cN0) low-risk tumors (PeIN, pTa, pT1a G1), surveillance of the inguinal lymph nodes is recommended instead of operative staging. | DGU2020; ESMO2024; NCCN2025 |
| Diagnosis & Staging | For clinically node-negative (cN0) intermediate-risk tumors (T1a G2), surveillance may be discussed as an alternative to surgical staging with patients who are willing to comply with strict follow-up. | EAU-ASCO2025 |
| Diagnosis & Staging | For clinically node-negative (cN0) intermediate/high-risk tumors (≥pT1b, or high grade), invasive surgical lymph node staging is recommended. | DGU2020; EAU-ASCO2025; ESMO2024; NCCN2025 |
| Diagnosis & Staging | The preferred method for surgical staging in cN0 patients is dynamic sentinel node biopsy (DSNB). If DSNB is unavailable or not feasible, modified inguinal lymph node dissection (ILND) is an alternative. If DSNB is positive, a completion ILND is recommended. | DGU2020; EAU-ASCO2025; ESMO2024; NCCN2025 |
| Diagnosis & Staging | If DSNB is planned, perform inguinal ultrasound (US) first, with fine needle aspiration cytology (FNAC) of any sonographically abnormal lymph nodes. | EAU-ASCO2025 |
| Diagnosis & Staging | In cN0 patients undergoing staging, frozen section analysis should not be performed on tissue from a modified inguinal lymphadenectomy or DSNB. | DGU2020 |
| Diagnosis & Staging | For clinically node-positive (cN+) disease, an (image-guided) biopsy of the suspicious node is required to confirm metastasis before initiating treatment. | EAU-ASCO2025; NCCN2025 |
| Primary Treatment (General Principles) | Patients with early-stage, low-grade, and non-invasive tumors should be offered organ- and function-preserving therapy, depending on the tumor stage and differentiation grade. | AUA2020; DGU2020; EAU-ASCO2025 |
| Primary Treatment (General Principles) | The goal of surgical treatment is complete excision with negative margins (R0). A margin of 5 mm for G1/G2 tumors and 10 mm for G3 tumors is suggested. In case of a positive surgical margin, a re-excision should be performed. | AUA2020; DGU2020 |
| Primary Treatment (General Principles) | Offer patients a balanced and individualized discussion on the benefits and harms of possible treatment options. Inform them of the higher risk of local recurrence when using organ-sparing treatments compared to amputative surgery. | EAU-ASCO2025 |
| Primary Treatment (Topical Therapy) | Topical therapy with 5-fluorouracil (5-FU) or imiquimod can be offered to patients with biopsy-confirmed superficial disease (PeIN/Tis, Ta). Patients should be informed about limited efficacy and potential off-label use. | AUA2020; DGU2020; EAU-ASCO2025; ESMO2024; NCCN2025 |
| Primary Treatment (Topical Therapy) | If topical treatment fails, it should not be repeated. | DGU2020; EAU-ASCO2025 |
| Primary Treatment (Topical Therapy) | Example regimens for topical therapy include: • Imiquimod 5% cream: Apply at night three times per week for 4–16 weeks. • 5-FU cream 5%: Apply twice daily for 2–6 weeks. | NCCN2025 |
| Primary Treatment (Topical Therapy) | Topical therapy is ineffective for more advanced stages of penile cancer. | AUA2020 |
| Primary Treatment (Laser Therapy) | Laser ablation (e.g., CO₂ or Nd:YAG laser) can be used for biopsy-confirmed superficial or early-stage tumors (PeIN/Tis, Ta, T1). | AUA2020; DGU2020; EAU-ASCO2025; ESMO2024; NCCN2025 |
| Primary Treatment (Laser Therapy) | A surgical margin of 5 mm is considered oncologically safe for laser therapy. | AUA2020 |
| Primary Treatment (Post-Therapy Biopsy) | After topical or laser therapy, a control biopsy should be performed to verify local tumor control, especially in cases of clinical doubt. | DGU2020; EAU-ASCO2025; ESMO2024 |
| Primary Treatment (Surgical) | Organ-sparing surgery options (circumcision, wide local excision, glansectomy, Mohs) are offered for lesions confined to the glans and prepuce (PeIN, Ta, T1-T2) in patients willing to comply with strict follow-up. | EAU-ASCO2025; NCCN2025 |
| Primary Treatment (Surgical) | Intra-operative frozen section analysis of resection margins should be performed to confirm negative margins, especially in cases of doubt. | EAU-ASCO2025; NCCN2025 |
| Primary Treatment (Surgical) | Circumcision can be a curative treatment for tumors confined to the prepuce (PeIN/Tis, Ta). | AUA2020; DGU2020; ESMO2024; NCCN2025 |
| Primary Treatment (Surgical) | When using radiotherapy or topical therapy for a preputial lesion, a circumcision should be performed first. | DGU2020; NCCN2025 |
| Primary Treatment (Surgical) | Wide local excision (WLE) is an option for early-stage tumors (PeIN, Ta, T1). Complete excision with wide negative margins is required. | DGU2020; ESMO2024; NCCN2025 |
| Primary Treatment (Surgical) | Mohs micrographic surgery can be considered as an alternative to WLE for select, low-grade, superficial lesions, particularly on the glans or distal penis, if the appropriate expertise is available. | AUA2020; ESMO2024; NCCN2025 |
| Primary Treatment (Surgical) | Glansectomy (partial or total) or glans resurfacing are options for tumors located on the glans penis (PeIN, Ta, T1-T2). Reconstruction may be performed with a split-thickness skin graft. | AUA2020; DGU2020; ESMO2024; NCCN2025 |
| Primary Treatment (Surgical) | Partial or total penectomy is recommended for patients with higher stage tumors (T2, T3, T4), particularly with invasion of the corpora cavernosa, unfavorable histopathology, or for those not suitable for organ-sparing approaches. | AUA2020; DGU2020; EAU-ASCO2025; ESMO2024; NCCN2025 |
| Primary Treatment (Radiotherapy) | Radiotherapy (often brachytherapy) can be an alternative to surgery for selected patients with T1-T2 tumors (<4 cm), particularly those who are unfit for surgery. Patients should be informed of a potentially lower local control rate compared to surgery. | AUA2020; DGU2020; EAU-ASCO2025; ESMO2024; NCCN2025 |
| Primary Treatment (Radiotherapy) | For T3–4 or N+ surgically unresectable disease, consider EBRT with concurrent chemotherapy to the penis and regional lymph nodes. | NCCN2025 |
| Primary Treatment (Radiotherapy) | Specific radiotherapy doses may include: • EBRT: 66-74 Gy in 2 Gy fractions. • Brachytherapy (HDR): 42-45 Gy in 12-14 fractions. • Brachytherapy (LDR/PDR): 60-65 Gy delivered continuously over ~5 days. | ESMO2024 |
| Primary Treatment (Multimodal) | For non-resectable, locally advanced primary tumors (T4), consider induction/neoadjuvant chemotherapy followed by surgery for responders, or chemo-radiotherapy. | DGU2020; EAU-ASCO2025 |
| Recurrent Disease | Local recurrence after organ-sparing surgery should be treated according to the principles of primary tumor management. Small, non-invasive recurrences can be treated with salvage organ-sparing options. | DGU2020; EAU-ASCO2025; ESMO2024; NCCN2025 |
| Recurrent Disease | For large local recurrences or those with invasion of the corpora cavernosa, partial or total penectomy is warranted. | AUA2020; EAU-ASCO2025; ESMO2024 |
| Recurrent Disease | After radiotherapy, local recurrence should be treated with surgical salvage therapy. | DGU2020 |
| Recurrent Disease | After topical or laser therapy, local recurrence should be treated with a different treatment modality. | DGU2020 |
| Recurrent Disease | Regional lymph node recurrence after initial surveillance should be managed according to the same principles as primary lymph node metastases (i.e., with ILND +/- systemic therapy). | DGU2020; ESMO2024; NCCN2025 |
| Recurrent Disease | Regional lymph node recurrence after a prior radical lymphadenectomy should be managed with a multimodal approach, including neoadjuvant chemotherapy, surgery, and/or radiotherapy. | DGU2020; ESMO2024 |
| Lymph Node Management | Following circumcision for a primary tumor, patients with moderately or poorly differentiated tumors (grades 2 and 3) will require inguinal node dissection. | AUA2020 |
| Lymph Node Management | For palpable, mobile inguinal nodes (cN1-cN2), inguinal lymph node dissection (ILND) is recommended. The dissection may be fascial-sparing or radical, with preservation of the saphenous vein where possible. | DGU2020; EAU-ASCO2025; ESMO2024; NCCN2025 |
| Lymph Node Management | If intraoperatively suspicious nodes are removed during surgery for cN+ disease, frozen section analysis should be performed. If positive, an ipsilateral radical lymphadenectomy should be completed in the same session. | DGU2020 |
| Lymph Node Management | For bulky, fixed, or extensive inguinal nodes (cN3), neoadjuvant chemotherapy (NAC) with a cisplatin- and taxane-based regimen is preferred over up-front surgery. | DGU2020; EAU-ASCO2025; ESMO2024; NCCN2025 |
| Lymph Node Management | Minimally-invasive ILND for cN1–2 disease should only be offered as part of a clinical trial. It is contraindicated for cN3 disease. | EAU-ASCO2025 |
| Lymph Node Management | Following a response to neoadjuvant chemotherapy, patients should undergo consolidative surgery, including radical inguinal lymphadenectomy and possible pelvic lymphadenectomy. | DGU2020; EAU-ASCO2025; ESMO2024; NCCN2025 |
| Lymph Node Management | Ipsilateral pelvic lymph node dissection (PLND) is recommended for patients with ≥2-3 positive inguinal nodes on one side or with extranodal extension. | DGU2020; EAU-ASCO2025; ESMO2024; NCCN2025 |
| Systemic Therapy | Recommended neoadjuvant chemotherapy regimens are cisplatin- and taxane-based triplets, such as TIP (Paclitaxel/Ifosfamide/Cisplatin) or TPF (Cisplatin/Paclitaxel/5-Fluorouracil). | DGU2020; EAU-ASCO2025; ESMO2024; NCCN2025 |
| Systemic Therapy | Adjuvant chemotherapy should be considered for patients with pN2 or pN3 disease following lymph node dissection. | DGU2020; EAU-ASCO2025; ESMO2024; NCCN2025 |
| Systemic Therapy | Adjuvant radiotherapy (with or without chemo-sensitization) should be offered or considered for patients with high-risk pathological findings such as pN2/N3 disease or extranodal extension. | DGU2020; EAU-ASCO2025; ESMO2024; NCCN2025 |
| Systemic Therapy | Definitive radiotherapy (with or without chemo sensitization) can be offered to patients who are unwilling or unable to undergo surgery. | EAU-ASCO2025 |
| Systemic Therapy | For first-line treatment of metastatic disease, platinum-based combination chemotherapy is the preferred approach. Recommended regimens include TIP, TPF, or cisplatin + 5-FU. | DGU2020; EAU-ASCO2025; ESMO2024; NCCN2025 |
| Systemic Therapy | Bleomycin-containing chemotherapy regimens should not be used due to the risk of pulmonary toxicity. | DGU2020; EAU-ASCO2025; NCCN2025 |
| Systemic Therapy | For patients with progressive disease, enrollment in a clinical trial is the preferred option for subsequent-line therapy. | EAU-ASCO2025; ESMO2024; NCCN2025 |
| Systemic Therapy | Subsequent-line therapy options may include paclitaxel monotherapy or, for biomarker-selected tumors (MSI-H, dMMR, TMB-H), immunotherapy such as pembrolizumab. | DGU2020; ESMO2024; NCCN2025 |
| Palliative & Supportive Care | Patients with metastatic disease or requiring multimodal therapy should be discussed in an interdisciplinary tumor board. | DGU2020 |
| Palliative & Supportive Care | Palliative radiotherapy can be offered for symptom control in advanced disease (e.g., 30 Gy in 10 fractions). | EAU-ASCO2025; NCCN2025 |
| Palliative & Supportive Care | For chronic lymphedema resulting from treatment, a complex decongestive therapy (lymphatic drainage, compression therapy, and skin care) should be performed. | DGU2020 |
| Follow-up & Survivorship | Close, regular, and risk-stratified follow-up is crucial after treatment, especially for the first 2-5 years, to detect recurrences and functional disorders. | AUA2020; DGU2020; EAU-ASCO2025; ESMO2024 |
| Follow-up & Survivorship | A general follow-up schedule is every 3-4 months for the first 2 years, then less frequently. Follow-up should extend for at least 5 years. | AUA2020; EAU-ASCO2025; ESMO2024 |
| Follow-up & Survivorship | Specific follow-up schedules after laser therapy are: • CO2 Laser: After 2 months, then every 3 months for 3 years, then biannually. • Nd:YAG Laser: Every 3 months for 2 years, then biannually. | AUA2020 |
| Follow-up & Survivorship | Specific follow-up schedules based on treatment: • Organ Preservation: Every 3 mo (Yrs 1-2), every 6 mo (Yrs 3-5). • Total Penectomy: Every 3 mo (Yrs 1-2), annually (Yrs 3-5). | DGU2020 |
| Follow-up & Survivorship | Patients should be taught self-examination for early detection of recurrence and given easy access back to the clinic if concerns arise. | DGU2020; EAU-ASCO2025 |
| Follow-up & Survivorship | Penile cancer care should be delivered by an extended multi-disciplinary team including specialists in urology, oncology, nursing, pathology, radiology, psychology, and lymphedema therapy. | EAU-ASCO2025 |
| Follow-up & Survivorship | Patients should be offered psychological support and counseling, and physicians should discuss the psychological and functional (urinary, sexual) impact of the disease and its treatments. | EAU-ASCO2025; ESMO2024 |
| Follow-up & Survivorship | Patients should be assessed for lymphedema at follow-up and referred to lymphedema therapists early if it develops. | DGU2020; EAU-ASCO2025; ESMO2024 |

# Section 3: Stance classification

## Prompt

The following prompt instructs the large language model to analyse the stance of each guideline toward the harmonized recommendation items (Supplementary Material 2).

You are given two texts:

- Treatment Recommendation – a proposed approach or method for treating penile cancer.
- Guideline – a comprehensive clinical guideline on penile cancer treatment.

Your task is to analyze the stance of the guideline toward the recommendation and classify it using one of the following labels:

- R (Recommended) – The guideline supports or aligns with the treatment recommendation.
- DR (Different Recommendation) – The guideline suggests a different treatment or approach compared to the recommendation.
- NA (Not Addressed) – The guideline does not mention or address the treatment recommendation at all.

Additionally, provide a concise justification (2–4 sentences) summarizing why you selected that stance, referencing key elements from both the recommendation and the guideline.

**Treatment Recommendation**

RECOMMENDATION_TEXT

**Guideline (context)**

GUIDELINE_TEXT

**Formatting Instructions**

Provide a classification code that can be one out of [R (Recommended); DR (Different recommendation); NA (Not Addressed)] and a summary that justifies that classification referencing key elements from both the recommendation and the guideline.

## Output Classification

The following table shows the stance of the individual guidelines on the harmonized recommendation items. Three categories were defined: R (Recommended), DR (Different recommendation), or NA (Not addressed). For more intuitive accessibility, the actual output table was divided into sections and grouped without changing the content.

**1. Diagnosis & Staging**

1.1 Definitive diagnosis must be achieved through tissue sampling/excision. A pre-treatment biopsy of the primary lesion is required, especially when malignancy is not clinically obvious or when non-surgical treatment (e.g., topical agents, laser, radiotherapy) is planned.

| AUA2020 | R | The guideline directly supports the recommendation for definitive diagnosis via tissue sampling. Under the "DIAGNOSIS" section, the guideline explicitly states, "Definitive diagnosis is achieved through tissue sampling/excision..." Furthermore, it reinforces the importance of this step before non-surgical treatment, noting in its conclusion that an "Accurate pathological diagnosis is necessary before initiating therapy" with topical agents, laser, or surgery. |
| --- | --- | --- |
| DGU2020 | R | The guideline aligns with the treatment recommendation by stating that a histological diagnosis is essential ("unabdinglich") and that a simple visual diagnosis is insufficient (p. 38). Recommendation 6.1 states that for unclear penile lesions, an early histological diagnosis should be obtained (p. 37). Furthermore, section 4.4.5 emphasizes that a biopsy with a sufficient amount of tissue is required for a reliable diagnosis before initiating definitive therapy (p. 32). |
| EAU-ASCO2025 | R | The guideline strongly supports the treatment recommendation. Section 5.3 explicitly states with a "Strong" rating to "Obtain a pre-treatment biopsy of the primary lesion when malignancy is not clinically obvious, or when non-surgical treatment of the primary lesion is planned (e.g., topical agents, laser, radiotherapy)." This directly aligns with the recommendation's requirement for a pre-treatment biopsy under the same conditions. Furthermore, Section 5.1.3 reinforces this by stating histological confirmation is necessary to guide management when non-surgical treatments are planned. |
| ESMO2024 | R | The guideline supports the recommendation for tissue biopsy to confirm a diagnosis. In the "Diagnosis" section, it explicitly states, "Incisional or excisional biopsies of suspected penile cancer should be carried out to confirm a histological diagnosis" (p. 1). This statement directly aligns with the recommendation that a definitive diagnosis must be achieved through tissue sampling or excision prior to treatment. |
| NCCN2025 | R | The NCCN guideline aligns with the recommendation by emphasizing the necessity of a histologic diagnosis. On page PN-1, under "PRIMARY EVALUATION" for a suspicious penile lesion, the guideline explicitly lists "Histologic diagnosis" to be achieved via "Punch, excisional, or incisional biopsy." The discussion section (page MS-5) further reinforces this, stating that a histologic diagnosis via biopsy is "paramount in determining the treatment algorithm," which directly supports the recommendation's stance on mandatory pre-treatment tissue sampling. |

1.2 A thorough physical examination of the primary lesion is required to assess and record its size, location, morphology, and involvement of adjacent structures like the corporal body or urethra.

| AUA2020 | R | The AUA guideline explicitly supports the recommendation. In the "DIAGNOSIS" section, it states, "Diagnosis begins with a thorough physical examination. The lesion is assessed for size, location and involvement of the corporal body or urethra." This statement directly aligns with the recommendation's call for a thorough physical examination to assess the lesion's characteristics and involvement of adjacent structures. |
| --- | --- | --- |
| DGU2020 | R | The guideline aligns with the treatment recommendation. Specifically, in consensus-based recommendation 6.2 on page 37, the guideline states that the "Location, size, growth pattern and mobility of the primary tumor should be examined and documented." This directly corresponds to the recommendation's call to assess and record the lesion's size, location, morphology, and involvement of adjacent structures. |
| EAU-ASCO2025 | R | The EAU-ASCO guideline strongly supports the recommendation for a thorough physical examination of the primary lesion. Section 5.1.1 on page 15 explicitly states that "Physical examination should include inspection and palpation of the entire penis... The dimensions, anatomic location, and extent of local invasion should be noted." This is further reinforced in the summary recommendations on page 18, which gives a "Strong" rating to performing a "detailed physical examination... recording morphology, size and location of the penile lesion, including extent and invasion of penile (adjacent) structures." |
| ESMO2024 | R | The guideline strongly supports the treatment recommendation. Under the section "Assessment of the primary tumour," it explicitly states, "Clinical assessment of the primary tumour should record the size, morphology and relationship to adjacent structures..." This statement is repeated verbatim in the "Recommendations" section, directly aligning with the proposed physical examination to assess and record the lesion's characteristics. |
| NCCN2025 | R | The guideline aligns with the recommendation by emphasizing the importance of a physical examination as part of the initial workup. On page PN-1, under "PRIMARY EVALUATION," the guideline specifies assessing "Lesion characteristics," including "Diameter, location, number of lesions, morphology... relationship to other structures (submucosal, corpora spongiosa, cavernosa, and/or urethra)." This is further reinforced in the discussion on page MS-4, which states that a "good physical examination of the penile lesion(s)" is "vital to the initial management" and lists the same characteristics to be evaluated. |

1.3 A physical examination of both groins must be performed to palpate for lymphadenopathy. The number, laterality, and characteristics (e.g., mobile, fixed) of any palpable or suspicious inguinal nodes should be recorded.

| AUA2020 | R | The guideline directly supports the recommendation as a fundamental part of the diagnostic process. In the "DIAGNOSIS" section, the guideline explicitly states that "Inguinal lymph nodes must be palpated for lymphadenopathy." This aligns perfectly with the recommendation to perform a physical examination of the groins to palpate for and characterize any suspicious nodes. |
| --- | --- | --- |
| DGU2020 | R | The guideline aligns with the treatment recommendation. Guideline recommendation 6.4 states that the inguinal lymph node status must be determined and documented through clinical examination. The background information further clarifies that this examination must include a thorough palpation of the groins. It also specifies that for any palpable lymph node swellings, the number, size, and mobility must be recorded for each side, which directly corresponds to the recommendation's call to record the number, laterality, and characteristics of palpable nodes. |
| EAU-ASCO2025 | R | The guideline strongly supports the treatment recommendation. Section 5.2.1, "Physical examination," explicitly states, "Careful palpation of both groins for enlarged/pathologic inguinal LNs must be part of the initial physical examination..." It further advises that "...the number, location, size and whether the node is fixed or mobile, should be noted." This is reiterated in the summary of recommendations on page 18, which gives a "Strong" rating to performing a physical examination of both groins and recording the number, laterality, and characteristics of any palpable nodes. |
| ESMO2024 | R | The guideline's entire algorithm for managing inguinal lymph nodes (LNs) is predicated on a clinical assessment to determine if the LNs are palpable. The guideline states that management "depends on the clinical stage, which is still classified according to whether they are palpable or not" and subsequently outlines different pathways for impalpable (cN0) versus palpable (cN1-2, cN3) nodes. This aligns directly with the recommendation to perform a physical examination of the groins to palpate for lymphadenopathy and record its characteristics. |
| NCCN2025 | R | The NCCN guideline strongly supports the recommendation. The "Management of Regional Lymph Nodes" discussion (MS-9) explicitly states that evaluation of the groin is an "essential component of the metastatic workup." It further details that the clinical examination should assess for the exact characteristics mentioned in the recommendation, including "palpability, number of inguinal masses, unilateral or bilateral localization, dimensions, [and] mobility or fixation of nodes." This direct alignment confirms that a thorough physical examination of the inguinal nodes is a standard and recommended practice. |

1.4 Perform magnetic resonance imaging (MRI) or penile ultrasound (US) of the primary tumor when there is uncertainty regarding corporal invasion (T3) and/or the feasibility of organ-sparing surgery.

| AUA2020 | R | The guideline supports this recommendation. In the "DIAGNOSIS" section, the document states that imaging is often required to determine the extent of tumor invasion. It specifically mentions that Doppler ultrasound is recommended "to determine whether organ sparing techniques can be performed in potential candidates." Furthermore, it adds that "Magnetic resonance imaging...is also used for local staging in cases of organ preserving surgery," directly aligning with the recommendation to use these imaging modalities for assessing corporal invasion and the feasibility of organ-sparing approaches. |
| --- | --- | --- |
| DGU2020 | R | The guideline supports using imaging to assess tumor infiltration. Section 6.1 (p. 38) states that ultrasound ("Sonographie") can be useful for evaluating the depth of infiltration. It also identifies MRI ("Magnetresonanztherapie") as an alternative for this purpose, although it notes that this procedure is often painful and therefore not generally recommended. Furthermore, Recommendation 6.3 (p. 37) aligns by suggesting the use of cross-sectional imaging for T3/T4 tumors, which is consistent with evaluating for corporal invasion. |
| EAU-ASCO2025 | R | The guideline directly supports this recommendation. Section 5.3 includes a specific recommendation to "Perform magnetic resonance imaging (MRI) of the penis/primary tumour... when there is uncertainty regarding corporal invasion and/or the feasibility of (organ-sparing) surgery. If MRI is not available, offer ultrasound (US) as alternative option." This aligns perfectly with the proposed treatment. The rationale is further detailed in Section 5.1.2, which states that while physical examination is reliable, MRI is helpful for assessing corporal invasion (cT3) and planning organ-sparing surgery. |
| ESMO2024 | R | The guideline supports this recommendation. In the section "Assessment of the primary tumour," it states that when assessing for invasion into the corpus cavernosum, "Where there is uncertainty, magnetic resonance imaging (MRI) or penile ultrasound (US) combined with an intracavernosal injection of prostaglandin E1... can be helpful to stage the primary lesion." This is reiterated in the summary recommendations and in the diagnostic algorithm in Figure 1. |
| NCCN2025 | R | The NCCN guideline supports the use of magnetic resonance imaging (MRI) or ultrasound for assessing the primary penile tumor. The "Management of Primary Lesions" discussion section explicitly states, "MRI or ultrasound can be used to evaluate the depth of tumor invasion" (MS-4). This statement directly aligns with the recommendation's purpose of using these imaging modalities to determine the extent of corporal invasion and inform decisions about organ-sparing surgery. |

1.5 Computerized tomography (CT) of the chest, abdomen, and pelvis is performed to evaluate for distant metastatic spread. For high-risk or node-positive disease, ¹⁸F-FDG-PET/CT can also be used.

| AUA2020 | R | The guideline supports the recommendation. Under the "DIAGNOSIS" section, the guideline states, "Computerized tomography of the chest, abdomen and pelvis is typically performed to evaluate the spread of cancer to the lymph nodes and more distant locations." This aligns directly with the recommendation to use CT scans to evaluate for distant metastatic spread. |
| --- | --- | --- |
| DGU2020 | R | The guideline recommends using cross-sectional imaging (`Schnittbildverfahren`), which includes CT, to diagnose distant metastases (Section 6.3). It specifies that these methods should only be used for patients with advanced T3/T4 tumors or a positive lymph node status (Recommendation 6.3). This aligns with the treatment recommendation to perform CT of the chest, abdomen, and pelvis and to use ¹⁸F-FDG-PET/CT for high-risk or node-positive disease. |
| EAU-ASCO2025 | R | The guideline aligns with the treatment recommendation. In the recommendations table for clinically node-positive (cN+) patients on page 18, it strongly recommends to "stage the pelvis and exclude distant metastases with ¹⁸F-fluoro-2-deoxy-D-glucose positron emission tomography (¹⁸FDG-PET) computed tomography (CT) or CT of the chest and abdomen before initiating treatment." This directly supports performing CT or PET/CT to evaluate for distant metastases in node-positive patients, as stated in the recommendation. Section 5.2.3 further elaborates that while such imaging is not for cN0 patients, it is valuable in cN+ patients to exclude distant metastases. |
| ESMO2024 | R | The guideline aligns with the treatment recommendation. Under the section "Assessment of distant metastases," the guideline states that for routine staging and follow-up, "CT of the chest, abdomen and pelvis is sufficient." It further specifies that "MRI or CT fusion PET...can be used in high-risk cases," which is consistent with the recommendation's suggestion to use ¹⁸F-FDG-PET/CT for high-risk disease. |
| NCCN2025 | R | The guideline aligns with the recommendation by advising cross-sectional imaging of the chest, abdomen, and pelvis for the initial workup and staging of penile cancer, listing CT as a primary modality (PN-E). Furthermore, it specifically recommends considering FDG-PET/CT for staging in patients with suspected inguinal lymph node-positive disease (PN-E). The discussion section also confirms that PET/CT is best used as a diagnostic modality in patients with clinically node-positive (cN+) penile cancer (MS-9). |

1.6 Cross-sectional imaging (CT, PET/CT) for primary staging should generally be reserved for T3/T4 tumors or in cases of positive lymph node status.

| AUA2020 | NA | The guideline mentions that computerized tomography is "typically performed to evaluate the spread of cancer to the lymph nodes and more distant locations." However, it does not specify the clinical scenarios or tumor stages for which this imaging is indicated. The recommendation provides specific criteria (T3/T4 tumors or positive lymph nodes), which are not discussed in the guideline text. |
| --- | --- | --- |
| DGU2020 | R | The guideline aligns with the treatment recommendation. Specifically, consensus-based recommendation 6.3 on page 37 states that for primary staging of penile cancer, cross-sectional imaging and hybrid imaging methods should only be used for T3/T4 tumors or in cases of a positive lymph node status ("nur bei T3/T4 Tumoren oder positivem Lymphknotenstatus eingesetzt werden"). This directly mirrors the proposed recommendation to reserve CT and PET/CT for these specific clinical scenarios. |
| EAU-ASCO2025 | R | The EAU-ASCO guideline aligns with the recommendation to reserve cross-sectional imaging for advanced disease. For clinically node-negative (cN0) patients, the guideline states that imaging modalities like CT and PET/CT are of "limited value and are not recommended for routine use" (p. 16). In contrast, for clinically node-positive (cN+) patients, it is strongly recommended to "stage the pelvis and exclude distant metastases with... PET computed tomography (CT) or CT of the chest and abdomen" (p. 18). |
| ESMO2024 | DR | The guideline advises that CT of the chest, abdomen, and pelvis is recommended "in all cases for the assessment of distant metastases [III, A]." This contrasts with the treatment recommendation, which suggests that such imaging should be reserved for higher-risk patients (T3/T4 tumors or positive lymph node status). Although the guideline does note that MRI or CT fusion PET can be used for high-risk disease, its recommendation for routine CT staging is broader and applies to all patients, not just a select high-risk group. |
| NCCN2025 | DR | The NCCN guideline recommends cross-sectional imaging for a broader range of primary tumors than the recommendation suggests. Specifically, the guideline indicates imaging for patients with intermediate/high-risk lesions, defined as T1b and any T2 or greater tumors (PN-3), whereas the recommendation reserves it for T3/T4 tumors. However, both the guideline and the recommendation align on the use of imaging, including considering PET/CT, for patients with palpable or suspected positive lymph nodes (PN-4, PN-E). |

1.7 For superficial, non-invasive penile carcinomas (Carcinoma in situ), a fluorescence-diagnostic technique can be used to better determine the extent of the lesion.

| AUA2020 | DR | The guideline mentions "fluorescence guidance" as a technique that "some have argued for" to improve laser surgery outcomes but immediately qualifies this by stating, "more data are required to clearly elucidate the role." The primary section on diagnosis recommends established methods such as physical examination, Doppler ultrasound, MRI, and CT scans. By presenting fluorescence guidance as an investigational approach lacking sufficient data while detailing other standard diagnostic modalities, the guideline effectively recommends a different, more established approach. |
| --- | --- | --- |
| DGU2020 | R | The guideline directly supports the recommendation. Recommendation 7.5 on page 46 explicitly states that for superficial, non-invasive penile carcinomas, a fluorescence-diagnostic technique can be used to better determine the extent of the carcinoma in situ. The background information further details this, mentioning photodynamic diagnostics (PDD) with 5-aminolevulinic acid as an example of such a technique. The guideline assigns an open recommendation grade ("kann" / "can"), confirming it is an acceptable option. |
| EAU-ASCO2025 | NA | The EAU-ASCO guideline provides recommendations for the treatment of superficial non-invasive disease (PeIN, Ta), including topical therapies, laser ablation, photodynamic therapy, and surgery (Section 6.1.1). For diagnostic evaluation of the primary lesion, it discusses physical examination, MRI, and ultrasound (Section 5.1). However, the guideline does not mention or address the use of a fluorescence-diagnostic technique to better determine the extent of these superficial lesions. |
| ESMO2024 | NA | The guideline provides a detailed algorithm for the diagnostic work-up of penile cancer, including clinical assessment, MRI, and ultrasound for staging the primary tumor. For premalignant disease (PeIN or Carcinoma in situ), it discusses management options like circumcision and topical treatments. However, the guideline does not mention or address the use of a fluorescence-diagnostic technique to determine the extent of these lesions. |
| NCCN2025 | NA | The NCCN guideline does not mention the use of fluorescence-diagnostic techniques to determine the extent of superficial, non-invasive penile carcinomas (PeIN or CIS). The guideline's recommended methods for initial evaluation include a history and physical exam, lesion characterization, and histologic diagnosis via biopsy (PN-1). For the specific purpose of delineating lesions before laser therapy, the guideline mentions the perioperative application of 3%-5% acetic acid to identify acetowhite areas (PN-A), but this is a different technique. Since the specific modality of fluorescence diagnostics is not discussed, the recommendation is not addressed. |

1.8 For clinically node-negative (cN0) low-risk tumors (PeIN, pTa, pT1a G1), surveillance of the inguinal lymph nodes is recommended instead of operative staging.

| AUA2020 | R | The guideline supports the recommendation. It explicitly states that patients with moderately or poorly differentiated tumors (grades 2 and 3) require inguinal node dissection. This implies that low-risk tumors, such as the Grade 1 tumors specified in the recommendation, do not require routine operative staging. This selective approach aligns with the recommendation for surveillance of the inguinal lymph nodes in clinically node-negative, low-risk cases. |
| --- | --- | --- |
| DGU2020 | R | The guideline explicitly supports the recommendation for surveillance in low-risk, clinically node-negative patients. Recommendation 7.29 on page 62 states with a strong recommendation grade (A) that clinical surveillance ("Eine klinische Überwachung") should be performed only for patients with tumor stages pTa and pT1aG1. Additionally, Recommendation 6.6 on page 40 states that operative staging can be omitted in favor of regular clinical follow-up for patients up to category pT1a. This directly matches the proposed treatment recommendation. |
| EAU-ASCO2025 | R | The guideline supports the recommendation. On page 17, section 5.2.2.2, it defines low-risk tumors as "Well-differentiated (G1), pTa, pTis and pT1 tumours without lymphovascular/perineural invasion (pT1a)." For these patients, the guideline states that "the risk of metastases is too low to justify surgical staging." This directly aligns with the recommendation to avoid operative staging and instead perform surveillance for clinically node-negative, low-risk tumors. |
| ESMO2024 | R | The guideline fully supports the treatment recommendation. In the section "Management of cN0 disease," it explicitly states that "patients with cN0 low-risk disease should be managed with clinical surveillance." It further reinforces this in the final "Recommendations" section, specifying that "Clinical surveillance of the inguinal nodes is recommended in patients presenting with cN0 tumours with low-risk features (pTa/pTis and pT1G1)." |
| NCCN2025 | R | The guideline supports the recommendation. The "Management of Non-Palpable Inguinal Lymph Nodes" algorithm (PN-3) stratifies patients by risk based on the primary lesion. For the "Low risk (PeIN, Ta, T1a)" group, the guideline explicitly recommends "Surveillance". The discussion section (MS-15) further clarifies that patients with low-risk disease are followed with a surveillance protocol and that observation alone is sufficient. |

1.9 For clinically node-negative (cN0) intermediate-risk tumors (T1a G2), surveillance may be discussed as an alternative to surgical staging with patients who are willing to comply with strict follow-up.

| AUA2020 | DR | The treatment recommendation suggests that for intermediate-risk tumors (T1a G2), surveillance may be an alternative to surgical staging of the lymph nodes. However, the AUA guideline document explicitly states a different course of action for this patient group. On page 76, under the "SURGERY" section, the guideline specifies that "close follow-up is critical for patients with moderately or poorly differentiated tumors (grades 2 and 3), as these will require inguinal node dissection." This indicates a requirement for surgical intervention, directly contradicting the option of surveillance for Grade 2 tumors proposed in the recommendation. |
| --- | --- | --- |
| DGU2020 | DR | The guideline explicitly recommends a different management strategy. Guideline recommendation 7.29 states that clinical surveillance for clinically node-negative (cN0) patients should only be performed for tumor stages pTa and pT1aG1. The background information on page 63 further clarifies that surveillance for patients with a tumor stage of ≥ pT1G2 (which includes the T1a G2 mentioned in the treatment recommendation) "soll nicht durchgeführt werden" (should not be performed). This directly contradicts the proposal to consider surveillance for T1a G2 tumors. |
| EAU-ASCO2025 | R | The guideline directly supports the treatment recommendation. In the recommendations table on page 18, under the "Clinically node-negative (cN0)" section, it explicitly states: "In case of T1a G2 disease, also discuss surveillance as an alternative to surgical staging with patients willing to comply with strict follow-up." The guideline text on page 17 further contextualizes this by identifying pT1a G2 tumors as "intermediate-risk" and advising that the decision for surgical staging should balance the risk of metastasis against surgical morbidity on a case-by-case basis. |
| ESMO2024 | DR | The guideline explicitly recommends active treatment for patients with cN0 intermediate-risk disease, stating it is the recommended approach. Specifically, for patients with intermediate-risk disease (defined as pT1G2), the guideline recommends dynamic sentinel lymph node biopsy (DSLNB). In contrast, the treatment recommendation suggests that surveillance may be discussed as an alternative for this same patient group, which differs from the guideline's clear preference for active management. |
| NCCN2025 | R | The NCCN guideline stratifies clinically node-negative patients based on the primary lesion (PN-3). It categorizes T1a tumors as "low risk" and explicitly recommends "Surveillance" as the appropriate management for this group. Although the treatment recommendation uses the term "intermediate-risk" for T1a G2 tumors, the patient population and the recommended approach of surveillance are fully consistent with the NCCN guidelines. |

1.10 For clinically node-negative (cN0) intermediate/high-risk tumors (≥pT1b, or high grade), invasive surgical lymph node staging is recommended.

| AUA2020 | R | The AUA Update Series guideline aligns with the recommendation for invasive surgical lymph node staging in patients with high-risk penile cancer. The guideline specifically states on page 76 that for patients with "moderately or poorly differentiated tumors (grades 2 and 3)," which are considered high-risk, they "will require inguinal node dissection." This directly supports the recommendation to perform invasive surgical staging for high-grade tumors, even when they are clinically node-negative (cN0). |
| --- | --- | --- |
| DGU2020 | R | The guideline aligns with the treatment recommendation by stating that for clinically node-negative patients with tumors from category pT1b onwards, inguinal lymph nodes "should be investigated invasively" ("sollen ... invasiv untersucht werden") (Recommendation 6.5, page 40). This is a strong recommendation (Grade A) for the same intermediate/high-risk group (≥pT1b) identified in the treatment proposal. The guideline further specifies that invasive staging is recommended for high-grade tumors and stages T1b and higher due to the increased risk of micrometastases, thus fully supporting the proposed approach. |
| EAU-ASCO2025 | R | The guideline strongly supports the recommendation for invasive surgical lymph node staging in patients with clinically node-negative (cN0) high-risk penile cancer. Section 5.2.2.2 explicitly states, "surgical staging is recommended in all high-risk tumours (T1 with presence of lympho-vascular invasion, peri-neural invasion or poorly differentiated, and T2–T4 with any grade)." This is further reinforced in the summary recommendations (Section 5.3), which advise to "offer surgical LN staging to all patients at high risk of having micro-metastatic disease (T1b or higher)." The guideline's definition of high-risk tumors (T1b or higher) directly aligns with the criteria presented in the treatment recommendation. |
| ESMO2024 | R | The guideline aligns with the recommendation by stratifying clinically node-negative (cN0) patients into low-, intermediate-, and high-risk groups. It states that while low-risk cN0 patients should be managed with surveillance, "active treatment is recommended for patients with intermediate- and high-risk disease." The guideline specifically recommends invasive surgical staging via dynamic sentinel lymph node biopsy (DSLNB) for these intermediate- and high-risk cN0 patients. |
| NCCN2025 | R | The guideline supports the recommendation. On page PN-3, under "MANAGEMENT OF NON-PALPABLE INGUINAL LYMPH NODES," patients with non-palpable nodes and intermediate/high-risk primary lesions (defined as T1b or any T2 or greater) are recommended for treatment with either bilateral inguinal lymph node dissection (ILND) or bilateral dynamic sentinel node biopsy (DSNB). Both ILND and DSNB are forms of invasive surgical lymph node staging, which aligns directly with the proposed treatment recommendation. The discussion section (MS-15) further reinforces this, stating that for these patients, a "modified or radical bilateral inguinal lymphadenectomy is strongly recommended." |

1.11 The preferred method for surgical staging in cN0 patients is dynamic sentinel node biopsy (DSNB). If DSNB is unavailable or not feasible, modified inguinal lymph node dissection (ILND) is an alternative. If DSNB is positive, a completion ILND is recommended.

| AUA2020 | NA | The AUA Update Series guideline does not address the recommendation regarding surgical staging of lymph nodes. The document's scope is the treatment of the primary, early-stage penile tumor, detailing options such as topical therapy, laser ablation, and organ-sparing surgery. While it emphasizes the need to evaluate inguinal lymph nodes, it provides no specific guidance on surgical staging methods like dynamic sentinel node biopsy (DSNB) or modified inguinal lymph node dissection for clinically node-negative patients. |
| --- | --- | --- |
| DGU2020 | R | The guideline recommends invasive staging for clinically node-negative (cN0) patients with penile carcinoma from stage pT1b onwards. It explicitly lists both modified inguinal lymphadenectomy and dynamic sentinel node biopsy (DSNB) as valid procedures for this purpose (Recommendation 6.5, p. 40). This aligns with the treatment recommendation, as the guideline presents both DSNB and modified lymphadenectomy as appropriate options for surgical staging in this patient group. |
| EAU-ASCO2025 | R | The guideline strongly aligns with the treatment recommendation. In the recommendations table on page 18, under "Clinically node-negative (cN0)," it states with a "Strong" rating to "offer dynamic sentinel node biopsy (DSNB)" when surgical staging is indicated. The guideline further specifies that if DSNB is unavailable or not feasible, "offer inguinal lymph node dissection (ILND)." Additionally, Section 5.2.2.3 on page 17 explicitly states that if histopathology identifies sentinel node metastasis, "ipsilateral completion ILND is indicated," fully corroborating all aspects of the proposed recommendation. |
| ESMO2024 | R | The guideline aligns with the recommendation, stating that for intermediate- and high-risk cN0 patients, dynamic sentinel lymph node biopsy (DSLNB) is the recommended staging procedure. The guideline specifies that if metastatic nodes are found, this should be followed by a radical inguinal lymphadenectomy. It also concurs that when DSLNB is not available, a modified inguinal lymph node dissection (ILND) is a suitable alternative. |
| NCCN2025 | DR | The guideline does not establish dynamic sentinel node biopsy (DSNB) as the single preferred method for staging cN0 patients. Instead, for intermediate- and high-risk patients with non-palpable inguinal lymph nodes, the guideline presents bilateral inguinal lymph node dissection (ILND) and bilateral DSNB as equivalent alternatives, connected by an "or" (PN-3). The guideline further qualifies the use of DSNB, recommending it only when the physician has substantial experience and noting its limited practice (PN-3, MS-15). While the guideline does agree that a positive DSNB necessitates a completion ILND, it differs from the recommendation by not prioritizing DSNB over ILND as the primary choice. |

1.12 If DSNB is planned, perform inguinal ultrasound (US) first, with fine needle aspiration cytology (FNAC) of any sonographically abnormal lymph nodes.

| AUA2020 | NA | prior to a planned Dynamic Sentinel Node Biopsy (DSNB). The document's scope is limited to the treatment of the primary early-stage tumor, covering topical therapies, laser ablation, and various surgical excisions. While it broadly states that "inguinal lymph nodes must be evaluated for lymphadenopathy," it provides no details on the specific diagnostic pathway or staging procedures like DSNB or the use of ultrasound/FNAC for this purpose. |
| --- | --- | --- |
| DGU2020 | R | The guideline explicitly supports the combination of dynamic sentinel lymph node biopsy (DSNB) with ultrasound and fine needle biopsy (FNB). On page 41, it states that FNB of sonographically suspicious lymph nodes can improve the sensitivity of DSNB, citing a study where the combination increased sensitivity to 94%. Similarly, on page 64, it mentions that a possible improvement ("mögliche Verbesserung") of DSNB can be achieved with an additional ultrasound examination and targeted puncture of suspicious lymph nodes, directly aligning with the proposed recommendation. |
| EAU-ASCO2025 | R | The guideline explicitly supports the treatment recommendation. In the recommendations table for "Clinically node-negative (cN0)" patients on page 18, it states with a "Strong" rating: "If DSNB is planned, perform inguinal US first, with fine needle aspiration cytology (FNAC) of sonographically abnormal LNs." Section 5.2.2.1 further elaborates that while a negative ultrasound is unreliable for excluding metastasis, a positive US + FNAC can confirm it, reducing the need for DSNB and allowing for earlier therapeutic lymph node dissection. |
| ESMO2024 | R | The guideline aligns with the recommendation. In the section on "Management of cN0 disease," it describes a "proven protocol" for patients undergoing Dynamic Sentinel Node Biopsy (DSLNB). This protocol explicitly includes "preoperative US combined with FNAC for morphologically abnormal LNs," which is the exact procedure outlined in the treatment recommendation. |
| NCCN2025 | R | The NCCN guideline recommends the use of preoperative ultrasonography with fine-needle aspiration (FNA) cytology as a refinement to the dynamic sentinel node biopsy (DSNB) technique. In the "Dynamic Sentinel Node Biopsy" discussion on page MS-10, the guideline states that including "preoperative ultrasonography with and without fine-needle aspiration (FNA) cytology" is part of the refined technique that decreased the false-negative rate. This directly supports the treatment recommendation to perform an inguinal ultrasound with FNAC of abnormal nodes prior to a planned DSNB. |

1.13 In cN0 patients undergoing staging, frozen section analysis should not be performed on tissue from a modified inguinal lymphadenectomy or DSNB.

| AUA2020 | NA | The guideline focuses on the treatment of early-stage primary penile tumors, covering topical therapies, laser ablation, and surgical excisions like glansectomy and Mohs surgery. Although it mentions the need to evaluate and potentially dissect inguinal lymph nodes, it does not provide details on the specific intraoperative techniques for these staging procedures. The document makes no mention of dynamic sentinel node biopsy (DSNB) or the use of frozen section analysis for lymph node tissue. |
| --- | --- | --- |
| DGU2020 | R | The guideline directly supports the recommendation. Recommendation 7.33 on page 65 explicitly states that no frozen section analysis should be performed on resected tissue from a modified inguinal lymphadenectomy or a dynamic sentinel lymph node biopsy. The background text on page 66 further explains that this is because frozen section analysis in clinically unremarkable lymph nodes increases the risk of overlooking micrometastases in the definitive histology. This aligns perfectly with the proposed treatment recommendation. |
| EAU-ASCO2025 | NA | The guideline discusses the use of intra-operative frozen section analysis in Section 6.1.2.2, but only in the context of assessing resection margins of the primary tumor during organ-sparing surgery. It concludes that routine use is not recommended for that specific purpose. The guideline does not mention or provide any recommendation regarding the use of frozen section analysis on lymph node tissue from a dynamic sentinel node biopsy (DSNB) or a modified inguinal lymphadenectomy during staging for cN0 patients. |
| ESMO2024 | DR | The recommendation advises against performing frozen section analysis on tissue from a modified inguinal lymphadenectomy (ILND) or dynamic sentinel node biopsy (DSNB) in cN0 patients. However, the ESMO guideline recommends a different approach. For intermediate- and high-risk cN0 patients where DSNB is unavailable, the guideline suggests a "superficial modified inguinal lymphadenectomy with frozen section" and states that this procedure "requires intraoperative frozen section analysis." This is a direct contradiction to the provided recommendation. |
| NCCN2025 | DR | The treatment recommendation advises against performing frozen section analysis during staging procedures like modified inguinal lymphadenectomy (ILND) or DSNB in cN0 patients. The NCCN guideline directly contradicts this by stating, "A modified/superficial inguinal dissection with intraoperative frozen section is an acceptable alternative to stage the inguinal lymph nodes" (PN-3, footnote h). The guideline further reinforces this by recommending that if nodes are positive on frozen section, the surgery should be converted to a more extensive dissection, indicating frozen section is an integral part of its decision-making algorithm (MS-15). |

1.14 For clinically node-positive (cN+) disease, an (image-guided) biopsy of the suspicious node is required to confirm metastasis before initiating treatment.

| AUA2020 | NA | The AUA Update Series guideline focuses exclusively on the "Treatment of Early Stage Squamous Cell Carcinoma of the Penis," covering non-invasive and early-stage malignancies (Tis, Ta, T1). The recommendation, however, pertains to the management of clinically node-positive (cN+) disease, which represents a more advanced stage of cancer. Because the management of node-positive disease is outside the stated scope of this educational document, the guideline does not address the specific procedure of performing a biopsy to confirm metastasis in suspicious nodes before initiating treatment. |
| --- | --- | --- |
| DGU2020 | R | The guideline aligns with the treatment recommendation. In the background information for recommendation 7.35 (page 67), it is stated that for patients with palpable inguinal lymph nodes (cN+), histological or cytological confirmation is required first ("erfolgt zunächst eine histologische oder zytologische Sicherung"). The guideline specifies this can be achieved through an ultrasound-guided biopsy ("Sonographie-gestützte Biopsie") or an excisional biopsy, which supports the recommendation to confirm metastasis with a biopsy before initiating definitive treatment like radical lymphadenectomy. |
| EAU-ASCO2025 | R | The guideline explicitly supports the recommendation. In the recommendations table under "Clinically node-positive (cN+)" on page 18, it states with a "Strong" rating: "If there is a palpable/suspicious node at physical examination (cN+), obtain (image-guided) biopsy to confirm nodal metastasis before initiating treatment." This is further supported by the text in Section 5.2.3 (page 17), which notes that in patients with palpable nodes, "Lymph node metastasis should preferably be histopathologically confirmed by image-guided biopsy (e.g., US or CT)." |
| ESMO2024 | R | The guideline aligns with the recommendation by stating that for palpable inguinal nodes (clinically node-positive), metastasis "can be confirmed by carrying out percutaneous FNAC or a biopsy of the LN." The recommendations section and the diagnostic algorithm both specify that clinicians should perform a percutaneous FNAC or biopsy on palpable nodes to confirm the presence of cancer. This confirmatory step is advised before proceeding with definitive treatments like radical lymphadenectomy. |
| NCCN2025 | R | The NCCN guideline recommends a percutaneous (ultrasound- or CT-guided) biopsy as the initial step for patients with palpable inguinal lymph nodes, which corresponds to clinically node-positive (cN+) disease. The algorithms for "Management of Palpable Non-Bulky Inguinal Lymph Nodes" (PN-4) and "Management of Palpable Bulky Inguinal Lymph Nodes" (PN-5) both begin with a "Percutaneous lymph node biopsy." The discussion text further supports this, stating that percutaneous biopsy is the "favored approach" for palpable nodes to differentiate metastatic disease from inflammatory swelling before proceeding to definitive treatment (MS-11). |

**2. Primary Treatment (General Principles)**

2.1 Patients with early-stage, low-grade, and non-invasive tumors should be offered organ- and function-preserving therapy, depending on the tumor stage and differentiation grade.

| AUA2020 | R | The guideline strongly supports the recommendation for organ- and function-preserving therapy in early-stage, low-grade penile cancer. The document explicitly states there has been a "shift towards organ preserving techniques for patients with low volume disease of favorable histopathology (stages Tis, Ta, T1 and grades 1, 2)." The conclusion reinforces this, stating that "Organ preserving approaches are safe and feasible for early stage, low grade squamous cell carcinoma of the penis." Various organ-sparing options, including topical therapies, laser treatment, and specific surgical excisions, are presented as primary treatments for this patient population. |
| --- | --- | --- |
| DGU2020 | R | The guideline recommends organ- and function-preserving therapy for patients with penile cancer, with a specific focus on early-stage, non-invasive tumors. Recommendation 7.1 (page 43) states that patients should receive organ-preserving therapy depending on the stage and differentiation grade. More specifically, Recommendation 7.3 (page 45) states that for superficial, non-invasive penile carcinomas, an organ- and function-preserving curative treatment should be pursued. This directly aligns with the proposed treatment recommendation. |
| EAU-ASCO2025 | R | The guideline strongly supports the recommendation. Section 6.1.2 states, "When feasible, small and localised invasive lesions should receive organ-sparing treatment." Furthermore, the recommendations table on page 25 explicitly offers a "Strong" recommendation to "Offer organ-sparing surgery and reconstructive techniques to patients with lesions confined to the glans and prepuce (PeIN, Ta, T1–T2)". The guideline details multiple organ-preserving options, including topical therapies, laser ablation, surgery, and radiotherapy for non-invasive (PeIN, Ta) and early-stage (T1/T2) tumors, which aligns directly with the proposed recommendation. |
| ESMO2024 | R | The guideline strongly recommends the use of organ- and function-preserving therapies for early-stage penile cancer. It explicitly states, "The primary aim of surgical intervention is to remove the tumour using penile-preserving techniques. Preservation of the aesthetic, sexual and urinary function is an important outcome" (p. 3). The document then details multiple organ-sparing options for premalignant (PeIN), Ta-1, and T2 disease, including wide local excision, glans resurfacing, and glansectomy, which aligns directly with the treatment recommendation. |
| NCCN2025 | R | The NCCN guideline aligns with the recommendation by explicitly stating that organ-sparing approaches are suitable for early-stage penile cancer. For non-invasive tumors (PeIN, Ta) and low-grade invasive tumors (pT1a), the guideline recommends multiple penis-preserving techniques as primary treatment. These options include topical therapy, wide local excision, laser therapy, glansectomy, and Mohs surgery, all of which aim to preserve organ function. |

2.2 The goal of surgical treatment is complete excision with negative margins (R0). A margin of 5 mm for G1/G2 tumors and 10 mm for G3 tumors is suggested. In case of a positive surgical margin, a re-excision should be performed.

| AUA2020 | R | The guideline strongly supports the treatment recommendation. It explicitly states that "Most experts consider R0 surgical excision of the penile tumor to be the gold standard of treatment." The document directly aligns with the proposed margins by citing a histological analysis which found the maximum proximal extent of tumor spread was "5 mm for grade 1 and 2 tumors and 10 mm for grade 3 tumors." The guideline emphasizes the importance of negative margins, noting that "positive margins were associated with a higher rate of recurrence." |
| --- | --- | --- |
| DGU2020 | R | The guideline aligns with the treatment recommendation. Both state that the goal of surgical therapy is complete tumor removal with negative margins (p. 33), and both recommend a secondary operation (re-excision) if surgical margins are positive (Empfehlung 7.18, p. 54). While the guideline is less prescriptive about the exact margin width, suggesting "at least one to a few millimeters" (Empfehlung 7.17, p. 54), it acknowledges that the margin depends on the differentiation grade. The recommendation's specific 5 mm and 10 mm margins based on grade are a consistent application of the principles and evidence discussed in the guideline. |
| EAU-ASCO2025 | DR | While both the guideline and the recommendation agree on the fundamental goal of complete tumor removal with negative margins, they differ on the specific margin width. The recommendation suggests a strict rule of 5 mm for G1/G2 tumors and 10 mm for G3 tumors. In contrast, the guideline (Section 6.1.2.1) states there is "no clear evidence as to what constitutes an oncologically safe width of macroscopic negative surgical margins." The guideline suggests a more nuanced approach, noting that for lower-risk tumors, "macroscopic margins can indeed be minimal" as long as they are >1mm, while for higher-grade lesions, a "wider margin...may be prudent," rather than prescribing a specific distance. |
| ESMO2024 | DR | The guideline suggests a different approach to surgical margins compared to the treatment recommendation. While both agree on the importance of achieving clear margins, the guideline states that for T2 disease, "Surgical margins of >1 mm are now accepted, with the risk of local recurrence being low." This directly contrasts with the recommendation's proposal of a graded 5 mm margin for G1/G2 tumors and a 10 mm margin for G3 tumors. |
| NCCN2025 | R | The NCCN guideline strongly supports the core principles of the treatment recommendation. For various surgical approaches, including wide local excision and penectomy, the guideline repeatedly emphasizes the importance of achieving negative margins (PN-A, PN-B). It also explicitly states that if surgical margins are positive, "re-resection may be considered" (PN-A). While the guideline does not specify different margin sizes based on tumor grade, the discussion section (MS-7) notes that studies have shown surgical margins of 5 to 10 mm provide adequate tumor control, aligning with the range suggested in the recommendation. |

2.3 Offer patients a balanced and individualized discussion on the benefits and harms of possible treatment options. Inform them of the higher risk of local recurrence when using organ-sparing treatments compared to amputative surgery.

| AUA2020 | R | The guideline aligns with the recommendation by explicitly endorsing a shared decision-making model for treatment selection. It states, "The choice of therapy should involve a shared decision making process between the patient and the surgeon highlighting the risks and benefits of each approach." Furthermore, the guideline supports informing patients about the risk of recurrence with organ-sparing treatments, noting that the "high rate of local recurrence after laser therapy... remains a primary limitation of its use." |
| --- | --- | --- |
| DGU2020 | R | The guideline strongly recommends an individualized, evidence-based consultation for patients, covering all stage-appropriate therapy options, including their impact on tumor control, organ preservation, and quality of life (Recommendation 7.2, p. 43). It emphasizes that treatment decisions should be a compromise weighed between tumor control, organ preservation, and patient satisfaction, requiring thorough patient information (p. 43). Specifically aligning with the recommendation, the guideline states that a slightly higher rate of local recurrence is accepted with organ-sparing treatments compared to amputation, as these recurrences can often be treated effectively later (p. 43). |
| EAU-ASCO2025 | R | The guideline strongly recommends the proposed treatment approach. In the recommendations table in section 6.1.5, the guideline explicitly states to "Offer a balanced and individualised discussion on benefits and harms of possible treatment options with the goal of shared decision making." It also specifically advises to "Inform patients of the higher risk of local recurrence when using organ-sparing treatments compared to amputative surgery." Both statements are rated as "Strong" recommendations, directly aligning with the provided text. |
| ESMO2024 | R | The guideline supports an individualized approach to treatment, stating that surgical options should be "tailored according to the disease stage, patient willingness for reconstruction and clear surgical margins." This aligns with offering patients a balanced discussion of their options. The guideline also confirms the higher risk of local recurrence with organ-sparing treatments, noting that as "penile-preserving techniques have now become standard of care... recurrence rates are 20%-50%," necessitating close clinical follow-up. |
| NCCN2025 | R | The NCCN guideline strongly supports the recommendation for a balanced, individualized discussion with patients. The guideline explicitly states on page MS-8 that "A clear and frank discussion should be had with the patient" regarding treatment options and their risks. Furthermore, it directly corroborates the information about recurrence, noting on page MS-5 that a systematic review found "a higher risk of local recurrence with penile-sparing approaches" compared to amputative surgery. The Surveillance section on page MS-16 reinforces this, stating that "penile-sparing therapies carry a significantly higher risk of local recurrence (28%) than partial or total penectomy (5%)." |

**3. Primary Treatment (Topical Therapy)**

3.1 Topical therapy with 5-fluorouracil (5-FU) or imiquimod can be offered to patients with biopsy-confirmed superficial disease (PeIN/Tis, Ta). Patients should be informed about limited efficacy and potential off-label use.

| AUA2020 | R | The AUA guideline recommends topical therapy for early-stage penile cancer, aligning with the treatment recommendation. The guideline states, "Topical treatments... are often used as first line treatment options for non-invasive and early stage penile malignancies (carcinoma in situ, Ta and T1)." It explicitly names fluorouracil (5-FU) and imiquimod as the most common topical agents for these indications. Furthermore, the treatment algorithm in Figure 4 lists "Topical treatment" as a primary option for early/low-stage disease. |
| --- | --- | --- |
| DGU2020 | R | The guideline aligns with the treatment recommendation. Recommendation 7.6 on page 47 explicitly states that topical therapy with 5-fluorouracil or imiquimod can be used for the treatment of penile intraepithelial neoplasia (PeIN) or precancerous lesions. Furthermore, recommendation 7.7 mirrors the proposed recommendation's caveat, emphasizing that patients should be informed about the limited efficacy and the "off-label" use of these therapies. |
| EAU-ASCO2025 | R | The guideline supports the use of topical therapy for superficial, non-invasive penile cancer. Section 6.1.1.1 describes topical therapy with imiquimod (IQ) or 5-fluorouracil (5-FU) as "effective non-invasive first-line treatment options" for PeIN and Ta disease. Furthermore, the recommendations table on page 24 explicitly states to "Offer topical therapy with 5-fluorouracil or imiquimod to patients with biopsy-confirmed penile intra-epithelial neoplasia (PeIN)," directly aligning with the proposed treatment. |
| ESMO2024 | R | The guideline supports the recommendation, stating that for biopsy-proven premalignant disease (PeIN), "any residual PeIN can be treated using topical agents, such as 5-fluorouracil (5-FU) or imiquimod" after an initial circumcision. The management algorithm for Tis or Ta disease also includes "Topical ChT or immunotherapy" as a valid treatment option. This aligns directly with offering topical 5-FU or imiquimod for superficial penile cancer. |
| NCCN2025 | R | The NCCN guideline aligns with the treatment recommendation. The primary treatment algorithm (PN-1) lists "Topical therapy" as a first-line option for patients with Penile intraepithelial neoplasia (PeIN) or Ta disease. Furthermore, the "Principles of Penile Organ-Sparing Approaches" section (PN-A) and the discussion section (MS-8) specify that for patients with PeIN or Ta disease, treatment can include "Imiquimod 5%" or "5-FU cream 5%," directly matching the drugs and indications in the recommendation. |

3.2 If topical treatment fails, it should not be repeated.

| AUA2020 | DR | The guideline presents a different approach than the recommendation. While the recommendation states that topical treatment should not be repeated if it fails, the guideline discusses a study by Alnajjar et al. where the first-line therapy was 5-fluorouracil (5-FU) and the "second line topical agent was IQ" (imiquimod). This indicates that using a different topical agent after the failure of the first is a viable strategy, which directly contradicts the recommendation's absolute stance against repeating topical treatment. |
| --- | --- | --- |
| DGU2020 | R | The guideline aligns with the treatment recommendation. On page 48, in the background information for topical chemotherapy, the guideline states, "Bei Tumorpersistenz oder Rezidiv nach topischer Anwendung ist eine Wiederholung mit diesen Verfahren nicht angeraten." This translates to, "In case of tumor persistence or recurrence after topical application, a repetition with these methods is not advisable." This directly supports the recommendation that failed topical treatment should not be repeated. |
| EAU-ASCO2025 | R | The guideline directly supports the recommendation. In Section 6.1.1.1, "Topical therapies," the guideline discusses the use of agents like imiquimod and 5-fluorouracil. It explicitly states, "Insufficient responses and recurrences may signify underlying invasive disease; hence, if topical treatment fails, it should not be repeated." This statement is also formalized in a recommendations table on page 25, confirming the guideline's alignment with the provided treatment recommendation. |
| ESMO2024 | R | The guideline supports the recommendation. For premalignant disease (PeIN), it states that after initial treatment with topical agents, the response should be assessed. The guideline then explicitly states, "If topical treatment fails, wide local excision or glans resurfacing... should be considered." This aligns with the recommendation, as it advises escalating to a different, surgical treatment modality rather than repeating the failed topical therapy. |
| NCCN2025 | DR | The recommendation states that if topical treatment fails, it should not be repeated. The NCCN guidelines suggest a different approach. The algorithm for managing recurrent disease (PN-9) directs clinicians to treat according to the recurrence stage, which refers back to the initial treatment options on PN-1. For a recurrent PeIN or Ta lesion, topical therapy is again listed as a valid option. The discussion (MS-5) also mentions a study where imiquimod was used as a "second-line topical agent" after 5-fluorouracil, indicating that repeating treatment with a different topical agent is a considered strategy. |

3.3 Example regimens for topical therapy include: • Imiquimod 5% cream: Apply at night three times per week for 4–16 weeks. • 5-FU cream 5%: Apply twice daily for 2–6 weeks.

| AUA2020 | R | The guideline strongly supports the use of topical therapy for early-stage penile cancer. It explicitly identifies 5-fluorouracil (5-FU) and imiquimod as the "most common chemotherapeutic compounds" for this purpose. The document discusses the efficacy of both agents for carcinoma in situ and mentions the use of 5% imiquimod, aligning directly with the agents and concentrations in the recommendation. |
| --- | --- | --- |
| DGU2020 | R | The guideline explicitly supports the use of topical therapy with 5-Fluorouracil or Imiquimod for penile intraepithelial neoplasia or precancerous lesions (Recommendation 7.6). The background text on pages 47-48 discusses various application regimens from the literature for both 5% Imiquimod cream and 5-FU. Although the guideline notes that no definitive standards for application exist, the example regimens in the treatment recommendation are consistent with the schedules described in the cited literature. |
| EAU-ASCO2025 | R | The guideline recommends topical therapy with imiquimod or 5-fluorouracil (5-FU) as effective first-line treatment options for superficial non-invasive disease (PeIN). Section 6.1.1.1, "Topical therapies," specifically discusses both agents. The guideline's description of a common imiquimod regimen ("three times per week for twelve weeks") aligns with the recommended range of 4–16 weeks. While it describes a different 5-FU schedule than the one recommended, it explicitly states that "no standard protocol exists" and "There is no consensus or comparative data on the optimal treatment schedules for these therapies," indicating that various regimens are in use. |
| ESMO2024 | R | The guideline recommends the use of topical agents for treating residual premalignant disease (PeIN) following circumcision. It specifically names the two agents from the treatment recommendation, stating that "any residual PeIN can be treated using topical agents, such as 5-fluorouracil (5-FU) or imiquimod." This directly aligns with and supports the proposed treatment approach. |
| NCCN2025 | R | The guideline recommends the exact topical therapy regimens mentioned in the treatment recommendation. On page PN-A, under "Principles of Penile Organ-Sparing Approaches," the guideline states, "For patients with PeIN or Ta disease: ► Imiquimod 5%: apply at night three times per week for 4–16 weeks. ► 5-FU cream 5%: apply twice daily for 2–6 weeks." This aligns perfectly with the proposed treatment, confirming its recommended status. |

3.4 Topical therapy is ineffective for more advanced stages of penile cancer.

| AUA2020 | R | The guideline directly supports the recommendation. It explicitly states that "topical treatment is ineffective for more advanced stages of penile cancer" and should be reserved for early-stage disease. The text further reinforces this by identifying topical treatments as first-line options for "non-invasive and early stage penile malignancies (carcinoma in situ, Ta and T1)." |
| --- | --- | --- |
| DGU2020 | R | The guideline supports the recommendation by limiting the use of topical therapy to early-stage disease. Section 7.1.1.1 specifies that topical chemotherapy can be applied for superficial, non-invasive tumors and precancerous lesions (PeIN, CIS). In contrast, for advanced stages such as T3 (Section 7.1.4) and T4 (Section 7.1.5), the guideline recommends more aggressive treatments like partial or radical penectomy and radiotherapy, omitting topical therapy as a viable option. |
| EAU-ASCO2025 | R | The guideline supports the recommendation by exclusively positioning topical therapies, such as imiquimod (IQ) or 5-fluorouracil (5-FU), as treatment options for "superficial non-invasive disease (PeIN, Ta)" (page 19, section 6.1.1.1). For more advanced stages, including invasive disease confined to the glans (cT1/T2) and locally advanced disease (T3–T4), the guideline recommends surgical, radiotherapeutic, and chemotherapeutic approaches, making no mention of topical agents (pages 20, 23). This omission for advanced stages implicitly confirms that topical therapy is not considered an effective treatment in those settings. |
| ESMO2024 | R | The guideline aligns with the recommendation by limiting the use of topical therapy to the earliest stages of penile cancer. It explicitly recommends topical agents such as 5-fluorouracil (5-FU) and imiquimod for premalignant disease (PeIN) and Tis/Ta lesions. For more advanced stages (T1b-T4 and node-positive disease), the guideline recommends surgery, radiotherapy, and systemic chemotherapy, implicitly agreeing that topical therapy is not an effective option for advanced disease. |
| NCCN2025 | R | The guideline recommends topical therapy as a primary treatment option for early, non-invasive stages of penile cancer, specifically Penile Intraepithelial Neoplasia (PeIN) and Ta disease (PN-1). For more advanced stages (T1, T2, and greater), the guideline does not list topical therapy as a viable treatment, instead recommending more invasive approaches such as wide local excision, penectomy, or radiation therapy (PN-2). This approach directly supports the recommendation that topical therapy is not effective for more advanced stages of penile cancer. |

**4. Primary Treatment (Laser Therapy)**

4.1 Laser ablation (e.g., CO₂ or Nd:YAG laser) can be used for biopsy-confirmed superficial or early-stage tumors (PeIN/Tis, Ta, T1).

| AUA2020 | R | The guideline recommends laser ablation for early-stage penile cancer, aligning with the treatment recommendation. On page 74, under "TOPICAL THERAPIES," the guideline states that "laser and photodynamic therapy are often used as first line treatment options for non-invasive and early stage penile malignancies (carcinoma in situ, Ta and T1)." The "LASER THERAPY" section on page 75 specifically discusses the use of CO₂ and YAG lasers for these conditions, which are the same laser types mentioned in the recommendation. |
| --- | --- | --- |
| DGU2020 | R | The guideline recommends laser therapy for early-stage penile cancer, aligning with the treatment recommendation. Specifically, Recommendation 7.8 in section 7.1.1.2 states that laser systems can be used for the ablation of superficial penile carcinomas. It further specifies that the CO₂ laser is suitable for penile intraepithelial neoplasia (PeIN) and precancerous lesions, while the Nd:YAG laser can be used for Ta/T1 tumors, which directly corresponds to the methods and indications mentioned in the treatment recommendation. |
| EAU-ASCO2025 | R | The guideline supports the use of laser ablation for superficial and early-stage penile cancer. On page 25, in the recommendations table under "Laser ablation," the guideline explicitly states: "Offer laser ablation using CO₂ or Nd:YAG laser to patients with biopsy-confirmed PeIN, Ta or T1 lesions." This aligns directly with the treatment, laser types, and tumor stages mentioned in the recommendation. While the guideline assigns a "Weak" strength rating to this recommendation and discusses high recurrence rates (page 21), it is still presented as a valid treatment option to be offered to patients. |
| ESMO2024 | R | The guideline recommends the use of laser ablation for treating premalignant and early-stage penile cancer, aligning with the proposed treatment. Specifically, for premalignant disease (PeIN), the guideline states that “carbon dioxide (CO₂) laser ablation…can be used.” Furthermore, the guideline’s treatment algorithm (Figure 2) explicitly lists “Laser therapy” as a management option for tumors staged as Tis, Ta, and T1. |
| NCCN2025 | R | The NCCN guideline supports the use of laser therapy for early-stage penile cancer. In the treatment algorithm for "Penile intraepithelial neoplasia (PeIN) or Ta" (PN-1) and for "pT1a" tumors (PN-2), "Laser therapy (category 2B)" is listed as a primary treatment option. Furthermore, the "Principles of Penile Organ-Sparing Approaches" section (PN-A) explicitly states that therapeutic lasers, including CO₂ and Nd:YAG, can be used to treat selected cases of PeIN, Ta, and T1 tumors. |

4.2 A surgical margin of 5 mm is considered oncologically safe for laser therapy.

| AUA2020 | R | The guideline directly supports the recommendation for a 5 mm margin with laser therapy. The "LASER THERAPY" section states, "According to current guidelines a margin of 5 mm is considered oncologically safe." The appendix on laser modalities further details the CO2 laser technique, specifying an "Incision depth shall be... 5 mm from visible tumor border." |
| --- | --- | --- |
| DGU2020 | R | The guideline recommends a surgical margin of "at least one to a few millimeters" for organ-preserving operative therapies (Recommendation 7.17). The background text further elaborates that a margin of a few millimeters is considered oncologically safe, moving away from older recommendations of 2 cm. Crucially, it cites a systematic review by Kamel et al. [174] which concluded that a 5 mm resection margin is sufficient for organ-preserving surgery, directly aligning with the proposed recommendation. |
| EAU-ASCO2025 | DR | The guideline does not explicitly state that a 5 mm surgical margin is oncologically safe for laser therapy. In Section 6.1.2.3, it vaguely mentions resecting the tumor with "ample millimetres of margin" but offers no specific measurement. Furthermore, this section cautions that laser therapy may result in higher local recurrence rates when compared to surgical excision. While the guideline does mention that a 5-10 mm margin for surgical excision results in low recurrence rates (Section 6.1.2.1), it does not apply this standard to laser therapy and emphasizes the overall lack of clear evidence for what constitutes an oncologically safe margin. |
| ESMO2024 | NA | laser ablation as a treatment option for premalignant disease (PeIN) and Tis/Ta tumors. However, it does not specify what constitutes a safe surgical margin for this procedure. While surgical margins are discussed for other techniques like glansectomy, there is no mention of a 5 mm margin or any other specific margin size in the context of laser therapy. |
| NCCN2025 | NA | The NCCN guideline discusses laser therapy as an organ-sparing approach for early-stage penile cancer and mentions the importance of achieving "adequate lateral margins" to prevent recurrence. However, it does not specify a particular margin size, such as 5 mm, that is considered oncologically safe for laser therapy. While the guideline does provide margin size recommendations (5 to 10 mm) for wide local excision, it does not address a specific numerical margin for laser therapy itself. |

**5. Primary Treatment (Post-Therapy Biopsy)**

5.1 After topical or laser therapy, a control biopsy should be performed to verify local tumor control, especially in cases of clinical doubt.

| AUA2020 | NA | The guideline extensively discusses the need for close monitoring and follow-up after topical and laser therapies due to the high risk of local recurrence. For instance, it states that after laser therapy, "close follow-up especially during the first 6 months is recommended." However, the document does not specify the methods for this surveillance and never mentions or discusses the use of a "control biopsy" to verify tumor control after these treatments. |
| --- | --- | --- |
| DGU2020 | R | The guideline directly aligns with the treatment recommendation. Recommendation 7.9 on page 49 explicitly states, "Nach topischer medikamentöser Therapie oder Lasertherapie soll postinterventionell eine Kontrollbiopsie zur Verifikation der lokalen Tumorkontrolle und eine regelmäßige langfristige Nachsorge erfolgen" (After topical medicinal therapy or laser therapy, a control biopsy should be performed to verify local tumor control and regular long-term follow-up should be carried out). This recommendation, which has 100% consensus, mirrors the proposed treatment approach for the same purpose of verifying local tumor control. The background information on page 46 further supports this, noting that therapies preserving the epithelium require a control biopsy to confirm tumor control. |
| EAU-ASCO2025 | R | The guideline aligns with the recommendation. In the section on topical therapies for superficial non-invasive disease (PeIN), it states, "It is advised that treatment effects must be clinically assessed and in cases of doubt evaluated by biopsy" (page 19). This is reinforced in the recommendations table on page 25. Furthermore, the follow-up regimen table suggests an optional "Repeat biopsy after topical or laser treatment for PeIN" (page 38), which supports the recommendation's call for a control biopsy, especially in cases of clinical doubt. |
| ESMO2024 | R | The guideline supports the recommendation. In the section on "Premalignant disease (PeIN)," it states that "Following topical treatment, the response should be assessed clinically or with a repeat biopsy of any new lesions which may indicate progression to invasive disease." This aligns with the recommendation to perform a control biopsy to verify tumor control, particularly when there is clinical uncertainty. |
| NCCN2025 | DR | The NCCN guideline outlines a specific surveillance schedule after topical or local therapy which consists of regular clinical examinations of the penis and inguinal region (PN-8). While it suggests imaging for abnormal clinical findings, it does not mention or recommend performing a control biopsy to verify local tumor control. The guideline's specified follow-up protocol (clinical examination) differs from the recommendation's proposed method (control biopsy). |

**6. Primary Treatment (Surgical)**

6.1 Organ-sparing surgery options (circumcision, wide local excision, glansectomy, Mohs) are offered for lesions confined to the glans and prepuce (PeIN, Ta, T1-T2) in patients willing to comply with strict follow-up.

| AUA2020 | R | The guideline strongly recommends organ-preserving surgical techniques for early-stage penile cancer, which aligns with the treatment recommendation. The "SURGERY" section states there has been "a shift towards organ preserving techniques for patients with low volume disease of favorable histopathology (stages Tis, Ta, T1 and grades 1, 2)." It explicitly lists the same four common approaches: circumcision, tumor-only excision, Mohs surgery, and glansectomy. The guideline also reinforces the need for strict patient monitoring, stating, "Close follow-up for at least 5 years is required to detect and re-treat any potential recurrences." |
| --- | --- | --- |
| DGU2020 | R | The guideline strongly supports an organ-preserving approach for early-stage penile cancer, which aligns with the treatment recommendation. Specifically, it recommends organ- and function-preserving treatment for superficial, non-invasive tumors (PeIN, Ta) in Section 7.1.1. For tumors confined to the foreskin, circumcision is recommended (Section 7.1.2, Recommendation 7.11). Furthermore, for invasive tumors limited to the glans (T1/T2), the guideline explicitly suggests local excision and glansectomy as treatment options (Section 7.1.3). |
| EAU-ASCO2025 | R | The guideline strongly supports the recommendation to use organ-sparing surgery for early-stage penile cancer. In its recommendations table on page 25, it gives a "Strong" rating to offering "organ-sparing surgery and reconstructive techniques to patients with lesions confined to the glans and prepuce (PeIN, Ta, T1–T2) and who are willing to comply with strict follow-up." The guideline specifically discusses circumcision, wide local excision, and glansectomy as options for these stages. While it notes that Mohs surgery is "not routinely recommended" due to very limited data (p. 21), the overall principle and the other listed surgical options align directly with the treatment recommendation. |
| ESMO2024 | R | The guideline aligns with the recommendation by advocating for organ-sparing and penile-preserving surgery for localized disease. It specifically recommends circumcision for premalignant disease (PeIN), wide local excision for Ta-T1 disease, and glansectomy for T2 tumors on the glans penis. Additionally, it mentions Mohs micrographic surgery as a potential option for low-grade T1 lesions, covering all procedures and stages mentioned in the recommendation. |
| NCCN2025 | DR | The NCCN guideline recommends organ-sparing surgery options, including wide local excision, glansectomy, and Mohs surgery, for early-stage penile cancer, specifically PeIN, Ta, and T1a disease (PN-A, PN-2). However, the guideline differs from the recommendation for T2 disease. For T2 or greater lesions, the guideline's primary treatment pathway indicates more extensive surgery, such as partial or total penectomy, is typically required (PN-2, MS-8). Since the recommendation includes T2 lesions as being eligible for these organ-sparing approaches, it proposes a less aggressive treatment for this stage than the guideline advises. |

6.2 Intra-operative frozen section analysis of resection margins should be performed to confirm negative margins, especially in cases of doubt.

| AUA2020 | R | The guideline supports the use of intraoperative frozen section analysis to ensure negative margins during surgical excision. It explicitly states that "In experienced hands tumor excision with negative intraoperative frozen section margins can be performed safely in well selected patients." The document also compares Mohs surgery to negative intraoperative frozen section margins, treating it as a standard technique for achieving oncologic control. |
| --- | --- | --- |
| DGU2020 | R | The guideline supports the use of intraoperative frozen section analysis to ensure negative resection margins. Section 4.4.6 explicitly states that the goal of surgery is complete tumor removal with negative margins and that these margins "can be secured intraoperatively by pathohistological frozen section analysis [101]." This directly aligns with the treatment recommendation to perform intra-operative frozen section analysis to confirm negative margins. |
| EAU-ASCO2025 | R | The guideline recommends performing intra-operative frozen section analysis in cases of doubt, which aligns with the treatment recommendation. Specifically, in the recommendations table on page 25, the guideline states to "Perform intra-operative frozen section analysis of resection margins in cases of doubt on the completeness of resection." Section 6.1.2.2 further clarifies that while routine use is not recommended due to conflicting evidence, the Panel's opinion is that it is a "helpful tool to achieve definitive tumour-free margins." |
| ESMO2024 | NA | The guideline recommends the use of intra-operative frozen section analysis, but only in the context of inguinal lymph node management. Specifically, it mentions using frozen section analysis during a superficial modified inguinal lymphadenectomy to decide whether to proceed to a radical dissection. The recommendation, however, suggests using frozen sections to confirm negative resection margins of the primary tumor, a topic which is not addressed in the guideline. |
| NCCN2025 | R | The NCCN guideline explicitly supports the use of intra-operative frozen sections to ensure negative surgical margins. Under the "Principles of Surgery" for penectomy, the guideline states, "Intraoperative frozen sections are recommended to determine negative margins" (PN-B). Similarly, for glansectomy, it specifies that "Negative surgical margins should be determined from frozen sections of the cavernosal bed and urethral stump" (PN-A, 2 of 2), directly aligning with the proposed recommendation. |

6.3 Circumcision can be a curative treatment for tumors confined to the prepuce (PeIN/Tis, Ta).

| AUA2020 | R | The guideline explicitly supports the recommendation. In the "SURGERY" section, it states that "Foreskin circumcision... can be curative for low grade and low stage disease." The treatment algorithm in Figure 4 also lists circumcision as a primary treatment option for de novo, uncircumcised lesions located on the foreskin (distal prepuce). This directly aligns with the recommendation for using circumcision as a curative treatment for tumors confined to the prepuce. |
| --- | --- | --- |
| DGU2020 | R | The guideline directly supports the recommendation. Section 7.1.2, Recommendation 7.11, explicitly states that tumors confined to the foreskin should be treated by circumcision. The background information for this recommendation further confirms that circumcision is considered a curative ("kurativ") treatment for these tumors, provided tumor-free margins are achieved. |
| EAU-ASCO2025 | R | The guideline recommends circumcision as a primary treatment option for superficial non-invasive disease (PeIN, Ta) located on the prepuce. Section 6.1.1 states, "Most PeIN lesions are located on the mucosal surfaces of the glans or prepuce... Thus, circumcision should be the primary surgical option." Section 6.1.2.5 further supports this, noting that for small, distal preputial penile cancer, "circumcision alone usually presents adequate treatment." This aligns directly with the provided treatment recommendation. |
| ESMO2024 | R | The guideline supports the recommendation. Under the "Ta-1 disease" section, it explicitly states, "Patients with tumour localised to the foreskin can undergo a circumcision, which is often therapeutic." Additionally, for premalignant disease (PeIN) located on the prepuce, the guideline notes that "circumcision is mandatory as the initial management." This directly aligns with the recommendation that circumcision can be a curative treatment for these early-stage tumors confined to the prepuce. |
| NCCN2025 | R | The guideline supports the treatment recommendation. In the "Principles of Penile Organ-Sparing Approaches" section (PN-A, 2 of 2), under "Wide Local Excision," the guideline explicitly states, "Circumcision alone may be reasonable for tumors of the distal prepuce." This aligns directly with the recommendation for using circumcision as a treatment for early-stage tumors (PeIN, Ta) confined to the prepuce. The NCCN Recommendations section (MS-8) further lists "circumcision and wide local excision" among the penis-preserving techniques for PeIN or Ta disease. |

6.4 When using radiotherapy or topical therapy for a preputial lesion, a circumcision should be performed first.

| AUA2020 | NA | The guideline identifies foreskin circumcision as a primary and often curative treatment for preputial disease in uncircumcised men. The treatment algorithm for a lesion on the foreskin lists circumcision and topical therapy as alternative options. However, the guideline does not explicitly address the recommendation to perform a circumcision as a mandatory preparatory step before initiating topical or radiation therapy for a preputial lesion. |
| --- | --- | --- |
| DGU2020 | R | The guideline directly supports the treatment recommendation. In section 7.1.3, recommendation 7.13 explicitly states, "Bei Behandlung mittels Strahlentherapie oder topischer Therapie sollte eine Zirkumzision durchgeführt werden" (When treating with radiotherapy or topical therapy, a circumcision should be performed). The background information on page 52 further clarifies that the non-diseased foreskin should be removed as it facilitates the healing process. This aligns perfectly with the proposed recommendation. |
| EAU-ASCO2025 | R | The guideline aligns with the treatment recommendation. It states that for superficial non-invasive disease (PeIN) on the prepuce, "circumcision should be the primary surgical option" (p. 19). Furthermore, it explicitly notes that "circumcision combined with topical treatment, laser therapy or brachytherapy, facilitates follow-up examinations" (p. 22), reinforcing the role of circumcision in this context. A systematic review cited by the panel also found that patients undergoing brachytherapy had it performed "after circumcision" (p. 23). |
| ESMO2024 | R | The guideline aligns with the recommendation. For premalignant disease (PeIN) on the prepuce, the guideline states that "circumcision is mandatory as the initial management" before subsequent treatment with topical agents. Similarly, when discussing radiotherapy, it specifies that "In all cases, a circumcision should be carried out before brachytherapy." |
| NCCN2025 | DR | The guideline recommends that circumcision should be performed before radiotherapy to prevent radiation-related complications. However, the guideline does not state that circumcision is a prerequisite for topical therapy. By specifying this requirement for only one of the two modalities, the guideline presents a different, more nuanced recommendation than the one provided, which applies the prerequisite to both treatments. |

6.5 Wide local excision (WLE) is an option for early-stage tumors (PeIN, Ta, T1). Complete excision with wide negative margins is required.

| AUA2020 | R | The guideline strongly supports this recommendation, stating that "R0 surgical excision of the penile tumor" is considered the "gold standard of treatment" for early-stage disease (Tis, Ta, T1). The document explicitly lists "tumor-only excision" as one of the four commonly used surgical approaches for these patients. The guideline also reinforces the importance of achieving negative margins to ensure complete removal of the tumor. |
| --- | --- | --- |
| DGU2020 | R | The guideline recommends local excision ("Lokalexzision") as a treatment option for early-stage penile cancer. Specifically, recommendation 7.14 on page 52 states that for smaller, invasive tumors of the glans (T1/T2), a local excision should be performed. The accompanying background text emphasizes that this is a reasonable organ-preserving approach and that negative surgical margins ("Negative Schnittränder") are required, which directly aligns with the proposed treatment recommendation. |
| EAU-ASCO2025 | R | The guideline explicitly supports the use of wide local excision (WLE) for early-stage penile cancer. Section 6.1.2 identifies WLE as a surgical option for invasive disease confined to the glans (cT1/T2). Furthermore, the recommendations table on page 25 gives a "Strong" rating for offering organ-sparing surgery, including wide local excision, for patients with PeIN, Ta, and T1–T2 lesions. The guideline also aligns with the need for complete removal, stating in section 6.1.2.5 that WLE should include a "margin of clinically normal-appearing skin around the tumour." |
| ESMO2024 | R | The guideline supports the use of wide local excision (WLE) for early-stage penile cancer, aligning with the recommendation. For premalignant disease (PeIN), the guideline suggests WLE if topical treatments fail (p. 4). For Ta-1 disease, it is explicitly listed as a recommended treatment option (p. 9). The management algorithm in Figure 2 further illustrates WLE as a viable surgical approach for Tis, Ta, and T1 tumors (p. 4). |
| NCCN2025 | R | The NCCN guidelines recommend wide local excision as a primary treatment option for patients with Penile Intraepithelial Neoplasia (PeIN) or Ta disease (PN-1). The guidelines also list wide local excision as a treatment for pathologic stage pT1a and pT1b tumors (PN-2). Furthermore, the "Principles of Penile Organ-Sparing Approaches" section explicitly states this procedure is designated for early-stage penile cancer (PeIN, Ta, T1) and requires complete excision with a wide negative margin (PN-A). |

6.6 Mohs micrographic surgery can be considered as an alternative to WLE for select, low-grade, superficial lesions, particularly on the glans or distal penis, if the appropriate expertise is available.

| AUA2020 | R | The guideline supports the recommendation by listing Mohs surgery as one of the four commonly used surgical approaches for early-stage penile cancer. It explicitly states that "Mohs surgery can be considered for disease of favorable histopathology and when the affected area is within the distal penis or glans penis," which directly corresponds to the recommendation for its use in select, low-grade, superficial lesions in those specific locations. The treatment algorithm (Figure 4) further includes Mohs surgery as an option for de novo and recurrent lesions. |
| --- | --- | --- |
| DGU2020 | NA | The guideline provides a comprehensive overview of surgical treatments for primary penile cancer, including local excision, laser therapy, glansectomy, and partial amputation (p. 51). However, it does not mention or discuss Mohs micrographic surgery as a specific therapeutic option. Although "Mohs-Chirurgie" is mentioned once in the document, it is only in the context of summarizing the scope of a cited systematic review (p. 55), not as part of a formal recommendation or discussion. Therefore, the guideline does not address the recommendation to use Mohs surgery for select lesions. |
| EAU-ASCO2025 | DR | The guideline explicitly states that Mohs micrographic surgery is not routinely recommended due to very limited data. This differs from the treatment recommendation, which suggests it "can be considered as an alternative" for select lesions. While both mention the importance of appropriate expertise, the guideline's explicit statement against routine use (Section 6.1.2.4) represents a more cautious and disapproving stance compared to the recommendation's conditional consideration. |
| ESMO2024 | R | The guideline supports the use of Mohs micrographic surgery for low-grade penile lesions (T1), which aligns with the recommendation for select, low-grade, superficial lesions. The guideline echoes the recommendation's condition regarding expertise, stating it should only be performed if "the right clinical set-up, including a pathologist, is available." While the guideline notes a high recurrence rate (32%), it is still presented as a valid therapeutic option for Ta-1 disease. |
| NCCN2025 | R | The NCCN guideline aligns with the recommendation by listing Mohs surgery as a primary treatment option for early-stage penile cancer (PeIN, Ta, and pT1a) in its treatment algorithms (PN-1, PN-2). Under the "Principles of Penile Organ-Sparing Approaches" (PN-A, 2 of 2), it explicitly states that "Mohs surgery is an alternative to wide local excision in select cases." The guideline further specifies that it may be preferable for "a small superficial lesion," which is consistent with the recommendation's conditions. |

6.7 Glansectomy (partial or total) or glans resurfacing are options for tumors located on the glans penis (PeIN, Ta, T1-T2). Reconstruction may be performed with a split-thickness skin graft.

| AUA2020 | R | The guideline supports the recommendation, explicitly listing glansectomy and glans resurfacing as primary surgical options for early-stage penile cancer located on the glans. The "SURGERY" section identifies glansectomy as one of the four common surgical approaches, and the treatment algorithm (Figure 4) clearly indicates that glansectomy and glans resurfacing are appropriate for glans lesions. Furthermore, the guideline includes figures and discussion related to reconstruction with partial thickness skin grafting after these procedures. |
| --- | --- | --- |
| DGU2020 | R | The guideline supports the recommendation by presenting glans resurfacing ("glans resurfacing") as a therapeutic option for carcinoma in situ (CIS/PeIN) (Recommendation 7.10) and strongly recommending glansectomy ("Glansektomie") for T1/T2 tumors (Recommendation 7.15). The guideline further aligns with the recommendation by describing the use of skin grafts, including split-thickness grafts, for reconstruction following these procedures (p. 50, 53). Both treatments are listed as organ-preserving options for tumors located on the glans. |
| EAU-ASCO2025 | R | The guideline supports the recommendation by identifying glansectomy and glans resurfacing as primary surgical options for tumors confined to the glans. Section 6.1.2.7 states that patients with tumors confined to the glans (T1-T2) are "good candidates for glansectomy," and Section 6.1.2.6 discusses glans resurfacing for T1/T2 lesions. Furthermore, the guideline strongly recommends offering organ-sparing surgery, explicitly including glansectomy and glans resurfacing, for lesions confined to the glans and prepuce staged as PeIN, Ta, and T1-T2 (page 25). The guideline also corroborates the use of a split-thickness skin graft for reconstruction after these procedures. |
| ESMO2024 | R | The guideline supports the use of glansectomy and glans resurfacing for tumors on the glans penis, aligning with the recommendation. For premalignant disease (PeIN) and Ta-1 lesions, the guideline recommends glans resurfacing with a split-thickness skin graft (SSG). For T2 disease, it states that glansectomy with SSG reconstruction is the surgical treatment of choice. These recommendations are also outlined in the provided treatment algorithm (Figure 2). |
| NCCN2025 | R | The NCCN guideline recommends glansectomy as a treatment option for distal tumors, specifically for clinical stages PeIN, Ta, and T1, which aligns with the recommendation. The guideline further states that for PeIN or Ta disease, complete glansectomy is a category 2B recommendation, and for pT1a disease, glansectomy is an option in select cases. Additionally, the guideline mentions that glans resurfacing can be considered in select patients and that reconstruction with a split-thickness skin graft (STSG) may follow the procedure. |

6.8 Partial or total penectomy is recommended for patients with higher stage tumors (T2, T3, T4), particularly with invasion of the corpora cavernosa, unfavorable histopathology, or for those not suitable for organ-sparing approaches.

| AUA2020 | R | The guideline supports the recommendation, stating that for "higher stage/grade tumors this would mean partial or total penectomy." It further specifies that more aggressive surgical approaches like partial and total penectomy are "acceptable options for patients with unfavorable histopathology or recurrent disease." This aligns perfectly with the recommendation to use these procedures for higher-stage tumors (T2-T4) and unfavorable histopathology. |
| --- | --- | --- |
| DGU2020 | R | The guideline supports the recommendation for partial or total penectomy in higher-stage tumors. For T3 tumors, defined by invasion of the corpora cavernosa, Recommendation 7.22 (p. 59) advises a partial penectomy (`Penisteilamputation`), with radical penectomy as an option for extensive disease. For advanced T4 tumors, Recommendation 7.24 (p. 60) suggests extensive operative resection, which includes radical penectomy. The guideline also recommends partial penectomy for T1/T2 tumors when more conservative options are not feasible (Recommendation 7.16, p. 53), aligning with the proposed approach. |
| EAU-ASCO2025 | R | The guideline aligns with the recommendation to use partial or total penectomy for higher-stage tumors. Specifically, section 6.1.3.1 states that for cT3 patients with "obvious involvement of the corpora cavernosa, partial amputation is standard," and that total penectomy is the standard treatment for more extensive cases. Furthermore, the recommendations table on page 25 gives a "Strong" rating to offering partial penectomy for patients with invasion of the corpora cavernosa (T3) and total penectomy for large invasive tumors not amenable to partial amputation (e.g., T4). |
| ESMO2024 | R | The guideline aligns with the treatment recommendation. For T3 and T4 disease, it explicitly states that partial or total penectomy is the recommended treatment, particularly when the cancer infiltrates the corpus cavernosum. While the guideline notes that glansectomy is the treatment of choice for T2 tumors on the glans, it also qualifies that partial or total penectomy remain "valid alternatives whenever adequate surgical margins cannot be guaranteed or when the patient is unfit for reconstruction," which is consistent with the recommendation's condition for patients not suitable for organ-sparing approaches. |
| NCCN2025 | R | The NCCN guideline aligns with the treatment recommendation. In the treatment algorithm on page PN-2, both "Partial penectomy" and "Total penectomy" are listed as primary treatment options for patients with tumors staged "T2 or greater." Furthermore, the "Principles of Surgery" section (page PN-B) explicitly states that "Partial or total penectomy is necessary when there is invasion into the corpora cavernosum." This directly supports performing a penectomy for higher-stage tumors, particularly when there is invasion of the corpora cavernosa. |

**7. Primary Treatment (Radiotherapy)**

7.1 Radiotherapy (often brachytherapy) can be an alternative to surgery for selected patients with T1-T2 tumors (<4 cm), particularly those who are unfit for surgery. Patients should be informed of a potentially lower local control rate compared to surgery.

| AUA2020 | R | The guideline supports the recommendation. It states that radiation therapy, including brachytherapy, has been used for early-stage penile cancers (T1 and T2). The guideline specifically mentions, "Urologists may consider radiation for patients who are unfit for surgery," which directly aligns with the patient population in the recommendation. Furthermore, it acknowledges that radiotherapy has "lower control rates" and that surgery is the "preferred approach," which is consistent with the recommendation's framing of radiation as an alternative with potential drawbacks. |
| --- | --- | --- |
| DGU2020 | R | The guideline recommends local radiotherapy as an organ-preserving therapy for T1 and T2 tumors (Recommendation 7.19). It specifically presents brachytherapy as an alternative to surgery, stating it is equivalent regarding overall prognosis (Statement 7.20). Crucially, the guideline aligns with the recommendation's caveat by advising that patients be informed about the potentially lower local control rates of radiotherapy compared to surgery (Recommendation 7.21), with background text noting tumor size >4cm is a risk factor for recurrence. |
| EAU-ASCO2025 | R | The guideline strongly supports the use of radiotherapy as a treatment option for T1-T2 penile cancer. Section 6.1.2.9 specifically describes radiotherapy as an "organ-preserving approach with good results in selected patients with T1–2 lesions." The guideline aligns with the recommendation's details, noting that brachytherapy should be limited to tumors <4 cm and that local control rates may be slightly lower compared to surgery. This is further reinforced by a "Strong" recommendation on page 25 to "Offer radiotherapy to selected patients with biopsy-confirmed T1 or T2 lesions." |
| ESMO2024 | R | The guideline supports the recommendation, stating that for localized penile cancer (including T1 and T2 stages), treatment can include radiotherapy (RT), such as brachytherapy, in selected cases. The guideline specifies that optimal candidates for brachytherapy are those with tumors <4 cm, aligning with the recommendation's criteria. It further recommends brachytherapy as an alternative to surgery for pT1 disease and as an option for selected patients with low-volume T1-T2 disease, particularly if surgery is not an option. |
| NCCN2025 | R | The NCCN guideline recommends radiotherapy (RT) as a primary treatment option for T1-T2 tumors, aligning with the recommendation. On page PN-C, under "Primary Radiation/Chemoradiation Therapy," it specifies options for T1-2 tumors <4 cm, including brachytherapy (category 2B) and EBRT (category 2B). The guideline also supports the recommendation's caveat, with the discussion on page MS-9 noting that while survival rates are similar, penectomy offers higher local control rates compared to brachytherapy. Furthermore, the guideline mentions considering RT for patients who are not surgical candidates, reinforcing its role as an alternative to surgery. |

7.2 For T3–4 or N+ surgically unresectable disease, consider EBRT with concurrent chemotherapy to the penis and regional lymph nodes.

| AUA2020 | NA | The AUA Update Series guideline focuses explicitly on the "Treatment of Early Stage Squamous Cell Carcinoma of the Penis," covering topics like topical therapies, laser ablation, and organ-preserving surgery for Tis, Ta, and T1 disease. The recommendation addresses a different patient population: those with advanced (T3–4 or N+) and surgically unresectable disease. Since the guideline's scope is limited to early-stage cancer, it does not address the management of this advanced clinical scenario. |
| --- | --- | --- |
| DGU2020 | R | The guideline aligns with the recommendation for treating advanced, unresectable penile cancer. Specifically, Recommendation 7.46 (page 73) states that for T4 tumors, "neoadjuvant chemotherapy or radio-chemotherapy should be considered" ("Eine neoadjuvante Chemotherapie oder eine Radio-Chemotherapie sollte erwogen werden"). This supports the proposed approach of using concurrent chemoradiotherapy for surgically unresectable disease. The guideline further reinforces the use of multimodal therapy for patients with fixed (unresectable) lymph node metastases (N+) in section 7.2.3. |
| EAU-ASCO2025 | R | The EAU-ASCO guideline supports the recommendation to consider chemoradiotherapy for surgically unresectable disease. Section 6.1.3.2 states that for non-resectable disease that does not sufficiently respond to induction chemotherapy, "palliative chemo-radiotherapy is an option." Furthermore, the recommendation table in Section 6.1.5 explicitly advises to "Offer... chemo-radiotherapy to patients with non-resectable advanced primary lesions." This aligns directly with the proposed treatment for the specified patient population (T3-T4 or N+ unresectable). |
| ESMO2024 | R | The guideline supports the use of radiotherapy (RT) with concurrent chemotherapy for surgically unresectable disease. The management algorithm for inguinal lymph nodes recommends "RT with concurrent ChT" for patients with fixed (cN3) disease (Figure 3). Furthermore, in the context of advanced and metastatic disease, the guideline states that "Palliative RT or RT with concurrent ChT for sites requiring local control should be considered [V, C]." This aligns with the recommendation to use this combination therapy for unresectable T3-4 or N+ disease. |
| NCCN2025 | R | The guideline recommends this treatment approach for the specified patient population. On page PN-C, under "Principles of Radiotherapy," for "T3–4 or N+ (surgically unresectable)" disease, the guideline explicitly states to perform circumcision followed by "EBRT with concurrent chemotherapy... to whole penile shaft, pelvic lymph nodes, and bilateral inguinal lymph nodes." This directly aligns with the recommendation to use concurrent EBRT and chemotherapy for the penis and regional lymph nodes in this setting. |

7.3 Specific radiotherapy doses may include: • EBRT: 66-74 Gy in 2 Gy fractions. • Brachytherapy (HDR): 42-45 Gy in 12-14 fractions. • Brachytherapy (LDR/PDR): 60-65 Gy delivered continuously over ~5 days.

| AUA2020 | NA | The AUA Update Series guideline discusses radiation therapy, including external beam radiation and brachytherapy, as a treatment option for early-stage penile cancer, particularly for patients who are not surgical candidates. However, the guideline does not provide any specific details or recommendations regarding dosage, fractionation, or duration for these radiotherapeutic approaches. Since the treatment recommendation focuses entirely on specific dose regimens, which are absent from the guideline, the recommendation is not addressed. |
| --- | --- | --- |
| DGU2020 | NA | The guideline discusses local radiotherapy, including brachytherapy (LDR, PDR, HDR) and external radiation, as organ-preserving treatment options for T1, T2, and T3 tumors. It compares their efficacy to surgery and mentions their use in palliative settings. However, the guideline does not specify any exact dose ranges or fractionation schedules for curative-intent radiotherapy. The only dose mentioned (12 x 3 Gy) is an example for palliative hypofractionated radiotherapy, which is a different context than the curative doses proposed in the recommendation. |
| EAU-ASCO2025 | NA | The guideline recommends radiotherapy as an organ-preserving approach for selected patients with T1-T2 lesions. It mentions external beam radiotherapy (EBRT) and brachytherapy as valid options. However, the guideline does not specify the detailed dosing schedules for these modalities as proposed in the treatment recommendation. It only provides a "minimum dose of 60 Gy EQD2" for EBRT (Section 6.1.2.9) and offers no specific dose recommendations for any form of brachytherapy, making the specific regimens in the recommendation unaddressed. |
| ESMO2024 | R | The guideline fully supports the specific radiotherapy doses mentioned in the treatment recommendation. Under the "RT for primary disease" section, the guideline explicitly states that a typical EBRT course is 66-74 Gy in 2 Gy fractions. It also recommends a usual dose of 60-65 Gy delivered over 5 days for LDR/PDR brachytherapy and a frequently proposed schedule of 42-45 Gy in 12-14 fractions for HDR brachytherapy, directly aligning with the provided recommendation. |
| NCCN2025 | DR | The NCCN guideline recommends a different dose for External Beam Radiation Therapy (EBRT) and is less specific than the treatment recommendation. For primary tumors (T1-2, <4 cm), the guideline suggests a total EBRT dose of 65–70 Gy (PN-C, p. 19), which differs from the recommended range of 66-74 Gy. Furthermore, while the guideline lists brachytherapy as a treatment option, it does not specify any doses or fractionation schedules for either HDR or LDR/PDR modalities, unlike the very specific doses provided in the recommendation. |

**8. Primary Treatment (Multimodal)**

8.1 For non-resectable, locally advanced primary tumors (T4), consider induction/neoadjuvant chemotherapy followed by surgery for responders, or chemo-radiotherapy.

| AUA2020 | NA | The guideline explicitly states that its focus is on the "Treatment of Early Stage Squamous Cell Carcinoma of the Penis," covering stages such as carcinoma in situ, Ta, and T1. The treatment recommendation, however, is for non-resectable, locally advanced primary tumors (T4), which represents a much more advanced stage of the disease. As the provided guideline's scope is limited to early-stage cancer, it does not discuss or provide recommendations for the management of T4 tumors. |
| --- | --- | --- |
| DGU2020 | R | The guideline aligns with the treatment recommendation. Recommendation 7.46 states that for T4 tumors, neoadjuvant chemotherapy or radio-chemotherapy should be considered ("sollte erwogen werden"). The background information on page 73 further clarifies that for a non-resectable primary tumor, neoadjuvant chemotherapy is indicated ("angezeigt") to potentially improve local resectability. This directly supports the proposed approach of using induction chemotherapy followed by surgery, or chemo-radiotherapy, for non-resectable T4 tumors. |
| EAU-ASCO2025 | R | The guideline directly aligns with the treatment recommendation. Section 6.1.3.2, "Non-resectable disease," states that induction chemotherapy is used to downstage the disease and enable surgical resection in responders. The recommendation table on page 25 further confirms this by advising to "Offer induction chemotherapy followed by surgery to responders, or chemo-radiotherapy to patients with non-resectable advanced primary lesions," which mirrors the proposed treatment approach. |
| ESMO2024 | R | The guideline supports the recommendation to consider neoadjuvant chemotherapy for T4 tumors. The text explicitly states that "neoadjuvant ChT with up to four cycles of a triplet regimen... in all eligible patients with locally advanced (T4), inoperable, primary penile... SCC... should be discussed" (p. 8). While the recommendation also suggests chemo-radiotherapy as an option, the guideline primarily discusses this modality in the context of adjuvant treatment for nodal disease rather than as a primary treatment for non-resectable T4 tumors. However, the core recommendation for using neoadjuvant chemotherapy in this setting is clearly aligned. |
| NCCN2025 | R | The NCCN guideline supports the recommendation for treating non-resectable T4 primary tumors. On page PN-D, under "Principles of Systemic Therapy," it states that "Patients with initially unresectable (T4) primary tumors may be downstaged by response to chemotherapy," aligning with the use of induction chemotherapy followed by surgery for responders. Additionally, on page PN-C, under "Principles of Radiotherapy," for "T3–4... (surgically unresectable)" tumors, the guideline recommends EBRT with concurrent chemotherapy, which is consistent with the chemo-radiotherapy option in the recommendation. |

**9. Recurrent Disease**

9.1 Local recurrence after organ-sparing surgery should be treated according to the principles of primary tumor management. Small, non-invasive recurrences can be treated with salvage organ-sparing options.

| AUA2020 | R | The guideline supports treating local recurrence similarly to primary tumors. The treatment algorithm in Figure 4 explicitly outlines management for "Recurrent lesion at a different site" and "Recurrent lesion at the same site," listing many of the same organ-sparing options (laser, surgical excision, Mohs surgery, glans resurfacing) as it does for "De novo lesion." The text also states that more aggressive surgery like penectomy is an acceptable option for recurrent disease, and provides an example where recurrence after Mohs surgery was successfully managed with a repeat organ-sparing procedure. |
| --- | --- | --- |
| DGU2020 | R | The guideline aligns directly with the treatment recommendation. Specifically, recommendation 7.25 (page 61) states that "Local recurrences after organ-preserving operative therapy should be treated according to the same principles as primary tumors." The accompanying background information reinforces this by explaining that renewed therapy is sensible and that organ-preserving strategies are equally applicable for recurrences, which supports the recommendation to use salvage organ-sparing options. |
| EAU-ASCO2025 | R | The guideline explicitly supports the recommendation. Section 6.1.4, "Local recurrence after organ-sparing surgery," states that "A second organ-sparing procedure can be performed if there is no corpus cavernosum invasion" (page 24). This is reinforced by a specific recommendation in the table on page 25 to "Offer salvage organ-sparing surgery to patients with small recurrences not involving the corpora cavernosa." The guideline further differentiates treatment for larger recurrences, recommending partial or total amputation, which aligns with the principle of managing the recurrence based on its characteristics, similar to a primary tumor. |
| ESMO2024 | R | The guideline supports the recommendation for managing local recurrence. Under the "Recurrent disease" section, it states that "Recurrent disease on the glans without corpus cavernosum invasion can be managed with wide local excision, distal corporectomy, or in selected cases, brachytherapy may be an option." This directly aligns with the proposed approach. Furthermore, the guideline's specific recommendations include considering "salvage penile-sparing options" for recurrences without invasion of the corpora cavernosa, which is consistent with treating these recurrences according to the principles of primary tumor management. |
| NCCN2025 | R | The NCCN guideline aligns with the recommendation to treat local recurrence after organ-sparing surgery according to the principles of primary tumor management. The "Management of Recurrent Disease" algorithm (PN-9) explicitly states that for a "Recurrence of penile lesion after initial penile sparing treatment," clinicians should "Treat according to recurrence stage (PN-1 and PN-2)." Pages PN-1 and PN-2 detail the primary evaluation and treatment pathways, which include the very organ-sparing options mentioned in the recommendation for early-stage disease. |

9.2 For large local recurrences or those with invasion of the corpora cavernosa, partial or total penectomy is warranted.

| AUA2020 | R | The guideline supports the recommendation for using partial or total penectomy for recurrent and advanced disease. The "Surgery" section explicitly states, "More aggressive surgical approaches such as partial and total penectomy are acceptable options for patients with unfavorable histopathology or recurrent disease." Furthermore, the treatment algorithm in Figure 4 lists "Partial penectomy" and "Radical penectomy" as treatments for a "Recurrent lesion at the same site." |
| --- | --- | --- |
| DGU2020 | R | The guideline states that local recurrences after organ-preserving therapy should be treated according to the same principles as primary tumors (Section 7.1.6, Recommendation 7.25). For primary tumors with invasion of the corpora cavernosa (T3 tumors), the guideline recommends a partial penectomy, or a radical (total) penectomy in cases of extensive tumor involvement (Section 7.1.4, Recommendation 7.22). This approach directly aligns with the treatment recommendation to perform a partial or total penectomy for large local recurrences or those with invasion of the corpora cavernosa. |
| EAU-ASCO2025 | R | The guideline aligns with the treatment recommendation. Section 6.1.4, "Local recurrence after organ-sparing surgery," explicitly states, "For large or high-stage recurrence, partial or total amputation is required." Furthermore, the recommendations table on page 25 includes a specific guideline to "Offer amputative surgery to patients with large local recurrences or corpora cavernosa involvement," directly supporting the proposed treatment. |
| ESMO2024 | R | The guideline aligns with the treatment recommendation. In the "Recurrent disease" section, the guideline states that "More extensive disease involving the corpus cavernosum may require a partial or total penectomy." This is further reinforced in the formal recommendations section, which specifies that "Invasion of the corpora cavernosa warrants partial or total penectomy [IV, B]," directly supporting the proposed treatment. |
| NCCN2025 | R | The guideline recommends that recurrence of a penile lesion after initial treatment should be managed according to the recurrence stage (PN-9). According to the AJCC staging table, invasion of the corpora cavernosa is classified as T3 disease (ST-1). For T2 or greater disease, the guideline lists partial penectomy and total penectomy as primary treatment options (PN-2). Furthermore, under the Principles of Surgery, it explicitly states that "Partial or total penectomy is necessary when there is invasion into the corpora cavernosum" (PN-B), directly aligning with the recommendation. |

9.3 After radiotherapy, local recurrence should be treated with surgical salvage therapy.

| AUA2020 | R | The guideline supports this recommendation. It establishes surgery as the "preferred approach" for penile cancer due to higher success rates and lower complication rates compared to radiotherapy. The document explicitly states that "more aggressive surgical approaches such as partial and total penectomy are acceptable options for patients with...recurrent disease." This general principle for managing recurrence, combined with the stated preference for surgery over radiation, aligns with using surgical salvage for a recurrence after radiotherapy. |
| --- | --- | --- |
| DGU2020 | R | The guideline fully supports the treatment recommendation. In Chapter 7.1.6, "Behandlung von Lokalrezidiven" (Treatment of Local Recurrences), consensus-based recommendation 7.26 explicitly states, "Lokalrezidive nach Strahlentherapie sollen mit operativer Salvage-Therapie behandelt werden" (Local recurrences after radiotherapy should be treated with operative salvage therapy) (p. 61). This statement is a direct match to the proposed recommendation. |
| EAU-ASCO2025 | R | The guideline supports the recommendation to use surgical salvage therapy for local recurrence after radiotherapy. Section 6.1.2.9 on page 23 explicitly states, "Local recurrence after radiotherapy can be salvaged by surgery [170]." This directly aligns with the proposed treatment recommendation. |
| ESMO2024 | R | The guideline supports the recommendation. In a discussion on brachytherapy, it cites a study where salvage therapy for local recurrence resulted in a 100% local disease control rate. Additionally, the section on managing recurrent disease explicitly states that when feasible, "further surgery can help palliate these patients," detailing options like wide local excision or penectomy for recurrence at the primary tumor site. |
| NCCN2025 | R | The NCCN guideline recommends managing recurrent disease based on its location and prior treatment. The "Management of Recurrent Disease" algorithm (PN-9) specifically addresses local recurrence in the inguinal region. For patients who have had prior radiotherapy (RT), the guideline recommends subsequent therapies including inguinal lymph node dissection (ILND), which is a form of surgical salvage. |

9.4 After topical or laser therapy, local recurrence should be treated with a different treatment modality.

| AUA2020 | DR | The guideline suggests a different approach than the recommendation. The recommendation mandates using a different treatment modality for local recurrence after topical or laser therapy. However, the guideline provides examples where recurrence was successfully treated with the same modality; for instance, it cites a study where recurrences after Tm:YAG laser were "re-treated with Tm:YAG laser." Furthermore, the treatment algorithm for a "Recurrent lesion at the same site" lists laser therapy as a viable option, without restricting its use based on the initial treatment modality. |
| --- | --- | --- |
| DGU2020 | R | The guideline explicitly supports the treatment recommendation. In section 7.1.6, "Behandlung von Lokalrezidiven" (Treatment of Local Recurrences), recommendation 7.27 states that local recurrences after topical medicinal therapy or laser therapies should be treated with a different therapy modality ("Lokalrezidive nach topischer medikamentöser Therapie oder nach Lasertherapien sollen jeweils mit einer anderen Therapiemodalität behandelt werden"). This directly aligns with the proposed treatment approach. |
| EAU-ASCO2025 | DR | The guideline's stance on this recommendation is nuanced and differs depending on the initial treatment. For topical therapy, the guideline aligns with the recommendation, stating that if it fails, "it should not be repeated" (page 19). However, for recurrence after laser therapy, the guideline presents a different option, noting that "Repeat laser treatment for recurrence has been described in some series" (page 20). Because the guideline allows for the possibility of repeating laser therapy, it does not fully support the recommendation's absolute statement that a different modality should always be used for local recurrence. |
| ESMO2024 | R | The guideline aligns with the recommendation by stating that if topical treatment for premalignant disease (PeIN) fails, different modalities such as wide local excision or glans resurfacing (surgical options) should be considered. The guideline also notes the high (~50%) local recurrence rate after laser therapy, further underscoring the need for alternative treatment strategies upon failure or recurrence. This directly supports switching to a different treatment modality when initial therapies are unsuccessful. |
| NCCN2025 | DR | The guideline recommends that treatment for a recurrent penile lesion should be based on the stage of the recurrence, not necessarily by switching to a different modality. The algorithm for "Recurrence of penile lesion after initial penile sparing treatment" (PN-9) directs clinicians to "Treat according to recurrence stage (PN-1 and PN-2)." This approach means that if the recurrence is of a stage where topical or laser therapy is still an appropriate option (e.g., PeIN, Ta, or select T1a), the guideline does not forbid using that modality again, which contradicts the recommendation to always use a different treatment modality. |

9.5 Regional lymph node recurrence after initial surveillance should be managed according to the same principles as primary lymph node metastases (i.e., with ILND +/- systemic therapy).

| AUA2020 | NA | The guideline focuses on the treatment of "early stage" squamous cell carcinoma of the penis, detailing primary local therapies such as topical agents, laser ablation, and various organ-sparing surgical techniques. The recommendation, in contrast, addresses the management of regional lymph node recurrence after a period of surveillance, which is a more advanced stage of disease. While the guideline acknowledges the importance of evaluating inguinal lymph nodes at initial diagnosis, it does not provide any guidance on managing nodal recurrence found during follow-up. |
| --- | --- | --- |
| DGU2020 | R | The guideline aligns with the treatment recommendation. In section 7.2.4, consensus-based statement 7.49 explicitly states that regional lymph node recurrence after active surveillance should be treated according to the same principles as primary lymph node metastases. The background information further clarifies that the appearance of lymph node metastases during surveillance is therapeutically equivalent to primary metastatic penile cancer and should be treated accordingly. |
| EAU-ASCO2025 | R | The guideline aligns with the treatment recommendation. Section 7.2.3, "Regional recurrence," states that the highest rate of regional recurrence occurs in patients managed by surveillance. It explicitly recommends that "Regional recurrence requires timely treatment by rILND with (neo)adjuvant chemotherapy/chemo-radiotherapy," which directly supports the recommendation to manage this condition with ILND and systemic therapy. |
| ESMO2024 | R | The guideline supports the recommendation. It explicitly addresses the management of recurrent inguinal disease under the "Salvage ILND" section. The guideline recommends "salvage inguinal lymphadenectomy with myocutaneous flap reconstruction in recurrent inguinal disease" and states it should be part of a "multimodal treatment which should also include neoadjuvant or adjuvant ChT." This combination of surgery (ILND) and systemic therapy (ChT) aligns with the recommendation to manage recurrence using the same principles as for primary lymph node metastases. |
| NCCN2025 | R | The guideline recommends managing regional lymph node recurrence in a previously untreated inguinal region using the same principles as for primary nodal disease. The algorithm on page PN-9, "MANAGEMENT OF RECURRENT DISEASE," shows that for local recurrence in the inguinal region with "No prior inguinal lymphadenectomy or RT," treatment involves biopsy and, if positive, proceeds to inguinal lymph node dissection (ILND) and further management based on nodal stage, mirroring the primary treatment pathways. The discussion on page MS-17 further supports this, stating, "If no prior inguinal lymphadenectomy or RT was given, primary treatment for the management of ILNs can be followed." |

9.6 Regional lymph node recurrence after a prior radical lymphadenectomy should be managed with a multimodal approach, including neoadjuvant chemotherapy, surgery, and/or radiotherapy.

| AUA2020 | NA | The treatment recommendation pertains to the management of regional lymph node recurrence after a radical lymphadenectomy, which represents an advanced or recurrent disease state. The provided AUA guideline is explicitly focused on the "Treatment of Early Stage Squamous Cell Carcinoma of the Penis," as stated in its title and learning objectives. The document details various treatments for primary tumors (Tis, Ta, T1) but does not address the specific clinical scenario of managing nodal recurrence after a prior definitive lymph node dissection. |
| --- | --- | --- |
| DGU2020 | R | The guideline directly addresses the management of regional lymph node recurrence after a prior radical lymphadenectomy in section 7.2.4. Recommendation 7.50 explicitly states that this condition should be treated with neoadjuvant chemotherapy and operative therapy. The supporting text on page 76 further clarifies that lymph node recurrences can be influenced by a multimodal therapy consisting of neoadjuvant chemotherapy and salvage lymphadenectomy. This aligns with the treatment recommendation for a multimodal approach. |
| EAU-ASCO2025 | R | The guideline supports the recommendation for a multimodal approach to regional lymph node recurrence. Section 7.2.3, "Regional recurrence," explicitly states that "Regional recurrence requires timely treatment by rILND with (neo)adjuvant chemotherapy/chemo-radiotherapy" (page 38). This statement directly aligns with the recommendation's call for a multimodal approach involving radical inguinal lymph node dissection (surgery), neoadjuvant chemotherapy, and/or radiotherapy. |
| ESMO2024 | R | The guideline directly supports the recommendation for a multimodal approach to regional lymph node recurrence. In the "Salvage ILND" section, it states that salvage inguinal lymphadenectomy should be part of a "multimodal treatment which should also include neoadjuvant or adjuvant ChT" (page 9). Furthermore, under the "Recurrent disease" recommendations, it advises clinicians to "consider systemic ChT, EBRT, surgery or a combination" for regional recurrences in the inguinal and pelvic lymph nodes (page 11). |
| NCCN2025 | R | The guideline aligns with the recommendation for managing regional lymph node recurrence after a prior lymphadenectomy. In the "Management of Recurrent Disease" algorithm (PN-9), for patients with local recurrence in the inguinal region who have had a "Prior inguinal lymphadenectomy or RT," the guideline recommends a multimodal approach. The options listed are "Chemotherapy followed by ILND," "ILND," or "Chemo/RT," which directly correspond to the neoadjuvant chemotherapy, surgery, and radiotherapy components mentioned in the recommendation. |

**10. Lymph Node Management**

10.1 Following circumcision for a primary tumor, patients with moderately or poorly differentiated tumors (grades 2 and 3) will require inguinal node dissection.

| AUA2020 | R | The guideline directly supports the treatment recommendation. In the "SURGERY" section, the document states, "close follow-up is critical for patients with moderately or poorly differentiated tumors (grades 2 and 3), as these will require inguinal node dissection." This statement is nearly identical to the proposed recommendation, confirming that this course of action is advised for this specific patient population. |
| --- | --- | --- |
| DGU2020 | R | The guideline aligns with the recommendation for inguinal node dissection in patients with moderately or poorly differentiated tumors. It explicitly states that clinical surveillance is not sufficient for tumors of grade 2 (G2) or higher (p. 63). Furthermore, Recommendation 6.5 (p. 40) advises invasive investigation of the inguinal lymph nodes—either through modified inguinal lymphadenectomy or sentinel lymph node biopsy—for tumors from category pT1b onwards, a category that includes poorly differentiated tumors (p. 35). Therefore, the guideline supports invasive staging for G2 and G3 tumors. |
| EAU-ASCO2025 | DR | The guideline recommends a more nuanced, risk-stratified approach than the treatment recommendation. While it supports surgical staging for poorly differentiated (Grade 3) tumors, which are considered high-risk, it does not universally recommend it for all moderately differentiated (Grade 2) tumors. According to the guideline (page 17), moderately differentiated (G2) pT1a tumors are considered intermediate-risk, and the decision for surgical staging should be made on a case-by-case basis, balancing risk against morbidity. The recommendation's blanket statement for all G2 tumors is therefore not fully aligned with the guideline's detailed criteria. |
| ESMO2024 | DR | The recommendation suggests that all patients with grade 2 or 3 tumors require an inguinal node dissection. The guideline, however, proposes a more nuanced, risk-stratified approach for patients with clinically negative nodes (cN0). For intermediate-risk (pT1G2) and high-risk (pT1-4G3/G4) disease, the guideline first recommends a dynamic sentinel lymph node biopsy (DSLNB) or modified inguinal lymph node dissection. A radical dissection is only recommended if metastasis is confirmed by these initial, less invasive staging procedures, thereby avoiding unnecessary morbidity in node-negative patients. |
| NCCN2025 | DR | The NCCN guideline uses a risk-stratified approach for the management of non-palpable inguinal lymph nodes, which does not fully align with the provided recommendation. While the guideline does recommend inguinal lymph node dissection (ILND) or dynamic sentinel node biopsy (DSNB) for high-risk patients—including those with high-grade (Grade 3) tumors (pT1b) or any T2 or greater tumor—it recommends surveillance for low-risk patients (PN-3). A moderately differentiated (Grade 2) T1 tumor without other high-risk features would be classified as T1a and fall into the low-risk category, for which surveillance, not immediate dissection, is the recommended management. Therefore, the guideline differs by not universally recommending inguinal node dissection for all Grade 2 tumors. |

10.2 For palpable, mobile inguinal nodes (cN1-cN2), inguinal lymph node dissection (ILND) is recommended. The dissection may be fascial-sparing or radical, with preservation of the saphenous vein where possible.

| AUA2020 | NA | The guideline focuses on the management of "early stage" penile cancer, specifically addressing treatments for the primary tumor (Tis, Ta, T1) such as topical therapies, laser ablation, and organ-preserving surgery. While the document mentions that inguinal lymph nodes must be evaluated for lymphadenopathy, it does not provide any specific treatment recommendations for patients who present with clinically palpable, positive nodes (cN1-cN2). The recommendation to perform inguinal lymph node dissection (ILND) for palpable nodes is not discussed in this guideline. |
| --- | --- | --- |
| DGU2020 | DR | The guideline recommends a more aggressive approach than the treatment recommendation. For patients with palpable inguinal nodes (cN1/cN2) and confirmed metastases, the guideline explicitly recommends a radical inguinal lymphadenectomy (RIL) (Recommendation 7.35, Page 67). The description of the RIL procedure in the guideline includes the ligation of the great saphenous vein ("die V. saphena magna am Übergang zur V. femoralis ligiert") (Section 7.2.2.1, page 67). This directly contradicts the treatment recommendation's suggestion to preserve the saphenous vein where possible and its allowance for a less aggressive, fascial-sparing dissection. |
| EAU-ASCO2025 | R | The guideline strongly supports the recommendation. Section 6.2.2.1 states that radical inguinal lymph node dissection (ILND) is the standard of care for patients with cN1-2 disease. Furthermore, the recommendations table on page 28 explicitly advises offering either fascial-sparing ILND or open radical ILND (sparing the saphenous vein, if possible) for cN1 disease, and open radical ILND (sparing the saphenous vein, if possible) for cN2 disease, which directly aligns with the proposed treatment. |
| ESMO2024 | R | The guideline supports the recommendation. Under the section "Management of cN1-2 disease," the guideline states that patients with clinically doubtful inguinal lymph node disease should undergo radical inguinal lymphadenectomy if metastatic disease is confirmed. It further specifies that "Radical inguinal lymphadenectomy removes the superficial and deep inguinal LNs with preservation of the long saphenous vein and fascia lata where possible," which directly aligns with the recommendation. The formal recommendations section on page 9 reiterates this for cN1-2 disease. |
| NCCN2025 | DR | The NCCN guideline recommends a different initial management approach for palpable, mobile inguinal nodes compared to the treatment recommendation. The guideline (page PN-4) advocates for a percutaneous lymph node biopsy first to confirm malignancy in patients with unilateral, mobile nodes <4 cm. Inguinal lymph node dissection (ILND) is only recommended as a subsequent step if the biopsy is positive. This differs from the treatment recommendation, which suggests proceeding directly to ILND for all patients with cN1-cN2 disease. |

10.3 If intraoperatively suspicious nodes are removed during surgery for cN+ disease, frozen section analysis should be performed. If positive, an ipsilateral radical lymphadenectomy should be completed in the same session.

| AUA2020 | NA | The guideline focuses exclusively on the treatment of "early stage" penile cancer, detailing various organ-preserving approaches for the primary tumor such as topical therapies, laser treatment, and conservative surgery (e.g., circumcision, glansectomy). The recommendation, however, describes a specific intraoperative management strategy for clinically positive lymph nodes (cN+ disease), which represents a more advanced stage of cancer. The provided guideline does not discuss the surgical management of lymph nodes in detail, nor does it mention the use of intraoperative frozen section analysis to guide immediate radical lymphadenectomy. |
| --- | --- | --- |
| DGU2020 | R | The guideline fully supports the treatment recommendation. Recommendation 7.34 states that for intraoperatively suspicious inguinal lymph nodes, a frozen section analysis should be performed, and if the result is positive, an ipsilateral radical lymphadenectomy should be carried out. The background information on page 67 further specifies that this radical lymphadenectomy should be performed in the same session, mirroring the proposed recommendation exactly. |
| EAU-ASCO2025 | NA | The guideline does not address the specific recommendation of using intraoperative frozen section analysis on suspicious lymph nodes in clinically node-positive (cN+) patients to decide on completing a radical lymphadenectomy in the same session. For cN+ patients, the guideline instead recommends pre-operative confirmation of metastasis via image-guided biopsy before initiating treatment (Section 5.2.3). The guideline discusses the use of frozen section analysis, but only in the context of assessing the resection margins of the primary tumor during organ-sparing surgery (Section 6.1.2.2). |
| ESMO2024 | DR | The guideline proposes a different management strategy for clinically node-positive (cN+) disease compared to the recommendation. For palpable inguinal nodes (cN1-2), the guideline's algorithm (Figure 3) and text recommend confirming metastases pre-operatively with a fine-needle aspiration or biopsy, which is then followed by a radical inguinal lymphadenectomy. The guideline does mention using intraoperative frozen section analysis to guide a radical lymphadenectomy, but this is specifically recommended for high-risk, clinically node-negative (cN0) patients undergoing a modified dissection when a sentinel node biopsy is unavailable. |
| NCCN2025 | DR | The NCCN guideline recommends a different diagnostic and therapeutic pathway for clinically node-positive (cN+) disease. For palpable, non-bulky nodes, the guideline recommends a pre-operative percutaneous biopsy to confirm metastasis (PN-4). If the biopsy is positive, the guideline then recommends a bilateral inguinal lymph node dissection (ILND), not an ipsilateral one as suggested in the recommendation. The rationale provided in the discussion section is that in patients with a unilateral palpable node, approximately 30% will have positive contralateral nodes that are not palpable, thus warranting a bilateral procedure (MS-12). |

10.4 For bulky, fixed, or extensive inguinal nodes (cN3), neoadjuvant chemotherapy (NAC) with a cisplatin- and taxane-based regimen is preferred over up-front surgery.

| AUA2020 | NA | The treatment recommendation specifies management for bulky, fixed, or extensive inguinal nodes (cN3), which represents advanced nodal disease. The provided AUA guideline, as indicated by its title "Treatment of Early Stage Squamous Cell Carcinoma of the Penis" and its learning objective, explicitly focuses on early-stage cancer. The document details treatments for localized tumors (Tis, Ta, T1) but does not address the management of advanced cN3 nodal disease or the use of neoadjuvant chemotherapy in this specific context. |
| --- | --- | --- |
| DGU2020 | R | The guideline aligns with the treatment recommendation. Recommendation 7.44 states that patients with fixed inguinal lymph nodes (equivalent to cN3) should receive neoadjuvant chemotherapy. The background information on page 73 further explains that neoadjuvant chemotherapy is indicated for patients with large and fixed lymph node metastases because primary surgery is often not curative. Finally, Recommendation 7.47 specifies that the neoadjuvant chemotherapy should be a polychemotherapy regimen including paclitaxel (a taxane) and cisplatin, which matches the regimen mentioned in the recommendation. |
| EAU-ASCO2025 | R | The guideline explicitly supports the treatment recommendation. In the recommendations table for the management of cN3 disease, it states, "Offer neoadjuvant chemotherapy (NAC) using a cisplatin- and taxane-based combination to chemotherapy-fit patients with... extensive inguinal involvement (cN3), in preference to up-front surgery" (p. 31, 33). The text further elaborates that NAC is a "reasonable strategy" for cN3 patients, while upfront surgery is "discouraged in routine management" due to high complication rates (p. 30, Section 6.3.2). |
| ESMO2024 | R | The ESMO guideline supports the recommendation for neoadjuvant chemotherapy (NAC) in patients with bulky, ulcerated, or fixed inguinal nodes (cN3). The guideline specifically recommends that patients with cN3 disease should be considered for NAC with triplet regimens. The suggested regimens, such as TIP (cisplatin-paclitaxel-ifosfamide) and TPF (docetaxel-cisplatin-fluorouracil), are consistent with the cisplatin- and taxane-based approach mentioned in the recommendation. This NAC approach is advised prior to surgical consolidation, aligning with the recommendation that it is preferred over up-front surgery. |
| NCCN2025 | R | The NCCN guideline aligns with the recommendation for treating bulky, fixed, or extensive inguinal nodes (cN3). The algorithms on pages PN-5 and PN-6 show that for patients with unilateral fixed nodes or bilateral nodes, a positive biopsy leads to "Neoadjuvant TIP chemotherapy" followed by surgery (ILND/PLND). The "Principles of Systemic Therapy" section (PN-D, 1 of 5) explicitly states that the preferred neoadjuvant regimen is "TIP (paclitaxel, ifosfamide, and cisplatin)," which is a taxane- and cisplatin-based regimen, and it is preferred prior to surgery for these patients. |

10.5 Minimally-invasive ILND for cN1–2 disease should only be offered as part of a clinical trial. It is contraindicated for cN3 disease.

| AUA2020 | NA | The treatment recommendation provides specific guidance on the use of minimally-invasive inguinal lymph node dissection (ILND) for patients with cN1–3 disease. The provided guideline, "Treatment of Early Stage Squamous Cell Carcinoma of the Penis," focuses exclusively on managing the primary tumor in early stages (Tis, Ta, T1) through topical therapies, laser ablation, and organ-sparing surgery. While the guideline acknowledges the need for inguinal node evaluation, it does not discuss the management of clinically positive nodes or compare different surgical techniques for lymphadenectomy, such as minimally-invasive ILND. |
| --- | --- | --- |
| DGU2020 | DR | The treatment recommendation suggests that minimally-invasive inguinal lymph node dissection (ILND) for cN1-2 disease should only be performed within a clinical trial. The guideline, however, presents minimally invasive techniques (video-endoscopic and robot-assisted) as options that can be used to reduce the complication rates of open surgery (page 68). It states that studies have shown these procedures to be safe with lower morbidity and comparable short-term oncological outcomes, implying a broader acceptance than the "clinical trial only" restriction. The guideline does not explicitly contraindicate the procedure for cN3 disease, instead focusing on a neoadjuvant chemotherapy approach for these patients. |
| EAU-ASCO2025 | R | The guideline fully supports the treatment recommendation. For cN1–2 disease, the recommendations table on page 28 explicitly states, "Offer minimally-invasive ILND to patients with cN1–2 disease only as part of a clinical trial." For cN3 disease, the guideline states on page 31 that minimally-invasive techniques are "considered inappropriate," and the corresponding recommendations table states, "Do not offer video endoscopic inguinal lymphadenectomy." This aligns perfectly with the recommendation that the procedure is contraindicated for cN3 disease. |
| ESMO2024 | DR | The guideline recommends radical inguinal lymphadenectomy as the standard procedure for cN1-2 disease. While it acknowledges that minimally invasive (robotic or laparoscopic) approaches have demonstrated some benefits like reduced blood loss and shorter hospital stays, it explicitly states that the evidence is based on small patient series and is insufficient to conclude on oncological outcomes. The guideline does not endorse offering minimally invasive ILND, even in a clinical trial setting, instead recommending the established open radical procedure. |
| NCCN2025 | DR | The guideline's stance differs from the recommendation's restriction of minimally-invasive inguinal lymph node dissection (ILND) to clinical trials. The guideline's Discussion section (MS-11) acknowledges that "Data on robotic ILND are limited," but states that a systematic review has reported the approach is "safe and effective, with lower morbidity than open surgery when carefully selecting for patients with non-palpable or non-bulky inguinal nodes." By presenting it as a viable option for a select patient group without mandating a clinical trial, the guideline offers a less restrictive recommendation. |

10.6 Following a response to neoadjuvant chemotherapy, patients should undergo consolidative surgery, including radical inguinal lymphadenectomy and possible pelvic lymphadenectomy.

| AUA2020 | NA | The guideline is explicitly focused on the "Treatment of Early Stage Squamous Cell Carcinoma of the Penis," detailing local therapies such as topical treatments, laser therapy, and organ-sparing surgery for Tis, Ta, and T1 tumors. The treatment recommendation addresses a more advanced stage of disease requiring neoadjuvant chemotherapy followed by consolidative lymphadenectomy. This specific clinical scenario for advanced or node-positive cancer is outside the scope of the provided guideline, which therefore does not address this recommendation. |
| --- | --- | --- |
| DGU2020 | R | The guideline supports the recommendation for consolidative surgery following a response to neoadjuvant chemotherapy. Recommendation 7.48 states that if neoadjuvant chemotherapy leads to a response, surgery should be performed after a maximum of 4 cycles. The background information for recommendations 7.43 to 7.45 also discusses the use of neoadjuvant chemotherapy before performing a radical lymphadenectomy, which is the type of surgery specified in the treatment recommendation. This confirms a clear alignment between the guideline and the proposed treatment. |
| EAU-ASCO2025 | R | The guideline aligns with the treatment recommendation. Section 6.3.2 states that for cN3 patients who respond to neoadjuvant chemotherapy (NAC), "surgical resection to remove all residual disease utilising radical inguinal and PLND techniques is the preferred strategy." This is further supported by a "Strong" recommendation in Section 6.3.5 to "Offer surgery to patients responding to NAC in whom resection is feasible," directly mirroring the proposed treatment. |
| ESMO2024 | R | The guideline aligns with the recommendation by advising that for patients with bulky or ulcerated (cN3) disease, neoadjuvant chemotherapy should be followed by surgery in those who respond. Specifically, the guideline states that "Neoadjuvant chemotherapy (ChT) followed by ipsilateral radical inguinal and pelvic lymphadenectomy in responders is recommended." Furthermore, in the final recommendations section, it advises that responders to neoadjuvant chemotherapy "should be considered for consolidation surgery." |
| NCCN2025 | R | The guideline recommends consolidative surgery for patients who have a stable or responding disease following neoadjuvant chemotherapy. The algorithm for managing enlarged pelvic lymph nodes (PN-7) shows that surgical candidates who have a "Stable or clinical response" to neoadjuvant chemotherapy should proceed to "Consolidation surgery." A footnote defines this surgery as "bilateral superficial and deep ILND and unilateral/bilateral PLND." This is further supported in the discussion section (MS-13), which states that patients with a stable, partial, or complete response following systemic chemotherapy may benefit from surgical consolidation. |

10.7 Ipsilateral pelvic lymph node dissection (PLND) is recommended for patients with ≥2-3 positive inguinal nodes on one side or with extranodal extension.

| AUA2020 | NA | The AUA Update Series guideline focuses exclusively on the "Treatment of Early Stage Squamous Cell Carcinoma of the Penis," as stated in its title and learning objective. The provided text discusses management options for primary tumors classified as Tis, Ta, and T1. The recommendation, however, pertains to managing advanced nodal disease (multiple positive inguinal nodes or extranodal extension) with a pelvic lymph node dissection, a topic outside the stated scope of this early-stage guideline. |
| --- | --- | --- |
| DGU2020 | R | The guideline directly supports the treatment recommendation. Specifically, recommendation 7.36 on page 69 states that ipsilateral pelvic lymph node dissection should be performed for patients with "two or more affected inguinal lymph nodes" or with "capsule-penetrating lymph node metastases." These criteria directly correspond to the recommendation's indications of "≥2-3 positive inguinal nodes" and "extranodal extension," respectively. |
| EAU-ASCO2025 | R | The guideline aligns with the treatment recommendation. Section 6.2.3.1.1 identifies risk factors for pelvic nodal metastasis, stating that "the number of positive inguinal LNs (1–2 vs. 3, or more...)" and "the presence of extracapsular spread" are key predictors. The formal recommendation table on page 30 advises to "Offer...prophylactic ipsilateral pelvic lymphadenectomy" to patients if "three or more inguinal nodes are involved on one side" or "extranodal extension is reported." This directly supports the criteria outlined in the treatment recommendation. |
| ESMO2024 | R | The guideline aligns with the treatment recommendation. Under the "Recommendations" section for PLND, it explicitly states, "Unilateral pelvic lymphadenectomy is recommended in patients with two or more ipsilateral inguinal metastases, metastasis with a diameter of ≥30 mm or extranodal extension [III, A]." This directly supports performing the procedure for patients with two or more positive inguinal nodes or extranodal extension, as specified in the recommendation. |
| NCCN2025 | R | The NCCN guideline supports performing a pelvic lymph node dissection (PLND) for patients with an increased burden of inguinal nodal disease. The "Principles of Surgery" section (page PN-B) states that "PLND should be considered... in patients with ≥2 positive inguinal nodes on the ipsilateral ILND site or in the presence of extranodal extension." The discussion section (page MS-12) further recommends PLND for patients with three or more positive inguinal lymph nodes and notes that unilateral PLND is a feasible approach for three or fewer metastases. This directly aligns with the recommendation's criteria and laterality. |

**11. Systemic Therapy**

11.1 Recommended neoadjuvant chemotherapy regimens are cisplatin- and taxane-based triplets, such as TIP (Paclitaxel/Ifosfamide/Cisplatin) or TPF (Cisplatin/Paclitaxel/5-Fluorouracil).

| AUA2020 | NA | The guideline's stance is Not Addressed (NA). The document's scope is explicitly limited to the "Treatment of Early Stage Squamous Cell Carcinoma of the Penis," which primarily involves topical treatments, laser therapy, and various surgical excisions. The recommendation for specific neoadjuvant chemotherapy regimens like TIP or TPF is for advanced disease, a topic that falls outside the focus of this guideline. The guideline mentions neoadjuvant chemotherapy only once, in the context of laser therapy, referencing a different drug combination and concluding that its role is not clearly established. |
| --- | --- | --- |
| DGU2020 | R | The guideline aligns with the treatment recommendation. Recommendation 7.47 on page 74 explicitly states that neoadjuvant chemotherapy should be administered as a polychemotherapy consisting of either "Paclitaxel, Ifosfamid und Cisplatin" (the TIP regimen) or "Cisplatin, Paclitaxel und 5-Fluorouracil" (the TPF regimen). This directly supports the proposed recommendation to use these specific cisplatin- and taxane-based triplets. |
| EAU-ASCO2025 | R | The guideline supports the use of cisplatin- and taxane-based combinations for neoadjuvant chemotherapy. It specifically discusses phase II trials that evaluated the efficacy of the TIP (paclitaxel, cisplatin, and ifosfamide) and TPF (docetaxel, cisplatin, and 5-FU) regimens mentioned in the recommendation. The guideline concludes in its summary of evidence that "The available evidence favours a cisplatin- and taxane-based combination (doublet or triplet) as the preferred approach" (page 33), directly aligning with the proposed treatment. |
| ESMO2024 | R | The guideline fully supports the treatment recommendation. It explicitly states that for patients with locally advanced disease (cN3 fixed nodes), neoadjuvant chemotherapy with a triplet regimen should be considered. The guideline specifically names TIP (cisplatin–paclitaxel–ifosfamide) and TPF (docetaxel–cisplatin–fluorouracil) as the recommended regimens in this setting. |
| NCCN2025 | DR | The NCCN guideline recommends a more specific neoadjuvant approach than the one proposed. While it identifies TIP (paclitaxel, ifosfamide, and cisplatin) as the "Preferred Regimen" for neoadjuvant chemotherapy, aligning with part of the recommendation, it does not mention TPF. Furthermore, the guideline explicitly states that the 5-FU + cisplatin combination, a core component of the TPF regimen, is "not recommended for neoadjuvant setting," thereby differing from the recommendation which suggests TPF as a viable option. |

11.2 Adjuvant chemotherapy should be considered for patients with pN2 or pN3 disease following lymph node dissection.

| AUA2020 | NA | The AUA Update Series guideline focuses exclusively on the "Treatment of Early Stage Squamous Cell Carcinoma of the Penis," covering topics like topical therapies, laser therapy, and organ-preserving surgery for Tis, Ta, and T1 tumors. The treatment recommendation, however, pertains to adjuvant chemotherapy for advanced nodal disease (pN2 or pN3) following lymph node dissection. As the guideline's scope is explicitly limited to early-stage cancer, it does not address the management of patients with confirmed, high-volume lymph node metastasis. |
| --- | --- | --- |
| DGU2020 | R | The guideline aligns with the treatment recommendation. Specifically, in section 7.2.2.3, recommendation 7.37 states that adjuvant chemotherapy "can be performed" (kann ... erfolgen) for patients with involved inguinal lymph nodes pN2/pN3. This directly supports the proposal that adjuvant chemotherapy "should be considered" for this specific patient group. The background information further clarifies that the existing data primarily supports a recommendation for patients with a pN2/pN3 status. |
| EAU-ASCO2025 | DR | The guideline does not recommend considering adjuvant chemotherapy for all patients with pN2 or pN3 disease. It explicitly states there is "no strong data supporting the use of adjuvant chemotherapy to improve OS" and that a recent meta-analysis found no difference in survival. Instead of a broad recommendation, the guideline suggests a "balanced discussion of risks and benefits" for high-risk patients, particularly those with pathological N3 disease, based on weak evidence. This is a more cautious and selective approach than the treatment recommendation provided. |
| ESMO2024 | R | The guideline explicitly supports the treatment recommendation. In the recommendations section, it states that "Patients with pN2 and pN3 disease following LND should be considered for adjuvant ChT following surgery [III, B]." This statement is in direct alignment with the proposed recommendation to consider adjuvant chemotherapy for patients with pN2 or pN3 disease after lymph node dissection. |
| NCCN2025 | R | The NCCN guideline recommends considering adjuvant therapy, including chemotherapy, for patients with pN2 or pN3 disease. The algorithm "MANAGEMENT OF PALPABLE NON-BULKY INGUINAL LYMPH NODES" (PN-4) explicitly lists "Chemotherapy (category 2B)" as a potential treatment following lymph node dissection for patients with pN2-3 disease. Furthermore, the discussion section reinforces this, stating that for patients with pN2 or pN3 disease, "treatment may entail adjuvant...chemotherapy following ILND or PLND" (MS-14). |

11.3 Adjuvant radiotherapy (with or without chemo-sensitization) should be offered or considered for patients with high-risk pathological findings such as pN2/N3 disease or extranodal extension.

| AUA2020 | NA | The guideline is explicitly focused on the "Treatment of Early Stage Squamous Cell Carcinoma of the Penis," as stated in the title and learning objective. The treatment recommendation addresses adjuvant radiotherapy for high-risk pathological findings like pN2/N3 disease or extranodal extension, which represent advanced nodal disease. The provided guideline does not discuss the management of advanced nodal disease and therefore does not address this specific recommendation. |
| --- | --- | --- |
| DGU2020 | R | The guideline aligns with the treatment recommendation. Recommendation 7.42 states that while adjuvant radiotherapy after lymph node dissection cannot be generally recommended, it can be considered ("kann erwogen werden") for patients with a high risk of lymph node recurrence. The background information on page 72 further clarifies that high-risk factors include extranodal extension ("extranodaler Befall") and ≥ 2 lymph node metastases, which corresponds to the pN2/N3 disease mentioned in the treatment recommendation. This specific consideration for a high-risk subgroup is consistent with the proposal to offer or consider adjuvant radiotherapy for these patients. |
| EAU-ASCO2025 | R | The guideline recommends offering adjuvant radiotherapy, with or without chemo-sensitization, to patients with pN2/N3 disease. This directly aligns with the proposed treatment recommendation for patients with high-risk pathological findings like pN2/N3 disease or extranodal extension (ENE). The recommendations table on page 35 specifically states: "Offer adjuvant radiotherapy (with or without chemo sensitisation) to patients with pN2/N3 disease...". Although the guideline assigns this a "Weak" strength rating and notes the evidence is controversial, it still presents this as a treatment option for the specified high-risk group. |
| ESMO2024 | R | The guideline supports the treatment recommendation. Under the section "Adjuvant post-operative RT for regional LN metastases," it explicitly states that clinicians may consider adjuvant radiotherapy in combination with adjuvant chemotherapy for patients with pN3 disease. This aligns with the recommendation to offer or consider adjuvant radiotherapy (with or without chemo-sensitization) for patients with high-risk findings, including pN2/N3 disease and extranodal extension. |
| NCCN2025 | R | The NCCN guideline consistently supports the use of adjuvant radiotherapy in patients with high-risk pathological findings. Multiple sections recommend considering adjuvant radiotherapy (RT) or chemoradiotherapy (chemo/RT) for pN2-3 disease and/or the presence of extranodal extension following surgery (PN-4, PN-12). The "Principles of Radiotherapy" section (PN-C) further specifies adjuvant chemo/RT as a consideration for pN2–3 disease and for boosting areas of extracapsular extension, directly aligning with the provided recommendation. |

11.4 Definitive radiotherapy (with or without chemo sensitization) can be offered to patients who are unwilling or unable to undergo surgery.

| AUA2020 | R | The guideline aligns with the recommendation by identifying radiation therapy as a treatment option for a specific patient population. The text states, "Urologists may consider radiation for patients who are unfit for surgery and are willing to accept the late onset side effects associated with it." This directly supports offering radiotherapy to patients who are "unwilling or unable to undergo surgery," as specified in the recommendation. |
| --- | --- | --- |
| DGU2020 | R | The guideline presents surgery as the primary therapy for T3 and T4 tumors. However, it explicitly states that local radiotherapy can be performed as an alternative for T3 tumors (Recommendation 7.23) and should be considered for T4 tumors (Recommendation 7.24). Additionally, the section on palliative radiotherapy (7.3.2) indicates its use when intensive therapy is not possible due to the patient's condition or comorbidities, which aligns with the recommendation for patients "unable to undergo surgery". |
| EAU-ASCO2025 | R | The guideline directly supports the treatment recommendation. In the recommendations table under section 6.4.2.3, "Summary of evidence and guidelines for pre- and post-operative radiotherapy," the guideline explicitly states: "Offer definitive radiotherapy (with or without chemo sensitisation) to patients unwilling or unable to undergo surgery" (page 35). This language is nearly identical to the proposed recommendation, confirming that this approach is advised for this specific patient population. |
| ESMO2024 | R | The guideline supports this recommendation. It states that for localized penile cancer, treatment should be carried out with curative intent using either surgical resection or radiotherapy (RT). The text explicitly mentions that brachytherapy, a form of RT, is suitable for selected cases where "the patient does not want to undergo surgical intervention" and is also recommended "especially if surgery is not an option." This aligns with offering radiotherapy to patients who are unwilling or unable to have surgery. |
| NCCN2025 | R | The NCCN guideline supports the recommendation to offer definitive radiotherapy to patients who are unable or unwilling to undergo surgery. On page PN-C, under "Principles of Radiotherapy," the guideline lists External Beam Radiation Therapy (EBRT), with or without concurrent chemotherapy, as a primary treatment option for T1–2 tumors. It also specifically states to "Consider prophylactic EBRT to inguinal lymph nodes in patients who are not surgical candidates or who decline surgical management," directly aligning with the patient population described in the recommendation. |

11.5 For first-line treatment of metastatic disease, platinum-based combination chemotherapy is the preferred approach. Recommended regimens include TIP, TPF, or cisplatin + 5-FU.

| AUA2020 | NA | The guideline exclusively focuses on the "Treatment of Early Stage Squamous Cell Carcinoma of the Penis," as stated in its title and learning objective. It details local and organ-preserving therapies such as topical treatments, laser therapy, and various surgical excisions for non-invasive (Tis) and early-stage (Ta, T1) disease. The treatment recommendation, however, pertains to first-line systemic chemotherapy for metastatic disease, an advanced stage of cancer that is outside the scope of the provided guideline. |
| --- | --- | --- |
| DGU2020 | R | The guideline aligns with the recommendation by stating that chemotherapy for metastatic disease should be cisplatin-based (Recommendation 7.52) and given as a combination therapy (Recommendation 7.53). It specifically recommends a regimen of cisplatin, ifosfamide, and paclitaxel (TIP) (Recommendation 7.54) and alternatively, the combination of cisplatin and 5-fluorouracil (Recommendation 7.55). These are two of the three specific regimens mentioned in the treatment recommendation, indicating strong support for this approach. |
| EAU-ASCO2025 | R | The guideline aligns with the recommendation to use platinum-based combination chemotherapy as the preferred first-line treatment for metastatic disease. Section 6.5.2 states, "These data support the recommendation of platinum-based chemotherapy as the preferred approach to first-line palliative systemic therapy," and specifically mentions triplet regimens like TPF and TIP, as well as doublets like PF (cisplatin + 5-FU). This is formalized in the recommendations table in Section 6.5.4, which advises to "Offer patients with distant metastatic disease, platinum-based chemotherapy as the preferred approach to first-line palliative systemic therapy." |
| ESMO2024 | R | The guideline supports the use of platinum-based combination chemotherapy as the first-line treatment for metastatic penile cancer. Under the section "Palliative ChT for metastatic disease," it states that "First-line cisplatin-based combination ChT... is recommended for the treatment of metastatic disease." The guideline further identifies cisplatin plus 5-FU, TIP, and TPF as "reasonable choices for first-line systemic therapy," directly aligning with the regimens specified in the recommendation. |
| NCCN2025 | R | The guideline aligns with the treatment recommendation by identifying platinum-based combination chemotherapy as the standard first-line approach for metastatic penile cancer. The "Principles of Systemic Therapy" section explicitly lists TIP (paclitaxel, ifosfamide, and cisplatin) as the "Preferred Regimen" for first-line systemic therapy for metastatic/recurrent disease (PN-D 2 of 5). Additionally, it includes 5-FU + cisplatin as an "Other Recommended Regimen," directly supporting the core components of the proposed treatment. |

11.6 Bleomycin-containing chemotherapy regimens should not be used due to the risk of pulmonary toxicity.

| AUA2020 | NA | The guideline mentions a retrospective study where some patients received a chemotherapy regimen including bleomycin, vincristine, and methotrexate prior to CO2 laser excision. However, the text does not offer any recommendation for or against the use of bleomycin. Furthermore, it does not address the specific risk of pulmonary toxicity associated with bleomycin-containing regimens. |
| --- | --- | --- |
| DGU2020 | R | The guideline directly aligns with the treatment recommendation. Recommendation 7.56 (page 78) explicitly states, "Bleomycin-containing combination therapies should no longer be used because of toxicity" ("Bleomycin-haltige Kombinationstherapien sollten wegen der Toxizität nicht mehr verwendet werden"). Furthermore, the background text on page 79 reinforces this by declaring that the use of bleomycin-containing regimens is "obsolete due to pulmonary toxicity" ("Die Anwendung von Bleomycin-haltigen Schemata ist aufgrund der pulmonalen Toxizität obsolet"). |
| EAU-ASCO2025 | R | The guideline explicitly supports the recommendation. In Section 6.5.2, it mentions that for bleomycin-containing regimens, "Toxicity was a concern..., especially pulmonary toxicity from bleomycin" (page 35). This is formalized in the recommendations table in Section 6.5.4, which gives a "Strong" recommendation to "Do not offer bleomycin because of the pulmonary toxicity risk" (page 37). |
| ESMO2024 | R | The guideline supports the recommendation, stating that older chemotherapy regimens for metastatic disease, such as methotrexate-bleomycin-cisplatin, "have been largely superseded due to concerns over toxicity." This aligns directly with the recommendation to avoid bleomycin-containing regimens due to their high toxicity profile. The guideline reinforces that these older treatments are no longer standard of care. |
| NCCN2025 | R | The guideline explicitly supports the recommendation to avoid bleomycin. On page PN-D (2 of 5), under "First-Line Systemic Therapy for Metastatic/Recurrent Disease," it states, "Not recommended: Bleomycin-containing regimens are associated with unacceptable toxicity." The discussion section on page MS-17 further clarifies this by stating, "The Panel does not recommend regimens containing bleomycin because of high pulmonary-related toxicity," directly aligning with the recommendation's reasoning. |

11.7 For patients with progressive disease, enrollment in a clinical trial is the preferred option for subsequent-line therapy.

| AUA2020 | NA | The guideline focuses exclusively on the "Treatment of Early Stage Squamous Cell Carcinoma of the Penis," as stated in its title and learning objective. It details various local management options such as topical therapies, laser therapy, and organ-preserving surgery for non-invasive or early invasive disease (Tis, Ta, T1). The treatment recommendation, however, pertains to patients with "progressive disease" requiring "subsequent-line therapy," which implies a more advanced stage of cancer that is beyond the scope of this guideline. Therefore, the guideline does not address or provide recommendations for this patient population or the role of clinical trials in their management. |
| --- | --- | --- |
| DGU2020 | DR | The guideline states that there is no standard therapy for second-line treatment of progressive/metastatic penile cancer (Statement 7.57). Instead of preferring clinical trial enrollment, it suggests that paclitaxel monotherapy can be used as a second-line option (Recommendation 7.58). While the guideline generally mentions that patients should be informed about the possibility of participating in clinical trials, it does not prioritize this over other treatments for subsequent-line therapy. |
| EAU-ASCO2025 | R | The guideline strongly supports the recommendation. It explicitly states, "Offer patients with progressive disease under platinum chemotherapy the opportunity to enroll in clinical trials, including experimental therapies within phase I or basket trials," and assigns this a "Strong" strength rating. The text further justifies this by highlighting that "Effective second-line palliative chemotherapy regimens are lacking," making enrollment in clinical trials the preferred and recommended path for subsequent-line therapy. |
| ESMO2024 | R | The guideline aligns with the recommendation to consider clinical trials for progressive disease. In its recommendations for metastatic penile cancer, the guideline states that "Clinical trial enrolment is strongly recommended." More specifically, for patients with "no response or disease progression," it advises that "second-line systemic ChT or RT for local control and/or best supportive care or a clinical trial may be considered," directly supporting the use of clinical trials as an option for subsequent-line therapy. |
| NCCN2025 | R | The guideline aligns with the recommendation by explicitly stating that a clinical trial is the preferred regimen for subsequent-line systemic therapy in patients with metastatic or recurrent disease. The "Principles of Systemic Therapy" table (PN-D, 3 of 5) lists "Clinical trial" as the "Preferred Regimen" in this setting. This is further supported by the algorithm for managing metastatic disease (PN-10), which recommends considering a clinical trial for patients with disease progression. |

11.8 Subsequent-line therapy options may include paclitaxel monotherapy or, for biomarker-selected tumors (MSI-H, dMMR, TMB-H), immunotherapy such as pembrolizumab.

| AUA2020 | NA | The guideline focuses exclusively on the "Treatment of Early Stage Squamous Cell Carcinoma of the Penis," as stated in its title and learning objective. It details local and organ-preserving therapies such as topical agents (5-FU, imiquimod), laser therapy, and various surgical approaches. The treatment recommendation, however, pertains to systemic, subsequent-line therapies like paclitaxel and immunotherapy (pembrolizumab) for biomarker-selected tumors, which are typically used for advanced or metastatic disease. The guideline's scope does not extend to these advanced-stage treatments, and therefore it does not mention or discuss the specific agents or clinical scenarios in the recommendation. |
| --- | --- | --- |
| DGU2020 | R | The guideline aligns with the proposed subsequent-line therapy options. Guideline recommendation 7.58 (p. 78) explicitly states that paclitaxel can be used as monotherapy in the second line. Furthermore, the background text (p. 79) discusses immunotherapy with checkpoint inhibitors, suggesting that determining microsatellite status can be useful for considering immunotherapy, which corresponds to the biomarker-selected approach in the recommendation. Although the guideline notes there is no standard second-line therapy and evidence is limited, it presents both paclitaxel and biomarker-guided immunotherapy as valid considerations. |
| EAU-ASCO2025 | R | The guideline supports the recommendation for subsequent-line therapy. It explicitly mentions a retrospective study of paclitaxel single-agent chemotherapy as a second-line treatment, noting a 20% response rate (page 36). Regarding immunotherapy, the guideline identifies it as an emerging potential treatment for advanced penile cancer and strongly recommends enrolling patients with progressive disease into clinical trials, including experimental therapies in basket trials, which aligns with the recommendation's suggestion for biomarker-selected use (pages 36-37). |
| ESMO2024 | R | The guideline supports this recommendation for subsequent-line therapy. It notes that "Single-agent paclitaxel has been used in the second-line setting as it is well tolerated" (p. 10). For biomarker-selected patients, the guideline states that pembrolizumab has FDA approval for MSI-H, dMMR, and high TMB tumors and explicitly recommends that "In selected patients whose tumours have a high TMB, MSI-H or dMMR, the use of anti-PD-L1 immunotherapy can be considered" (p. 11). |
| NCCN2025 | R | The guideline aligns with the treatment recommendation. In the section "Subsequent-Line Systemic Therapy for Metastatic/Recurrent Disease," pembrolizumab is listed as a preferred regimen for patients with unresectable or metastatic tumors that are MSI-H, dMMR, or TMB-H (PN-D 3 of 5). The guideline also lists paclitaxel as a treatment option that is "Useful in Certain Circumstances" in this same setting. |

**12. Palliative & Supportive Care**

12.1 Patients with metastatic disease or requiring multimodal therapy should be discussed in an interdisciplinary tumor board.

| AUA2020 | NA | The guideline focuses exclusively on the "Treatment of Early Stage Squamous Cell Carcinoma of the Penis," as stated in its title and learning objective. The recommendation addresses the management of patients with metastatic disease, which is an advanced stage of cancer. Because the guideline's scope is limited to early-stage disease, it does not discuss or mention the management of metastatic patients or the use of an interdisciplinary tumor board. |
| --- | --- | --- |
| DGU2020 | R | The guideline strongly recommends the proposed treatment. Recommendation 7.43 explicitly states that "Patients with metastatic penile carcinoma and/or the necessity of multimodal therapy should be discussed in an interdisciplinary tumor board." The background information further clarifies that curative therapy for metastatic disease can only be achieved in a multimodal setting, and thus, therapeutic decisions should be made by an interdisciplinary tumor board. This principle is also established as a quality indicator (QI 7) for patient care. |
| EAU-ASCO2025 | R | The guideline strongly advocates for a multidisciplinary approach to penile cancer care, which aligns with the recommendation. Section 1.2 notes that the guideline panel itself is a "multi-disciplinary group," and the preface (Section 1.5) emphasizes a "holistic and multi-disciplinary approach" early in the patient pathway. More directly, Section 7.4 highlights that a key advantage of centralizing care is providing an environment where "multi-disciplinary discussion of cases can occur." Finally, a strong recommendation in Section 7.5 is to "Deliver penile cancer care as part of an extended multi-disciplinary team," which directly supports discussing complex cases, such as those with metastatic disease or needing multimodal therapy, in a tumor board setting. |
| ESMO2024 | R | The guideline aligns with the recommendation for a multidisciplinary approach in complex cases. Specifically, for patients with pT1 disease considering brachytherapy, it states they should be referred to specialist centers for "multidisciplinary consideration." This principle is also implicitly supported for advanced and metastatic disease, where the guideline discusses complex multimodal treatments involving surgery, chemotherapy, and/or radiation therapy, necessitating collaborative decision-making. |
| NCCN2025 | NA | The NCCN guideline provides detailed treatment algorithms and principles for managing metastatic penile cancer and using multimodal therapies, including chemotherapy, surgery, and radiation therapy. However, the document does not contain an explicit recommendation for or mention of discussing these complex cases in an interdisciplinary tumor board or multidisciplinary team meeting. While the nature of the therapies implies collaboration between different specialties, the specific procedural step of a formal tumor board discussion is not addressed. |

12.2 Palliative radiotherapy can be offered for symptom control in advanced disease (e.g., 30 Gy in 10 fractions).

| AUA2020 | NA | The AUA guideline explicitly focuses on the "Treatment of Early Stage Squamous Cell Carcinoma of the Penis," as stated in its title and learning objectives. Its content details management for non-invasive and early-stage disease (e.g., Tis, Ta, T1). The treatment recommendation, in contrast, concerns palliative radiotherapy for symptom control in advanced disease, a topic that falls outside the scope of this specific early-stage guideline. |
| --- | --- | --- |
| DGU2020 | R | The guideline supports the use of palliative radiotherapy for symptom control in patients with advanced penile cancer. Section 7.3.2 ("Palliative Strahlentherapie") explicitly states that palliative radiotherapy is used to improve symptoms and prevent complications in the palliative setting. The guideline suggests a hypofractionated approach with single doses of 3 to 5 Gy, providing an example of 12 x 3 Gy (36 Gy total), which is consistent with the recommendation's example of 30 Gy in 10 fractions (3 Gy per fraction). |
| EAU-ASCO2025 | R | The guideline strongly supports the use of palliative radiotherapy for symptom control in advanced penile cancer. Section 6.5.3, "Role of radiotherapy in palliation," states that it is "frequently necessary" and that "standard palliative regimens should be readily employed." Furthermore, the recommendations table in section 6.5.4 gives a "Strong" rating to the statement, "Offer radiotherapy for symptom control (palliation) in advanced disease," which directly aligns with the treatment recommendation. |
| ESMO2024 | R | The guideline supports the use of palliative radiotherapy for advanced penile cancer. In the "Recommendations" section for metastatic disease, it states, "Palliative RT or RT with concurrent ChT for sites requiring local control should be considered [V, C]." This aligns directly with the treatment recommendation to offer palliative radiotherapy for symptom control in advanced disease. |
| NCCN2025 | R | The NCCN guideline directly supports the treatment recommendation. On the "Principles of Radiotherapy" page (PN-C), under the specific heading "Palliative RT," the guideline states, "Consider a palliative dose of 30 Gy in 10 fractions." This aligns perfectly with the dose and fractionation schedule proposed in the recommendation for symptom control in advanced disease. |

12.3 For chronic lymphedema resulting from treatment, a complex decongestive therapy (lymphatic drainage, compression therapy, and skin care) should be performed.

| AUA2020 | NA | The guideline provides a comprehensive overview of primary treatment modalities for early-stage penile cancer, including topical therapies, laser therapy, surgery, and radiation. It discusses the epidemiology, diagnosis, and direct outcomes of these treatments. However, the document does not address the management of long-term or secondary complications resulting from these treatments, such as chronic lymphedema. Therefore, the specific recommendation for using complex decongestive therapy for lymphedema is not mentioned. |
| --- | --- | --- |
| DGU2020 | R | The guideline directly and explicitly supports the treatment recommendation. In section 8.3.3, "Symptom-oriented treatment of malignant lymphedema," recommendation 8.7 states that for chronic lymphedema in penile cancer patients, a "komplexe Entstauungsbehandlung" (complex decongestive therapy) should be performed. The guideline specifies this therapy includes the exact components mentioned in the recommendation: lymphatic drainage, compression therapy, and skin care. |
| EAU-ASCO2025 | R | The guideline aligns with the recommendation for treating chronic lymphedema with complex decongestive therapy. Section 7.3.5, "Lymphoedema," explicitly states that the "mainstay of treatment" includes advising patients on "good skin care, compression, exercise, massage, and elevation" (page 41). The following page further elaborates on "Manual lymphatic drainage in the form of specialised massage techniques" (page 42), directly supporting the key components of the proposed recommendation. |
| ESMO2024 | R | The guideline acknowledges that lower limb lymphedema is a common complication associated with inguinal and pelvic lymphadenectomy. In the "Follow-up, Long-Term Implications and Survivorship" section, it recommends that "dedicated lymphoedema teams can help reduce the risk of recurrent cellulitis, help with mobility and reduce the extent of lower limb and genital swelling." This aligns with the recommendation to perform complex decongestive therapy, as this is the standard care provided by such dedicated teams. |
| NCCN2025 | NA | The NCCN guideline for Penile Cancer provides comprehensive recommendations on the diagnosis, staging, and oncologic treatment of the disease, including surgery, radiotherapy, and systemic therapy. However, the document does not address the management of chronic post-treatment complications. There is no mention of lymphedema as a potential side effect or any discussion of therapies for it, such as complex decongestive therapy, lymphatic drainage, or compression. |

**13. Follow-up & Survivorship**

13.1 Close, regular, and risk-stratified follow-up is crucial after treatment, especially for the first 2-5 years, to detect recurrences and functional disorders.

| AUA2020 | R | The guideline strongly supports the recommendation for close and regular follow-up after treatment for early-stage penile cancer. It repeatedly emphasizes that "close postoperative follow-up is crucial" and explicitly states that "Close follow-up for at least 5 years is required to detect and re-treat any potential recurrences." This aligns with the recommendation's proposed 2-5 year timeframe. The guideline also supports a risk-stratified approach, noting that "More frequent follow-up is recommended for...higher risk cases" and providing specific follow-up schedules based on the treatment modality used. |
| --- | --- | --- |
| DGU2020 | R | The guideline strongly supports the recommendation for regular, risk-stratified follow-up. Guideline recommendation 8.1 states that penile cancer patients "should receive regular risk-stratified follow-up to detect both tumor recurrences and functional disorders." The background information further clarifies that recurrences occur up to five years after primary therapy, and therefore recommends intensive follow-up for the first two years, with reduced frequency from the third to the fifth year, directly matching the proposed timeframe. |
| EAU-ASCO2025 | R | The guideline strongly supports the recommendation for close, regular, and risk-stratified follow-up. Section 7.2.1 states that an "intensive follow-up regimen during the first two years, with a less intensive follow-up later for a total of at least five years" is supported by evidence that most recurrences happen within this period. Furthermore, Table 7.1 provides a detailed, risk-stratified follow-up schedule based on the type of primary tumor treatment and nodal status, recommending a minimum duration of five years. The guideline also emphasizes that follow-up is crucial for detecting recurrence and managing quality of life and functional issues. |
| ESMO2024 | R | The guideline strongly supports the recommendation for close and regular follow-up. In the section "FOLLOW-UP, LONG-TERM IMPLICATIONS AND SURVIVORSHIP," the guideline states that the majority of recurrences are detected within the first 2 years and explicitly recommends "Close follow-up every 3-4 months for the first 2 years following primary surgery is required to detect local recurrence." Furthermore, it mentions that regular follow-up allows for addressing "urinary and sexual dysfunction," aligning with the recommendation to monitor for functional disorders. The guideline also discusses varying risks of recurrence based on factors like nodal staging, which supports the principle of risk-stratified follow-up. |
| NCCN2025 | R | The NCCN guideline strongly supports the recommendation for close, regular, and risk-stratified follow-up. Page PN-8 provides a detailed "SURVEILLANCE SCHEDULE" that outlines specific follow-up protocols based on the initial treatment and pathologic lymph node status (pN0-N3). The recommended frequency of clinical examinations and imaging is highest in the first 2-4 years (e.g., every 3 or 6 months) and then decreases, directly aligning with the recommendation's emphasis on the first 2-5 years for detecting recurrence. The discussion on page MS-16 further reinforces this by citing evidence that most recurrences are detected within this timeframe. |

13.2 A general follow-up schedule is every 3-4 months for the first 2 years, then less frequently. Follow-up should extend for at least 5 years.

| AUA2020 | R | The guideline supports the recommended follow-up schedule. The appendix provides specific follow-up protocols for different laser modalities, including "every 3 months for the first 2 years" for Nd:YAG laser, which aligns with the recommended frequency. Furthermore, the section on surgery explicitly states that "Close follow-up for at least 5 years is required," which supports the total duration of follow-up mentioned in the recommendation. |
| --- | --- | --- |
| DGU2020 | R | The guideline recommends a risk-stratified follow-up schedule that is consistent with the treatment recommendation. Specifically, in the section "Nachbeobachtung und Nachsorgeschemata" (page 81-82), it states that an intensive follow-up is recommended for the first two years, with frequency reduced from the third to the fifth year. The provided tables detail this as follow-ups every 3 months for years 1-2, then every 6-12 months for years 3-5, which aligns with the recommendation's proposed schedule. |
| EAU-ASCO2025 | R | The guideline aligns with the recommendation for follow-up scheduling after penile cancer treatment. Section 7.2.1 states that follow-up for nodal metastases should be performed at "three-monthly intervals for the first two years," followed by a reduced frequency "for a total of five years of follow-up." Table 7.1 on page 38 further details this, recommending 3-monthly intervals for years 1-2 and less frequent intervals (6-monthly or annually) for years 3-5, with a minimum duration of 5 years. |
| ESMO2024 | R | The guideline aligns directly with the treatment recommendation. Under the "FOLLOW-UP, LONG-TERM IMPLICATIONS AND SURVIVORSHIP" section, the guideline explicitly states, "Close follow-up every 3-4 months for the first 2 years following primary surgery is required to detect local recurrence [IV, A]." This matches the frequency and initial duration proposed in the recommendation. The guideline's supporting text further notes that most recurrences occur within 5 years, with the majority detected in the first 2 years, justifying the intensified surveillance period. |
| NCCN2025 | R | The guideline supports the recommendation by providing several specific surveillance schedules that align with its general principles. On page PN-8, the guideline details follow-up frequencies that vary based on initial treatment, with some schedules recommending clinical examination every 3 months for the first 1-2 years (e.g., after topical therapy or for pN2/N3 disease). All provided schedules then decrease in frequency over time, consistent with the recommendation. Furthermore, the guideline's surveillance periods extend from 4 to 10 years, which encompasses the recommendation for at least 5 years of follow-up. |

13.3 Specific follow-up schedules after laser therapy are: • CO2 Laser: After 2 months, then every 3 months for 3 years, then biannually. • Nd:YAG Laser: Every 3 months for 2 years, then biannually.

| AUA2020 | R | The guideline's appendix, "Different laser modalities used for treatment of early stage penile cancer," explicitly outlines the same follow-up schedules mentioned in the recommendation. For the Carbon dioxide laser, it states, "Post-procedural follow-up after 2 months, then every 3 months for the first 3 years, and biannually thereafter." For the Nd:YAG laser, it recommends a "follow-up every 3 months for the first 2 years, biannually thereafter," which directly corresponds with the recommendation. |
| --- | --- | --- |
| DGU2020 | R | The guideline provides a general follow-up schedule for all organ-preserving therapies on page 82. This schedule recommends clinical examinations every three months for the first two years and then every six months for years three through five. The proposed follow-up schedules for both CO2 and Nd:YAG laser therapies are consistent with this general principle, recommending more frequent checks in the initial years before transitioning to biannual follow-ups. Since laser therapy is an organ-preserving treatment, the guideline's schedule supports the specific recommendation. |
| EAU-ASCO2025 | R | The guideline provides a follow-up regimen for all penile-preserving treatments, which includes laser therapy. Table 7.1 recommends surveillance every 3 months for the first two years, then every 6 months for years 3–5. This aligns well with the recommendation's specific schedules for both CO2 and Nd:YAG lasers, which also call for initial 3-monthly follow-ups followed by less frequent biannual checks. While the guideline does not differentiate between laser types, its general schedule for organ-sparing treatments is consistent with the principles and timing of the proposed recommendation. |
| ESMO2024 | DR | The guideline recommends CO2 laser ablation as a treatment for PeIN and highlights the importance of close clinical follow-up due to a high local recurrence rate. However, the guideline provides a general follow-up schedule for all primary surgery patients: "every 3-4 months for the first 2 years." This differs from the recommendation's more intensive and specific schedules, which vary based on the laser type (e.g., for CO2 laser: every 3 months for 3 years). Additionally, the guideline does not mention the Nd:YAG laser. |
| NCCN2025 | DR | The NCCN guideline recommends a single surveillance schedule for all patients who have received "Topical or local therapy," which includes laser therapy. According to the "Surveillance Schedule" on page PN-8, this involves a clinical examination every 3 months for the first 2 years, then every 6 months for years 3–5, and then annually for years 5–10. This approach differs from the treatment recommendation, which provides more frequent and laser-specific schedules (e.g., every 3 months for 3 years for CO2 laser) and does not mention a transition to annual follow-ups. |

13.4 Specific follow-up schedules based on treatment: • Organ Preservation: Every 3 mo (Yrs 1-2), every 6 mo (Yrs 3-5). • Total Penectomy: Every 3 mo (Yrs 1-2), annually (Yrs 3-5).

| AUA2020 | R | The guideline recommends a follow-up schedule for organ-preserving laser therapy that aligns with the treatment recommendation. The appendix details post-procedural follow-up for Nd:YAG laser therapy as "every 3 months for the first 2 years, biannually thereafter," which is identical to the proposed schedule for organ preservation. While the guideline does not specify a schedule for total penectomy, its recommendation for organ preservation is a direct match. |
| --- | --- | --- |
| DGU2020 | R | The guideline recommends the exact same follow-up schedule as the treatment recommendation. On page 82, the table "Empfohlenes Nachsorgeschema für den Primärtumor" specifies the follow-up intervals based on the initial therapy. For organ preservation ("Organerhaltung"), it lists every 3 months for years 1-2 and every 6 months for years 3-5. For total penectomy ("Totale Penektomie"), it recommends every 3 months for years 1-2 and annually for years 3-5, which is a direct match to the proposed recommendation. |
| EAU-ASCO2025 | R | The guideline's recommendations for follow-up schedules align perfectly with the proposed treatment recommendation. Table 7.1 on page 38, under "Recommendations for follow-up of the primary tumour," outlines the exact same intervals. For "Penile-preserving treatment" (organ preservation), it specifies a 3-monthly interval for years 1-2 and a 6-monthly interval for years 3-5. For "Amputation" (total penectomy), it recommends a 3-monthly interval for years 1-2 and an annual interval for years 3-5, matching the recommendation precisely. |
| ESMO2024 | R | The guideline supports the recommendation for frequent follow-up in the initial years post-treatment. It explicitly states that since most recurrences are detected within the first 2 years, "close clinical follow-up is required for the first 2 years after surgery." The guideline's specific recommendation is for "Close follow-up every 3-4 months for the first 2 years," which aligns with the recommendation's schedule of every 3 months for that period. While the guideline does not specify a follow-up schedule for years 3-5, its core advice for the most critical period is consistent with the proposed recommendation. |
| NCCN2025 | DR | The NCCN guideline suggests a different surveillance frequency for patients after partial or radical penectomy compared to the recommendation. The guideline (page PN-8) recommends clinical examinations every 6 months for the first two years after a partial or radical penectomy, followed by every 12 months for years 3-10. In contrast, the treatment recommendation suggests a more frequent schedule of every 3 months for the first two years. While both sources agree on the follow-up schedule for organ-preserving local therapies, this specific difference in post-penectomy surveillance makes the overall recommendation distinct from the guideline. |

13.5 Patients should be taught self-examination for early detection of recurrence and given easy access back to the clinic if concerns arise.

| AUA2020 | NA | The treatment recommendation advises teaching patients self-examination for the early detection of recurrence. The AUA guideline repeatedly emphasizes the critical importance of close, clinician-led follow-up to detect potential recurrences, recommending specific schedules for monitoring after various treatments. However, the guideline does not mention or discuss the specific practice of teaching patients to perform self-examinations as part of their post-treatment care. |
| --- | --- | --- |
| DGU2020 | R | The guideline explicitly supports the recommendation. Recommendation 8.2 on page 81 states that as part of patient education, self-examination for the early detection of recurrences should be taught to patients ("soll die Selbstuntersuchung zur frühen Detektion von Rezidiven ... den Patienten beigebracht werden"). Furthermore, the background information on page 82 advises that patients with self-observed changes should present themselves immediately and not wait for the next routine appointment, aligning with the principle of providing easy access back to the clinic. |
| EAU-ASCO2025 | R | The guideline strongly supports this recommendation. Section 7.2.2 states that "Patient education is an essential part of follow-up," and that local recurrence can be detected by the patient himself. Furthermore, the recommendations table in Section 7.5 explicitly advises to "recommend self-examination with easy access back to the clinic as local recurrence can occur late," which directly aligns with the proposed treatment recommendation. |
| ESMO2024 | NA | NAThe guideline's section on "FOLLOW-UP, LONG-TERM IMPLICATIONS AND SURVIVORSHIP" outlines the strategy for detecting recurrence. It recommends "close clinical follow-up every 3-4 months for the first 2 years," which should include clinical examination and imaging. However, the guideline does not mention teaching patients self-examination as part of the follow-up protocol. |
| NCCN2025 | NA | The NCCN guideline provides a detailed "Surveillance Schedule" (PN-8) that outlines the frequency of follow-up care after treatment. This schedule is based on clinician-led activities, specifically "Clinical examination," which includes examination of the penis and inguinal region by a professional. While the guideline emphasizes regular follow-up to detect recurrence, it does not mention or recommend teaching patients to perform self-examination as part of this process. |

13.6 Penile cancer care should be delivered by an extended multi-disciplinary team including specialists in urology, oncology, nursing, pathology, radiology, psychology, and lymphedema therapy.

| AUA2020 | NA | The guideline, which is an AUA Update Series lesson, focuses on the specific treatment modalities for early-stage penile cancer, including topical therapies, laser therapy, and surgical techniques. The recommendation advocates for a multi-disciplinary team approach to manage patient care. The provided guideline document does not address the structure of the care team or mention the concept of multi-disciplinary care. |
| --- | --- | --- |
| DGU2020 | NA | The guideline strongly supports a multi-disciplinary approach to penile cancer care. Recommendation 7.43 explicitly states that patients with metastatic disease or needing multimodal therapy should be discussed in an interdisciplinary tumor board. Furthermore, the guideline was developed by a large multi-disciplinary group of specialists, including those from urology, radio-oncology, medical oncology, pathology, and psycho-oncology, reflecting the specialists mentioned in the treatment recommendation. The guideline's stated goals also include improving interdisciplinary collaboration among care providers. |
| EAU-ASCO2025 | R | The guideline strongly recommends the treatment approach. Section 7.5 on page 43 explicitly states, "Deliver penile cancer care as part of an extended multi-disciplinary team," which directly aligns with the recommendation. The guideline then lists a comprehensive team of specialists, including urologists, specialist nurses, pathologists, radiologists, oncologists, lymphoedema therapists, and psychologists, covering all roles mentioned in the recommendation. This multidisciplinary approach is also emphasized in the preface (page 5) and as a key advantage of centralizing care (page 42). |
| ESMO2024 | R | The guideline supports the recommendation for a multi-disciplinary team approach to penile cancer care. The guideline itself is authored by a multi-disciplinary group of specialists in urology, oncology, pathology, and radiology. It explicitly advises "multidisciplinary consideration" for treatment decisions such as brachytherapy and highlights the role of psychological support and dedicated lymphedema teams during follow-up, aligning with the specific specialists mentioned in the recommendation. |
| NCCN2025 | R | The NCCN guideline strongly supports a multidisciplinary approach, which is fundamentally demonstrated by the composition of the guideline panel itself. The "NCCN Penile Cancer Panel Members" list (page 2) includes a diverse group of specialists, including those from urology, medical oncology, radiation oncology, and pathology, directly aligning with the core of the recommendation. The guideline's integrated treatment algorithms, which outline various modalities like surgery (PN-B), radiotherapy (PN-C), and systemic therapy (PN-D), necessitate the collaboration of these exact specialists for comprehensive patient care. |

13.7 Patients should be offered psychological support and counseling, and physicians should discuss the psychological and functional (urinary, sexual) impact of the disease and its treatments.

| AUA2020 | NA | The AUA guideline provides a detailed review of the clinical management of early-stage penile cancer, including epidemiology, diagnosis, and various treatment modalities such as topical therapies, laser therapy, and surgery. While the document mentions functional outcomes related to sexual function and micturition, it does not address the recommendation to offer patients psychological support or counseling. The guideline's focus is on the oncological and technical aspects of treatment, and it does not contain any discussion of the psychosocial care of the patient. |
| --- | --- | --- |
| DGU2020 | R | The guideline strongly recommends psycho-oncological care as an integral part of treatment for penile cancer. Recommendation 3.6 states that psychosocial counseling, support, and treatment should be available to all patients and their relatives throughout the course of the disease. Furthermore, Recommendation 8.5 specifies that sexual dysfunctions and limitations should be openly discussed in both curative and palliative contexts, directly aligning with the suggestion to discuss the functional impact of the disease and its treatments. |
| EAU-ASCO2025 | R | The guideline strongly supports the recommendation by dedicating a full chapter (Chapter 7) to "Follow-up and Quality of Life." Section 7.3.1 identifies access to psychological support and counseling as "critical components" of a holistic survivorship service. The summary of recommendations on page 43 reinforces this with a "Strong" rating for discussing the psychological impact with patients and offering counseling, as well as discussing the negative functional impacts on urinary and sexual function. |
| ESMO2024 | R | The guideline aligns with the recommendation by emphasizing the importance of addressing the non-oncological impacts of penile cancer and its treatment. In the "Follow-up, Long-Term Implications and Survivorship" section, it states that "Regular follow-up also allows patients to access psychological support and address urinary and sexual dysfunction." It further makes a formal recommendation that "Regular follow-up can provide psychological support and address sexual and urinary dysfunction as well as lymphoedema-related complications [IV, A]." |
| NCCN2025 | R | The NCCN guideline recommends considering "Best supportive care (NCCN Guidelines for Palliative Care)" for patients with metastatic disease (PN-10, PN-15). Palliative and supportive care inherently include psychological support, counseling, and managing the functional impacts of treatment. Additionally, the surveillance schedule (PN-8) directs clinicians to the NCCN Guidelines for Survivorship, which focus on managing the long-term physical and psychosocial effects of cancer and its treatment. This demonstrates that the guideline aligns with and supports the recommendation for comprehensive patient support. |

13.8 Patients should be assessed for lymphedema at follow-up and referred to lymphedema therapists early if it develops.

| AUA2020 | NA | The provided guideline focuses on the epidemiology, diagnosis, and various treatment modalities for early-stage penile squamous cell carcinoma, such as topical therapies, laser ablation, and surgical excision. While the text discusses follow-up protocols, their purpose is explicitly stated as monitoring for cancer recurrence. The document does not contain any information or recommendations regarding the assessment, management, or therapeutic referral for lymphedema as a potential post-treatment complication. |
| --- | --- | --- |
| DGU2020 | R | The guideline aligns with the recommendation by dedicating section 8.3.3 to the "Symptom-oriented treatment of malignant lymphedema." Recommendation 8.7 strongly advises that patients with chronic lymphedema should undergo complex decongestive therapy, which includes lymphatic drainage and compression therapy. This treatment is administered by specialized therapists, thereby supporting the recommendation's call for early assessment and referral. The guideline also notes in section 3.1.2 that patients should be informed about measures to avoid lymphedema, underscoring the importance of proactive management. |
| EAU-ASCO2025 | R | The guideline strongly supports the recommendation. Section 7.3.5 states, "Men should be assessed for genital and lower limb lymphoedema at each outpatient clinic appointment... Following nodal surgery, ideally, they would be referred to specialist lymphoedema services for assessment and management before any significant lymphoedema occurs" (p. 41). Furthermore, in the final recommendations table on page 43, the guideline explicitly states with a "Strong" rating to "assess patients for it at follow-up and refer to lymphoedema therapists early." |
| ESMO2024 | R | The guideline supports the recommendation by identifying lymphedema as a known complication of inguinal and pelvic lymphadenectomy. It explicitly states that follow-up should address "lymphoedema-related complications." Furthermore, it mentions that "dedicated lymphoedema teams can help" manage the condition, which aligns with the recommendation to refer patients to lymphedema therapists. |
| NCCN2025 | NA | The guideline provides a detailed surveillance schedule (PN-8) that outlines the frequency of clinical examinations and imaging for patients after treatment. However, the guideline does not specify that these follow-up assessments should include an evaluation for lymphedema. Additionally, there is no mention of managing potential treatment side effects like lymphedema or referring patients to lymphedema therapists. |

# Section 4: Model Consistency Check

## Prompt

**The following prompt instructed OpenAI’s GPT-5.1, and xAI’s Grok-4 to detect potential misinterpretations**

You are given the following three components:

- Guideline – a comprehensive clinical guideline on penile cancer treatment.
- Treatment Recommendation – a proposed approach or method for treating penile cancer.
- Extracted Stance – a previously determined stance that classifies the guideline's position toward the recommendation, including a concise justification.
- Your task is to evaluate whether the extracted stance accurately reflects the guideline's position on the treatment recommendation.
- Determine whether the extracted justification is correct and supported by the given context or contains errors.

**Treatment Recommendation**

RECOMMENDATION_TEXT

**Extracted Stance**

GUIDELINE_SNIPPET

**Guideline (context)**

GUIDELINE_TEXT

**Formatting Instructions**

Output in JSON format with keys: error (True/False) and suggestion (description on how to fix the error if error=True, otherwise empty if error=False).

Output Table: prompt_guidelines_consensus_table_assessment.tsv

# Section 5: Corrected Stance Classification

## Prompt

You are given the following components:

- Guideline – a comprehensive clinical guideline on penile cancer treatment.
- Treatment Recommendation – a proposed approach or method for treating penile cancer.
- Extracted Stance – a previously determined stance that classifies the guideline's position toward the recommendation.
- Correction Suggestions – an evaluative assessment of the guideline's position on the recommended treatment.
- Based on the correction suggestions, formulate a corrected version of the guideline's stance on the recommended treatment.

**Treatment Recommendation**

RECOMMENDATION_TEXT

**Extracted Stance**

GUIDELINE_SNIPPET_TEXT

**Guideline (context)**

GUIDELINE_TEXT

**Correction Suggestions**

SUGGESTION_TEXT

**Formatting Instructions**

Provide an updated classification code that can be one out of [R (Recommended); DR (Different Recommendation); NA (Not Addressed)] and an updated summary that justifies that classification referencing key elements from both the recommendation and the guideline.

Output Table: prompt_guidelines_consensus_table_correction

# Section 6: Recommendation-Extraction From Current Literature

## Prompt: Single Paper Extraction

Extraction of treatment-recommendations from current publications. The following prompt was used to instruct the LLM.

From the provided paper on penile cancer, identify and extract all explicit treatment recommendations. For each recommendation, ensure the following elements are included and clearly presented:

1. Aspect of Care: Categorize according to the relevant clinical area (e.g., diagnosis, primary treatment, lymph node management, systemic therapy, follow-up care).

2. Specific Clinical Indications:

- Stage-specific details: Clearly specify applicable T-stages (e.g., PeIN, Ta, Tis, T1, T2, etc.) or relevant classification (e.g., grade, invasive vs. non-invasive).
- Grading information: Include tumor grade where mentioned (e.g., G1/G2/G3).
- Anatomical specifics: Clarify the anatomical location (e.g., prepuce, glans, shaft).

3. Treatment Modality with Precise Conditions:

- Include precise treatment modality (e.g., laser therapy, wide local excision, glansectomy) along with qualification clauses (e.g., "if clinically feasible", "if facilities available", "not recommended after failed treatment").
- Indications of treatment limitations: Clearly state any limitations (e.g., "off-label use", "not to be repeated after failure").

4. Additional Clinical Clarifications:

- For recommendations involving lymph node management, explicitly clarify if they refer to palpable or non-palpable nodes.
- For topical therapies or surveillance, specify applicable stages (e.g., PeIN/Tis/Ta), and include any mandatory control assessments (e.g., biopsy after treatment).
- For follow-up, explicitly state if it applies after curative treatment and specify follow-up frequency and duration.

## Prompt: Unique Recommendation List

The following prompt instructs the LLM to merge all extracted recommendations by unifying them.

You are given a list of treatment recommendations for penile cancer. Your task is to identify and remove duplicates — that is, recommendations that convey the same clinical advice or intent, even if worded differently.

However, do not merge or group recommendations that represent distinct treatments, approaches, or clinical decisions, even if they appear under a shared heading or are closely related.

If multiple options are listed under a broader category (e.g., organ-sparing surgery), treat each bullet or treatment modality as a potentially distinct recommendation unless two entries are clearly semantically equivalent.

When removing duplicates, always retain the most comprehensive, specific, or medically precise version. But do not combine multiple unique recommendations into a single, summarized entry.

When in doubt, err on the side of treating treatments as separate unless they are clearly interchangeable.

When duplicates are merged, list all the source papers that provided this unified recommendation in the "Papers" column.

If a recommendation is unique (not merged), the "Papers" column should list only the source paper(s) where it appeared.

If multiple recommendations are merged, the "Papers" column should contain all relevant papers, separated by semicolons.

## Unique Recommendation List – Penile Cancer

| Aspect of Care | Recommendation | Papers |
| --- | --- | --- |
| **General Principles / Management** | All patients with penile cancer should be managed in centralized high-volume tertiary centers by an experienced multidisciplinary team to facilitate individualized management and optimize outcomes. | [1]; [2]; [3]; [4] |
| **General Principles / Management** | For patients with locally advanced disease who are kidney transplant recipients, treatment should be individualized to minimize graft injury, preferring non-cytotoxic options over radiotherapy and cytotoxic treatments. | [5] |
| **General Principles / Management** | For patients with Penile Metastasis (PM), surgery and chemotherapy are common treatment approaches. | [2] |
| **Cancer Prevention** | Voluntary, safe male circumcision (childhood/adolescent) is an effective strategy to reduce the incidence and persistence of penile HPV infection and prevent penile cancer. | [6]; [7]; [8] |
| **Cancer Prevention** | HPV vaccination, particularly early vaccination of boys (e.g., at ages 11-13) before the onset of sexual activity, is recommended to prevent infection with high-risk HPV. Vaccines covering HPV6 and HPV11 may be more adequate for preventing penile neoplasia. | [9]; [8] |
| **Cancer Prevention** | The use of male condoms is recommended as the most effective method for reducing the transmission of Sexually Transmitted Infections (STIs) like HPV and HIV, which are established risk factors for penile cancer. | [8] |
| **Complication Management** | **Primary Penile Surgery Complications:**• **Surgical Site Infections (SSIs):** Manage with empirical antibiotics, transitioning to culture-specific therapy, along with regular wet-to-dry dressing changes.• **Meatal Stenosis:** Manage with self-catheterization or formal surgical revision.• **Partial Skin Graft Loss:** Manage expectantly with observation.• **Total Skin Graft Loss:** Manage with wet-to-dry dressing changes followed by delayed regrafting. | [10] |
| **Complication Management** | **Inguinal Surgery Complications - Wound Issues:** • **Infected Inguinal Wounds:** Manage with meticulous wound care, daily inspection, and culture-specific antibiotics.• **Wound Dehiscence / Flap Necrosis:** Manage with wet-to-dry dressings or vacuum-assisted closure (VAC) dressings, followed by delayed closure. • **Patients with Large Body Habitus:** Place dry gauze ("Interdry") in groin folds to prevent moisture buildup. | [10] |
| **Complication Management** | **Inguinal Surgery Complications - Fluid Collections:** • **Refractory Seroma/Lymphocele:** Management may require image-guided drain placement, lymphatic embolization, or operative exploration. • **Lower Extremity Lymphedema:** Minimize risk with saphenous-vein sparing techniques, prevention of SSIs, early use of fitted compression devices, and a preemptive referral to a lymphedema specialist. | [10] |
| **Complication Management** | For patients who develop lower extremity lymphedema following inguinal lymphadenectomy, microsurgical lymphovenous bypass is a valuable prophylactic or therapeutic intervention. | [11] |
| **Diagnosis / Staging** | It is recommended to conduct p16 testing through Immunohistochemistry (IHC) as part of the initial diagnostic evaluation for all individuals diagnosed with Penile Squamous Cell Carcinoma (PSCC) to differentiate between HPV-associated and HPV-independent disease. | [12]; [13] |
| **Diagnosis / Staging** | For cases where Penile Verrucous Carcinoma (PVC) is highly suspected, repeated deeper biopsies that include the basement membrane of the papillomatous tumor are recommended to avoid misdiagnosis. | [14] |
| **Diagnosis / Staging** | Preoperative MRI is recommended for local staging of penile cancer, particularly to distinguish between T2 (spongiosum invasion) and T3 (cavernosa involvement) disease, to inform planning for organ-sparing surgery. | [15]; [4]; [16] |
| **Diagnosis / Staging** | Cross-sectional imaging (CT and MRI) is recommended as helpful for detecting metastases in the pelvis/retroperitoneum and in patients whose body habitus limits physical examination. | [17] |
| **Diagnosis / Staging** | For biopsy-confirmed Penile Verrucous Carcinoma (PVC) with no clinically palpable inguinal lymph nodes, further workups like CT or ultrasonography can be postponed initially. | [14] |
| **Diagnosis / Staging** | For patients with a high clinical suspicion of inguinal lymph node metastasis (ILNM), a comprehensive workup at the time of initial biopsy is suggested, including blood and tumor markers (e.g., CRP, NLR, PD-L1, p53, SCC-Ag) and advanced imaging. | [17] |
| **Diagnosis / Staging (Imaging Protocol)** | When performing preoperative local staging MRI, institutions may choose to omit artificial erection (induced by intracavernosal injection) from their protocols, as it has similar accuracy to MRI without it. | [15] |
| **Follow-up Care** | Close, strict follow-up is essential for all patients after treatment, particularly after penile-preserving surgery, local excision for PeIN, or a negative DSNB, due to the risk of recurrence. | [18]; [14]; [4]; [19] |
| **Follow-up Care** | Following curative Penile-Preserving Surgery (PPS), surveillance for local recurrence should be conducted every 3 months for the first 2 years, and then every 6 months until at least 5 years post-operatively. | [20] |
| **Follow-up Care** | Men with penile cancers demonstrating a p53 mutant pattern should be considered for a more intensive follow-up strategy. | [21] |
| **Follow-up Care** | Multidisciplinary follow-up with a psychologist trained in sexual therapy is recommended to aid in the recovery of post-operative sexual function and address psychosocial needs. | [22]; [23] |
| **Intraoperative Care** | **Principles to Reduce Postoperative Complications during ILND:** • **Tissue Handling:** Use atraumatic handling of anatomic structures. •**Hemostasis/Lymphostasis:** Perform careful control of lymphatics and small vessels. • **Incisions:** Use incisions that follow natural skin creases. • **Flap Creation:** Create the skin flap underneath the Scarpa fascia. • **Wound Closure:** Use myocutaneous flaps if primary closure is associated with excess tension or after prior radiotherapy. | [10] |
| **Intraoperative Management** | Intraoperative Frozen Section Examination (FSE) of surgical margins should be considered during surgery to confirm complete excision, which allows for more conservative macroscopic margins, maximal preservation of penile length, and a reduced risk of positive margins and local recurrence. | [20]; [23]; [4] |
| **Intraoperative Management** | **Specific Biopsy Protocols during Penile-Preserving Surgery (PPS) with FSE:** • **During glansectomy:** Biopsies of the complete circumference of the urethra and of each corporal tip should be sent for FSE. • **During total glans resurfacing:** Spongiosal biopsies of the deep margin should be taken for FSE. | [20] |
| **Intraoperative Management** | Following glansectomy, deep separate tunical biopsies should be obtained from the corpora to ensure an accurate pathological assessment of the deep surgical margins. | [4] |
| **Lymph Node Management** | For patients with low-risk penile cancer (e.g., Tis, Ta, T1a) and clinically non-palpable inguinal lymph nodes (cN0), surveillance/active monitoring is the recommended management strategy. | [24]; [17]; [25] |
| **Lymph Node Management** | For patients with intermediate or high-risk penile cancer (e.g., ≥pT1b/G2) and clinically non-palpable inguinal nodes (cN0), prophylactic surgical lymph node staging is recommended. Options include dynamic sentinel lymph node biopsy (DSNB) or inguinal lymph node dissection (ILND). | [17]; [26]; [27]; [28]; [15]; [29]; [30] |
| **Lymph Node Management** | For patients with clinically node-negative (cN0) penile cancer requiring surgical staging (indicated for intermediate- or high-risk tumors), Dynamic Sentinel Node Biopsy (DSNB) is the favored procedure, especially when performed in high-volume, experienced centers. | [31]; [19]; [30] |
| **Lymph Node Management** | For patients with clinically palpable, suspicious, or confirmed metastatic inguinal lymph nodes, radical inguinal lymph node dissection (ILND) is the standard of care. | [10]; [1]; [27]; [17]; [32]; [28]; [29] |
| **Lymph Node Management** | Early lymphadenectomy is recommended as it improves survival. Delaying ILND, particularly beyond 3 months in high-risk patients, should be avoided. | [1]; [33] |
| **Lymph Node Management** | Surveillance or delayed ILND for patients with clinically suspected positive nodes (cN+) is not recommended as it risks missing a curative opportunity. | [1] |
| **Lymph Node Management** | In patients diagnosed with metastatic inguinal nodes on histology following a DSNB, offering a completion radical inguinal lymph node dissection (RLND) is the standard treatment approach. | [19] |
| **Lymph Node Management** | For very selected groups of patients with a positive dynamic sentinel lymph node biopsy (DSNB), strict surveillance might be considered as a potential alternative strategy to immediate completion RLND, but better stratification methods are needed. | [19] |
| **Lymph Node Management** | Pelvic Lymph Node Dissection (PLND) should be performed after ILND for patients with pathological stage pN2 or predictors for pelvic lymph node involvement, such as three or more positive inguinal nodes or extranodal extension (ENE). | [1]; [34] |
| **Lymph Node Management** | Prophylactic inguinal lymphadenectomy is not an appropriate treatment for patients with Penile Verrucous Carcinoma (PVC) and non-palpable lymph nodes. | [14] |
| **Lymph Node Management** | For patients with Penile Verrucous Carcinoma (PVC) presenting with lymphadenopathy, treatment with anti-inflammatory drugs may be the treatment of choice, followed by a lymph node biopsy as needed. | [14] |
| **Lymph Node Management** | For low-stage (T1c N0 or below), clinically node-negative patients, guidelines do not recommend the removal of lymph nodes. | [24] |
| **Lymph Node Management** | The management of inguinal nodal disease in transplanted patients should be consistent with standard oncological guidelines. | [5] |
| **Lymph Node Management (Surgical Approach)** | Minimally Invasive Inguinal Lymphadenectomy (MIS-ILND), including video-endoscopic (VEIL) and robot-assisted (RA-VEIL) techniques, is recommended over traditional Open ILND to reduce morbidity. This approach has fewer complications and is considered oncologically equivalent for suitable patients (e.g., cN0 disease) when performed in high-volume centers. | [10]; [1]; [35]; [31]; [25]; [36]; [33]; [29] |
| **Lymph Node Management (Surgical Approach)** | For patients with cN1-2 disease, open surgery (O-ILND) should be the preferred option over minimally invasive techniques to reduce the risk of large lymph node fractures during dissection. | [29] |
| **Lymph Node Management (Surgical Approach)** | Modified Inguinal Lymph Node Dissection (ILND) is proposed for patients with high-risk primary penile cancer and clinically negative groins (cN0) to minimize morbidity. The technique involves limiting incision size, preserving the saphenous vein, and excluding dissection lateral to the femoral artery. | [10] |
| **Lymph Node Management (Surgical Technique)** | Indocyanine green (ICG) fluorescence-guided ILND is recommended for patients with cN0M0 disease undergoing staging ILND, as it improves the number of retrieved lymph nodes without increasing complications. | [30] |
| **Lymph Node Management (Surgical Technique)** | To reduce post-operative lymphorrhoea, intraoperative mapping and clipping of lymphatic channels using methylene blue is recommended during therapeutic inguinal dissection. | [37] |
| **Lymph Node Management (Surgical Technique)** | During inguinal lymphadenectomy, ligation of lymphatic vessels is the preferred technique over diathermic coagulation to reduce morbidity. | [27] |
| **Patient Counseling** | Patients undergoing surgery where Intraoperative Frozen Section Examination (FSE) is planned must be informed that discordance between FSE and final histology can occur, potentially leading to early local recurrence. | [20] |
| **Patient Counseling** | Before performing penile-preserving surgery, clinicians must ensure that patients are capable of and will commit to regular self-examination and attending scheduled follow-up appointments. | [4] |
| **Perioperative Care** | **Antibiotic Prophylaxis:** • Administer prophylactic antibiotics within 1 hour of incision. • Continue for 24 hours after primary penile surgery. • Continue until removal of surgical drains for ILND. | [10] |
| **Perioperative Care** | **Thromboprophylaxis (DVT/PE Prevention) for ILND:** • Use perioperative sequential compression devices. • Administer prophylactic low-molecular-weight heparin or direct oral anticoagulants. • Encourage early ambulation. | [10] |
| **Perioperative Care** | General patient management should include early ambulation post-surgery and optimization of nutritional support, especially for patients with poor health status. | [10]; [26] |
| **Perioperative Care** | Strict diabetes control is crucial for avoiding wound infection-related adverse events in patients undergoing ILND. | [26] |
| **Postoperative Care** | **Primary Penile Surgery - Wound and Catheter Management:** • **Dressing:** Use compressive surgical dressings for 2-7 days. • **Foley Catheter:** Typically remove around 7 days post-surgery for patients who undergo neomeatus creation or STSG application. | [10] |
| **Postoperative Care** | **Inguinal Lymph Node Dissection (ILND) - Specific Management:** • **Lymphedema Prevention:** Use compression stockings. • **Fluid Management:** Conduct daily measurement of "ins/outs" and regular stripping of surgical drains. • **Drain Management:** Maintain closed suction drains until output is <30-50 mL per 24 hours. • **Foley Catheter:** Typically remove on postoperative day 2. | [10]; [26] |
| **Primary Treatment** | Surgery is the standard, curative treatment for localized penile cancer. | [12]; [38]; [22]; [39] |
| **Primary Treatment** | Penile-preserving/organ-sparing surgery should be considered for all patients with localized penile cancer whenever clinically feasible to preserve organ form and function without compromising oncological outcomes. | [40]; [20]; [41]; [16]; [4] |
| **Primary Treatment** | For Penile Intraepithelial Neoplasia (PeIN) located on the prepuce, circumcision is the preferred treatment. | [18] |
| **Primary Treatment** | For Penile Intraepithelial Neoplasia (PeIN) / Carcinoma in Situ (Tis) of the glans, first-line options include organ-sparing approaches such as topical treatments (e.g., imiquimod, 5-fluorouracil) or laser treatments. | [18]; [3] |
| **Primary Treatment** | For Penile Intraepithelial Neoplasia (PeIN) of the glans after failure of a first-line topical or laser treatment, patients should be offered a second topical or laser treatment. | [18] |
| **Primary Treatment** | For extensive or recurrent PeIN of the glans after failure of first- and second-line treatments, surgical options like wide local excision or glans resurfacing should be offered. | [18] |
| **Primary Treatment** | For early-stage, superficial, or non-invasive penile cancer, treatment typically involves limited excision or non-invasive therapies like topical treatments. | [38]; [22] |
| **Primary Treatment** | For early-stage penile SCC, treatment options include Wide Local Excision (WLE), Mohs Micrographic Surgery (MMS), glansectomy, or partial penectomy. | [42] |
| **Primary Treatment** | For localized penile cancer (stages pTis, pTa, pT1), viable penile-sparing surgery options include circumcision (if limited to foreskin), wide local excision (WLE), laser ablation, glans resurfacing, and glansectomy. | [3]; [16]; [43] |
| **Primary Treatment** | Glansectomy is the indicated treatment and widely accepted procedure of choice for invasive penile cancer confined to the glans, including stage cT2 (invasion of glans spongiosum). | [16]; [4] |
| **Primary Treatment** | For invasive or advanced penile cancer, more extensive surgical intervention is required, such as partial or total penectomy. | [42]; [22]; [23]; [3] |
| **Primary Treatment** | For patients with T3 disease (invasion of corpora cavernosa), organ-sparing surgical approaches are usually precluded. Complete or partial penectomy is warranted. | [15] |
| **Primary Treatment** | For penile SCC (particularly smaller, T1 lesions), Mohs Micrographic Surgery (MMS) should be considered an effective option, as it is associated with a lower local recurrence rate compared to Wide Local Excision (WLE). | [42]; [40] |
| **Primary Treatment** | Radiation therapy is an alternative organ-preserving approach for primary tumors, particularly for T1-2 lesions < 4 cm in diameter. Brachytherapy may offer better recurrence-free rates compared to External Beam Radiotherapy (EBRT). | [3]; [34]; [40] |
| **Primary Treatment** | For patients with HPV-related PSCC, conservative surgical approaches, such as organ-preserving techniques, may be considered in appropriately selected cases given the favorable prognosis. | [13] |
| **Primary Treatment** | For Penile Verrucous Carcinoma (PVC) tumors < 3 cm and classified as Stage T1, local excision with minimal surgical margins followed by careful observation is recommended as the first-line treatment. | [14] |
| **Primary Treatment** | For tumors initially thought to be Penile Verrucous Carcinoma (PVC) but later considered not to be based on findings, the excision should be widened. | [14] |
| **Primary Treatment** | For Stage T2 penile cancers (defined as invasive cancers different from PVC), aggressive treatment is recommended. | [14] |
| **Primary Treatment** | For localized penile cancer in kidney transplant recipients, the surgical treatment should be consistent with established guidelines for the general population. | [5] |
| **Prognostic Assessment** | Testing of the tumour tissue for p53 expression is suggested to be useful for clinical planning to stratify patients and help inform management and follow-up strategy. | [28]; [21] |
| **Reconstructive Surgery** | For patients undergoing glansectomy who desire a neoglans, a split-thickness skin graft (SSG) can be used for reconstruction. | [16] |
| **Salvage Treatment** | Patients who experience a local recurrence or are found to have positive surgical margins after initial organ-sparing surgery can be offered a salvage penectomy. | [4] |
| **Screening** | General screening protocols for penile cancer are not recommended in kidney transplant recipients. | [5] |
| **Surgical Margins** | For penile-preserving surgery, conservative surgical margins of 3-5 mm (or a few millimeters) are considered acceptable to achieve oncological control, a reduction from historical recommendations. | [41]; [23]; [4] |
| **Surgical Margins** | When performing Wide Local Excision (WLE) for penile SCC, a 1 cm margin is often suggested to reduce recurrence. | [42] |
| **Surgical Technique** | If tumors extend into or macroscopically involve the distal corporal tips during glansectomy, a distal corporectomy should be performed. | [16]; [4] |
| **Systemic Therapy** | A multimodal therapeutic approach is typically warranted for patients with lymph node involvement (locally advanced and advanced PSCC). | [12] |
| **Systemic Therapy** | For unresectable tumours or disseminated metastatic disease, cytotoxic chemotherapy is employed, which can be used in a neoadjuvant or adjuvant context. | [38] |
| **Systemic Therapy (Adjuvant)** | Adjuvant chemotherapy (ACT) should be considered for patients with pathologically confirmed pN2-3 disease, as it may be of benefit, particularly for those with pN3 or pelvic lymph node metastases. | [1]; [44] |
| **Systemic Therapy (Adjuvant)** | Adjuvant radiotherapy (ART) should be considered after lymph node dissection for patients with pN2-3 disease, as it may reduce recurrence and improve survival. | [1]; [29] |
| **Systemic Therapy (Adjuvant)** | Adjuvant Chemoradiotherapy (ACRT) may provide a modest additional benefit in reducing locoregional recurrence and improving survival in pN3 disease, especially with extranodal extension (ENE) or HPV positivity. | [1] |
| **Systemic Therapy (Adjuvant)** | (Neo)adjuvant treatment (chemotherapy or radiotherapy) is not recommended for pN1 disease as it has no proven survival benefit. | [1] |
| **Systemic Therapy (Adjuvant)** | Adjuvant Radiotherapy (RT) following Pelvic Lymph Node Dissection (PLND) is not recommended as a routine practice and should be restricted to prospective controlled clinical trials. | [34] |
| **Systemic Therapy (First-Line Advanced/Metastatic)** | For advanced, locally advanced, or metastatic disease, first-line systemic therapy typically involves platinum-based chemotherapy, such as the TIP regimen (paclitaxel, ifosfamide, and cisplatin). | [45]; [3] |
| **Systemic Therapy (Investigational)** | The combination of Nivolumab and Ipilimumab appears unpromising for patients with PeCa. | [38] |
| **Systemic Therapy (Neoadjuvant)** | Neoadjuvant chemotherapy (NACT) is recommended for patients with fixed, bulky, or advanced nodal disease (cN2-3), particularly when not immediately resectable, with the goal of downstaging to enable subsequent surgical consolidation. | [1]; [44]; [29] |
| **Systemic Therapy (Neoadjuvant)** | Neoadjuvant Chemotherapy (NC) can be utilized when initial local control is desired, with the specific goal of enabling a subsequent, less aggressive radical local surgery. | [44] |
| **Systemic Therapy (Radiotherapy for Penile Metastasis)** | Radiotherapy for symptomatic Penile Metastasis (PM) is primarily palliative to manage pain, priapism, bleeding, and urinary dysfunction, with dosages from 25 Gy to 60 Gy depending on goals. | [2] |
| **Systemic Therapy (Radiotherapy for Penile Metastasis)** | Stereotactic body radiotherapy (SBRT), e.g., 25 Gy in 5 fractions, is a valuable approach for Penile Metastasis (PM) that can provide good symptomatic relief and local control, especially in oligometastatic disease. | [2] |
| **Systemic Therapy (Radiotherapy for Penile Metastasis)** | Adjuvant radiotherapy (e.g., 30 Gy in 10 fractions) can be considered after surgery for Penile Metastasis (PM) to help prevent recurrence. | [2] |
| **Systemic Therapy (for Refractory Disease)** | For patients with advanced or metastatic disease refractory to first-line chemotherapy, immunotherapy with immune checkpoint inhibitors (e.g., Avelumab, Nivolumab/Ipilimumab) may be considered, particularly for those with Microsatellite Instability-High (MSI-H) or high Tumor Mutational Burden (TMB-H). | [45]; [3] |
| **Systemic Therapy (Targeted / Immunotherapy - Investigational)** | Based on molecular profiles, the following are proposed: PARP inhibitors for defective DNA repair/MSI; mTOR inhibitors for *TP53*-mutated tumors; EGFR/COX2 inhibitors for tumors with alterations in *ADAM6/EGFR/COX2*; and imatinib for *PDGFA*-mutated tumors. | [46] |
| **Systemic Therapy (Targeted / Immunotherapy - Investigational)** | For patients with NOTCH1-mutated tumors, enrollment in clinical trials focused on PI3K/mTOR inhibitors is suggested. | [46] |

# Section 7: Stance Analysis Of The Guidelines Toward Literature-Derived Recommendations

## Prompt

You are given two texts:

- Treatment Recommendation - a proposed approach or method for treating penile cancer.
- Guideline - a comprehensive clinical guideline on penile cancer treatment.

Your task is to analyze the stance of the guideline toward the recommendation and classify it using one of the following labels:

- R (Recommended) - The guideline supports or aligns with the treatment recommendation.
- DR (Different Recommendation) - The guideline suggests a different treatment or approach compared to the recommendation.
- NA (Not Addressed) - The guideline does not mention or address the treatment recommendation at all.

Additionally, provide a concise justification (2–4 sentences) summarizing why you selected that stance, referencing key elements from both the recommendation and the guideline.

\# Treatment Recommendation:

RECOMMENDATION_TEXT

\# Guideline (context):

GUIDELINE_TEXT

\# Formatting Instructions:

Provide a classification code that can be one out of [R (Recommended); DR (Different Recommendation); NA (Not Addressed)] and a summary that justifies that classification referencing key elements from both the recommendation and the guideline.

## Output Table on Stance Classification

**1. General Principles / Management**

**1.1 All patients with penile cancer should be managed in centralized high-volume tertiary centers by an experienced multidisciplinary team to facilitate individualized management and optimize outcomes.**

[1]; [2]; [3]; [4]

| AUA2020 | NA | The treatment recommendation specifies that all penile cancer patients should be managed in centralized, high-volume tertiary centers by a multidisciplinary team. The AUA guideline focuses on the clinical treatment modalities for early-stage penile cancer, detailing various options like topical therapies, laser therapy, and different surgical techniques. However, the guideline does not make any statements or recommendations regarding the setting of care, such as the advantages of high-volume centers or the necessity of a multidisciplinary team approach. |
| --- | --- | --- |
| DGU2020 | R | The guideline aligns with the recommendation for managing penile cancer in specialized, multidisciplinary settings. It explicitly states that because penile cancer is rare, the option of presenting patients in "spezialisierten Zentren" (specialized centers) should be utilized (p. 44). Furthermore, it strongly supports a multidisciplinary team approach, recommending that patients with metastatic disease or requiring multimodal therapy be discussed in an interdisciplinary tumor board (Recommendation 7.43, p. 72), and includes this as a quality indicator (QI 7, p. 94). |
| EAU-ASCO2025 | R | The guideline strongly supports the recommendation to manage penile cancer in centralized, high-volume centers with a multidisciplinary team. Section 7.4, "Centralisation of penile cancer services," explicitly discusses the advantages of this approach, including improved survival, access to a larger team of specialists, and better adherence to guidelines. Furthermore, the first recommendation in the summary table in Section 7.5 is to "Deliver penile cancer care as part of an extended multi-disciplinary team," which is rated as "Strong." |
| ESMO2024 | R | The guideline recommends that patients considering specific treatments, such as brachytherapy for pT1 or T1-T2 disease, should be referred to "specialist centres for multidisciplinary consideration" (p. 9). This aligns with the recommendation's core principle of managing patients in specialized centers with multidisciplinary teams to optimize outcomes. While the guideline does not mandate this for every single patient, its repeated recommendation for referral to specialist centers for complex cases supports the overall strategy of centralized care. |
| NCCN2025 | R | The NCCN guideline strongly supports the principle of managing penile cancer at experienced, high-volume centers, particularly for complex procedures. While it does not make a blanket statement for all patients, it explicitly recommends that specialized techniques like Dynamic Sentinel Node Biopsy (DSNB) and brachytherapy be performed at "tertiary care referral centers with substantial experience" (MS-15, MS-11). The guideline further defines this for DSNB by suggesting centers where "at least 20 procedures are done per year," directly aligning with the recommendation's emphasis on high-volume, experienced, multidisciplinary settings to optimize outcomes (MS-11). |

**1.2 For patients with locally advanced disease who are kidney transplant recipients, treatment should be individualized to minimize graft injury, preferring non-cytotoxic options over radiotherapy and cytotoxic treatments.**

[5]

| AUA2020 | NA | The guideline focuses exclusively on the "Treatment of Early Stage Squamous Cell Carcinoma of the Penis," as stated in its title and learning objective. The recommendation, however, pertains to the management of "locally advanced disease," which is outside the scope of the provided text. While the guideline briefly mentions "transplant recipients" in the context of topical therapy for early-stage disease, it does not provide any guidance for treating locally advanced penile cancer in this specific patient population or address the need to balance treatment with potential graft injury. |
| --- | --- | --- |
| DGU2020 | NA | The guideline provides comprehensive recommendations for treating locally advanced penile cancer, including surgery, chemotherapy, and radiotherapy. While it generally acknowledges that treatment choices should be adapted based on patient comorbidities (p. 77, p. 80), it does not specifically mention kidney transplant recipients as a distinct patient group. The guideline offers no discussion or recommendations regarding the need to individualize therapy to minimize graft injury or the preference for non-cytotoxic options in this specific, immunosuppressed population. |
| EAU-ASCO2025 | NA | The guideline provides detailed treatment recommendations for locally advanced (T3-T4) penile cancer, including surgery, induction chemotherapy, and radiotherapy (sections 6.1.3 and 6.4). However, it does not address the specific clinical scenario of patients who are kidney transplant recipients. There is no mention of tailoring therapy to minimize graft injury or any discussion on the preference for non-cytotoxic versus cytotoxic or radiotherapeutic options in this specific patient population. |
| ESMO2024 | NA | The guideline does not address the specific patient population of kidney transplant recipients with locally advanced penile cancer. While it recommends selecting first-line chemotherapy for metastatic disease based on general patient comorbidities and performance status, it provides no specific guidance for managing treatment in transplant patients. The guideline does not discuss the need to individualize therapy to minimize graft injury or a preference for non-cytotoxic options in this context. |
| NCCN2025 | NA | The NCCN guideline provides comprehensive treatment algorithms for penile cancer based on disease stage and nodal status, including options for locally advanced disease such as surgery, radiotherapy, and various systemic chemotherapy regimens (e.g., PN-5, PN-C, PN-D). However, a thorough review of the guideline document, including the detailed discussion section, shows no specific recommendations or considerations for patients who are kidney transplant recipients. The guideline does not mention this patient population, the need to minimize graft injury, or a preference for non-cytotoxic over cytotoxic or radiotherapy options in this context. |

**1.3 For patients with Penile Metastasis (PM), surgery and chemotherapy are common treatment approaches.**

[2]

| AUA2020 | NA | The AUA guideline is explicitly focused on the "Treatment of Early Stage Squamous Cell Carcinoma of the Penis," covering non-invasive and early invasive stages (e.g., Tis, Ta, T1). The treatment options discussed, such as topical therapies, laser ablation, and organ-preserving surgeries, are all for localized disease. The recommendation, however, pertains to Penile Metastasis (PM), which is an advanced stage of cancer not covered within the scope of this guideline. |
| --- | --- | --- |
| DGU2020 | R | The guideline aligns with the recommendation that surgery and chemotherapy are common treatments for metastatic penile cancer. Section 7.3, "Management von metastasierten Stadien" (Management of metastatic stages), details the use of palliative systemic chemotherapy, with recommendations 7.53 and 7.54 advocating for combination chemotherapy in patients with good general condition. Furthermore, for patients with advanced lymph node metastases, the guideline recommends a multimodal approach, often involving neoadjuvant chemotherapy followed by surgery (e.g., Recommendation 7.48), thereby confirming both modalities as standard treatment approaches. |
| EAU-ASCO2025 | DR | The guideline addresses the treatment of distant metastatic disease (M1) under "Systemic and palliative therapies for advanced disease." It recommends platinum-based chemotherapy as the preferred first-line palliative approach for these patients. However, the guideline does not mention or recommend surgery for the treatment of distant metastases. Since the provided recommendation includes both surgery and chemotherapy, while the guideline only supports chemotherapy for this stage, it represents a different treatment strategy. |
| ESMO2024 | R | The guideline supports the recommendation. Under the "MANAGEMENT OF ADVANCED AND METASTATIC DISEASE" section, it explicitly states that palliative chemotherapy (ChT) is a primary intervention for metastatic inoperable disease, with platinum-based regimens being the "classical backbone of therapy". The guideline also discusses the role of surgery for recurrent disease, including regional recurrences in inguinal and pelvic lymph nodes, which constitutes a form of metastasis. |
| NCCN2025 | R | The guideline supports the recommendation that surgery and chemotherapy are common treatments for metastatic penile cancer. The "Management of Metastatic Disease" algorithm (PN-10) explicitly recommends starting with systemic chemotherapy. For patients who respond to chemotherapy, the guideline then recommends "Consolidation surgery," which is defined as lymph node dissection (ILND/PLND). The discussion on metastatic disease (MS-17) further reinforces this, stating that cisplatin-based chemotherapy should be considered and may facilitate curative resection. |

**2. Cancer Prevention**

**2.1 Voluntary, safe male circumcision (childhood/adolescent) is an effective strategy to reduce the incidence and persistence of penile HPV infection and prevent penile cancer.**

[6]; [7]; [8]

| AUA2020 | R | The guideline aligns with the recommendation by identifying a lack of circumcision as a risk factor for squamous cell carcinoma of the penis. It further supports this by stating that the prevalence of penile cancer is lower in countries that practice neonatal circumcision. This directly corroborates the recommendation's assertion that circumcision is an effective preventative strategy against penile cancer. |
| --- | --- | --- |
| DGU2020 | R | The guideline aligns with the treatment recommendation. In section 4.3 (Statement 4.1), it provides a consensus-based statement that "Circumcision in childhood and genital hygiene lower the individual risk of developing penile cancer." The background information for this statement further explains that childhood circumcision reduces the risk of carcinoma development by improving hygiene and reducing susceptibility to HPV, which is consistent with the rationale provided in the recommendation. |
| EAU-ASCO2025 | R | The guideline supports the recommendation that circumcision is a preventative strategy for penile cancer. Section 3.3, "Risk factors, prognosis, and prevention," explicitly states that "Neonatal circumcision reduces the incidence of penile cancer" (page 10). Furthermore, the text identifies phimosis, a condition preventable by circumcision, as being "strongly associated with invasive penile cancer." This information directly aligns with the recommendation's position on circumcision as an effective preventative measure. |
| ESMO2024 | R | The guideline supports the recommendation by stating that in countries where circumcision is a routine practice, penile cancer is "almost non-existent." It further identifies the prevalence of neonatal circumcision as one of the "most important" aetiological factors linked to the geographical distribution of penile cancer. This epidemiological evidence aligns with the recommendation that circumcision is an effective strategy for preventing the disease. |
| NCCN2025 | R | The guideline supports the recommendation by identifying "lack of neonatal circumcision" as a risk factor for penile cancer (PN-1, MS-3). The discussion section on "Risk Factors" (MS-3) explicitly states that "Neonatal circumcision is associated with a lower rate of penile cancer." The guideline further aligns with the recommendation's proposed mechanism by noting that this reduced incidence may reflect a "lower incidence and duration of HPV infections in this population." |

**2.2 HPV vaccination, particularly early vaccination of boys (e.g., at ages 11-13) before the onset of sexual activity, is recommended to prevent infection with high-risk HPV. Vaccines covering HPV6 and HPV11 may be more adequate for preventing penile neoplasia.**

[9]; [8]

| AUA2020 | NA | The AUA Update Series guideline focuses exclusively on the treatment of existing early-stage squamous cell carcinoma of the penis, detailing options such as topical therapies, laser therapy, and surgery. The recommendation, however, concerns the prevention of penile cancer through HPV vaccination in boys. While the guideline acknowledges HPV infection as a primary risk factor for developing penile cancer, it does not address or make any recommendations regarding vaccination as a preventative measure. |
| --- | --- | --- |
| DGU2020 | R | The guideline aligns with the treatment recommendation by explicitly stating that the risk of developing HPV-associated penile carcinoma can be reduced through HPV vaccination in boys. Specifically, Statement 4.2 on page 27 recommends this vaccination for boys aged 9–14 years, which corresponds well with the 11–13 age range suggested in the recommendation. Both documents emphasize the preventive nature of the vaccine against HPV-related penile cancer. |
| EAU-ASCO2025 | R | The guideline supports the recommendation for HPV vaccination. It identifies human papillomavirus (HPV) as the main risk factor for penile cancer and notes that up to 50% of invasive penile carcinomas are HPV-associated. While the guideline acknowledges a lack of definitive evidence for preventing invasive penile carcinoma specifically, it ultimately concludes that "HPV vaccination is encouraged" (page 10). This aligns with the recommendation's goal of preventing HPV infection as a primary preventative measure. |
| ESMO2024 | NA | The guideline does not address the recommendation for prophylactic HPV vaccination in young boys as a primary prevention strategy for penile cancer. The guideline discusses the role of HPV as an aetiological factor in penile cancer but its recommendations are focused on the diagnosis, treatment, and follow-up of existing disease. The only mention of HPV vaccination is in the context of treating patients already diagnosed with HPV-related penile intraepithelial neoplasia (PeIN), where it is presented as an option for unvaccinated men that requires further validation. |
| NCCN2025 | NA | The NCCN guideline for penile cancer is focused on the diagnosis, staging, and treatment of patients with suspected or confirmed disease. While the guideline explicitly identifies human papillomavirus (HPV) as a major risk factor for penile cancer (MS-3), it does not address primary prevention strategies. The document contains no discussion or recommendations regarding HPV vaccination for the prevention of penile cancer in the general population or in at-risk groups. |

**2.3 The use of male condoms is recommended as the most effective method for reducing the transmission of Sexually Transmitted Infections (STIs) like HPV and HIV, which are established risk factors for penile cancer.**

[8]

| AUA2020 | NA | The AUA guideline is focused on the diagnosis and treatment of existing early-stage penile cancer. While it identifies risk factors such as HPV infection and multiple sexual partners, its scope does not extend to primary prevention. The document details therapeutic options like topical agents, laser therapy, and surgery but does not address or make recommendations on preventative measures like condom use. |
| --- | --- | --- |
| DGU2020 | NA | The guideline identifies HPV infection as a major risk factor for penile cancer and discusses several preventative measures, including HPV vaccination, circumcision, and genital hygiene (p. 27). However, the guideline does not mention or discuss the use of condoms as a method for reducing the transmission of STIs like HPV in the context of preventing penile cancer. Since this specific recommendation is not addressed, the classification is NA. |
| EAU-ASCO2025 | NA | The guideline extensively covers "Risk factors, prognosis, and prevention" in Section 3.3, with a significant focus on HPV as the main risk factor for penile cancer. It discusses preventive measures such as HPV vaccination and neonatal circumcision (page 10). However, the document does not mention the use of condoms or any other barrier method for reducing the transmission of STIs as a strategy for penile cancer prevention. |
| ESMO2024 | NA | The guideline acknowledges human papilloma virus (HPV) as a significant risk factor for penile cancer. However, the document's scope is strictly limited to the diagnosis, treatment, and follow-up of patients with established penile cancer or premalignant disease. It does not address primary prevention strategies, such as the use of condoms to reduce the transmission of STIs like HPV. |
| NCCN2025 | NA | The guideline focuses on the evaluation, diagnosis, staging, and treatment of penile cancer in patients with a suspicious lesion or confirmed disease. While the guideline explicitly identifies sexually transmitted infections, including Human Papillomavirus (HPV) and Human Immunodeficiency Virus (HIV), as significant risk factors (page PN-1, page MS-3), it does not address primary prevention methods. Therefore, the recommendation to use condoms to reduce the transmission of these infections is not mentioned. |

**3. Complication Management**

**3.1 Primary Penile Surgery Complications: • Surgical Site Infections (SSIs): Manage with empirical antibiotics, transitioning to culture-specific therapy, along with regular wet-to-dry dressing changes. • Meatal Stenosis: Manage with self-catheterization or formal surgical revision. • Partial Skin Graft Loss: Manage expectantly with observation. • Total Skin Graft Loss: Manage with wet-to-dry dressing changes followed by delayed regrafting.**

[10]

| AUA2020 | NA | The guideline provides a comprehensive overview of the primary treatment options for early-stage penile cancer, such as topical therapies, laser ablation, and various surgical techniques. While it briefly mentions potential side effects of surgery, including wound contraction and issues with skin grafts near the urethral meatus, it does not offer any specific guidance on how to manage these or other postoperative complications. The recommendation details the management of specific complications like infections, meatal stenosis, and skin graft loss, which are topics not addressed within the scope of the provided guideline. |
| --- | --- | --- |
| DGU2020 | NA | The guideline acknowledges that complications such as meatal stenosis and the need for re-grafting can occur after penile surgery (p. 53). However, it does not provide any specific recommendations on how to manage these or other post-operative issues like surgical site infections or skin graft loss. The detailed management strategies proposed in the recommendation are not discussed in the guideline document. |
| EAU-ASCO2025 | NA | The EAU-ASCO guideline acknowledges that complications such as meatal stenosis and skin graft loss can occur following primary penile surgeries like glansectomy (page 22). However, the guideline does not provide the specific management strategies for these surgical complications as detailed in the recommendation. While the guideline mentions self-catheterization for meatal stenosis, it is in the context of radiotherapy complications, not primary surgery (page 23). The recommended management for surgical site infections and varying degrees of skin graft loss is not discussed. |
| ESMO2024 | NA | The ESMO guideline provides a comprehensive overview of the diagnosis, staging, and primary treatment of penile cancer, including detailed surgical and radiotherapeutic options. While the guideline acknowledges potential complications of treatment, such as meatal stenosis following radiotherapy or wound complications after inguinal lymphadenectomy, it does not provide specific recommendations for their management. The guideline does not address the post-operative management of surgical site infections, meatal stenosis, or partial/total skin graft loss resulting from primary penile surgery, which are the focus of the treatment recommendation. |
| NCCN2025 | NA | The NCCN guideline provides comprehensive recommendations for the diagnosis, staging, and oncologic treatment of penile cancer, including the types of surgical procedures to perform (e.g., penectomy, glansectomy, skin grafting). However, the guideline does not address the specific management of post-operative complications. The document does not contain information or recommendations on how to treat surgical site infections, meatal stenosis, or partial/total skin graft loss following primary penile surgery. |

**3.2 Inguinal Surgery Complications - Wound Issues: • Infected Inguinal Wounds: Manage with meticulous wound care, daily inspection, and culture-specific antibiotics. • Wound Dehiscence / Flap Necrosis: Manage with wet-to-dry dressings or vacuum-assisted closure (VAC) dressings, followed by delayed closure. • Patients with Large Body Habitus: Place dry gauze ("Interdry") in groin folds to prevent moisture buildup.**

[10]

| AUA2020 | NA | The guideline focuses exclusively on the diagnosis and treatment of the primary, early-stage penile tumor itself. It details various management strategies for the primary lesion, including topical therapies, laser ablation, and organ-preserving surgical techniques. The recommendation, however, provides specific guidance on managing post-operative wound complications that arise from inguinal surgery (lymphadenectomy). The guideline mentions the need to assess inguinal lymph nodes for staging but does not discuss the surgical techniques or the management of complications from inguinal dissection. |
| --- | --- | --- |
| DGU2020 | NA | The guideline acknowledges that complications such as wound infections ("Wundinfektionen") and skin necrosis ("Hautnekrosen") can occur after inguinal lymphadenectomy (p. 68). However, its discussion focuses on the prevention of these complications (e.g., intravenous antibiotic prophylaxis, wound drains) rather than the specific management once they arise. The detailed treatment strategies proposed in the recommendation, such as using culture-specific antibiotics for established infections, wet-to-dry dressings, or VAC dressings for dehiscence and necrosis, are not mentioned in the guideline. |
| EAU-ASCO2025 | R | The guideline supports the principles of the treatment recommendation. It explicitly identifies wound infections and skin necrosis as major complications following radical inguinal lymph node dissection (rILND) (page 27). Furthermore, Table 6.2 on morbidity factors lists "Prevention of surgical site infections, e.g., prophylactic antibiotics" as a measure with a positive impact, which aligns with the recommendation's use of antibiotics. The guideline also mentions vacuum dressings, a key part of the recommendation, though it classifies their benefit as "uncertain" (page 27, Table 6.2). |
| ESMO2024 | DR | The guideline's stance differs from the recommendation, particularly regarding the use of vacuum-assisted closure (VAC) dressings. The recommendation suggests VAC dressings for managing wound dehiscence and flap necrosis. In contrast, the guideline explicitly states that "Epidermal vacuum-assisted wound closure has not shown a net benefit for preventing post-operative complications and so is not currently recommended" (p. 6). While the guideline acknowledges the high morbidity and risk of wound infection associated with inguinal lymphadenectomy, it does not provide specific management details for these complications. |
| NCCN2025 | NA | The treatment recommendation provides specific instructions for managing postoperative inguinal wound complications like infections, dehiscence, and necrosis. The NCCN guideline acknowledges that inguinal lymph node dissection (ILND) is associated with "surgical morbidity" and "wound-related issues," and it mentions considering prophylactic antibiotics to reduce these risks (MS-11). However, the guideline does not provide any specific protocols or recommendations for managing these complications once they have developed. |

**3.3 Inguinal Surgery Complications - Fluid Collections: • Refractory Seroma/Lymphocele: Management may require image-guided drain placement, lymphatic embolization, or operative exploration. • Lower Extremity Lymphedema: Minimize risk with saphenous-vein sparing techniques, prevention of SSIs, early use of fitted compression devices, and a preemptive referral to a lymphedema specialist.**

[10]

| AUA2020 | NA | The provided guideline is an AUA Update Series lesson focused specifically on the "Treatment of Early Stage Squamous Cell Carcinoma of the Penis." Its scope covers the diagnosis and management of the primary penile lesion through topical therapies, laser ablation, and various surgical approaches to the penis (e.g., circumcision, glansectomy). While the guideline briefly mentions that higher-grade tumors require inguinal node dissection, it does not discuss the surgical techniques for this procedure or the management of its potential post-operative complications, such as seroma, lymphocele, or lymphedema, which are the specific subjects of the treatment recommendation. |
| --- | --- | --- |
| DGU2020 | R | The guideline aligns with the recommendation by addressing the complications of inguinal surgery. Section 7.2.2.1 and the background information on pages 66 and 68 explicitly mention lymphoceles and lymphedema ("Ödeme der unteren Extremität") as potential morbidities of inguinal lymphadenectomy. For lymphedema prevention and management, the guideline supports saphenous vein-sparing techniques (as part of modified lymphadenectomy) and recommends complex decongestive therapy, including compression and lymphatic drainage, for established lymphedema (Recommendation 8.7). While the guideline does not detail the specific management of refractory seromas, its focus on preventing and treating these known complications is consistent with the recommendation. |
| EAU-ASCO2025 | R | The guideline strongly supports the strategies for managing and preventing complications from inguinal surgery as outlined in the recommendation. It identifies lymphoedema, lymphocele, and seroma as significant morbidities of radical inguinal lymph node dissection (rILND) (page 27, section 6.2.2.4). Specifically for lymphedema, Table 6.2 (page 27) lists "saphenous sparing" and "prevention of surgical site infections" as having a positive impact. Furthermore, Section 7.3.5 (page 41) explicitly recommends compression and states that patients should ideally be referred to specialist lymphoedema services for assessment and management, aligning with a preemptive approach. |
| ESMO2024 | R | The guideline acknowledges that inguinal lymphadenectomy is associated with high morbidity, including debilitating lymphedema. It recommends managing these complications through follow-up and the involvement of "dedicated lymphoedema teams," which aligns with the recommendation's proposal for referral to a lymphedema specialist. Although the guideline does not specify management for seroma or lymphocele, its overall focus on addressing lymphoedema-related complications is consistent with the recommendation. |
| NCCN2025 | NA | The guideline acknowledges that inguinal lymph node dissection (ILND) is associated with "concerns of surgical morbidity" and discusses techniques to reduce this risk, such as preservation of the saphenous vein and the use of closed suction drains post-operatively (MS-11). However, it does not provide specific recommendations for managing established or refractory complications once they occur. The guideline does not mention the management of refractory seroma/lymphocele with drains, embolization, or exploration, nor does it detail the use of compression devices or specialist referral for lymphedema as outlined in the treatment recommendation. |

**3.4 For patients who develop lower extremity lymphedema following inguinal lymphadenectomy, microsurgical lymphovenous bypass is a valuable prophylactic or therapeutic intervention.**

[11]

| AUA2020 | NA | The guideline focuses on the treatment of early stage primary penile tumors, covering options such as topical therapies, laser ablation, and various organ-sparing surgical techniques. While it mentions the need to evaluate inguinal lymph nodes and perform dissection for higher-grade tumors, it does not discuss the management of complications arising from such procedures. The recommendation for microsurgical lymphovenous bypass to treat lymphedema is a specialized intervention for a sequela of nodal surgery, a topic that falls outside the scope of this guideline. |
| --- | --- | --- |
| DGU2020 | DR | The guideline suggests a different approach for managing lymphedema after inguinal lymphadenectomy. The recommendation proposes microsurgical lymphovenous bypass, which is an operative intervention. In contrast, the guideline's section 8.3.3 explicitly recommends conservative management consisting of complex decongestive therapy (lymphatic drainage, compression therapy, and skin care). Furthermore, on page 89, the guideline states that an operative intervention on the lymphatic system is not indicated ("Eine operative Intervention am Lymphabflusssystem ist bei MLÖ nicht indiziert"). |
| EAU-ASCO2025 | DR | The guideline suggests a different primary approach for managing lymphedema. It identifies non-surgical methods such as "good skin care, compression, exercise, massage, and elevation" as the "mainstay of treatment" (page 41, Section 7.3.5). While the guideline does mention surgical options, it explicitly states there is "limited evidence for the benefit of other surgical interventions such as... lymphatico-venous anastomosis in penile cancer" (page 42). This cautious stance, emphasizing limited evidence, contrasts with the recommendation's claim that the procedure is "valuable," positioning non-surgical therapies as the standard recommended approach. |
| ESMO2024 | NA | The guideline acknowledges that inguinal and pelvic lymphadenectomy is associated with lower limb lymphedema. It recommends management by "dedicated lymphoedema teams" to help reduce swelling, the risk of recurrent cellulitis, and mobility issues. However, the guideline does not mention or discuss the specific intervention of microsurgical lymphovenous bypass as a prophylactic or therapeutic option for this condition. |
| NCCN2025 | NA | The NCCN guideline provides detailed recommendations for the diagnosis, staging, and oncologic treatment of penile cancer, including the surgical management of inguinal lymph nodes (ILND). While the guideline acknowledges that ILND is associated with "concerns of surgical morbidity" (MS-11), it does not discuss the specific management of long-term complications such as lower extremity lymphedema. The document does not mention or provide any recommendations regarding microsurgical lymphovenous bypass or other interventions for treating lymphedema. |

**4. Diagnosis / Staging**

**4.1 It is recommended to conduct p16 testing through Immunohistochemistry (IHC) as part of the initial diagnostic evaluation for all individuals diagnosed with Penile Squamous Cell Carcinoma (PSCC) to differentiate between HPV-associated and HPV-independent disease.**

[12]; [13]

| AUA2020 | NA | The AUA guideline provides a comprehensive overview of the diagnosis and treatment of early-stage squamous cell carcinoma of the penis. While it identifies HPV infection as a primary risk factor in the epidemiology section, the "Diagnosis" section focuses on physical examination, tissue sampling, and imaging for staging. The document does not mention or recommend p16 testing via Immunohistochemistry (IHC) as a part of the diagnostic workup to differentiate between HPV-associated and HPV-independent disease. |
| --- | --- | --- |
| DGU2020 | R | The guideline strongly recommends (Empfehlungsgrad A) that the pathology report should include a statement on whether the penile carcinoma is associated with an HPV infection (Recommendation 4.8). The background information for this recommendation explicitly identifies p16 as a marker for HPV and notes that evidence of HPV or p16 positivity is associated with better disease-specific survival. This directly aligns with the proposed recommendation to conduct p16 testing to differentiate between HPV-associated and HPV-independent disease as part of the initial diagnostic workup. |
| EAU-ASCO2025 | R | The guideline strongly supports the treatment recommendation. Section 3.4.7 provides a "Strong" recommendation that "The pathological evaluation of penile carcinoma specimens must include an assessment of p16 by immunohistochemistry." This aligns perfectly with the proposed recommendation. Furthermore, Section 3.4.5 explains that p16 IHC is used as a surrogate marker to differentiate between HPV-associated and HPV-independent subtypes of penile squamous cell carcinoma, which is the exact purpose stated in the treatment recommendation. |
| ESMO2024 | R | The guideline recommends that the pathological assessment for penile cancer should include HPV status [III, A]. In the section on "HPV-related penile cancer," it explicitly states that HPV-associated SCC demonstrates p16 expression, thereby linking p16 testing to the determination of HPV status. This aligns directly with the recommendation to conduct p16 testing to differentiate between HPV-associated and HPV-independent disease as part of the initial diagnostic work-up. |
| NCCN2025 | R | The NCCN guideline supports the treatment recommendation. The "Primary Evaluation" algorithm (PN-1) explicitly includes "Assess human papillomavirus (HPV) status" as a key step in the initial workup for a suspicious penile lesion. Furthermore, the "Principles of Pathology" page (PN-F) outlines methods for identifying HPV status, specifically listing "Morphology/p16 immunohistochemistry/molecular methods." This directly aligns with the recommendation to use p16 testing via IHC to differentiate between HPV-associated and HPV-independent disease during the diagnostic process. |

**4.2 For cases where Penile Verrucous Carcinoma (PVC) is highly suspected, repeated deeper biopsies that include the basement membrane of the papillomatous tumor are recommended to avoid misdiagnosis.**

[14]

| AUA2020 | NA | The AUA Update Series guideline focuses on the treatment of early-stage squamous cell carcinoma of the penis. While its diagnosis section mentions that a "definitive diagnosis is achieved through tissue sampling/excision," it provides no specific details on biopsy techniques. The document does not mention Penile Verrucous Carcinoma (PVC) as a distinct entity, nor does it address the recommendation for repeated, deeper biopsies including the basement membrane to avoid misdiagnosis of this particular subtype. |
| --- | --- | --- |
| DGU2020 | R | The guideline aligns with the recommendation by emphasizing the necessity of an adequate biopsy for an accurate histopathological diagnosis. Recommendation 4.10 explicitly states that a sufficiently large amount of tissue must be obtained to guarantee a reliable diagnosis. The background text further elaborates that the size and depth of the biopsy must be sufficient to capture the invasive front of the tumor, which is precisely the goal of the recommended deeper biopsies for suspected verrucous carcinoma. |
| EAU-ASCO2025 | R | The guideline aligns with the treatment recommendation. Section 3.4.6, "Penile biopsy: pathological and technical considerations," emphasizes the importance of biopsy quality for accurate diagnosis. While a punch biopsy may suffice in most cases, the guideline explicitly states that when "assessment of depth of invasion is necessary, an incisional biopsy which is deep enough to properly assess the degree of invasion and stage is preferable." This directly supports the recommendation for deeper biopsies in suspected Penile Verrucous Carcinoma, where assessing the invasion pattern at the base is crucial to avoid misdiagnosis. |
| ESMO2024 | NA | The guideline recommends that "Incisional or excisional biopsies of suspected penile cancer should be carried out to confirm a histological diagnosis." While it also lists verrucous carcinoma as a specific histological subtype under the pathology section, it does not provide any specific instructions on the biopsy technique for this particular subtype. The recommendation to perform repeated, deeper biopsies that include the basement membrane specifically for suspected Penile Verrucous Carcinoma to avoid misdiagnosis is not addressed in the guideline. |
| NCCN2025 | NA | The NCCN guideline provides general recommendations for the histologic diagnosis of a suspicious penile lesion, suggesting a "Punch, excisional, or incisional biopsy" (PN-1). While the guideline acknowledges verrucous carcinoma as a specific subtype of squamous cell carcinoma with a good prognosis (PN-F), it does not offer distinct biopsy instructions for this particular histology. The specific recommendation to perform "repeated deeper biopsies that include the basement membrane" to avoid misdiagnosing Penile Verrucous Carcinoma is not mentioned. |

**4.3 Preoperative MRI is recommended for local staging of penile cancer, particularly to distinguish between T2 (spongiosum invasion) and T3 (cavernosa involvement) disease, to inform planning for organ-sparing surgery.**

[15]; [4]; [16]

| AUA2020 | R | The guideline supports the use of magnetic resonance imaging for local staging in the context of organ-preserving surgery. In the "DIAGNOSIS" section, the guideline states, "Magnetic resonance imaging with or without artificial erection is also used for local staging in cases of organ preserving surgery." This statement directly aligns with the treatment recommendation. |
| --- | --- | --- |
| DGU2020 | DR | The guideline explicitly states that the extent and infiltration depth of the primary tumor cannot be satisfactorily clarified with MRI (p. 38). It mentions that while MRI can be used as an alternative to assess infiltration depth, the examination is often painful for patients and is therefore "not generally recommended" (p. 38, "nicht grundsätzlich empfohlen"). This is a different approach from the treatment recommendation, which advises routine preoperative MRI for local staging to differentiate T2 and T3 disease. |
| EAU-ASCO2025 | R | The guideline recommends using MRI for local staging in specific scenarios that align with the treatment recommendation. In section 5.3, the guideline states to "Perform magnetic resonance imaging (MRI) ... when there is uncertainty regarding corporal invasion and/or the feasibility of (organ-sparing) surgery." This directly supports the recommendation's purpose of using MRI to distinguish T2 from T3 disease to inform planning for organ-sparing surgery. Section 5.1.2 further clarifies that while MRI doesn't outperform physical examination for T1 vs. T2 disease, it "can be helpful" when corporal invasion (cT3) is uncertain and organ-sparing options are being considered. |
| ESMO2024 | R | The guideline supports the use of MRI for the local staging of penile cancer. Under the "Assessment of the primary tumour" section, it states that "Where there is uncertainty, magnetic resonance imaging (MRI)...can be helpful to stage the primary lesion." This is reiterated in a formal recommendation which states, "The use of MRI or US combined with an intracavernosal injection of prostaglandin E1 is useful to assess the primary lesion [III, B]." This aligns with the recommendation's purpose of using MRI to inform planning for organ-sparing surgery. |
| NCCN2025 | R | The guideline supports the use of MRI for the local staging of penile cancer. The discussion section, under "Management of Primary Lesions" (MS-4), explicitly states that "MRI or ultrasound can be used to evaluate the depth of tumor invasion." This directly aligns with the recommendation's proposal to use MRI to differentiate between T2 (spongiosum) and T3 (cavernosa) invasion, which is a critical factor in planning for organ-sparing surgery versus more radical procedures. |

**4.4 Cross-sectional imaging (CT and MRI) is recommended as helpful for detecting metastases in the pelvis/retroperitoneum and in patients whose body habitus limits physical examination.**

[17]

| AUA2020 | R | The guideline supports the use of cross-sectional imaging for staging penile cancer. In the "Diagnosis" section, it states that "Imaging is often required to determine the extent of tumor invasion and inguinal lymph nodes," as physical examination alone can result in false negatives, "especially in obese patients." The guideline specifically mentions that "Computerized tomography of the chest, abdomen and pelvis is typically performed to evaluate the spread of cancer to the lymph nodes and more distant locations," which directly aligns with the recommendation. |
| --- | --- | --- |
| DGU2020 | R | The guideline aligns with the recommendation by stating that further cross-sectional imaging ("Schnittbilddiagnostik") is indicated for the diagnosis of pelvic and retroperitoneal lymph nodes, as well as distant metastases, in advanced stages (p. 42). It also specifies that cross-sectional imaging can be used for very obese ("adipösen") patients where clinical palpation is unreliable (p. 41). This directly supports both aspects of the treatment recommendation. |
| EAU-ASCO2025 | R | The guideline aligns with the recommendation by advising the use of cross-sectional imaging for staging in specific patient populations. In clinically node-positive (cN+) patients, the guideline strongly recommends using CT (or PET/CT) to stage the pelvis and exclude distant metastases. For clinically node-negative (cN0) patients, it states that while routine imaging is not recommended, modalities like CT and MRI "can be of value to detect enlarged/abnormal nodes in patients when physical examination is challenging (e.g., due to obesity)," which directly supports the recommendation's point about body habitus. |
| ESMO2024 | R | The guideline supports the use of cross-sectional imaging for staging. Under the "Assessment of distant metastases" section, it states that for routine staging, "CT of the chest, abdomen and pelvis is sufficient." The guideline further advises that "MRI or CT fusion PET probably has the highest sensitivity for detecting distant metastasis and can be used in high-risk cases," directly aligning with the recommendation's mention of both CT and MRI for detecting metastases in the pelvis. |
| NCCN2025 | R | The NCCN guideline supports the recommendation for using cross-sectional imaging. Under "Principles of Imaging" (PN-E), the initial workup includes "Abdomen/pelvis CT or MRI" and specifically "CT/MRI of pelvis for nodal evaluation if difficult to clinically assess." The discussion section further confirms that CT or MRI may be used to assess for pelvic and retroperitoneal lymph nodes (MS-9). The guideline also notes that during surveillance, imaging can be considered for patients affected by obesity, aligning with the recommendation's mention of body habitus limiting physical examination (PN-8, footnote x). |

**4.5 For biopsy-confirmed Penile Verrucous Carcinoma (PVC) with no clinically palpable inguinal lymph nodes, further workups like CT or ultrasonography can be postponed initially.**

[14]

| AUA2020 | DR | The guideline recommends a different diagnostic approach. It states that for early-stage penile cancer, "Imaging is often required to determine the extent of...inguinal lymph nodes," and that "Computerized tomography... is typically performed to evaluate the spread of cancer to the lymph nodes." The guideline explicitly warns that relying only on palpation can lead to "false-negative findings," directly contradicting the recommendation to postpone imaging based on a lack of palpable nodes. |
| --- | --- | --- |
| DGU2020 | R | The guideline aligns with the recommendation. Recommendation 7.29 explicitly states that clinical surveillance ("klinische Überwachung") is the appropriate management for patients with pTa tumors, which includes verrucous carcinoma. The background information on page 63 further supports this by noting that imaging techniques like sonography and CT are unreliable for detecting occult metastases in patients with clinically non-palpable inguinal lymph nodes. Therefore, postponing these specific workups in favor of clinical surveillance is consistent with the guideline's approach for this low-risk patient group. |
| EAU-ASCO2025 | R | The guideline aligns with the recommendation. It identifies verrucous carcinoma as a non-invasive (Ta), low-risk subtype with no reported metastasis (Table 3.1 and 4.1). For clinically node-negative (cN0) patients, the guideline explicitly states that imaging modalities like CT and ultrasonography are of limited value and are "not recommended for routine use" (Section 5.2.2.1). This directly supports the recommendation to postpone these workups in patients with biopsy-confirmed PVC and no palpable nodes. |
| ESMO2024 | DR | The treatment recommendation suggests that for biopsy-confirmed Penile Verrucous Carcinoma (PVC) with no palpable inguinal lymph nodes (cN0), further workups like CT or ultrasonography (US) can be postponed. However, the ESMO guideline recommends a different initial approach. The guideline's diagnostic algorithm explicitly states that all patients with clinically impalpable inguinal LNs (cN0) "should undergo US imaging and fine-needle aspiration cytology (FNAC) of morphologically abnormal inguinal nodes." This recommendation is presented as a standard step in the diagnostic work-up for all cN0 patients, without making a specific exception for verrucous carcinoma. |
| NCCN2025 | R | The guideline supports the recommendation. On page PN-3, footnote 'f' states that "Ta verrucous carcinoma is by definition a well-differentiated tumor. Therefore, only surveillance of the inguinal lymph nodes is required." The corresponding flowchart for low-risk disease (which includes Ta verrucous carcinoma) with non-palpable inguinal lymph nodes leads directly to "Surveillance (PN-8)," without an initial imaging step. This aligns with the recommendation to postpone further workups like CT or ultrasonography in this specific clinical scenario. |

**4.6 For patients with a high clinical suspicion of inguinal lymph node metastasis (ILNM), a comprehensive workup at the time of initial biopsy is suggested, including blood and tumor markers (e.g., CRP, NLR, PD-L1, p53, SCC-Ag) and advanced imaging.**

[17]

| AUA2020 | NA | The guideline addresses the diagnosis of penile cancer and mentions the need to evaluate inguinal lymph nodes through palpation and imaging, such as CT scans. However, the document does not mention or discuss a comprehensive workup that includes the specific panel of blood and tumor markers (e.g., CRP, NLR, PD-L1, p53, SCC-Ag) proposed in the recommendation. Since the guideline is silent on the use of these particular markers for assessing nodal metastasis, it does not address this specific recommendation. |
| --- | --- | --- |
| DGU2020 | DR | The guideline proposes a different diagnostic pathway for patients with high clinical suspicion of inguinal lymph node metastasis (i.e., palpable nodes). It recommends direct surgical removal for histological confirmation as the primary step (Recommendation 6.7). This contrasts with the recommendation's suggestion of an initial comprehensive workup with markers and imaging. Moreover, the guideline explicitly states that there are currently no molecular markers, including p53 and PD-L1, recommended for the routine diagnosis of penile carcinoma (Statement 4.9), and that tumor markers have no role in follow-up (p. 82). |
| EAU-ASCO2025 | DR | The guideline aligns with the recommendation for using advanced imaging in patients with clinically node-positive (cN+) disease, strongly recommending either 18FDG-PET/CT or CT of the chest and abdomen to stage the pelvis and exclude distant metastases. However, it recommends a different overall approach by omitting the use of blood and tumor markers. The guideline explicitly states in section 5.2.2.1 that "there are no validated nomograms or tumour markers that can reliably predict LN involvement," and it does not recommend CRP, NLR, PD-L1, p53, or SCC-Ag as part of the initial workup. Instead, it emphasizes histopathological confirmation through image-guided biopsy in addition to imaging. |
| ESMO2024 | DR | The guideline recommends a diagnostic workup for suspected inguinal lymph node metastasis based on clinical assessment (palpable vs. impalpable nodes), imaging (ultrasound, CT, MRI), and tissue sampling (FNAC or biopsy). While it discusses advanced imaging, it does not mention or recommend the use of the specific blood and tumor markers (e.g., CRP, NLR, PD-L1, p53, SCC-Ag) suggested in the recommendation for the initial diagnostic evaluation. Therefore, the guideline outlines a different diagnostic pathway that does not include these specific serological or tumor markers. |
| NCCN2025 | DR | The guideline recommends a comprehensive workup for patients with suspected or high risk for inguinal lymph node metastasis that includes advanced cross-sectional imaging (CT, MRI) and consideration of PET/CT (PN-E, MS-9). This aligns with the imaging component of the recommendation. However, the guideline does not mention or recommend the use of any of the suggested blood or tumor markers (e.g., CRP, NLR, PD-L1, p53, SCC-Ag) for the initial workup. The guideline's pathway for evaluation and risk stratification relies on history and physical exam, histologic diagnosis from biopsy, HPV status, and imaging, making its recommended approach different from the one proposed. |

**5. Diagnosis / Staging (Imaging Protocol)**

**5.1 When performing preoperative local staging MRI, institutions may choose to omit artificial erection (induced by intracavernosal injection) from their protocols, as it has similar accuracy to MRI without it.**

[15]

| AUA2020 | R | The guideline supports the recommendation. In the "DIAGNOSIS" section, the guideline states that "Magnetic resonance imaging with or without artificial erection is also used for local staging in cases of organ preserving surgery." This statement explicitly acknowledges that performing MRI without an artificial erection is an acceptable option for local staging, which aligns directly with the recommendation. |
| --- | --- | --- |
| DGU2020 | R | The treatment recommendation suggests that artificial erection may be omitted from preoperative local staging MRI protocols. The guideline aligns with this by stating that MRI with artificial erection is often painful for patients and, for this reason, is not generally recommended (page 38). While the guideline's primary reason is patient discomfort rather than a comparison of accuracy, it ultimately supports the same action of not performing this procedure. |
| EAU-ASCO2025 | R | The guideline directly supports the treatment recommendation. In the summary of evidence, it states, "Magnetic resonance imaging with and without artificial erection showed similar accuracy in local staging" (p. 18). Furthermore, the formal recommendations advise to "Perform magnetic resonance imaging (MRI) of the penis/primary tumour (artificial erection not mandatory)..." (p. 18). This explicit statement that artificial erection is not mandatory aligns perfectly with the recommendation that it may be omitted from MRI protocols due to similar accuracy. |
| ESMO2024 | DR | The guideline recommends a different approach for preoperative local staging. It states that where there is uncertainty, "magnetic resonance imaging (MRI) or penile ultrasound (US) combined with an intracavernosal injection of prostaglandin E1 to induce an artificial erection can be helpful to stage the primary lesion." This contradicts the treatment recommendation, which suggests that omitting the artificial erection from MRI protocols is acceptable due to similar accuracy. |
| NCCN2025 | NA | The NCCN guideline recommends MRI for the initial workup and staging of penile cancer, including for nodal evaluation and to assess the depth of tumor invasion. However, the guideline does not provide specific technical protocols for how the MRI should be performed. There is no mention of using or omitting artificial erection induced by intracavernosal injection, so the guideline does not address this specific aspect of the imaging procedure. |

**6. Follow-up Care**

**6.1 Close, strict follow-up is essential for all patients after treatment, particularly after penile-preserving surgery, local excision for PeIN, or a negative DSNB, due to the risk of recurrence.**

[18]; [14]; [4]; [19]

| AUA2020 | R | The guideline strongly supports the recommendation for close and strict patient follow-up. The text repeatedly emphasizes this point, stating, "close postoperative follow-up is crucial" after circumcision and that for penile-preserving techniques in general, "Close follow-up for at least 5 years is required to detect and re-treat any potential recurrences." This directly aligns with the recommendation's rationale of monitoring for recurrence after treatment. |
| --- | --- | --- |
| DGU2020 | R | The guideline strongly aligns with the recommendation for close patient follow-up. Recommendation 8.1 explicitly states that regular, risk-stratified follow-up should be performed for penile cancer patients to detect tumor recurrences. The guideline provides detailed follow-up schedules on page 82, recommending more frequent examinations for patients who underwent organ-preserving surgery compared to total penectomy. Additionally, Recommendation 7.9 mandates regular long-term follow-up after local therapies, which corresponds to the recommendation's point about local excision for PeIN. |
| EAU-ASCO2025 | R | The guideline strongly supports the recommendation for close and strict follow-up. Section 7.2, "Rationale for follow-up," explicitly states that surveillance is important for the early detection of recurrence to increase the likelihood of curative treatment. The guideline details specific risks for the mentioned patient groups, noting that "Local recurrence is more likely with all types of local organ-sparing treatment" (Section 7.2.2) and that a risk of regional recurrence exists even in patients with a negative DSNB (Section 7.2.3). Finally, Table 7.1 provides a detailed follow-up schedule, recommending 3-monthly visits for the first two years post-penile-preserving surgery, which aligns with the "close, strict" nature of the proposed recommendation. |
| ESMO2024 | R | The guideline strongly supports the recommendation for close follow-up after treatment. The "Follow-Up, Long-Term Implications And Survivorship" section explicitly states that "close clinical follow-up is required for the first 2 years after surgery" to detect recurrence. It specifically highlights the high recurrence rates (20%-50%) after penile-preserving surgery and the risk associated with a negative dynamic sentinel node biopsy (DSNB), mirroring the key points in the recommendation. |
| NCCN2025 | R | The NCCN guideline strongly supports the recommendation for close follow-up after treatment. Page PN-8 provides a detailed "SURVEILLANCE SCHEDULE" for all patients, dictating the frequency of clinical examinations based on the initial treatment. The guideline's discussion on page MS-16 explicitly states that "penile-sparing therapies carry a significantly higher risk of local recurrence...and thus require closer surveillance," directly aligning with the recommendation's emphasis. This is reflected in the more frequent follow-up schedule for "Topical or local therapy" compared to penectomy, underscoring the necessity of strict monitoring due to recurrence risk. |

**6.2 Following curative Penile-Preserving Surgery (PPS), surveillance for local recurrence should be conducted every 3 months for the first 2 years, and then every 6 months until at least 5 years post-operatively.**

[20]

| AUA2020 | R | The guideline aligns with the recommendation for close surveillance following penile-preserving surgery. Under the "SURGERY" section, the text states, "Close follow-up for at least 5 years is required to detect and re-treat any potential recurrences." Furthermore, the appendix provides a specific follow-up schedule for Nd:YAG laser therapy, recommending checks "every 3 months for the first 2 years, biannually thereafter," which is nearly identical to the frequency and duration outlined in the recommendation. |
| --- | --- | --- |
| DGU2020 | R | The guideline provides a specific table on page 82, "Empfohlenes Nachsorgeschema für den Primärtumor" (Recommended Follow-up Schedule for the Primary Tumor). For patients who underwent organ-preserving surgery ("Organerhaltung"), the recommended schedule is a clinical examination every 3 months for the first two years ("Jahr 1-2: Alle 3 Monate"). From the third to the fifth year, the recommended interval is every 6 months ("Jahr 3-5: Alle 6 Monate"), which directly aligns with the treatment recommendation. |
| EAU-ASCO2025 | R | The guideline fully supports the treatment recommendation. Table 7.1, titled "Follow-up regime for penile cancer," specifically addresses the follow-up for "Penile-preserving treatment." The table outlines an identical surveillance schedule: 3-monthly intervals for the first 1-2 years and 6-monthly for years 3-5, with a minimum duration of 5 years. |
| ESMO2024 | R | The guideline aligns with the proposed treatment recommendation. The guideline states that most recurrences happen within the first two years and explicitly recommends "Close follow-up every 3-4 months for the first 2 years following primary surgery is required to detect local recurrence [IV, A]." This directly supports the recommendation's initial surveillance schedule of every 3 months for the first 2 years. |
| NCCN2025 | R | The NCCN guideline directly supports the surveillance schedule outlined in the recommendation. On page PN-8, under "SURVEILLANCE SCHEDULE," the guideline specifies that for patients who received "Topical or local therapy" (which includes penile-preserving surgery), surveillance should consist of a clinical examination every 3 months for the first 2 years, followed by every 6 months for years 3-5. This schedule is identical to the one proposed in the treatment recommendation. |

**6.3 Men with penile cancers demonstrating a p53 mutant pattern should be considered for a more intensive follow-up strategy.**

[21]

| AUA2020 | NA | The guideline provides recommendations for follow-up based on clinical and pathological factors such as tumor stage, grade, pattern of invasion, and the type of treatment received. However, it does not mention or discuss the use of specific molecular markers, such as p53 mutation status, as a factor for risk stratification or for determining the intensity of a follow-up strategy. Therefore, the guideline does not address the recommendation. |
| --- | --- | --- |
| DGU2020 | DR | The treatment recommendation suggests using p53 mutation status to guide the intensity of patient follow-up. The guideline acknowledges that abnormal p53 expression is associated with a poorer prognosis (p. 32). However, it explicitly states that there are currently no molecular markers, including p53, recommended for routine diagnostics (Statement 4.9, p. 32). The follow-up schedules provided in the guideline are risk-stratified based on the initial therapy and pathological lymph node status, not on any molecular markers (p. 82). |
| EAU-ASCO2025 | NA | The guideline notes that strong p53 immunoreactivity is a reported predictor for pelvic lymph node involvement, thereby acknowledging its potential prognostic value. However, the document does not incorporate p53 status into its specific follow-up recommendations. The proposed follow-up schedules in Section 7.2 are stratified based on the type of primary treatment and pathological node status, not on molecular markers like p53. Therefore, the guideline does not address the recommendation to use p53 status to intensify follow-up strategies. |
| ESMO2024 | NA | The guideline details follow-up strategies based on time since primary surgery, pathological staging (e.g., pN status), and primary tumor histological subtype. The section on molecular profiling discusses markers like TMB, MSI-H, and dMMR in the context of immunotherapy for advanced disease, not for surveillance. The guideline does not mention p53 mutations or a "p53 mutant pattern" as a factor for determining the intensity of a follow-up strategy. |
| NCCN2025 | NA | The NCCN guideline provides a detailed surveillance schedule based on the initial treatment (e.g., topical therapy, penectomy) and the pathologic nodal stage (pN stage). The recommended follow-up frequency and imaging are determined by these clinical and pathologic factors. The guideline does not mention p53 mutations or any other specific molecular markers as criteria for risk-stratifying patients to determine the intensity of their follow-up strategy. |

**6.4 Multidisciplinary follow-up with a psychologist trained in sexual therapy is recommended to aid in the recovery of post-operative sexual function and address psychosocial needs.**

[22]; [23]

| AUA2020 | NA | The guideline provides a comprehensive overview of various treatment modalities for early-stage penile cancer, including topical agents, laser therapy, and surgical options. It mentions the importance of postoperative functional and sexual outcomes, noting that many patients maintain an active sexual life after organ-sparing procedures. However, the document does not address the recommendation for multidisciplinary follow-up involving a psychologist or sexual therapist to manage psychosocial needs or aid in the recovery of sexual function. |
| --- | --- | --- |
| DGU2020 | R | The guideline strongly supports the recommendation for psychological follow-up. Section 3.2, "Psychoonkologische Betreuung" (Psycho-oncological Care), recommends that psychosocial counseling and treatment should be available to all patients. Furthermore, Recommendation 8.5 explicitly states that sexual dysfunctions and limitations are common, significantly impact patients, and should be openly addressed in the treatment context. The guideline details the severe psychosocial and sexual consequences of treatment, making psychological support for these specific issues a core component of recommended care. |
| EAU-ASCO2025 | R | The guideline strongly supports the recommendation for multidisciplinary follow-up that includes psychological and sexual therapy. Section 7.3.1 explicitly states that "Access to psychological support, counselling and psychosexual therapy are critical components of a holistic and multi-disciplinary survivorship service." Furthermore, a formal recommendation in Section 7.5 advises delivering care through an extended multidisciplinary team that includes "psychologists, counsellors... and sex therapists" and to "offer psychological support and counselling services." This directly aligns with the proposed treatment recommendation. |
| ESMO2024 | R | The guideline recommends regular follow-up to provide psychological support and to address sexual and urinary dysfunction that may result from surgical interventions. This aligns with the treatment recommendation for follow-up with a psychologist to aid in the recovery of sexual function and address psychosocial needs. The guideline supports the principle of managing these specific side effects, even though it doesn't detail the exact type of specialist required. |
| NCCN2025 | NA | The NCCN guideline for penile cancer provides comprehensive recommendations for the diagnosis, treatment, and surveillance of the disease, focusing on oncological management through surgery, radiotherapy, and systemic therapies. While the discussion section acknowledges that certain surgical approaches can have a "negative impact on sexual function and quality of life" (MS-5), the guideline does not contain any specific recommendations for involving a psychologist or sexual therapist in post-operative care. The detailed surveillance schedule (PN-8) outlines clinical examinations and imaging but does not mention psychosocial assessments or referrals for sexual therapy. |

**7. Intraoperative Care**

**7.1 Principles to Reduce Postoperative Complications during ILND: • Tissue Handling: Use atraumatic handling of anatomic structures. • Hemostasis/Lymphostasis: Perform careful control of lymphatics and small vessels. • Incisions: Use incisions that follow natural skin creases. • Flap Creation: Create the skin flap underneath the Scarpa fascia. • Wound Closure: Use myocutaneous flaps if primary closure is associated with excess tension or after prior radiotherapy.**

[10]

| AUA2020 | NA | The guideline focuses on the treatment of the primary tumor in early-stage penile cancer, covering various modalities such as topical therapies, laser ablation, and organ-sparing surgeries like circumcision and glansectomy. While the guideline mentions that inguinal lymph node dissection is necessary for higher-grade tumors, it does not discuss the specific surgical techniques or principles for performing this procedure. The recommendation, which provides detailed guidance on how to perform an inguinal lymph node dissection (ILND) to reduce postoperative complications, is outside the scope of the provided guideline. |
| --- | --- | --- |
| DGU2020 | NA | The guideline acknowledges the high morbidity and complication rates associated with radical inguinal lymphadenectomy (RIL) on page 68. To avoid these complications, it recommends measures such as antibiotic prophylaxis, wound drainage, and the use of clips and ligatures to close lymphatic vessels. While the control of lymphatics is mentioned, the guideline does not address the other specific surgical principles detailed in the recommendation, such as incision placement, the creation of skin flaps underneath the Scarpa fascia, or the use of myocutaneous flaps for wound closure. |
| EAU-ASCO2025 | R | The guideline aligns with the principles outlined in the treatment recommendation for reducing postoperative complications during inguinal lymph node dissection (ILND). Table 6.2 on page 27 lists several strategies to reduce morbidity, including the use of a "Linear transverse incision," which corresponds to the recommendation for incisions following natural skin creases. The table also notes the positive impact of "Fascial sparing," which is related to the recommended flap creation plane. Additionally, the guideline mentions in section 6.1.3.1 (page 23) that "composite myocutaneous flaps... may be needed to cover the surgical defect" in advanced cases, supporting the recommendation for complex wound closure. |
| ESMO2024 | R | The guideline aligns with the recommendation's principles for reducing postoperative complications during inguinal lymph node dissection (ILND). The guideline acknowledges the high morbidity of the procedure and explicitly supports the use of myocutaneous flaps, stating that "myocutaneous flap reconstruction is an option to cover the groin defect" in cases of extensive skin involvement. Furthermore, it notes that a "fascial-sparing approach... appear[s] to reduce the wound complication rates," which is consistent with the recommendation's principles of careful tissue handling and flap creation. |
| NCCN2025 | NA | The NCCN guideline acknowledges that inguinal lymph node dissection (ILND) is associated with significant surgical morbidity and discusses strategies to minimize complications, such as postoperative drains, antibiotics, and the use of modified template lymphadenectomy (MS-11). However, the guideline does not mention the specific, granular intraoperative surgical techniques detailed in the recommendation. Principles such as atraumatic tissue handling, creating skin flaps underneath the Scarpa fascia, or placing incisions along natural skin creases are not addressed in the provided text. |

**8. Intraoperative Management**

**8.1 Intraoperative Frozen Section Examination (FSE) of surgical margins should be considered during surgery to confirm complete excision, which allows for more conservative macroscopic margins, maximal preservation of penile length, and a reduced risk of positive margins and local recurrence.**

[20]; [23]; [4]

| AUA2020 | R | The guideline supports the use of intraoperative frozen section examination (FSE) for margin control during surgery. In the "SURGERY" section, it explicitly states, "In experienced hands tumor excision with negative intraoperative frozen section margins can be performed safely in well selected patients." It also positions FSE as a peer technique to Mohs surgery, noting there is no evidence that Mohs is superior to "negative intraoperative frozen section margins" for oncologic outcomes, thereby endorsing its use. |
| --- | --- | --- |
| DGU2020 | R | The guideline aligns with the recommendation by stating that the goal of surgery is complete tumor removal with negative margins, which can be secured intraoperatively through frozen section examination (Section 4.4.6). It also supports the concept of more conservative surgery by noting that a safety margin of a few millimeters is now considered sufficient, a shift from the older 2 cm standard. The guideline cites evidence [174] showing that a 5 mm resection margin is adequate when confirmed by a negative intraoperative frozen section, which directly supports the recommendation's premise of using FSE to enable maximal preservation of penile length. |
| EAU-ASCO2025 | R | The guideline supports the use of intraoperative frozen section analysis, aligning with the recommendation. Section 6.1.2.2 acknowledges that the role of frozen section remains "uncertain" and that it is "not recommended to be used routinely" due to conflicting evidence. However, the Panel's opinion is that it is a "helpful tool," and the formal recommendation on page 25 advises to "Perform intra-operative frozen section analysis of resection margins in cases of doubt on the completeness of resection." This conditional support for using FSE in specific situations is consistent with the recommendation's suggestion that it "should be considered." |
| ESMO2024 | NA | The guideline recommends the use of intraoperative frozen section analysis specifically for assessing inguinal lymph nodes during a modified dissection to determine if a more radical procedure is needed. However, it does not address the recommendation's proposal of using intraoperative frozen section examination to assess the surgical margins of the primary penile tumor itself to ensure complete excision while maximizing tissue preservation. |
| NCCN2025 | R | The guideline recommends the use of intraoperative frozen sections to determine and achieve negative surgical margins. This is specifically mentioned for glansectomy (PN-A), penectomy (PN-B), and for lesions staged pT1b or T≥2 (MS-8). This aligns directly with the treatment recommendation to use intraoperative frozen section examination to confirm complete tumor excision. |

**8.2 Specific Biopsy Protocols during Penile-Preserving Surgery (PPS) with FSE: • During glansectomy: Biopsies of the complete circumference of the urethra and of each corporal tip should be sent for FSE. • During total glans resurfacing: Spongiosal biopsies of the deep margin should be taken for FSE.**

[20]

| AUA2020 | NA | The guideline supports glansectomy and glans resurfacing as surgical options for early-stage penile cancer and mentions the general practice of using "negative intraoperative frozen section margins." However, the guideline does not provide the specific, detailed surgical protocols outlined in the recommendation. There is no mention of biopsying the urethral circumference, corporal tips during glansectomy, or taking deep spongiosal biopsies during glans resurfacing. |
| --- | --- | --- |
| DGU2020 | R | The guideline supports the use of intraoperative frozen section examination to ensure complete tumor removal with negative margins (Section 4.4.6). It states that this method can be used to secure clear margins during surgery and highlights that areas along the penile urethra deserve special attention due to common patterns of tumor spread. While the guideline does not specify the exact biopsy protocols for glansectomy or glans resurfacing, the recommendation's detailed approach is a specific application of the general principle endorsed by the guideline. |
| EAU-ASCO2025 | R | The guideline supports the use of intra-operative frozen section examination (FSE) as a helpful tool to ensure complete tumor removal, though it does not recommend its routine use in all cases. In the context of resectable locally advanced disease (Section 6.1.3.1), it specifically states that "frozen sections of the corporeal tips and urethra may be helpful in assessing the radicality of the procedure peri-operatively." This directly aligns with the recommendation to send biopsies from the urethra and corporal tips for FSE during glansectomy to confirm clear margins. |
| ESMO2024 | NA | The guideline recommends penile-preserving surgeries like glansectomy and glans resurfacing for localized penile cancer and emphasizes the importance of achieving clear surgical margins. However, it does not specify the method for intraoperative margin assessment. The recommendation's detailed protocols for using frozen section examination (FSE) with specific biopsy sites during these procedures are not mentioned or discussed in the provided guideline. |
| NCCN2025 | R | The NCCN guideline supports the use of intraoperative frozen sections to ensure negative margins during penile-preserving surgeries. Specifically for glansectomy, the guideline states that "Negative surgical margins should be determined from frozen sections of the cavernosal bed and urethral stump" (PN-A, 2 of 2). This aligns with the recommendation to biopsy the corporal tips (cavernosal bed) and urethra. Although the guideline does not specify a protocol for glans resurfacing, its general recommendation for using intraoperative frozen sections to determine negative margins (PN-B) is consistent with the proposed approach. |

**8.3 Following glansectomy, deep separate tunical biopsies should be obtained from the corpora to ensure an accurate pathological assessment of the deep surgical margins.**

[4]

| AUA2020 | NA | The guideline extensively discusses glansectomy as a primary surgical treatment for early-stage penile cancer located on the glans and emphasizes achieving negative surgical margins. However, while it mentions the use of intraoperative frozen sections for margin assessment, it does not specifically address the technique of obtaining deep separate tunical biopsies from the corpora following the procedure to assess the deep surgical margin. This specific procedural detail is not mentioned in the provided text. |
| --- | --- | --- |
| DGU2020 | NA | The guideline extensively discusses glansectomy for T1/T2 tumors and emphasizes the critical importance of achieving negative surgical margins, suggesting intraoperative frozen section analysis for this purpose. However, it does not mention or address the specific technique of obtaining additional, separate deep tunical biopsies from the corpora cavernosa after the glans has been resected. The guideline's focus is on assessing the main resection margin, not on this particular additional biopsy method. |
| EAU-ASCO2025 | NA | The guideline addresses glansectomy for tumors confined to the glans and prepuce (Section 6.1.2.7). In cases where corporeal invasion is uncertain, it suggests that "frozen sections of the corporeal tips and urethra may be helpful in assessing the radicality of the procedure peri-operatively" (Section 6.1.3.1). However, the guideline does not specifically mention or recommend the practice of obtaining deep, separate tunical biopsies from the remaining corpora for pathological assessment after the glansectomy is complete. Since this specific technique for margin assessment is not discussed, the recommendation is considered not addressed. |
| ESMO2024 | NA | The guideline recommends glansectomy as a surgical treatment for T2 tumors and emphasizes the importance of achieving adequate surgical margins. However, it does not address the specific intraoperative technique of obtaining separate deep tunical biopsies from the corpora for pathological assessment of the deep surgical margin. The document discusses pre-operative assessment of corporal invasion but is silent on this particular post-resection step. |
| NCCN2025 | DR | The NCCN guideline recommends a different approach for assessing deep surgical margins after glansectomy. On page PN-A (2 of 2), under the "Glansectomy" section, the guideline specifies that "Negative surgical margins should be determined from frozen sections of the cavernosal bed and urethral stump." This method contrasts with the recommendation of obtaining "deep separate tunical biopsies from the corpora," as it details a different specimen (frozen sections from the bed and stump) for pathologic evaluation. |

**9. Lymph Node Management**

**9.1 For patients with low-risk penile cancer (e.g., Tis, Ta, T1a) and clinically non-palpable inguinal lymph nodes (cN0), surveillance/active monitoring is the recommended management strategy.**

[24]; [17]; [25]

| AUA2020 | DR | The recommendation proposes surveillance/active monitoring as the primary management for low-risk penile cancer (Tis, Ta, T1a). The guideline, however, advocates for active treatment for these same stages. It explicitly states that "Topical treatments, cryotherapy, circumcision, laser and photodynamic therapy are often used as first line treatment options for non-invasive and early stage penile malignancies (carcinoma in situ, Ta and T1)." The provided treatment algorithm also details various active interventions—such as topical therapy, laser therapy, and surgical excision—as the initial steps for early/low-stage disease, with no mention of surveillance as a primary management option. |
| --- | --- | --- |
| DGU2020 | R | The guideline directly supports the recommendation for surveillance in low-risk, node-negative patients. Recommendation 7.29 (page 62) explicitly states that clinical surveillance ("Eine klinische Überwachung") should be performed for patients with tumor stages pTa and pT1aG1, which corresponds to the low-risk group. Similarly, recommendation 6.6 (page 40) advises that for patients up to category pT1a without palpably enlarged lymph nodes (cN0), operative lymph node staging can be omitted in favor of regular clinical follow-up. This aligns perfectly with the proposed treatment recommendation. |
| EAU-ASCO2025 | R | The guideline aligns with the treatment recommendation. On page 17, under section 5.2.2.2, it defines low-risk tumors as "Well-differentiated (G1), pTa, pTis and pT1 tumours without lymphovascular/perineural invasion (pT1a)." For this exact patient group, it states, "the risk of metastases is too low to justify surgical staging." This directly supports a non-invasive management strategy like surveillance for low-risk, cN0 patients. |
| ESMO2024 | R | The guideline recommends clinical surveillance for patients with low-risk, clinically node-negative (cN0) penile cancer. This is explicitly stated in the "Recommendations" section for "cN0 disease," which advises, "Clinical surveillance of the inguinal nodes is recommended in patients presenting with cN0 tumours with low-risk features (pTa/pTis and pT1G1) [III, A]." Furthermore, the management algorithm for inguinal lymph nodes (Figure 3) clearly directs patients with impalpable nodes (cN0) and low-risk disease (Tis, Ta, T1G1) to surveillance, directly aligning with the proposed recommendation. |
| NCCN2025 | R | The NCCN guideline aligns directly with the treatment recommendation. The algorithm on page PN-3, "Management of Non-Palpable Inguinal Lymph Nodes," stratifies patients based on the primary lesion. For the "Low risk (PeIN, Ta, T1a)" group, which corresponds to the population in the recommendation, the guideline explicitly recommends "Surveillance." This is further supported by the discussion on page MS-15, which states that patients with low-risk disease are followed with a surveillance protocol due to the low probability of occult micrometastases. |

**9.2 For patients with intermediate or high-risk penile cancer (e.g., ≥pT1b/G2) and clinically non-palpable inguinal nodes (cN0), prophylactic surgical lymph node staging is recommended. Options include dynamic sentinel lymph node biopsy (DSNB) or inguinal lymph node dissection (ILND).**

[17]; [26]; [27]; [28]; [15]; [29]; [30]

| AUA2020 | NA | The AUA guideline focuses on the local treatment of the primary tumor in early-stage penile cancer, detailing options such as topical therapies, laser ablation, and organ-sparing surgery. While the document briefly mentions that patients with grade 2 and 3 tumors will require inguinal node dissection, it does not provide a comprehensive discussion on the specific strategy of prophylactic surgical lymph node staging for clinically node-negative (cN0) patients. Key elements of the recommendation, such as specific risk criteria (e.g., ≥pT1b) and dynamic sentinel lymph node biopsy (DSNB) as a staging option, are not mentioned, making the recommendation unaddressed by this guideline. |
| --- | --- | --- |
| DGU2020 | R | The guideline strongly recommends invasive investigation of clinically non-palpable inguinal lymph nodes for patients with penile cancer from category pT1b onwards (Recommendation 6.5, page 40). This aligns perfectly with the patient group (intermediate or high-risk, ≥pT1b/G2) and the action (prophylactic surgical lymph node staging) described in the treatment recommendation. Furthermore, the guideline explicitly names modified inguinal lymphadenectomy and dynamic sentinel lymph node biopsy as the two procedural options, matching the methods proposed in the recommendation. |
| EAU-ASCO2025 | R | The guideline aligns with the treatment recommendation. On page 17, section 5.2.2.2, it identifies tumors that are stage T1b or higher as "high-risk" and recommends surgical staging for these patients. The recommendations table on page 18 reinforces this with a "Strong" recommendation to offer surgical LN staging to all cN0 patients at high risk (T1b or higher). Furthermore, the guideline explicitly names dynamic sentinel lymph node biopsy (DSNB) as the preferred surgical staging option, with inguinal lymph node dissection (ILND) as the alternative, which mirrors the options provided in the recommendation. |
| ESMO2024 | R | The guideline aligns with the treatment recommendation. In the section "Management of cN0 disease," the guideline states that "active treatment is recommended for patients with intermediate- and high-risk disease." Furthermore, under the formal "Recommendations" for "cN0 disease," it specifies that "For patients with cN0 intermediate or high-risk disease (pT1G2, pT1-4G3/G4 or lymphovascular invasion), DSLNB should be carried out," directly matching the proposed action and patient population. |
| NCCN2025 | R | The NCCN guideline directly supports the treatment recommendation for managing clinically non-palpable inguinal nodes in patients with intermediate or high-risk penile cancer. The flowchart on page PN-3, "Management of Non-Palpable Inguinal Lymph Nodes," specifies that for patients with "Intermediate/High risk" disease (T1b or any T2 or greater), the recommended treatment is either "Bilateral inguinal lymph node dissection (ILND)" or "Bilateral dynamic sentinel node biopsy (DSNB)." This aligns perfectly with the proposed recommendation. |

**9.3 For patients with clinically node-negative (cN0) penile cancer requiring surgical staging (indicated for intermediate- or high-risk tumors), Dynamic Sentinel Node Biopsy (DSNB) is the favored procedure, especially when performed in high-volume, experienced centers.**

[31]; [19]; [30]

| AUA2020 | NA | The provided AUA guideline focuses on the treatment of the primary, early-stage penile tumor, covering topical therapies, laser ablation, and various organ-preserving surgical techniques like circumcision and glansectomy. While the document acknowledges that inguinal lymph nodes must be evaluated and that higher-grade tumors require "inguinal node dissection," it does not mention or discuss any specific surgical staging procedures. The recommendation for Dynamic Sentinel Node Biopsy (DSNB) as a favored procedure is therefore not addressed in this guideline. |
| --- | --- | --- |
| DGU2020 | R | The guideline recommends invasive lymph node investigation for patients with clinically node-negative (cN0) penile cancer and tumors from category pT1b onwards. It explicitly states that this can be performed using either a modified inguinal lymphadenectomy (MIL) or a dynamic sentinel lymph node biopsy (DSNB) (Recommendation 6.5, p. 40). By presenting DSNB as a primary option for surgical staging in this patient group, the guideline supports the treatment recommendation. Furthermore, the guideline's background text emphasizes that DSNB requires an experienced, interdisciplinary team, which aligns with the recommendation's condition of performing the procedure in high-volume centers (p. 64). |
| EAU-ASCO2025 | R | The guideline directly supports the treatment recommendation. In the recommendations table for clinically node-negative (cN0) patients (page 18), the guideline provides a "Strong" recommendation to "offer dynamic sentinel node biopsy (DSNB)" when surgical staging is indicated. The text further elaborates in section 5.2.2.3 that DSNB was developed to minimize the morbidity of surgical staging, is a reliable technique with high diagnostic accuracy, and has better outcomes when performed in experienced centers, all of which aligns perfectly with the proposed recommendation. |
| ESMO2024 | R | The guideline directly supports the recommendation for using Dynamic Sentinel Node Biopsy (DSNB) in patients with clinically node-negative (cN0) intermediate- or high-risk penile cancer. The "Management of cN0 disease" section states that active treatment is recommended for these patients, in contrast to surveillance for low-risk cases. The guideline explicitly says, "DSLNB is recommended in all patients with intermediate- or high-risk cN0 disease" (p. 6) and repeats this in its final summary of recommendations for cN0 disease (p. 9). |
| NCCN2025 | R | The guideline recommends Dynamic Sentinel Node Biopsy (DSNB) as a valid treatment option for patients with non-palpable inguinal lymph nodes and intermediate/high-risk primary tumors. The treatment algorithm on page PN-3 (page 8) explicitly lists "Bilateral dynamic sentinel node biopsy (DSNB)" as an alternative to inguinal lymph node dissection. This is further qualified by footnote 'j' and the discussion on page MS-11 (page 40), which state that DSNB is recommended provided it is performed by a physician or center with experience, aligning perfectly with the recommendation's conditions. |

**9.4 For patients with clinically palpable, suspicious, or confirmed metastatic inguinal lymph nodes, radical inguinal lymph node dissection (ILND) is the standard of care.**

[10]; [1]; [27]; [17]; [32]; [28]; [29]

| AUA2020 | NA | The AUA Update Series guideline focuses specifically on the "Treatment of Early Stage Squamous Cell Carcinoma of the Penis," detailing various organ-preserving approaches like topical therapies, laser ablation, and local excisions for the primary tumor. The treatment recommendation, however, pertains to a more advanced stage of disease involving clinically palpable or confirmed metastatic inguinal lymph nodes. While the guideline mentions the necessity of evaluating inguinal lymph nodes for lymphadenopathy, it does not provide detailed recommendations or management strategies for treating confirmed nodal metastases, as this falls outside its stated scope of early-stage disease. |
| --- | --- | --- |
| DGU2020 | R | The guideline aligns with the treatment recommendation. It states that for palpably enlarged inguinal lymph nodes (tastbare inguinale Leistenknoten), metastasis should be histologically confirmed. Upon confirmation, the guideline recommends performing a radical inguinal lymphadenectomy. Specifically, recommendation 7.35 on page 67 states that radical inguinal lymphadenectomy should be performed in patients with histologically or cytologically confirmed lymph node metastases, which directly supports the proposed treatment. |
| EAU-ASCO2025 | DR | The guideline aligns with the recommendation that radical inguinal lymph node dissection (ILND) is the standard of care for patients with cN1-N2 disease. However, for a subset of the patients described in the recommendation—those with more advanced, fixed inguinal masses or pelvic lymphadenopathy (cN3 disease)—the guideline suggests a different approach. It recommends neoadjuvant chemotherapy (NAC) in preference to upfront surgery, explicitly discouraging initial surgical treatment for this group. This nuanced, stage-dependent strategy differs from the blanket recommendation for radical ILND for all clinically node-positive patients. |
| ESMO2024 | R | The guideline recommends a diagnostic workup for palpable inguinal lymph nodes (cN1-2 disease), including FNAC or biopsy, to confirm metastasis. If metastatic disease is confirmed, the guideline explicitly states that radical inguinal lymphadenectomy is the recommended procedure. This is further reinforced in the final recommendations section, which states, "In patients with cN1-2 disease, radical inguinal lymphadenectomy is recommended..." [III, A], aligning directly with the proposed treatment. |
| NCCN2025 | DR | The guideline does not recommend immediate radical inguinal lymph node dissection (ILND) for all patients with clinically palpable or suspicious nodes. The guideline explicitly states that "Palpable lymphadenopathy at the time of diagnosis does not warrant an immediate ILND" (MS-11) because many cases are inflammatory. Instead, the recommended first step is a percutaneous lymph node biopsy to confirm metastasis (PN-4, PN-5). Only after a positive biopsy is ILND considered, and it is often preceded by neoadjuvant chemotherapy, particularly for bulky disease (PN-5, PN-6). |

**9.5 Early lymphadenectomy is recommended as it improves survival. Delaying ILND, particularly beyond 3 months in high-risk patients, should be avoided.**

[1]; [33]

| AUA2020 | NA | The AUA guideline focuses on the treatment of the primary, early-stage penile tumor, covering topical therapies, laser ablation, and various organ-preserving surgical techniques. Although the document mentions that inguinal lymph nodes must be evaluated and that higher-grade tumors will require inguinal node dissection, it does not provide any specific recommendations regarding the timing of this procedure. The guideline does not discuss the benefits of early versus delayed lymphadenectomy or the survival implications of delaying the intervention, which is the central point of the treatment recommendation. |
| --- | --- | --- |
| DGU2020 | R | The guideline strongly supports the recommendation for early lymphadenectomy in at-risk patients. It explicitly states that early inguinal lymphadenectomy is prognostically clearly superior to a lymphadenectomy performed only after metastases become apparent (p. 66). Furthermore, the guideline highlights studies showing a significant drop in long-term survival (from ~90% to <40%) when inguinal lymph node metastases are removed later in the clinically evident stage versus the occult stage, reinforcing the recommendation to avoid delays (p. 63). |
| EAU-ASCO2025 | R | The guideline strongly supports the recommendation for early lymphadenectomy. Section 5.2 (page 16) highlights a non-randomized trial where early lymph node surgery resulted in a significantly higher three-year survival rate compared to delayed surgery (84% vs. 35%). Furthermore, Section 6.2.2.3.2 (page 27) reinforces the importance of timely intervention, and the recommendations table on page 28 explicitly advises to "Complete surgical inguinal and pelvic nodal management within three months of diagnosis," directly aligning with avoiding delays as suggested in the recommendation. |
| ESMO2024 | R | The guideline supports the recommendation for early lymphadenectomy in appropriate patient populations to improve survival. It specifically states that for patients with intermediate- and high-risk clinically node-negative (cN0) disease, "active treatment is recommended," which aligns with the recommendation to avoid delaying intervention in high-risk patients. For patients with palpable nodes (cN1-2), the guideline also recommends radical inguinal lymphadenectomy after confirmation. While the guideline advocates for clinical surveillance in low-risk patients, its overall stance for high-risk and node-positive disease is consistent with the principle of early intervention to improve outcomes. |
| NCCN2025 | R | The NCCN guideline strongly supports the recommendation for early lymphadenectomy. In the "Delayed Inguinal Lymphadenectomy" discussion, it explicitly states that patients undergoing "immediate ILND have better survival outcomes than patients undergoing delayed ILND" (MS-12). The guideline further recommends that "in most circumstances, patients with high-risk penile tumors should undergo immediate ILND" (MS-12). This aligns directly with the recommendation's emphasis on early intervention and avoiding delays to improve survival. |

**9.6 Surveillance or delayed ILND for patients with clinically suspected positive nodes (cN+) is not recommended as it risks missing a curative opportunity.**

[1]

| AUA2020 | NA | The guideline, "Treatment of Early Stage Squamous Cell Carcinoma of the Penis," focuses on the management of the primary tumor in early-stage (non-invasive and T1) disease. It discusses local therapies such as topical agents, laser ablation, and various surgical excisions. The recommendation, however, addresses the management of patients with clinically positive lymph nodes (cN+), which constitutes a more advanced disease stage. While the guideline states that inguinal lymph nodes must be evaluated, it does not provide any treatment recommendations for node-positive patients, and therefore does not address the specific scenario of surveillance versus immediate intervention in this population. |
| --- | --- | --- |
| DGU2020 | R | The guideline supports the treatment recommendation by advising immediate surgical intervention for patients with clinically positive (palpable) inguinal lymph nodes (cN+). Recommendation 6.7 states that palpably enlarged lymph nodes should be histologically confirmed by surgical removal, and if metastasis is proven, a radical inguinal lymphadenectomy should follow. The background text for Recommendation 7.35 further clarifies that for patients with palpable nodes, histological confirmation is performed first, followed by a radical inguinal lymphadenectomy if metastases are found. This approach directly opposes surveillance or delayed ILND, aligning with the recommendation to act promptly to preserve a curative opportunity. |
| EAU-ASCO2025 | R | The guideline aligns with the recommendation by advising against delayed treatment for patients with clinically positive nodes (cN+). It explicitly states that radical inguinal lymph node dissection (rILND) is the standard of care for cN1-2 disease. Furthermore, the guideline highlights evidence that a delay in nodal management of more than three to six months may negatively affect disease-free survival and recommends completing surgical management within three months of diagnosis. |
| ESMO2024 | R | The guideline recommends immediate and active management for patients with clinically positive lymph nodes (cN+), which aligns with the recommendation against surveillance or delay. The guideline specifies that for cN1-2 disease, a diagnosis should be confirmed via biopsy, followed by radical inguinal lymphadenectomy (ILND) if metastatic disease is found. This direct approach to treatment contrasts with surveillance, thereby supporting the recommendation's stance that delaying treatment risks missing a curative opportunity. |
| NCCN2025 | R | The NCCN guideline aligns with the recommendation to avoid surveillance or delayed inguinal lymph node dissection (ILND) for patients with clinically positive nodes (cN+). The guideline's algorithms for palpable inguinal lymph nodes (PN-4, PN-5) consistently recommend an immediate workup, starting with a percutaneous lymph node biopsy. Surveillance is only considered as an option if a biopsy is negative; if it is positive, active treatment with ILND (with or without neoadjuvant therapy) is advised. The discussion section further supports this by stating that data suggests immediate ILND leads to better survival outcomes than delayed ILND once groins become clinically positive, reinforcing the risk of missing a curative opportunity by waiting. |

**9.7 In patients diagnosed with metastatic inguinal nodes on histology following a DSNB, offering a completion radical inguinal lymph node dissection (RLND) is the standard treatment approach.**

[19]

| AUA2020 | NA | The guideline's scope, as stated in its title and learning objective, is explicitly limited to the "Treatment of Early Stage Squamous Cell Carcinoma of the Penis." The document discusses evaluating inguinal lymph nodes but does not provide management strategies for confirmed metastatic disease. The treatment recommendation addresses a more advanced scenario—managing histologically confirmed metastatic inguinal nodes—which is outside the scope of this early-stage focused guideline. |
| --- | --- | --- |
| DGU2020 | R | The guideline recommends a radical inguinal lymphadenectomy for patients with histologically or cytologically proven lymph node metastases (Recommendation 7.35). The treatment recommendation specifies this exact scenario, where a dynamic sentinel node biopsy (DSNB) provides the histological proof of metastasis. Therefore, the guideline's general principle directly supports the specific action of performing a completion radical inguinal lymph node dissection as the standard treatment in this situation. |
| EAU-ASCO2025 | R | The guideline directly supports the treatment recommendation. In section 5.2.2.3, "Surgical staging options," the text explicitly states, "In case histopathology identifies SN (micro)metastasis, ipsilateral completion ILND is indicated (see Section 6.2.2) [119]." This aligns perfectly with the recommendation to perform a completion radical inguinal lymph node dissection (RLND/ILND) for patients found to have metastatic inguinal nodes after a DSNB. |
| ESMO2024 | R | The guideline recommends dynamic sentinel lymph node biopsy (DSLNB) for patients with intermediate- or high-risk cN0 disease. It explicitly states that this should be "followed by radical inguinal lymphadenectomy where there is micrometastatic disease detected in the sentinel node" (p. 6). This approach is also visually represented in the management algorithm (Figure 3) and listed in the formal recommendations (p. 9), aligning perfectly with the proposed treatment. |
| NCCN2025 | R | The guideline aligns with the treatment recommendation. In the algorithm for "Management of Non-Palpable Inguinal Lymph Nodes" (PN-3), Dynamic Sentinel Node Biopsy (DSNB) is presented as a treatment option for intermediate/high-risk patients. Footnote 'k' on this page, as well as the "Principles of Surgery" on page PN-B, explicitly state: "If positive lymph nodes are found on DSNB, ILND is recommended." This directly supports the recommendation to perform a completion inguinal lymph node dissection (ILND) after a positive DSNB. |

**9.8 For very selected groups of patients with a positive dynamic sentinel lymph node biopsy (DSNB), strict surveillance might be considered as a potential alternative strategy to immediate completion RLND, but better stratification methods are needed.**

[19]

| AUA2020 | NA | The guideline focuses on the treatment of the primary, early-stage penile tumor, covering options like topical therapies, laser ablation, and various organ-sparing surgical techniques. While it mentions the importance of evaluating inguinal lymph nodes for staging, it does not address the specific management of patients who have already undergone a positive dynamic sentinel lymph node biopsy (DSNB). Therefore, the recommendation to consider surveillance versus immediate completion lymph node dissection in this specific patient population is not discussed in the provided text. |
| --- | --- | --- |
| DGU2020 | DR | The guideline recommends a different treatment approach. Recommendation 7.35 on page 67 explicitly states that a radical inguinal lymphadenectomy should be performed in patients with histologically proven lymph node metastases. A positive dynamic sentinel lymph node biopsy (DSNB) provides this histological proof. The proposed recommendation to consider strict surveillance as an alternative to immediate completion lymphadenectomy is not mentioned and directly contradicts the guideline's directive for definitive surgical intervention upon detection of nodal metastases. |
| EAU-ASCO2025 | DR | The guideline recommends a different treatment approach than the one proposed. The guideline explicitly states that for patients with a positive sentinel node (SN) biopsy, completion inguinal lymph node dissection (ILND) is the indicated treatment. This directly contradicts the recommendation to consider surveillance as an alternative strategy. Specifically, on page 17, the guideline states, "In case histopathology identifies SN (micro)metastasis, ipsilateral completion ILND is indicated." This position is reinforced on page 26, where it notes that "Radical ILND remains the standard of care for... cN0 patients with a tumour positive SN at DSNB." Therefore, the guideline recommends definitive surgical treatment, not surveillance, following a positive DSNB. |
| ESMO2024 | DR | The guideline recommends a different treatment approach. For patients with intermediate- or high-risk cN0 disease and metastatic nodes detected by a dynamic sentinel lymph node biopsy (DSLNB), the guideline explicitly recommends proceeding to a radical inguinal lymphadenectomy. This is in direct contrast to the recommendation, which suggests that strict surveillance could be an alternative to immediate radical surgery for selected patients with a positive DSLNB. The guideline's algorithm (Figure 3) and text on page 6 clearly state that a positive DSLNB should be followed by radical surgery, not surveillance. |
| NCCN2025 | DR | The treatment recommendation suggests that for select patients with a positive dynamic sentinel lymph node biopsy (DSNB), strict surveillance might be a potential alternative to immediate completion inguinal lymph node dissection (ILND). In contrast, the NCCN guideline explicitly recommends a different approach. The guideline's algorithm on page PN-3 and the associated footnote 'k' state, "If positive lymph nodes are found on DSNB, ILND is recommended." This is also directly stated under the Principles of Surgery on page PN-B, with no mention of surveillance as an alternative in this scenario. |

**9.9 Pelvic Lymph Node Dissection (PLND) should be performed after ILND for patients with pathological stage pN2 or predictors for pelvic lymph node involvement, such as three or more positive inguinal nodes or extranodal extension (ENE).**

[1]; [34]

| AUA2020 | NA | The AUA guideline focuses exclusively on the treatment of "Early Stage Squamous Cell Carcinoma of the Penis," detailing management options for the primary tumor such as topical therapies, laser ablation, and organ-sparing surgeries. While it emphasizes the importance of evaluating inguinal lymph nodes for staging, it does not provide any recommendations for managing metastatic nodal disease. Consequently, the specific indications for performing a Pelvic Lymph Node Dissection (PLND) in patients with pN2 disease or other high-risk features are outside the scope of this document and are not addressed. |
| --- | --- | --- |
| DGU2020 | R | The guideline aligns with the treatment recommendation by indicating pelvic lymph node dissection (PLND) based on the findings from the inguinal lymphadenectomy. Recommendation 7.36 on page 69 explicitly states that ipsilateral PLND ("pelvine Lymphknotenentfernung") should be performed for patients with two or more affected inguinal lymph nodes or with capsule-penetrating metastases ("kapselüberschreitenden Lymphknotenmetastasen"). This corresponds directly to the recommendation's criteria of pathological stage pN2 and extranodal extension (ENE). The background information further confirms that the indication for PLND is based on the extent of inguinal metastasis. |
| EAU-ASCO2025 | R | The guideline aligns with the treatment recommendation. Section 6.2.3.1.1 identifies "the number of positive inguinal LNs (1–2 vs. 3, or more...)" and "the presence of extracapsular spread" (extranodal extension) as significant predictors for pelvic lymph node metastasis. Based on this evidence, the recommendations table on page 30 advises to "Offer... prophylactic ipsilateral pelvic lymphadenectomy to patients if: three or more inguinal nodes are involved... [or] extranodal extension is reported...". This directly mirrors the criteria and action proposed in the recommendation. |
| ESMO2024 | R | The guideline recommends unilateral pelvic lymphadenectomy (PLND) for patients with high-risk features found after inguinal lymph node dissection. The specific indications listed in the guideline are "two or more ipsilateral inguinal metastases, metastasis with a diameter of ≥30 mm or extranodal extension." This strongly aligns with the treatment recommendation, which advises PLND for patients with extranodal extension or multiple positive nodes (the guideline specifies two or more, while the recommendation states three or more). Both sources agree that these pathological findings warrant PLND. |
| NCCN2025 | R | The NCCN guideline aligns with the treatment recommendation. The "Principles of Surgery" page (PN-B) states that "PLND should be considered at the time or following ILND in patients with ≥2 positive inguinal nodes on the ipsilateral ILND site or in the presence of extranodal extension on final pathologic review." Furthermore, the discussion on Pelvic Lymphadenectomy (MS-12) explicitly recommends PLND "in patients with three or more positive ILNs," directly matching the criteria in the recommendation. The treatment algorithms also consistently direct patients with pN2-3 disease toward PLND (PN-9). |

**9.10 Prophylactic inguinal lymphadenectomy is not an appropriate treatment for patients with Penile Verrucous Carcinoma (PVC) and non-palpable lymph nodes.**

[14]

| AUA2020 | NA | The guideline provides a comprehensive overview of the treatment for early-stage squamous cell carcinoma of the penis, focusing on options based on tumor stage (Tis, Ta, T1) and grade. However, it does not discuss specific histopathological subtypes, such as Penile Verrucous Carcinoma (PVC). Therefore, the document does not address the specific recommendation regarding the role of prophylactic inguinal lymphadenectomy for patients with PVC and non-palpable lymph nodes. |
| --- | --- | --- |
| DGU2020 | R | The treatment recommendation advises against prophylactic inguinal lymphadenectomy for patients with Penile Verrucous Carcinoma (PVC) and non-palpable lymph nodes. The guideline aligns with this by recommending clinical surveillance ("klinische Überwachung") specifically for patients with pTa tumors (Recommendation 7.29, page 62). The background information on page 63 further clarifies that patients with verrucous carcinoma fall into this pTa category, thus supporting surveillance over prophylactic surgery. |
| EAU-ASCO2025 | R | The guideline supports the recommendation. Table 3.1 on page 7 describes verrucous carcinoma as a subtype of penile cancer for which there is "No metastasis reported." Furthermore, Section 5.2.2.2 on page 17 states that for low-risk tumors, which include Ta tumors like verrucous carcinoma, "the risk of metastases is too low to justify surgical staging." This directly aligns with the recommendation to avoid prophylactic inguinal lymphadenectomy in patients with Penile Verrucous Carcinoma (PVC) and non-palpable lymph nodes. |
| ESMO2024 | R | The guideline supports the treatment recommendation. It characterizes verrucous carcinoma as a low-grade subtype of penile cancer. For patients with clinically non-palpable lymph nodes (cN0) and low-risk disease features (e.g., pT1G1), the guideline explicitly recommends "clinical surveillance" rather than immediate surgery. This aligns with avoiding prophylactic inguinal lymphadenectomy, as the guideline notes that performing this procedure on all cN0 patients would be considered "overtreatment." |
| NCCN2025 | R | The guideline aligns with the treatment recommendation. On page PN-3, under "MANAGEMENT OF NON-PALPABLE INGUINAL LYMPH NODES," patients with low-risk lesions, including Ta, are directed to surveillance. Footnote 'f' specifically clarifies, "Ta verrucous carcinoma is by definition a well-differentiated tumor. Therefore, only surveillance of the inguinal lymph nodes is required." This supports the recommendation against performing prophylactic inguinal lymphadenectomy for this patient population. |

**9.11 For patients with Penile Verrucous Carcinoma (PVC) presenting with lymphadenopathy, treatment with anti-inflammatory drugs may be the treatment of choice, followed by a lymph node biopsy as needed.**

[14]

| AUA2020 | NA | The AUA Update Series guideline does not address this specific recommendation. The document provides a general overview for treating "early stage squamous cell carcinoma" but never mentions the specific "verrucous carcinoma" (PVC) subtype. Consequently, it does not discuss the unique management strategy of using anti-inflammatory drugs for associated lymphadenopathy, instead focusing on diagnostic evaluation of lymph nodes and primary treatments like surgery, laser therapy, and topical agents. |
| --- | --- | --- |
| DGU2020 | DR | The guideline recommends a different approach for managing lymphadenopathy in penile cancer patients. While the recommendation suggests a trial of anti-inflammatory drugs, the guideline explicitly states that palpably enlarged inguinal lymph nodes should be histologically confirmed via operative removal (Empfehlung 6.7, p. 42). Furthermore, the guideline warns against delaying diagnosis, noting that the formerly recommended week-long antibiotic therapy for inguinal lymph node swelling is no longer acceptable (p. 37). The guideline's strategy is to proceed directly to histological confirmation rather than attempting a course of medication first. |
| EAU-ASCO2025 | DR | The guideline suggests a different approach for managing lymphadenopathy than the one proposed in the recommendation. The recommendation is to first treat with anti-inflammatory drugs and then biopsy as needed. However, the guideline explicitly states that for clinically node-positive (cN+) patients, the standard procedure is to "obtain (image-guided) biopsy to confirm nodal metastasis before initiating treatment" (Page 18). Furthermore, it cautions against using treatments like antibiotics to resolve enlarged nodes as this may "delay further staging and treatment and is not recommended" (Page 16, Section 5.2.1), a principle that contradicts the recommendation's "treat first, biopsy later" approach. |
| ESMO2024 | DR | The recommendation suggests an initial trial of anti-inflammatory drugs for lymphadenopathy, particularly in Penile Verrucous Carcinoma (PVC). The guideline, however, proposes a different course of action. While it acknowledges that lymphadenopathy can be secondary to inflammatory changes, it recommends immediate diagnostic confirmation with percutaneous fine-needle aspiration cytology (FNAC) or biopsy for any palpable inguinal lymph nodes due to the high probability (>80%) of metastatic disease. The guideline's recommended pathway does not include a trial of anti-inflammatory medication prior to biopsy. |
| NCCN2025 | DR | The treatment recommendation suggests using anti-inflammatory drugs as the initial treatment for lymphadenopathy in patients with Penile Verrucous Carcinoma (PVC). In contrast, the NCCN guideline recommends a different approach for palpable inguinal lymph nodes. The guideline's favored initial step is a percutaneous lymph node biopsy to differentiate between inflammatory swelling and metastatic disease. While the guideline acknowledges that lymphadenopathy can be inflammatory and mentions a traditional (but less favored) use of antibiotics, it does not recommend a trial of anti-inflammatory drugs, instead prioritizing a biopsy for definitive diagnosis (MS-11, PN-4). |

**9.12 For low-stage (T1c N0 or below), clinically node-negative patients, guidelines do not recommend the removal of lymph nodes.**

[24]

| AUA2020 | DR | The guideline offers a different recommendation that is more nuanced than the one provided. While the guideline focuses on conservative management of the primary tumor in early-stage disease, it explicitly states that patients with moderately or poorly differentiated tumors (grades 2 and 3) "will require inguinal node dissection." This directly contradicts the broader recommendation that guidelines do not advise lymph node removal for all low-stage, clinically node-negative patients, as the guideline makes this decision contingent on tumor grade. |
| --- | --- | --- |
| DGU2020 | DR | The guideline offers a more nuanced, risk-stratified approach compared to the treatment recommendation. While it agrees that lymph node removal is not necessary for the lowest-risk stages (pTa and pT1aG1), recommending clinical surveillance instead (Recommendation 7.29), it explicitly advises invasive lymph node investigation for higher-risk T1 stages (pT1b and above) even in clinically node-negative (cN0) patients due to the high risk of occult micrometastases (Recommendation 6.5). This contradicts the broader suggestion to avoid lymph node removal for all low-stage T1 patients. |
| EAU-ASCO2025 | DR | The guideline presents a different and more nuanced approach than the recommendation. While it states that for low-risk tumors (pT1a, well-differentiated) the "risk of metastases is too low to justify surgical staging" (page 17), it explicitly defines a high-risk category even within the T1 stage. The guideline strongly recommends surgical lymph node staging for all clinically node-negative (cN0) patients with high-risk disease, which includes T1b tumors (T1 with lymphovascular invasion, perineural invasion, or poor differentiation) or any tumor stage T2 or higher (page 18). Therefore, the guideline does recommend lymph node evaluation for a subset of low-stage, clinically node-negative patients, which contradicts the blanket statement in the recommendation. |
| ESMO2024 | R | The guideline supports the recommendation. It specifies that for patients with clinically node-negative (cN0) disease and low-risk features (defined as pTa/pTis and pT1G1), the recommended management is "clinical surveillance of the inguinal nodes." This aligns with the recommendation to not perform lymph node removal, as surveillance is presented as the alternative to active treatment (such as lymph node biopsy or dissection), which is reserved for intermediate- and high-risk patients. |
| NCCN2025 | DR | The NCCN guideline presents a risk-stratified approach that differs from the recommendation's broader statement. While the guideline agrees that low-risk, clinically node-negative patients (PeIN, Ta, T1a) should undergo surveillance without lymph node removal, it explicitly recommends lymph node removal for intermediate/high-risk patients, including those with T1b tumors. This contradicts the recommendation's generalization that lymph node removal is not advised for all low-stage T1 patients, as the guideline's management is dependent on the specific T1 sub-stage. |

**9.13 The management of inguinal nodal disease in transplanted patients should be consistent with standard oncological guidelines.**

[5]

| AUA2020 | NA | The guideline focuses on the treatment of early-stage primary penile tumors, covering topical therapies, laser therapy, and organ-sparing surgery. While it mentions the need to evaluate inguinal lymph nodes for lymphadenopathy and states that higher-grade tumors require inguinal node dissection, it does not provide detailed management strategies for nodal disease. Furthermore, the guideline does not address the specific management of penile cancer or its nodal metastases in the distinct population of transplanted or immunosuppressed patients. |
| --- | --- | --- |
| DGU2020 | NA | The provided guideline offers comprehensive recommendations for the management of regional inguinal lymph nodes in patients with penile cancer, detailing approaches for clinically unsuspicious (cN0), palpable (cN1/cN2), and fixed lymph nodes. However, the document does not specifically mention or provide any distinct recommendations for the subpopulation of transplanted or otherwise immunosuppressed patients. Therefore, the guideline does not address the specific clinical scenario outlined in the treatment recommendation. |
| EAU-ASCO2025 | NA | The EAU-ASCO guideline provides comprehensive recommendations for the management of regional and inguinal lymph node disease in sections 6.2, 6.3, and 6.4. However, the guideline does not specifically mention or provide any distinct guidance for the management of "transplanted patients" or other immunosuppressed populations in this context. While it details the standard oncological approaches, it does not address whether these standards should be applied consistently or modified for this specific patient subgroup. |
| ESMO2024 | NA | and risk stratification. However, the guideline does not mention or provide any specific recommendations for the management of penile cancer in transplanted or otherwise immunosuppressed patients. The topic of how to manage this specific patient subgroup is not addressed. |
| NCCN2025 | NA | The NCCN guideline provides detailed management strategies for inguinal nodal disease based on factors like palpability, bulkiness, and risk stratification from the primary tumor. However, the guideline does not specifically mention or provide distinct recommendations for patients who have undergone organ transplantation. While it details standard oncological guidelines, it does not address whether this specific patient population requires any modification or special consideration, for instance, due to their immunosuppressed state. |

**10. Lymph Node Management (Surgical Approach)**

**10.1 Minimally Invasive Inguinal Lymphadenectomy (MIS-ILND), including video-endoscopic (VEIL) and robot-assisted (RA-VEIL) techniques, is recommended over traditional Open ILND to reduce morbidity. This approach has fewer complications and is considered oncologically equivalent for suitable patients (e.g., cN0 disease) when performed in high-volume centers.**

[10]; [1]; [35]; [31]; [25]; [36]; [33]; [29]

| AUA2020 | NA | The guideline does not address the recommendation to use minimally invasive inguinal lymphadenectomy (MIS-ILND) over open surgery. The provided AUA Update focuses exclusively on the treatment of the primary, early-stage penile tumor, detailing options like topical therapies, laser ablation, and organ-sparing surgical excision. While the document mentions the necessity of evaluating and dissecting inguinal lymph nodes, it does not discuss or compare the specific surgical techniques for performing a lymphadenectomy. |
| --- | --- | --- |
| DGU2020 | R | The guideline supports the use of minimally invasive inguinal lymphadenectomy. On page 68, it explicitly states that minimally invasive techniques, including both video-endoscopic and robot-assisted approaches, have been developed and evaluated to reduce the high complication rates associated with open radical inguinal lymphadenectomy (RIL). The guideline further notes that these techniques can be performed safely and result in lower complication rates with comparable short-term oncological outcomes compared to open surgery, which directly aligns with the treatment recommendation. |
| EAU-ASCO2025 | DR | The guideline suggests a different, more cautious approach than the treatment recommendation. While the guideline acknowledges that minimally invasive techniques (VEIL/RAVEIL) may reduce wound complications (page 28, section 6.2.2.5), it concludes that the evidence is very limited for cN1–2 patients and therefore no standard recommendation can be made for this group. The guideline explicitly states, "Offer minimally-invasive ILND to patients with cN1–2 disease only as part of a clinical trial" (page 28), which contrasts with the recommendation's suggestion of it being a preferred alternative to open ILND. Furthermore, for advanced cN3 disease, the guideline strongly recommends against video endoscopic inguinal lymphadenectomy (page 31). |
| ESMO2024 | R | The guideline supports the recommendation by acknowledging the key benefits of minimally invasive inguinal lymphadenectomy. The text states that minimally invasive approaches (robotic or laparoscopic) "have demonstrated similar oncological outcomes but with less intraoperative blood loss, shorter hospital stay, reduced wound infection rates and reduced skin necrosis rates." Although it adds a note of caution that patient numbers in these series are too small for a definitive conclusion on all outcomes, its overall description aligns with the recommendation's rationale of reduced morbidity without compromising oncological safety. |
| NCCN2025 | DR | The NCCN guideline acknowledges robotic inguinal lymph node dissection (ILND) in the discussion section, noting that it appears "safe and effective, with lower morbidity than open surgery" for select patients. However, it explicitly states that "Data on robotic ILND are limited" (MS-11). The guideline's primary treatment algorithms do not specify a minimally invasive approach, instead recommending "standard or modified ILND" (MS-15), which implies the open procedure is the standard of care. This cautious stance, based on limited data, differs from the treatment recommendation's explicit preference for minimally invasive techniques over the traditional open approach. |

**10.2 For patients with cN1-2 disease, open surgery (O-ILND) should be the preferred option over minimally invasive techniques to reduce the risk of large lymph node fractures during dissection.**

[29]

| AUA2020 | NA | The AUA Update Series guideline focuses exclusively on the "Treatment of Early Stage Squamous Cell Carcinoma of the Penis," covering management of the primary tumor with options like topical therapies, laser ablation, and organ-sparing surgery. The recommendation, however, addresses the surgical technique for managing regional nodal disease (cN1-2), which is a more advanced stage. The provided guideline does not discuss or compare different surgical approaches for inguinal lymph node dissection, such as open versus minimally invasive techniques. |
| --- | --- | --- |
| DGU2020 | DR | The guideline identifies open radical inguinal lymphadenectomy (RIL) as the standard treatment for patients with palpable lymph nodes (cN1/cN2) with proven metastases (p. 67, Recommendation 7.35). However, it contradicts the treatment recommendation's preference for open surgery over minimally invasive techniques. The guideline explicitly states that minimally invasive techniques (videoendoscopic and robot-assisted) have been developed to reduce the high complication rates of open RIL and can achieve lower complication rates with comparable short-term oncological results (p. 68). By presenting minimally invasive approaches as viable and potentially favorable alternatives, the guideline offers a different perspective than the recommendation's clear preference for open surgery. |
| EAU-ASCO2025 | DR | The guideline recommends open radical inguinal lymph node dissection (rILND) as the standard of care for cN1-2 disease and a strong recommendation. However, it does not express a preference for open surgery over minimally invasive techniques due to the risk of lymph node fractures. Instead, it states that evidence for minimally invasive ILND in cN1-2 patients is very limited and, for this reason, strongly recommends that it should only be offered as part of a clinical trial. The guideline does not explicitly prefer one over the other for routine care but rather designates open surgery as the established standard while considering the minimally invasive approach investigational in this specific patient group. |
| ESMO2024 | DR | The guideline recommends radical inguinal lymphadenectomy for cN1-2 disease but does not explicitly state a preference for open surgery over minimally invasive techniques. It acknowledges that minimally invasive (robotic or laparoscopic) approaches have demonstrated benefits such as less blood loss and shorter hospital stays. While it notes the data is from small series, making it difficult to conclude on oncological outcomes, it does not advise against these techniques, which contrasts with the recommendation's clear preference for open surgery. |
| NCCN2025 | DR | The recommendation states that open inguinal lymph node dissection (O-ILND) should be the preferred option for patients with cN1-2 disease over minimally invasive techniques. The NCCN guideline does not endorse this preference. Instead, the discussion section notes that for non-bulky inguinal nodes (which includes cN1-2 disease), the robotic approach is safe, effective, and has lower morbidity than open surgery, although data is limited (MS-11). By presenting minimally invasive surgery as a viable alternative with potential benefits, the guideline differs from the recommendation to outright prefer open surgery. |

**10.3 Modified Inguinal Lymph Node Dissection (ILND) is proposed for patients with high-risk primary penile cancer and clinically negative groins (cN0) to minimize morbidity. The technique involves limiting incision size, preserving the saphenous vein, and excluding dissection lateral to the femoral artery.**

[10]

| AUA2020 | NA | The guideline, "Treatment of Early Stage Squamous Cell Carcinoma of the Penis," focuses exclusively on the management of the primary tumor. It details various treatment options for early-stage lesions, including topical therapies, laser ablation, and organ-sparing surgeries like circumcision and glansectomy. The recommendation, however, pertains to a specific surgical technique for managing regional lymph nodes (Modified ILND) in high-risk, clinically node-negative (cN0) patients. While the guideline states that higher-grade tumors will require inguinal node dissection, it does not discuss the procedure's technique, its modifications, or its application in the cN0 setting. |
| --- | --- | --- |
| DGU2020 | R | The guideline recommends invasive lymph node staging for patients with high-risk (≥ pT1b), clinically node-negative (cN0) penile cancer. It explicitly identifies Modified Inguinal Lymphadenectomy (MIL) as a standard procedure for this indication (Recommendation 7.32). The guideline's description of MIL emphasizes its role in reducing morbidity compared to radical dissection, highlighting features such as preservation of the saphenous vein and limited resection boundaries, which directly aligns with the proposed treatment recommendation. |
| EAU-ASCO2025 | DR | The guideline suggests a different primary approach for surgical staging in high-risk, clinically node-negative (cN0) patients. It strongly recommends dynamic sentinel node biopsy (DSNB) as the preferred initial procedure due to its high diagnostic accuracy and lower complication rates compared to any form of inguinal lymph node dissection (ILND). While the guideline does describe modified ILND as an option developed to lower morbidity (Section 5.2.2.3, page 17), it is positioned as a secondary alternative only when DSNB is not available or feasible (Section 5.3, page 18). |
| ESMO2024 | R | The guideline recommends Modified Inguinal Lymph Node Dissection (ILND) for patients with intermediate- or high-risk clinically node-negative (cN0) disease. It is presented as an alternative surgical option to reduce post-operative morbidity, which aligns with the recommendation's goal. The guideline specifies that Modified ILND should be considered when the preferred procedure, Dynamic Sentinel Lymph Node Biopsy (DSLNB), is not available. |
| NCCN2025 | R | The NCCN guideline recommends modified inguinal lymph node dissection (ILND) for the exact patient population specified in the recommendation. The discussion on "Modified Template Lymphadenectomy" (page MS-11) details a procedure identical to the one proposed, highlighting its role in decreasing morbidity for patients with high-risk tumors and clinically negative groins. The guideline algorithm for non-palpable nodes (PN-3) also states that a "modified/superficial inguinal dissection" is an acceptable alternative for high-risk patients. |

**11. Lymph Node Management (Surgical Technique)**

**11.1 Indocyanine green (ICG) fluorescence-guided ILND is recommended for patients with cN0M0 disease undergoing staging ILND, as it improves the number of retrieved lymph nodes without increasing complications.**

[30]

| AUA2020 | NA | The treatment recommendation specifies the use of indocyanine green (ICG) fluorescence-guided inguinal lymph node dissection (ILND) for staging in patients with clinically node-negative disease. The provided AUA guideline, which focuses on the treatment of early-stage primary penile tumors, does not address this specific surgical technique. Although the guideline acknowledges the necessity of evaluating inguinal lymph nodes and performing dissection in certain cases, it offers no details or recommendations regarding the methods for doing so, such as using ICG or fluorescence guidance. |
| --- | --- | --- |
| DGU2020 | R | The guideline discusses invasive diagnostic options for patients with clinically non-palpable inguinal lymph nodes (cN0). It explicitly mentions that technical enhancements, including the use of indocyanine green (ICG) with a fluorescence camera, can improve the visual identification of sentinel lymph nodes during a dynamic sentinel lymph node biopsy (DSNB). This aligns with the recommendation to use ICG for fluorescence-guided lymph node staging in this patient group. |
| EAU-ASCO2025 | NA | The guideline discusses surgical staging options for clinically node-negative (cN0) patients and mentions dynamic sentinel node biopsy (DSNB) as the preferred method. In this context, it notes that "hybrid radioactive and fluorescent tracers" are a recent innovation, and the cited reference confirms this includes Indocyanine Green (ICG) for sentinel node biopsy. However, the treatment recommendation specifically suggests using ICG-guided fluorescence for a full staging inguinal lymph node dissection (ILND), not just a sentinel node biopsy. The guideline does not address or evaluate the use of ICG for this more extensive ILND procedure. |
| ESMO2024 | DR | The guideline describes a "proven protocol" for dynamic sentinel lymph node biopsy (DSLNB) in patients with cN0 disease that uses a combination of technetium-99m (99mTC) nanocolloid and patent blue dye. While the guideline acknowledges that indocyanine green (ICG) "has also been used" for sentinel node localization, it does not explicitly recommend this technique or discuss its benefits as the treatment recommendation does. The guideline's emphasis on a different tracer combination as the established method constitutes a different recommendation. |
| NCCN2025 | NA | The NCCN guideline provides recommendations for the management of non-palpable inguinal lymph nodes (cN0 disease), including surveillance, bilateral inguinal lymph node dissection (ILND), and dynamic sentinel node biopsy (DSNB). For DSNB, the guideline discussion mentions the use of technetium-99m–labeled nanocolloid and patent blue dye. However, the guideline does not mention or discuss the specific technique of using indocyanine green (ICG) for fluorescence-guided ILND. |

**11.2 To reduce post-operative lymphorrhoea, intraoperative mapping and clipping of lymphatic channels using methylene blue is recommended during therapeutic inguinal dissection.**

[37]

| AUA2020 | NA | The guideline focuses on the diagnosis and treatment of early-stage primary penile squamous cell carcinoma, detailing options such as topical therapies, laser ablation, and organ-preserving surgeries for the penile lesion itself. While it mentions the need to evaluate and dissect inguinal lymph nodes for staging and treatment of more advanced disease, it does not discuss the specific surgical techniques of the inguinal dissection. The recommendation to use methylene blue for intraoperative mapping and clipping of lymphatic channels to reduce lymphorrhea is a technical detail of lymph node surgery that is not addressed in this guideline. |
| --- | --- | --- |
| DGU2020 | R | The guideline acknowledges that inguinal lymphadenectomy is associated with significant morbidity, including complications like lymphoceles. To mitigate these risks, it recommends several measures, including the "application of clips and ligatures for the closure of lymphatic vessels" ("Anwendung von Klips und Ligaturen zum Verschluss von Lymphgefäßen") (p. 68). Although the guideline does not specifically mention the use of methylene blue for intraoperative mapping, it explicitly supports the core action of the recommendation, which is to clip or ligate lymphatic channels to prevent complications like post-operative lymphorrhoea. |
| EAU-ASCO2025 | NA | The guideline extensively discusses the complications associated with radical inguinal lymph node dissection (rILND), including lymphoedema and lymphocele (Section 6.2.2.4). It provides a detailed table (Table 6.2) listing pre-, intra-, and post-operative strategies with positive, negative, or uncertain impacts on morbidity. However, the specific technique of using methylene blue for intraoperative mapping and clipping of lymphatic channels to reduce lymphorrhoea is not mentioned anywhere in the document, including in this table of morbidity-reducing strategies. |
| ESMO2024 | NA | The treatment recommendation suggests using methylene blue for intraoperative mapping and clipping of lymphatic channels during therapeutic inguinal dissection to reduce post-operative lymphorrhoea. The guideline discusses various approaches to inguinal lymphadenectomy, its associated morbidity, and methods to reduce complications, such as a fascial-sparing approach and minimally invasive surgery. However, the guideline does not mention the specific technique of using methylene blue or any other dye for mapping and clipping lymphatic channels. |
| NCCN2025 | NA | The NCCN guideline provides detailed recommendations on the indications for inguinal lymph node dissection (ILND), the extent of the procedure (standard vs. modified), and general post-operative management to reduce complications, such as the use of closed suction drains (MS-11). However, the guideline does not mention or discuss the specific intraoperative technique of using methylene blue to map and clip lymphatic channels. Since this particular method for reducing post-operative lymphorrhea is not addressed, the guideline's stance is classified as Not Addressed. |

**11.3 During inguinal lymphadenectomy, ligation of lymphatic vessels is the preferred technique over diathermic coagulation to reduce morbidity.**

[27]

| AUA2020 | NA | The treatment recommendation specifies a preferred technique (ligation over diathermic coagulation) for managing lymphatic vessels during an inguinal lymphadenectomy to reduce morbidity. The provided AUA guideline, however, is titled "Treatment of Early Stage Squamous Cell Carcinoma of the Penis" and focuses on therapies for the primary tumor, such as topical treatments, laser therapy, and organ-preserving surgery. While the guideline acknowledges the importance of evaluating inguinal lymph nodes for staging, it does not provide any details on the surgical techniques used during a lymphadenectomy, and thus does not mention or compare ligation and diathermic coagulation. |
| --- | --- | --- |
| DGU2020 | R | The guideline recommends several measures to avoid complications during inguinal lymphadenectomy, including the "application of clips and ligatures for the closure of lymphatic vessels" (p. 68, "Anwendung von Klips und Ligaturen zum Verschluss von Lymphgefäßen"). This aligns with the treatment recommendation, which prefers ligation to reduce morbidity. Although the guideline does not explicitly contrast ligation with diathermic coagulation, its positive endorsement of ligation for preventing complications supports the proposed approach. |
| EAU-ASCO2025 | NA | The guideline extensively discusses the morbidity of radical inguinal lymph node dissection (rILND) and strategies to reduce complications in Section 6.2.2.4 and Table 6.2. While Table 6.2 mentions "Ultrasonic device" and "Advanced bipolar device" (which are forms of energy-based coagulation/dissection) as having an "uncertain benefit" on morbidity, it does not mention the technique of "ligation" of lymphatic vessels. The guideline does not make the specific comparison between ligation and diathermic coagulation, nor does it express a preference for either technique. |
| ESMO2024 | NA | The treatment recommendation specifies a preferred surgical technique for managing lymphatic vessels (ligation over diathermic coagulation) during an inguinal lymphadenectomy to reduce morbidity. The ESMO guideline discusses the indications for radical inguinal lymphadenectomy and broader strategies to lower complication rates, such as preserving the saphenous vein and using fascial-sparing approaches. However, the guideline does not address the specific technical detail of how lymphatic vessels should be controlled during the procedure, making no mention of either ligation or diathermic coagulation. |
| NCCN2025 | NA | The NCCN guideline provides detailed recommendations on when to perform an inguinal lymph node dissection (ILND), including the boundaries for standard and modified templates, and aspects of post-operative management. However, the document does not discuss the specific intraoperative surgical techniques for managing lymphatic vessels. There is no mention or comparison of ligation versus diathermic coagulation as methods to reduce morbidity during the procedure. |

**12. Patient Counseling**

**12.1 Patients undergoing surgery where Intraoperative Frozen Section Examination (FSE) is planned must be informed that discordance between FSE and final histology can occur, potentially leading to early local recurrence.**

[20]

| AUA2020 | NA | The guideline mentions "negative intraoperative frozen section margins" as a safe and effective approach for tumor excision in experienced hands. It even states that there is currently no evidence that Mohs surgery is superior to this technique. However, the guideline does not address the specific recommendation of counseling patients about the potential for discordance between frozen section and final histology or the associated risk of recurrence. |
| --- | --- | --- |
| DGU2020 | R | The guideline supports informing patients about the limitations of intraoperative frozen section examination (FSE). Section 4.4.5 explicitly states that FSE ("Schnellschnittproben") is only useful in exceptional cases because the technique can make it difficult to assess dignity, subtyping, and grading, which aligns with the "discordance" mentioned in the recommendation. Furthermore, Chapter 3 on "Umgang mit den Patienten" (Dealing with patients) generally mandates comprehensive patient information regarding the purpose, benefits, and risks of all procedures, which would include the risk of an inaccurate FSE leading to incomplete resection. |
| EAU-ASCO2025 | NA | The guideline discusses the use of intra-operative frozen section assessment in section 6.1.2.2, stating that its role remains "uncertain" and the supporting literature is "heterogeneous and conflicting." It provides a weak recommendation to perform frozen section analysis only in cases of doubt about the completeness of resection. However, the guideline does not address the specific recommendation of informing patients about the potential for discordance between frozen section results and final histology or its associated risks. The guideline's focus is on the clinical utility and indications for the procedure, not the specific content of patient counseling regarding its limitations. |
| ESMO2024 | NA | The guideline recommends intraoperative frozen section analysis as part of a superficial modified inguinal lymph node dissection (ILND) for intermediate- and high-risk cN0 patients when dynamic sentinel lymph node biopsy (DSLNB) is not an option. However, the treatment recommendation focuses on the specific need to counsel patients about the potential for discordance between the frozen section and final histology. The guideline does not mention this potential discordance or make any recommendations regarding patient communication on this topic. |
| NCCN2025 | NA | The NCCN guidelines recommend the use of intraoperative frozen sections for procedures like glansectomy and penectomy to determine negative surgical margins. However, the guidelines do not mention or discuss the process of informing patients about the potential for discordance between frozen section results and final pathology. Consequently, the specific recommendation regarding patient counseling on the limitations of this technique and the associated risk of recurrence is not addressed. |

**12.2 Before performing penile-preserving surgery, clinicians must ensure that patients are capable of and will commit to regular self-examination and attending scheduled follow-up appointments.**

[4]

| AUA2020 | R | The guideline supports the recommendation by emphasizing that patient selection and the ability to maintain surveillance are critical for the success of penile-preserving surgery. It states that "close postoperative follow-up is crucial" and is "required to detect and re-treat any potential recurrences." Most directly, the guideline suggests that more aggressive surgery, such as a penectomy, should be considered in cases where follow-up after an organ-preserving approach is "unreliable," aligning with the recommendation's prerequisite of patient commitment. |
| --- | --- | --- |
| DGU2020 | R | The guideline aligns with the treatment recommendation by emphasizing the importance of follow-up care after primary therapy. Recommendation 8.1 on page 81 states that regular, risk-stratified follow-up should be performed to detect tumor recurrences. Furthermore, Recommendation 8.2 on the same page explicitly states that patients should be taught self-examination for the early detection of recurrences, directly supporting both key components of the proposed recommendation. |
| EAU-ASCO2025 | R | The guideline strongly aligns with this recommendation. In the recommendation table on page 25, it explicitly states to "Offer organ-sparing surgery... to patients with lesions confined to the glans and prepuce... and who are willing to comply with strict follow-up." Conversely, the guideline recommends amputative surgery for patients who are "not willing to comply with strict follow-up." Furthermore, Chapter 7 on follow-up emphasizes that patient education and self-examination are essential parts of surveillance, especially after organ-preserving treatments. |
| ESMO2024 | R | The guideline supports the recommendation by emphasizing the necessity of rigorous follow-up after penile-preserving surgery. It states that as penile-preserving techniques have become the standard of care, "close clinical follow-up is required as recurrence rates are 20%-50%" (p. 11). This high risk of recurrence provides a strong rationale for ensuring patients are committed to attending scheduled appointments, which aligns directly with the treatment recommendation. |
| NCCN2025 | R | The guideline explicitly supports this recommendation. On pages MS-5 and MS-8, it states, "Careful consideration should be given to penile-preserving techniques if the patient is willing and able to undergo close follow-up." The guideline provides a rationale for this on page MS-16, noting that "penile-sparing therapies carry a significantly higher risk of local recurrence (28%) than partial or total penectomy (5%) and thus require closer surveillance." This directly aligns with the recommendation that patient commitment to follow-up is a critical prerequisite for these procedures. |

**13. Perioperative Care**

**13.1 Antibiotic Prophylaxis: • Administer prophylactic antibiotics within 1 hour of incision. • Continue for 24 hours after primary penile surgery. • Continue until removal of surgical drains for ILND.**

[10]

| AUA2020 | NA | The recommendation provides specific instructions for antibiotic prophylaxis related to penile surgery, including timing and duration. The AUA guideline focuses on evaluating and treating early-stage penile cancer with modalities like topical therapies, laser therapy, and various surgical techniques (e.g., glansectomy, Mohs surgery). The document does not address peri-operative management, and there is no mention of antibiotic prophylaxis for surgical procedures. |
| --- | --- | --- |
| DGU2020 | R | The guideline supports the use of prophylactic antibiotics to prevent complications following inguinal lymphadenectomy. On page 68, in the section on radical inguinal lymphadenectomy (7.2.2.1), it states that intravenous antibiotic prophylaxis can be recommended to avoid complications ("Zur Vermeidung von Komplikationen können die intravenöse Antibiotikaprophylaxe... empfohlen werden"). This aligns directly with the treatment recommendation to administer prophylactic antibiotics for inguinal lymph node dissection (ILND). |
| EAU-ASCO2025 | R | The guideline supports the use of prophylactic antibiotics to prevent surgical site infections following inguinal lymph node dissection. Table 6.2 on page 27 lists "Prevention of surgical site infections, e.g., prophylactic antibiotics" as having a "Positive impact" on reducing morbidity. This aligns directly with the treatment recommendation to administer prophylactic antibiotics for surgical procedures like ILND. |
| ESMO2024 | NA | The guideline provides detailed recommendations for the surgical management of primary penile tumors and regional lymph nodes. It acknowledges potential post-operative complications like wound infections, particularly after inguinal lymph node dissection. However, the guideline does not mention or offer any specific guidance on the use of prophylactic antibiotics, including the timing of administration or the duration of treatment, which are the specific points outlined in the treatment recommendation. |
| NCCN2025 | DR | The guideline addresses the use of postoperative antibiotics after inguinal lymph node dissection (ILND) but suggests a different approach than the recommendation. The guideline states that "Consideration should be given to keeping the patient on a suppressive dose of an oral cephalosporin...for several days to weeks postoperatively" for ILND, which differs from the recommendation to continue antibiotics until drain removal. Furthermore, the guideline does not specify an antibiotic protocol for primary penile surgery, unlike the 24-hour course proposed in the recommendation, and it explicitly notes that the data supporting antibiotic use is "very limited." |

**13.2 Thromboprophylaxis (DVT/PE Prevention) for ILND: • Use perioperative sequential compression devices. • Administer prophylactic low-molecular-weight heparin or direct oral anticoagulants. • Encourage early ambulation.**

[10]

| AUA2020 | NA | The treatment recommendation provides specific methods for thromboprophylaxis (DVT/PE prevention) for patients undergoing inguinal lymph node dissection (ILND), including the use of compression devices, anticoagulants, and early ambulation. The AUA guideline, "Treatment of Early Stage Squamous Cell Carcinoma of the Penis," focuses on managing the primary tumor through topical therapies, laser ablation, and organ-sparing surgery. Although the guideline mentions that inguinal node dissection is required for certain tumors, it does not discuss the perioperative management, specific surgical techniques, or prophylactic measures associated with this procedure. |
| --- | --- | --- |
| DGU2020 | R | The guideline aligns with the recommendation by explicitly stating that complications after inguinal lymphadenectomy include thrombosis and pulmonary embolism. To mitigate this risk, the guideline specifies that "The postoperative administration of low-molecular-weight heparin can reduce the occurrence of thrombosis and embolism" (p. 68). Although it does not mention sequential compression devices or direct oral anticoagulants, its direct support for heparin-based thromboprophylaxis is consistent with the core of the treatment recommendation. |
| EAU-ASCO2025 | R | The guideline explicitly supports the use of thromboprophylaxis to reduce morbidity following radical inguinal lymph node dissection (ILND). In Table 6.2 on page 27, "Deep venous thrombosis prophylaxis" is listed as a strategy with a "Positive impact" on patient outcomes. While the guideline does not detail the specific methods mentioned in the recommendation (e.g., heparin, compression devices), it clearly endorses the principle of preventing DVT, aligning with the proposed treatment. |
| ESMO2024 | NA | The treatment recommendation provides a specific protocol for thromboprophylaxis (DVT/PE prevention) for patients undergoing inguinal lymph node dissection (ILND), including mechanical and pharmacological methods. The provided ESMO-EURACAN guideline discusses the surgical management of inguinal lymph nodes and mentions associated morbidities such as high complication rates, wound infections, skin necrosis, and lymphoedema. However, the guideline does not contain any information or recommendations regarding the prevention of thromboembolic events like DVT or PE. |
| NCCN2025 | NA | The NCCN guideline provides detailed information on the indications and surgical techniques for Inguinal Lymph Node Dissection (ILND). However, it does not address the specific perioperative measures for thromboprophylaxis (DVT/PE prevention) mentioned in the treatment recommendation. There is no mention of using sequential compression devices, prophylactic low-molecular-weight heparin, or direct oral anticoagulants. While the guideline notes historical recommendations for bed rest post-ILND, it does not make a definitive recommendation for or against early ambulation. |

**13.3 General patient management should include early ambulation post-surgery and optimization of nutritional support, especially for patients with poor health status.**

[10]; [26]

| AUA2020 | NA | The treatment recommendation specifies general supportive care measures post-surgery, including early ambulation and nutritional support. The AUA guideline focuses in detail on the specific oncologic treatments for early-stage penile cancer, such as topical therapies, laser therapy, and surgical techniques. While it discusses postoperative follow-up for recurrence and procedure-specific complications, it does not address general patient management or supportive care protocols like ambulation or nutrition. |
| --- | --- | --- |
| DGU2020 | R | The guideline recommends a comprehensive rehabilitation approach for patients with penile cancer. Section 8.4 on rehabilitation explicitly mentions "ernährungsmedizinischer Maßnahmen" (nutritional medicine measures), which aligns with the recommendation for optimizing nutritional support. Furthermore, the guideline's emphasis on "aktiver und passiver physikalischer, sporttherapeutischer... Maßnahmen" (active and passive physical, sports therapeutic... measures) as part of a multimodal therapy concept supports the principle of early ambulation to restore function and mobility post-surgery. |
| EAU-ASCO2025 | R | The guideline indirectly supports the recommendation for early ambulation and nutritional optimization. Table 6.2, which discusses factors affecting morbidity after radical inguinal lymph node dissection, lists "Deep venous thrombosis prophylaxis" as having a positive impact; early ambulation is a standard component of this prophylaxis. The same table also identifies "Sarcopenia" (muscle wasting, often linked to poor nutrition) as a negative impact factor, implying that optimizing a patient's nutritional status is beneficial for reducing complications. |
| ESMO2024 | NA | The guideline provides comprehensive recommendations on the diagnosis, staging, and specific oncological treatments for penile cancer, including surgery, radiotherapy, and chemotherapy. While the document mentions that adjuvant radiotherapy can impact ambulation and that lymphoedema teams can help with mobility, it does not make a specific recommendation for "early ambulation post-surgery" as a general management strategy. Furthermore, the guideline does not contain any information or recommendations regarding the optimization of nutritional support for patients. |
| NCCN2025 | NA | The NCCN guideline for penile cancer does not contain specific recommendations regarding post-operative ambulation or nutritional support. The document focuses on oncologic management, including surgical techniques, systemic therapies, radiotherapy, and surveillance schedules. While the discussion section mentions a historical practice of bed rest after inguinal lymph node dissection, it notes this is "debatable" and does not make a current recommendation, effectively leaving the topic of ambulation unaddressed. |

**13.4 Strict diabetes control is crucial for avoiding wound infection-related adverse events in patients undergoing ILND.**

[26]

| AUA2020 | NA | The AUA guideline focuses on the treatment modalities for early-stage squamous cell carcinoma of the primary penile tumor, including topical therapies, laser therapy, and organ-preserving surgeries. The recommendation, however, pertains to the perioperative management of a specific comorbidity (diabetes) for patients undergoing Inguinal Lymph Node Dissection (ILND). While the guideline mentions the need to evaluate and sometimes dissect inguinal nodes, it does not provide any information on the surgical technique, complication management, or perioperative care for ILND. |
| --- | --- | --- |
| DGU2020 | NA | The guideline discusses complications associated with inguinal lymph node dissection, specifically mentioning "Wundinfektionen" (wound infections) as a potential issue (p. 68). It also lists several independent predictors for postoperative complications, including tumor stage, patient age, and BMI. However, the guideline does not mention diabetes as a risk factor or the importance of strict diabetes control for avoiding wound infection-related adverse events. |
| EAU-ASCO2025 | NA | The guideline extensively discusses the complications of radical inguinal lymph node dissection (ILND), including wound infections (2–43%), in section 6.2.2.4. Table 6.2 further outlines various factors that impact morbidity following this procedure. However, the document does not specifically mention diabetes or the importance of strict glycemic control as a factor for mitigating wound infection risk in the context of ILND. |
| ESMO2024 | NA | , including wound complications. However, its discussion on mitigating these risks focuses on surgical techniques, such as fascial-sparing and minimally invasive approaches. The document does not address patient-specific comorbidities like diabetes or recommend specific pre-operative management, such as glycemic control, to reduce adverse events. |
| NCCN2025 | NA | The NCCN guideline extensively covers the surgical procedure of Inguinal Lymph Node Dissection (ILND), including its potential for "surgical morbidity" and "wound-related issues" (MS-11). However, the document does not mention diabetes or any other specific patient comorbidities as risk factors for postoperative complications. Consequently, it does not provide any recommendations regarding glycemic control to mitigate the risk of wound infections following ILND. |

**14. Postoperative Care**

**14.1 Primary Penile Surgery - Wound and Catheter Management: • Dressing: Use compressive surgical dressings for 2-7 days. • Foley Catheter: Typically remove around 7 days post-surgery for patients who undergo neomeatus creation or STSG application.**

[10]

| AUA2020 | NA | The treatment recommendation provides specific details on post-operative care, including the use of compressive dressings for 2-7 days and the timing of Foley catheter removal around day 7. The AUA guideline focuses on the broader treatment strategies for early-stage penile cancer, such as the indications for topical therapies, laser ablation, and various surgical procedures (e.g., glansectomy, Mohs surgery). However, the guideline does not address the specific, practical aspects of post-operative wound or catheter management. |
| --- | --- | --- |
| DGU2020 | NA | The guideline provides a comprehensive overview of the diagnosis, staging, and various treatment options for penile cancer, including detailed surgical procedures like glansectomy and partial amputation. However, it does not specify the protocols for routine post-operative care. The document does not mention the recommended type or duration for compressive surgical dressings, nor does it provide a specific timeline for the removal of a Foley catheter following primary penile surgery. |
| EAU-ASCO2025 | NA | The EAU-ASCO guideline provides extensive detail on the indications, oncological outcomes, and complications of various primary surgical treatments for penile cancer, such as glansectomy and glans resurfacing with skin grafts (STSG). However, the document does not offer specific recommendations or guidance on the practical aspects of post-operative care. There is no mention of the type or duration of surgical dressings (e.g., compressive dressings for 2-7 days) or the specific timing for Foley catheter removal following these procedures. |
| ESMO2024 | NA | The ESMO-EURACAN guideline provides detailed recommendations on the diagnosis, staging, and selection of surgical procedures for penile cancer, including organ-sparing surgeries like glans resurfacing with a split-thickness skin graft (SSG). However, the guideline does not address the specifics of post-operative care. There is no mention of protocols for wound management, such as the type or duration of surgical dressings, nor does it provide guidance on the timing for Foley catheter removal after surgery. |
| NCCN2025 | NA | The NCCN guideline provides detailed recommendations for the diagnosis, staging, and various treatment modalities for penile cancer, including surgical options like glansectomy and the use of split-thickness skin grafts (STSG). However, the guideline does not address the specific details of post-operative care. There is no mention of protocols for wound management, such as the type or duration of surgical dressings, nor are there any recommendations regarding the timing for Foley catheter removal following primary penile surgery. |

**14.2 Inguinal Lymph Node Dissection (ILND) - Specific Management: • Lymphedema Prevention: Use compression stockings. • Fluid Management: Conduct daily measurement of "ins/outs" and regular stripping of surgical drains. • Drain Management: Maintain closed suction drains until output is <30-50 mL per 24 hours. • Foley Catheter: Typically remove on postoperative day 2.**

[10]; [26]

| AUA2020 | NA | The guideline focuses on the treatment of early-stage primary penile tumors, covering topical therapies, laser ablation, and organ-sparing surgical approaches. The treatment recommendation specifies post-operative care protocols following an Inguinal Lymph Node Dissection (ILND), such as drain management and lymphedema prevention. The provided guideline does not discuss the surgical management or subsequent post-operative care for inguinal lymph nodes, and therefore does not address the recommendation. |
| --- | --- | --- |
| DGU2020 | DR | The guideline's stance differs significantly from the treatment recommendation on a key point of lymphedema prevention. The recommendation advises the use of compression stockings. However, the guideline explicitly states on page 68 that a randomized controlled trial failed to prove a benefit for compression stockings in the prophylaxis of lymphedema after inguinal lymphadenectomy. While the guideline does support the use of wound drains, it does not specify the detailed management protocol mentioned in the recommendation, and it does not address the timing of Foley catheter removal at all. The clear statement against the efficacy of compression stockings constitutes a different recommendation. |
| EAU-ASCO2025 | R | The guideline supports the key principles for post-operative management after Inguinal Lymph Node Dissection (ILND) outlined in the recommendation. Table 6.2 (page 27) explicitly lists "Volume-controlled drainage" as having a positive impact on morbidity, which aligns with the recommendation to remove drains when output is below a specific volume (<30-50 mL/24h). Furthermore, Section 7.3.5 (page 41) identifies "compression" and "compression garments" as mainstays of lymphedema management, supporting the recommendation to use compression stockings. While the guideline does not specify the timing for Foley catheter removal or the practice of drain stripping, its alignment on the major strategies for reducing complications indicates a supportive stance. |
| ESMO2024 | NA | The guideline acknowledges that inguinal lymphadenectomy has a high morbidity rate and is associated with complications like lower limb lymphoedema. It recommends addressing lymphoedema-related complications during follow-up and suggests the involvement of dedicated lymphoedema teams. However, the guideline does not provide specific, detailed post-operative management protocols, such as the use of compression stockings, criteria for drain removal, or timing for Foley catheter removal, as outlined in the treatment recommendation. |
| NCCN2025 | NA | The NCCN guideline does not address the specific post-operative management details outlined in the recommendation. The guideline discusses the indications for Inguinal Lymph Node Dissection (ILND) and its surgical technique but omits granular details on perioperative care. While the guideline mentions that "Closed suction drains are placed at surgery and are typically removed when drainage is <50 to 100 mL per day" (MS-11), it does not provide recommendations on compression stockings, daily fluid measurement, or Foley catheter removal timing. |

**15. Primary Treatment**

**15.1 Surgery is the standard, curative treatment for localized penile cancer.**

[12]; [38]; [22]; [39]

| AUA2020 | R | The guideline supports the recommendation by stating that "Most experts consider R0 surgical excision of the penile tumor to be the gold standard of treatment." This aligns directly with the recommendation's claim that surgery is the standard approach. The guideline further reinforces this by noting that "surgery remains the preferred approach" compared to radiation due to its higher success rates. |
| --- | --- | --- |
| DGU2020 | R | The guideline aligns with the recommendation by identifying several treatment options for the primary tumor, including surgery, laser ablation, and radiation therapy, but explicitly states that "operative Therapie im Vordergrund steht" (operative therapy is in the foreground) (p. 43). Subsequent sections detail various surgical procedures, such as partial penectomy, as standard treatments for localized stages T1-T3 (p. 53, 59). While also discussing organ-sparing approaches and alternatives, the guideline consistently positions surgery as the primary and central treatment modality for localized penile cancer, supporting the recommendation. |
| EAU-ASCO2025 | DR | The guideline does not present surgery as the single standard treatment for localized penile cancer. Instead, it advocates for a multi-modal approach where several options are considered. The guideline states that local treatment modalities for localized penile cancer include "topical therapy, laser ablation, excisional surgery, external beam radiotherapy (EBRT) and brachytherapy" (page 19). It gives "Strong" recommendations for both organ-sparing surgery and radiotherapy for selected patients with T1 or T2 lesions, positioning them as viable primary treatments rather than naming surgery as the sole standard (page 25). |
| ESMO2024 | DR | The guideline states that for localized penile cancer (stage I and II), treatment should be carried out with curative intent. However, it presents both surgical resection and radiotherapy (RT), including brachytherapy and/or external beam RT (EBRT), as primary curative options for selected cases. This differs from the recommendation, which posits surgery as the singular standard, curative treatment. By including RT as a valid alternative with curative intent, the guideline offers a broader approach. |
| NCCN2025 | R | The NCCN guideline aligns with the recommendation that surgery is a standard, curative treatment for localized penile cancer. The guideline's treatment algorithms consistently list various surgical interventions as primary options. For example, on page PN-2, treatments for T1, T2, and greater tumors include wide local excision, partial penectomy, and total penectomy. Furthermore, under the "Principles of Surgery" on page PN-B, it is explicitly stated that "Partial penectomy should be considered the standard for high-grade primary penile tumors." While non-surgical options are presented for very early-stage disease, surgery remains a cornerstone of curative-intent therapy for localized penile cancer throughout the guideline. |

**15.2 Penile-preserving/organ-sparing surgery should be considered for all patients with localized penile cancer whenever clinically feasible to preserve organ form and function without compromising oncological outcomes.**

[40]; [20]; [41]; [16]; [4]

| AUA2020 | R | The guideline supports the recommendation, stating there has been a "shift towards organ preserving techniques for patients with low volume disease of favorable histopathology (stages Tis, Ta, T1 and grades 1, 2)." It lists several organ and sexual function preservation approaches, including circumcision, tumor-only excision, Mohs surgery, and glansectomy. The guideline concludes that "Organ preserving approaches are safe and feasible for early stage, low grade squamous cell carcinoma of the penis," directly aligning with the recommendation's stance. |
| --- | --- | --- |
| DGU2020 | R | The guideline strongly supports the recommendation for penile-preserving surgery in localized cancer. Section 7.1 on page 43 explicitly states that the goal is "weitgehender Organerhalt" (extensive organ preservation) and establishes the principle: "as much organ preservation as possible, as much radicality as necessary." Recommendation 7.1 on the same page reinforces this, advising that patients with penile cancer should receive organ-preserving therapy depending on the tumor stage and differentiation. The guideline details various organ-sparing techniques, emphasizing a paradigm shift towards preserving quality of life and sexual function whenever oncologically safe. |
| EAU-ASCO2025 | R | The EAU-ASCO guideline strongly supports the use of penile-preserving/organ-sparing surgery for localized penile cancer. Section 6.1 states that the aims of primary tumor treatment are "complete tumour removal with as much organ preservation as possible" and that "Penile preservation... is considered to be the primary treatment method for localised penile cancer" (p. 19). The guideline further provides a "Strong" recommendation to "Offer organ-sparing surgery and reconstructive techniques to patients with lesions confined to the glans and prepuce (PeIN, Ta, T1–T2)" (p. 25). This aligns directly with the treatment recommendation to prioritize organ-sparing approaches whenever clinically feasible. |
| ESMO2024 | R | The guideline strongly recommends the use of penile-preserving surgery for localized penile cancer, aligning with the treatment recommendation. It explicitly states, "The primary aim of surgical intervention is to remove the tumour using penile-preserving techniques." The guideline further supports this by noting that preserving aesthetic, sexual, and urinary function is a crucial outcome and that "penile-preserving techniques have now become standard of care." |
| NCCN2025 | R | The NCCN guideline strongly supports the recommendation to consider penile-preserving/organ-sparing surgery for localized penile cancer. The guideline dedicates a section to the "Principles of Penile Organ-Sparing Approaches" (PN-A) and lists options like wide local excision, glansectomy, laser therapy, and Mohs surgery as primary treatments for early-stage disease (PeIN, Ta, pT1a) in its algorithms (PN-1, PN-2). This aligns with the recommendation's emphasis on clinical feasibility by specifying that while these approaches are suitable for early lesions, more extensive surgery like partial or total penectomy is standard for higher-grade or more invasive localized tumors (pT1b, T≥2) (MS-8). |

**15.3 For Penile Intraepithelial Neoplasia (PeIN) located on the prepuce, circumcision is the preferred treatment.**

[18]

| AUA2020 | R | The guideline aligns with the recommendation. The treatment algorithm in Figure 4 explicitly lists "Circumcision" as a primary treatment option for a de novo, early/low stage lesion located on the foreskin of an uncircumcised patient. Furthermore, the "SURGERY" section states that "Foreskin circumcision is indicated for preputial disease in uncircumcised men...and can be curative for low grade and low stage disease," which directly supports the use of circumcision for PeIN on the prepuce. |
| --- | --- | --- |
| DGU2020 | R | The guideline directly supports the treatment recommendation. In section 7.1.2, "Behandlung von Tumoren der Vorhaut" (Treatment of tumors of the foreskin), recommendation 7.11 explicitly states that tumors restricted to the foreskin should be treated by circumcision ("Auf die Vorhaut beschränkte Tumoren sollen durch Zirkumzision behandelt werden"). This aligns perfectly with the proposed treatment for Penile Intraepithelial Neoplasia (PeIN) located on the prepuce. |
| EAU-ASCO2025 | R | The guideline aligns with the treatment recommendation. Section 6.1.1, "Treatment of superficial non-invasive disease (PeIN, Ta)," explicitly states that most PeIN lesions are located on the prepuce. Consequently, the guideline asserts, "circumcision should be the primary surgical option," which directly supports the proposed recommendation. |
| ESMO2024 | R | The guideline fully supports the recommendation. In the section on "Premalignant disease (PeIN)," it states that "In cases of biopsy-proven PeIN located on the glans or prepuce, circumcision is mandatory as the initial management." This is reiterated in the final recommendations, which advise that "Circumcision is recommended as the initial treatment" for PeIN in that location, aligning perfectly with the proposed treatment. |
| NCCN2025 | DR | The NCCN guideline does not single out circumcision as the preferred treatment for Penile Intraepithelial Neoplasia (PeIN) on the prepuce. Instead, it presents several penis-preserving techniques as viable options for PeIN. On page PN-1, the primary treatment algorithm for PeIN lists topical therapy, wide local excision, laser therapy, glansectomy, and Mohs surgery. While the "Principles of Penile Organ-Sparing Approaches" on page PN-A states that "Circumcision alone may be reasonable for tumors of the distal prepuce," it is presented as one possibility among many, not as the distinctly preferred method. |

**15.4 For Penile Intraepithelial Neoplasia (PeIN) / Carcinoma in Situ (Tis) of the glans, first-line options include organ-sparing approaches such as topical treatments (e.g., imiquimod, 5-fluorouracil) or laser treatments.**

[18]; [3]

| AUA2020 | R | The guideline supports the recommendation by identifying topical treatments and laser therapy as first-line options for non-invasive penile malignancies, specifically including carcinoma in situ (Tis). The text explicitly states, "Topical treatments, cryotherapy, circumcision, laser and photodynamic therapy are often used as first line treatment options for non-invasive and early stage penile malignancies (carcinoma in situ, Ta and T1)." It further names 5-fluorouracil and imiquimod as the most common topical compounds for these conditions. |
| --- | --- | --- |
| DGU2020 | R | The guideline aligns with the treatment recommendation by advising an organ- and function-preserving curative approach for superficial, non-invasive penile carcinomas, which include Penile Intraepithelial Neoplasia (PeIN) and Carcinoma in Situ (Tis) (p. 45, Recommendation 7.3). It explicitly mentions both topical therapy with 5-fluorouracil or imiquimod (p. 47, Recommendation 7.6) and laser therapy (p. 49, Recommendation 7.8) as applicable treatment options for these conditions. This directly supports the specific organ-sparing methods suggested in the recommendation. |
| EAU-ASCO2025 | R | The guideline supports the recommendation by identifying both topical therapies and laser ablation as treatment options for Penile Intraepithelial Neoplasia (PeIN). Section 6.1.1.1 states that topical therapy with imiquimod or 5-fluorouracil are "effective non-invasive first-line treatment options" for PeIN. Similarly, Section 6.1.1.2 describes laser ablation as an "alternative treatment option." The formal recommendation tables on pages 24-25 further reinforce this by explicitly advising to "Offer topical therapy" and "Offer laser ablation" for patients with biopsy-confirmed PeIN. |
| ESMO2024 | DR | The guideline recommends a different initial approach for Penile Intraepithelial Neoplasia (PeIN). It states that circumcision is the mandatory initial management for PeIN on the glans or prepuce. In contrast, the treatment recommendation suggests topical or laser therapies as first-line options. The guideline reserves these organ-sparing treatments, such as topical 5-FU, imiquimod, or CO2 laser ablation, for managing any residual PeIN following circumcision. |
| NCCN2025 | R | The NCCN guideline supports the recommendation for using organ-sparing approaches for Penile Intraepithelial Neoplasia (PeIN). The treatment algorithm on page PN-1 explicitly lists "Topical therapy" and "Laser therapy" as primary treatment options for patients with a clinical diagnosis of PeIN. Furthermore, the "Principles of Penile Organ-Sparing Approaches" section on page PN-A provides specific details on topical therapy for PeIN, including imiquimod 5% and 5-FU cream, and also discusses the use of laser therapy for PeIN. |

**15.5 For Penile Intraepithelial Neoplasia (PeIN) of the glans after failure of a first-line topical or laser treatment, patients should be offered a second topical or laser treatment.**

[18]

| AUA2020 | DR | The recommendation suggests offering a second topical or laser treatment for Penile Intraepithelial Neoplasia (PeIN) that has failed first-line therapy. The guideline’s treatment algorithm (Figure 4) addresses this scenario under "Recurrent lesion at the same site." While the guideline does include "Laser therapy" as a valid option for this situation, it notably omits topical therapy. Instead, for same-site recurrence, the guideline recommends more definitive treatments such as laser therapy or various forms of surgical excision and glansectomy. |
| --- | --- | --- |
| DGU2020 | DR | The treatment recommendation suggests offering a second course of topical or laser therapy after the failure of the first-line treatment for PeIN. However, the guideline explicitly recommends a different approach. Recommendation 7.27 on page 61 states that local recurrences after topical or laser therapy should be treated with a "different therapy modality" ("einer anderen Therapiemodalität"). This is further supported on page 48, which states that repeating topical therapy after recurrence is "nicht angeraten" (not advisable). |
| EAU-ASCO2025 | DR | The guideline explicitly contradicts the recommendation to offer a second topical treatment after the failure of a first-line therapy. In section 6.1.1.1 and the recommendations table on page 25, it states, "if topical treatment fails, it should not be repeated," due to the risk of underlying invasive disease. While the guideline notes that repeat laser treatment has been described for recurrence (section 6.1.1.2), its firm stance against repeating topical therapy makes the overall guideline approach different from the proposed recommendation, which groups both treatments as viable second-line options. |
| ESMO2024 | DR | The guideline recommends a different treatment pathway. For Penile Intraepithelial Neoplasia (PeIN), it states that if first-line topical treatment fails, "wide local excision or glans resurfacing... should be considered." This moves directly to surgical intervention, whereas the recommendation suggests attempting a second non-surgical treatment (topical or laser) first. |
| NCCN2025 | R | The guideline recommends that a "Recurrence of penile lesion after initial penile sparing treatment" should be managed by treating it "according to recurrence stage (PN-1 and PN-2)" (Page PN-9). For a recurrence that is still classified as Penile Intraepithelial Neoplasia (PeIN), page PN-1 lists several primary treatment options, including topical therapy and laser therapy. This pathway indicates that repeating a topical or laser treatment is a valid and recommended approach for recurrent PeIN, which aligns directly with the provided recommendation. |

**15.6 For extensive or recurrent PeIN of the glans after failure of first- and second-line treatments, surgical options like wide local excision or glans resurfacing should be offered.**

[18]

| AUA2020 | R | The guideline supports the recommendation. The treatment algorithm in Figure 4 explicitly addresses management for a "Recurrent lesion at the same site" and lists "Surgical excision" and "Glans resurfacing" as appropriate options, particularly for lesions on the glans penis. Furthermore, the conclusion states that tumors unresponsive to topical therapy can be managed with surgical approaches, including glans resurfacing, which aligns with the recommendation for recurrent disease after first- and second-line treatments have failed. |
| --- | --- | --- |
| DGU2020 | R | The guideline supports the treatment recommendation. Section 7.1.1.3 specifically discusses "glans resurfacing" ("operative komplette Epithelabtragung der Glans") as a surgical technique for extensive Carcinoma in situ (CIS/PeIN) and for cases of tumor persistence after other treatments like topical or laser therapy. Furthermore, Section 7.1.6, which covers local recurrences ("Lokalrezidiven"), advises that recurrences after topical or laser therapy should be treated with a different modality, implying a shift to surgical options as suggested in the recommendation. |
| EAU-ASCO2025 | R | The guideline aligns with the treatment recommendation. Section 6.1.1.3, "Surgery," explicitly states that "Extensive PeIN, residual PeIN in resection margins or recurrent disease after ablative or topical therapy, can be treated by surgical excision." The guideline proceeds to describe glans resurfacing as a specific surgical technique for this indication. This mirrors the recommendation's proposal to use surgical options, including glans resurfacing, for extensive or recurrent PeIN after other treatments have failed. |
| ESMO2024 | R | The guideline aligns with the recommendation for managing recurrent or extensive PeIN. Under the section "Premalignant disease (PeIN)," the guideline outlines initial treatments such as circumcision followed by topical agents (e.g., 5-FU, imiquimod) or laser ablation. It explicitly states, "If topical treatment fails, wide local excision or glans resurfacing... should be considered," which directly supports offering these surgical options after the failure of first- and second-line treatments as proposed in the recommendation. |
| NCCN2025 | R | The guideline supports the use of wide local excision for Penile Intraepithelial Neoplasia (PeIN) and considers glans resurfacing as a related option. On page PN-1, "Wide local excision" is listed as a primary treatment for PeIN. For recurrent lesions, the guideline on page PN-9 refers back to the initial treatment algorithm on PN-1. Furthermore, the "Principles of Penile Organ-Sparing Approaches" on page PN-A (2 of 2) explicitly designates wide local excision for PeIN and adds that "Glans resurfacing may be considered in highly select patients." |

**15.7 For early-stage, superficial, or non-invasive penile cancer, treatment typically involves limited excision or non-invasive therapies like topical treatments.**

[38]; [22]

| AUA2020 | R | The guideline supports the recommendation for using limited excision and non-invasive therapies for early-stage penile cancer. The "TOPICAL THERAPIES" section explicitly states that treatments like topical 5-FU and imiquimod are often used as first-line options for non-invasive and early-stage malignancies (carcinoma in situ, Ta, and T1). Furthermore, the "SURGERY" section notes a shift toward organ-preserving techniques, including tumor-only excision, for patients with low-volume, early-stage disease, which aligns with the recommendation for limited excision. |
| --- | --- | --- |
| DGU2020 | R | The guideline aligns with the treatment recommendation. Section 7.1.1, "Behandlung von oberflächlichen, nicht-invasiven Tumoren und Präkanzerosen der Glans" (Treatment of superficial, non-invasive tumors and precancerous lesions of the glans), explicitly supports organ- and function-preserving curative treatments for these early-stage cancers (Recommendation 7.3). Furthermore, Section 7.1.1.1 specifically discusses and allows for the use of topical chemotherapy with 5-Fluorouracil or Imiquimod (Recommendation 7.6), which directly corresponds to the "non-invasive therapies like topical treatments" mentioned in the recommendation. |
| EAU-ASCO2025 | R | The guideline strongly supports the recommendation. Section 6.1.1, "Treatment of superficial non-invasive disease (PeIN, Ta)," explicitly discusses therapies for early-stage penile cancer. It details the use of non-invasive topical therapies like imiquimod and 5-fluorouracil in section 6.1.1.1, referring to them as "effective non-invasive first-line treatment options." The guideline also describes various organ-sparing surgical approaches, such as circumcision and surgical excision, which align with the concept of limited excision for these conditions. |
| ESMO2024 | R | The guideline supports the recommendation for treating early-stage penile cancer with limited excision and non-invasive therapies. The guideline details several organ-sparing surgery (OSS) options for localized penile cancer, aligning with the concept of limited excision. Specifically for premalignant disease (PeIN) and Ta-1 disease, it recommends wide local excision, glans resurfacing, and the use of topical agents like 5-fluorouracil (5-FU) or imiquimod. |
| NCCN2025 | R | The NCCN guideline supports the recommendation for using limited excision and non-invasive therapies for early-stage penile cancer. The guideline's primary treatment algorithm (PN-1) for non-invasive disease (PeIN or Ta) lists "Topical therapy" and "Wide local excision" as primary treatment options. Furthermore, the "Principles of Penile Organ-Sparing Approaches" (PN-A) section explicitly states that PeIN, Ta, and T1 lesions are amenable to conservative approaches, including topical therapy, wide local excision, and Mohs surgery, which aligns directly with the provided recommendation. |

**15.8 For early-stage penile SCC, treatment options include Wide Local Excision (WLE), Mohs Micrographic Surgery (MMS), glansectomy, or partial penectomy.**

[42]

| AUA2020 | R | The guideline supports the treatment recommendation. The "SURGERY" section of the AUA Update Series explicitly lists the "4 commonly used surgical approaches for the treatment of early stage penile cancer," which include "tumor-only excision" (Wide Local Excision), "Mohs surgery," and "glansectomy." The guideline also states that more aggressive approaches like "partial and total penectomy are acceptable options" for patients with unfavorable histopathology or recurrent disease, aligning with all treatments mentioned in the recommendation. |
| --- | --- | --- |
| DGU2020 | R | The guideline aligns with the treatment recommendation by listing several surgical options for early-stage (T1/T2) tumors. Specifically, section 7.1.3 discusses "Behandlung invasiver auf die Glans beschränkter Tumoren (T1/T2)" and recommends local excision ("Lokalexzision"), glansectomy ("Glansektomie"), and partial penectomy ("Penisteilamputation") in recommendations 7.14, 7.15, and 7.16, respectively. While Mohs surgery is not given its own recommendation, it is mentioned in the background text (page 55) as a valid organ-sparing technique reviewed by the guideline authors, thus covering all modalities suggested in the recommendation. |
| EAU-ASCO2025 | R | The EAU-ASCO guideline supports the treatment recommendation for early-stage penile SCC. Section 6.1.2, "Treatment of invasive disease confined to the glans (cT1/T2)," details the surgical options, which align with those listed in the recommendation. Specifically, the guideline discusses Wide Local Excision (6.1.2.5), Mohs micrographic surgery (6.1.2.4), Glansectomy (6.1.2.7), and Partial penectomy (6.1.2.8) as treatment modalities for early-stage (T1/T2) disease. Although it notes that Mohs surgery is not routinely recommended due to limited data, it is still presented as a potential option, thus endorsing the recommendation's list of possible treatments. |
| ESMO2024 | R | The guideline supports the recommendation by detailing various organ-sparing surgical options for localized (early-stage) penile cancer. It specifically recommends Wide Local Excision (WLE) for Ta-1 disease and glansectomy for T2 tumors. The guideline also includes Mohs micrographic surgery as a conditional option for low-grade T1 lesions and partial penectomy as a valid alternative for T2-T3 disease, aligning with all the treatment options listed in the recommendation. |
| NCCN2025 | R | The NCCN guideline aligns with the treatment recommendation by listing all the proposed surgical options for early-stage penile cancer. The "Primary Treatment" algorithm on page PN-2 recommends "Wide local excision," "Partial penectomy," "Glansectomy in select cases," and "Mohs surgery in select cases" for pathologic stage pT1a. Furthermore, the "Principles of Penile Organ-Sparing Approaches" section (PN-A, page 17) explicitly details Wide Local Excision, Glansectomy, and Mohs Micrographic Surgery for early-stage disease (PeIN, Ta, T1), and the "Principles of Surgery" section (PN-B, page 18) discusses penectomy. |

**15.9 For localized penile cancer (stages pTis, pTa, pT1), viable penile-sparing surgery options include circumcision (if limited to foreskin), wide local excision (WLE), laser ablation, glans resurfacing, and glansectomy.**

[3]; [16]; [43]

| AUA2020 | R | The AUA guideline supports the treatment recommendation, outlining a shift toward organ-preserving techniques for early-stage penile cancer (stages Tis, Ta, T1). The "SURGERY" section on page 76 explicitly lists "circumcision, tumor-only excision, ... and glansectomy" as commonly used surgical approaches. The guideline also details laser therapy (page 75) and mentions that glans resurfacing has "gained interest" (page 76), confirming that all proposed treatments are considered viable options. |
| --- | --- | --- |
| DGU2020 | R | The guideline aligns with the treatment recommendation by discussing and supporting the same penile-sparing surgical options for localized penile cancer. For tumors confined to the foreskin, it recommends circumcision (Recommendation 7.11). For superficial, non-invasive tumors (pTis/CIS), it details treatments such as laser therapy and glans resurfacing (Sections 7.1.1.2 and 7.1.1.3). Furthermore, for invasive tumors limited to the glans (pTa/pT1), it presents options including local excision (WLE) and glansectomy (Sections 7.1.3.1 and 7.1.3.2), confirming the approaches listed in the recommendation. |
| EAU-ASCO2025 | R | The guideline supports the use of penile-sparing surgery for localized penile cancer. Section 6.1 is dedicated to the treatment of the primary tumor, with subsections 6.1.1 covering superficial non-invasive disease (PeIN, Ta) and 6.1.2 covering invasive disease confined to the glans (cT1/T2). These sections and the corresponding recommendations table on page 25 explicitly discuss circumcision, wide local excision, laser ablation, glans resurfacing, and glansectomy as treatment options for these stages, fully aligning with the proposed recommendation. |
| ESMO2024 | R | The guideline aligns with the recommendation by endorsing various penile-sparing surgical options for localized penile cancer. Specifically, for premalignant disease (PeIN or Tis) and Ta-1 disease, the guideline text recommends circumcision, wide local excision (WLE), CO2 laser ablation, and glans resurfacing. The management algorithm in Figure 2 further confirms these options and includes glansectomy as a viable treatment for T1 or T2 tumors, which is consistent with the proposed recommendation. |
| NCCN2025 | R | The NCCN guideline recommends several penile-sparing approaches for localized penile cancer. The treatment algorithm for Penile Intraepithelial Neoplasia (PeIN, equivalent to pTis) and Ta disease lists wide local excision, laser therapy, and glansectomy as primary treatment options (PN-1). The "Principles of Penile Organ-Sparing Approaches" section (PN-A) further specifies that these options, along with circumcision for prepuce tumors and glans resurfacing, are appropriate for PeIN, Ta, and T1 lesions, which directly aligns with the recommendation. |

**15.10 Glansectomy is the indicated treatment and widely accepted procedure of choice for invasive penile cancer confined to the glans, including stage cT2 (invasion of glans spongiosum).**

[16]; [4]

| AUA2020 | R | The guideline supports the treatment recommendation by repeatedly identifying glansectomy as a primary surgical option for penile cancer. It lists glansectomy as one of the four "commonly used surgical approaches for the treatment of early stage penile cancer... for tumors on the glans penis." Furthermore, the guideline's treatment algorithm (Figure 4) explicitly includes partial and total glansectomy as options for de novo lesions located on the glans, aligning with the recommendation's stance. |
| --- | --- | --- |
| DGU2020 | R | The guideline supports the recommendation. Section 7.1.3 discusses the treatment of invasive tumors confined to the glans (T1/T2). Specifically, recommendation 7.15 (page 52) states with a strong recommendation grade (A) that glansectomy should be offered to patients with T1/T2 tumors, depending on the tumor's location and extent. This aligns with the proposed treatment that glansectomy is the indicated procedure of choice for these stages. |
| EAU-ASCO2025 | R | The guideline supports the use of glansectomy for invasive penile cancer confined to the glans. Section 6.1.2.7 states that "Patients with tumours confined to the glans and prepuce that are not eligible for wide local excision or glans resurfacing are good candidates for glansectomy." The guideline's systematic review assessed glansectomy in patients with T1-T2 disease, confirming its effectiveness with a five-year recurrence-free rate ranging from 78.0–95.8%. While it is presented as one of several organ-sparing options, its prominent role and proven efficacy for the specified stage align with the treatment recommendation. |
| ESMO2024 | R | The guideline aligns with the treatment recommendation, stating that "Glansectomy, with or without distal urethrectomy, is the surgical treatment of choice for T2 tumours on the glans penis." In its formal recommendations section for T2-4 disease, it further specifies that "In T2 tumours of the glans penis, glansectomy...is recommended." This directly supports the proposed treatment for invasive cancer confined to the glans, including stage T2. |
| NCCN2025 | DR | The NCCN guideline recommends more extensive surgery for T2 penile cancer, which is defined as invasion into the corpus spongiosum. The primary treatment algorithm for "T2 or greater" lists partial penectomy or total penectomy, not glansectomy (PN-2). The guideline explicitly states that glansectomy is a penile organ-sparing approach considered for select patients with less invasive tumors (clinical stage T1) (PN-A, p. 2 of 2). The discussion section reinforces this, stating that T≥2 lesions "typically require more extensive surgical intervention with partial or total penectomy" (MS-8). |

**15.11 For invasive or advanced penile cancer, more extensive surgical intervention is required, such as partial or total penectomy.**

[42]; [22]; [23]; [3]

| AUA2020 | R | The guideline supports the recommendation, stating that for higher stage/grade tumors, treatment would involve partial or total penectomy. It further clarifies that these "more aggressive surgical approaches such as partial and total penectomy are acceptable options for patients with unfavorable histopathology or recurrent disease." This directly aligns with the recommendation's assertion that extensive surgery is required for invasive or advanced penile cancer. |
| --- | --- | --- |
| DGU2020 | R | The guideline aligns with the recommendation for surgical intervention in invasive and advanced penile cancer. For T3 tumors involving the corpora cavernosa, Recommendation 7.22 states that a partial penectomy should be performed, and a radical penectomy may be necessary for extensive tumors. Furthermore, for locally advanced T4 tumors, Recommendation 7.24 calls for extensive operative resection, which the background text clarifies involves radical penectomy. |
| EAU-ASCO2025 | R | The guideline recommends partial or total penectomy for invasive and advanced penile cancer. Section 6.1.3.1 states that for cT3 patients with obvious involvement of the corpora cavernosa, "partial amputation is standard," and that "Extensive partial amputation with wide margins or total penectomy...is the standard advisable treatment." Furthermore, the recommendations table on page 25 strongly advises offering partial penectomy for T3 disease and total penectomy for large invasive tumors that are not suitable for partial amputation. |
| ESMO2024 | R | The guideline supports the recommendation for more extensive surgical intervention in cases of invasive or advanced penile cancer. Specifically, for T3 disease where the cancer infiltrates the corpus cavernosum, the guideline identifies "Partial penectomy or total penectomy... are the treatments of choice." For more extensive T4 disease, the guideline states that "total penectomy with perineal urethrostomy is the recommended option." This aligns directly with the proposed treatment recommendation. |
| NCCN2025 | R | The guideline recommends partial or total penectomy for invasive penile cancer. The treatment algorithm on page PN-2 lists partial and total penectomy as primary treatment options for pathologic stages pT1b and T2 or greater. Furthermore, the "Principles of Surgery" section on page PN-B states that "Partial or total penectomy is necessary when there is invasion into the corpora cavernosum." This directly aligns with the recommendation for more extensive surgical intervention for invasive disease. |

**15.12 For patients with T3 disease (invasion of corpora cavernosa), organ-sparing surgical approaches are usually precluded. Complete or partial penectomy is warranted.**

[15]

| AUA2020 | R | The guideline, while primarily focused on early-stage penile cancer, directly supports the recommendation. It explicitly states that "for higher stage/grade tumors this would mean partial or total penectomy," contrasting this with organ-preserving techniques suitable for lower-stage disease (Tis, Ta, T1). This aligns perfectly with the recommendation that for T3 disease (a higher stage involving corpora cavernosa), organ-sparing approaches are usually precluded in favor of a partial or complete penectomy. |
| --- | --- | --- |
| DGU2020 | R | The guideline aligns with the treatment recommendation by stating that partial penectomy is the therapy of choice for T3 tumors (invasion of the corpora cavernosa). Recommendation 7.22 on page 59 specifies that a partial penectomy should be performed for T3 tumors, and a radical penectomy may be necessary for extensive disease. This directly supports the recommendation's assertion that complete or partial penectomy is warranted for this stage of disease. |
| EAU-ASCO2025 | R | The guideline supports the recommendation that amputative surgery is the standard for T3 penile cancer. Section 6.1.3.1 states, "For cT3 patients with obvious involvement of the corpora cavernosa, partial amputation is standard." Furthermore, the recommendations table on page 25 provides a "Strong" recommendation to "Offer partial penectomy, with or without reconstruction, to patients with invasion of the corpora cavernosa (T3)." This directly aligns with the proposal that partial or complete penectomy is warranted for this stage of disease. |
| ESMO2024 | R | The guideline aligns with the recommendation. On page 5, under the heading "T3 disease," the guideline explicitly states that "Partial penectomy or total penectomy combined with a perineal urethrostomy are the treatments of choice when the cancer infiltrates proximally into the corpus cavernosum." This is further summarized in the "Recommendations" section on page 9, which advises that partial or total penectomy is recommended for cancer infiltrating the corpus cavernosum (T3-T4). |
| NCCN2025 | R | The NCCN guideline aligns with the treatment recommendation. According to the "Principles of Surgery" (page PN-B), "Partial or total penectomy is necessary when there is invasion into the corpora cavernosum in order to achieve a negative margin." This directly corresponds to the definition of T3 disease and supports the recommendation that penectomy is warranted. Furthermore, the treatment algorithm for T2 or greater disease (page PN-2) lists partial and total penectomy as primary treatment options, reinforcing that organ-sparing approaches are typically precluded for this stage. |

**15.13 For penile SCC (particularly smaller, T1 lesions), Mohs Micrographic Surgery (MMS) should be considered an effective option, as it is associated with a lower local recurrence rate compared to Wide Local Excision (WLE).**

[42]; [40]

| AUA2020 | DR | The guideline contradicts the recommendation's assertion that Mohs surgery offers superior oncologic outcomes. While the guideline identifies Mohs surgery as a treatment option for early-stage disease, it explicitly states, "Currently there is no evidence to suggest Mohs surgery is superior to negative intraoperative frozen section margins in terms of oncologic outcomes." Furthermore, it cites a retrospective study where 32% of patients experienced recurrence after Mohs surgery, which does not support the claim of a lower recurrence rate compared to other standard excisions. |
| --- | --- | --- |
| DGU2020 | NA | The S3 guideline does not make a recommendation for or against the use of Mohs Micrographic Surgery (MMS). For T1/T2 tumors, the guideline lists operative treatment methods such as local excision, laser therapy, glansectomy, and partial penectomy. Mohs surgery is mentioned only once in the background information as part of a summary of a review article that analyzed various organ-sparing techniques, but it is not discussed further or included in the guideline's own therapeutic recommendations. |
| EAU-ASCO2025 | DR | The guideline explicitly states that Mohs micrographic surgery is "not routinely recommended" for treating penile cancer because the available data are "very limited." This directly contrasts with the treatment recommendation, which frames Mohs surgery as an effective option that should be considered. Although the guideline acknowledges the technique and reports high recurrence-free rates in a small number of patients, its overall conclusion is to advise against routine use, a different and more cautious stance than the one proposed. |
| ESMO2024 | DR | The guideline presents a different and more cautious view on Mohs Micrographic Surgery (MMS) compared to the recommendation. The recommendation claims MMS has a lower local recurrence rate, but the guideline explicitly states that for T1 lesions, "there is a high recurrence rate (32%)" associated with MMS. While the guideline does list MMS as a potential option for low-grade T1 lesions, its recommendation is highly conditional, stating it may be used "only if the right clinical set-up, including a pathologist, is available," and it highlights significant drawbacks that contradict the recommendation's positive assertion. |
| NCCN2025 | R | The NCCN guideline recommends Mohs surgery as a primary treatment option (category 2B) for early-stage penile cancer, including pT1a lesions. The guideline explicitly positions Mohs surgery as an "alternative to wide local excision in select cases" (PN-A 2 of 2). Furthermore, the discussion section (MS-7) cites a systematic review that found a 4% recurrence rate for PeIN treated with Mohs surgery versus a 25% recurrence rate for wide local excision, supporting the recommendation's claim of lower recurrence. |

**15.14 Radiation therapy is an alternative organ-preserving approach for primary tumors, particularly for T1-2 lesions < 4 cm in diameter. Brachytherapy may offer better recurrence-free rates compared to External Beam Radiotherapy (EBRT).**

[3]; [34]; [40]

| AUA2020 | DR | The guideline suggests a different treatment hierarchy than the recommendation. While the guideline acknowledges that radiation therapy can be considered for early-stage (T1-T2) disease, it explicitly states that "surgery remains the preferred approach" due to higher success rates. It positions radiation as an option for patients who are "unfit for surgery" and willing to accept significant side effects, noting it is associated with "more local complications and lower control rates." This contrasts with the recommendation, which presents radiation therapy as a more standard organ-preserving alternative without these strong qualifications. |
| --- | --- | --- |
| DGU2020 | R | The guideline recommends local radiation therapy as an organ-preserving treatment option for T1 and T2 tumors (Recommendation 7.19). The background information supports the recommendation's specific points by noting that brachytherapy generally reports better results than percutaneous radiation therapy (EBRT) (p. 57). Additionally, the guideline mentions that tumor size greater than 4 cm is a significant risk factor for recurrence, aligning with the recommendation's focus on smaller tumors (p. 58). |
| EAU-ASCO2025 | R | The guideline strongly aligns with the treatment recommendation. Section 6.1.2.9 identifies radiotherapy as an organ-preserving approach for selected patients with T1-2 lesions and gives this a "Strong" recommendation. The guideline specifies that brachytherapy has been studied for lesions < 4 cm, matching the recommended size criteria. Furthermore, it provides evidence supporting that brachytherapy offers better outcomes, citing a cumulative mean 5-year recurrence-free rate (RFR) of 78.6% for brachytherapy compared to 55.2% for EBRT. |
| ESMO2024 | R | The guideline supports using radiation therapy (RT) as an organ-preserving treatment for localized penile cancer, aligning with the recommendation. It specifically states that optimal candidates for RT have superficial lesions measuring < 4 cm. Furthermore, the guideline directly addresses the comparison between RT modalities, noting that a literature review found External Beam Radiotherapy (EBRT) had a "significantly worse local control rate" compared to brachytherapy, which substantiates the recommendation's claim of better recurrence-free rates with brachytherapy. |
| NCCN2025 | R | The NCCN guideline aligns with the recommendation by listing radiation therapy (RT) as a primary, organ-preserving treatment option for T1 and T2 tumors (p. PN-2, PN-C). The guideline specifically uses the < 4 cm tumor size criterion to guide RT choices for T1-2, N0 disease (p. PN-C). Furthermore, it supports the recommendation's preference for brachytherapy by stating it is the "preferred" RT modality for pT1a lesions (p. MS-8). |

**15.15 For patients with HPV-related PSCC, conservative surgical approaches, such as organ-preserving techniques, may be considered in appropriately selected cases given the favorable prognosis.**

[13]

| AUA2020 | DR | The guideline recommends organ-preserving surgery based on clinical and pathological staging, specifically for patients with low-volume, low-stage (Tis, Ta, T1), and low-grade (1, 2) disease with favorable histopathology. In contrast, the treatment recommendation bases the decision for conservative approaches specifically on the patient's HPV status, linking it to a favorable prognosis. While the guideline acknowledges HPV as a major risk factor, it does not use HPV etiology as a criterion for selecting a surgical approach, making the basis for the recommendation different. |
| --- | --- | --- |
| DGU2020 | R | The guideline strongly advocates for organ-preserving therapy ("Organerhalt") as a primary goal in treating penile cancer to maintain the patient's quality of life, stating the principle "as much organ preservation as possible, as much radicality as necessary" (p. 43). It also acknowledges that HPV-associated penile cancer has a better prognosis and higher disease-specific survival (p. 31, section 4.4.3). Therefore, the guideline's general principle of pursuing organ preservation, particularly in cases with a favorable prognosis, aligns with and supports the specific recommendation to consider conservative surgery for HPV-related PSCC. |
| EAU-ASCO2025 | DR | The guideline recommends organ-sparing surgery for localized penile cancer based on the tumor's stage and location, specifically for lesions confined to the glans and prepuce (e.g., PeIN, Ta, T1–T2). This recommendation is independent of the tumor's HPV status. In contrast, the proposed recommendation suggests using HPV status and its associated favorable prognosis as a key factor in selecting patients for conservative surgery. Although the guideline acknowledges that HPV-positive cases have a better prognosis, its specific treatment recommendations are stratified by T-stage, not by HPV status. |
| ESMO2024 | DR | The guideline recommends treatment strategies for penile cancer based on tumor stage and grade, not on HPV status. In fact, the guideline explicitly states that “No established prognostic or treatment differences between HPV-associated and HPV-independent penile cancers currently exist.” While the guideline does advocate for penile-preserving techniques in early-stage disease (e.g., T1-T2), this recommendation is applied regardless of HPV aetiology, making its stance different from the provided recommendation, which specifically suggests this approach for HPV-related cases. |
| NCCN2025 | R | The NCCN guideline supports the recommendation by extensively detailing conservative, penile organ-sparing approaches for appropriately selected cases. Specifically, for early-stage disease (PeIN, Ta, and T1), the guideline lists options such as wide local excision, laser therapy, glansectomy, and Mohs surgery (PN-A, pp. 16-17; MS-5, p. 34). The guideline also mandates assessing HPV status during the primary evaluation (PN-1, p. 6) and notes in the discussion that HPV positivity is a favorable prognostic factor (MS-3, p. 32), aligning with the rationale provided in the recommendation. |

**15.16 For Penile Verrucous Carcinoma (PVC) tumors < 3 cm and classified as Stage T1, local excision with minimal surgical margins followed by careful observation is recommended as the first-line treatment.**

[14]

| AUA2020 | NA | The treatment recommendation is for a specific histological subtype: Penile Verrucous Carcinoma (PVC). The AUA guideline provides a comprehensive overview of treatment for early-stage Squamous Cell Carcinoma (SCC) in general, based on tumor stage and location. However, the document never mentions "Verrucous Carcinoma" or "PVC." Therefore, the guideline does not address the specific management of this subtype, even though the proposed treatment (local excision) is discussed as a valid option for SCC broadly. |
| --- | --- | --- |
| DGU2020 | R | The guideline supports the recommendation for local excision of small, invasive T1 tumors as a reasonable organ-preserving strategy (Recommendation 7.14). It further specifies that minimal surgical margins of a few millimeters are considered oncologically safe, aligning with the proposed approach (Recommendation 7.17). Finally, the guideline recommends clinical surveillance for low-risk tumors, explicitly including verrucous carcinoma (pTa) and low-grade T1 tumors (pT1aG1), which corresponds to the "careful observation" follow-up in the recommendation (Recommendation 7.29). |
| EAU-ASCO2025 | R | The guideline supports the use of organ-sparing surgery with minimal margins for less aggressive lesions. It characterizes Verrucous Carcinoma as an "extremely well-differentiated" subtype with "no metastasis reported" (Table 3.1, page 7), which aligns with the profile of a low-risk tumor. The guideline recommends organ-sparing treatments like "wide local excision" for T1 tumors (Section 6.1.2, page 20) and explicitly states that for "smaller and less aggressive lesions... macroscopic margins can indeed be minimal" (Section 6.1.2.1, page 21). This directly supports the recommendation for local excision with minimal margins as a first-line treatment for this specific cancer type. |
| ESMO2024 | R | The guideline supports the recommended treatment. It identifies verrucous carcinoma as a subtype of non-HPV-related penile cancer. For Ta-1 disease, which includes T1 tumors, the guideline recommends organ-sparing surgery, stating that "Wide local excision of the lesion...is preferable for small tumours" (p. 4). This aligns directly with the recommendation for local excision for small, Stage T1 Penile Verrucous Carcinoma tumors. |
| NCCN2025 | R | The NCCN guideline identifies verrucous carcinoma as a low-risk, well-differentiated tumor with the best prognosis that does not metastasize (page PN-F). The guideline recommends penile-preserving techniques, including "Wide local excision," as a primary treatment for low-risk pT1a disease, provided the patient can undergo close follow-up (pages PN-2, MS-8). This approach aligns directly with the recommendation for local excision and careful observation for T1 Penile Verrucous Carcinoma. |

**15.17 For tumors initially thought to be Penile Verrucous Carcinoma (PVC) but later considered not to be based on findings, the excision should be widened.**

[14]

| AUA2020 | NA | The guideline provides a comprehensive overview of treatment options for early-stage squamous cell carcinoma of the penis, including topical therapies, laser ablation, and various surgical techniques. However, it does not specifically mention Penile Verrucous Carcinoma (PVC) or the management of tumors that are reclassified after initial diagnosis. The recommendation's specific instruction to widen an excision based on a changed diagnosis from PVC is not addressed in the text. |
| --- | --- | --- |
| DGU2020 | R | The guideline acknowledges that diagnostic discrepancies between initial biopsy and final pathology are common, particularly regarding histological subtype and grading (p. 33). The guideline's core principle for surgical treatment is to achieve tumor-free resection margins based on the definitive pathology (Rec. 7.18, p. 54). Since verrucous carcinoma (PVC) is considered to have low metastatic potential, a change in diagnosis to a more aggressive form of squamous cell carcinoma would necessitate a re-evaluation of the surgical margins. Therefore, the recommendation to widen the excision aligns with the guideline's principle of tailoring the surgical approach to the final, more aggressive histopathological findings to ensure oncological safety. |
| EAU-ASCO2025 | R | The guideline characterizes Verrucous Carcinoma as a low-risk, non-invasive (Ta) subtype with no reported metastasis. If a tumor initially thought to be verrucous carcinoma is later found to be a different, more aggressive subtype, its risk classification would increase. The guideline recommends that for "bulky or higher-grade lesions... adoption of a wider margin or partial penectomy may be prudent" (p. 21, Section 6.1.2.1). Therefore, widening the excision for a tumor re-diagnosed from low-risk verrucous carcinoma to a higher-grade cancer aligns with the guideline's principle of adjusting surgical margins based on tumor risk. |
| ESMO2024 | NA | The guideline identifies verrucous carcinoma as a histological subtype of non-HPV-related penile cancer. However, the document's treatment recommendations for local disease are based on the tumor's T-stage (e.g., Tis, Ta, T1-T4) rather than specific histological subtypes. The guideline does not address the clinical scenario of a revised diagnosis from suspected Penile Verrucous Carcinoma (PVC) or the corresponding action to widen the excision. |
| NCCN2025 | R | The NCCN guideline characterizes verrucous carcinoma as a low-risk subtype with a favorable prognosis that is often treated with conservative, organ-sparing approaches like wide local excision (MS-8, PN-F). The recommendation addresses a scenario where the diagnosis changes to a more aggressive histology after initial treatment. The guideline recommends escalating treatment based on the new, higher-risk pathologic diagnosis, which may include more extensive surgery such as partial or total penectomy to achieve negative margins (PN-2, MS-8). This escalation of surgical intervention is consistent with the recommendation to "widen the excision." |

**15.18 For Stage T2 penile cancers (defined as invasive cancers different from PVC), aggressive treatment is recommended.**

[14]

| AUA2020 | R | The guideline supports the recommendation for aggressive treatment for Stage T2 penile cancer. It consistently defines "early stage" cancer as Tis, Ta, and T1, for which it discusses organ-preserving therapies. In contrast, it explicitly states that for "higher stage/grade tumors," the appropriate treatment would be more aggressive approaches like "partial or total penectomy." |
| --- | --- | --- |
| DGU2020 | R | The guideline addresses the treatment of invasive T1/T2 tumors in section 7.1.3. It outlines several definitive, potentially curative treatment options that can be considered "aggressive." Specifically, it provides a strong recommendation (Grade A) for glansectomy (Recommendation 7.15) and, if that is not feasible, for partial penile amputation (Recommendation 7.16). While also discussing organ-sparing alternatives like radiotherapy, these substantial surgical interventions align with the recommendation for aggressive treatment of invasive Stage T2 cancers. |
| EAU-ASCO2025 | DR | The guideline presents a different and more nuanced approach than the one recommended. For T2 penile cancer, the guideline strongly recommends organ-sparing treatments, such as organ-sparing surgery (e.g., glansectomy) and radiotherapy, when feasible (page 25). The recommendation for "aggressive treatment" is not supported as a primary strategy. While the guideline acknowledges that more aggressive amputative surgeries may have lower recurrence rates and should be discussed as an option, its primary focus is on penile preservation for T1/T2 disease, weighing oncological outcomes against functional and quality of life impacts (page 20). |
| ESMO2024 | R | The guideline supports the recommendation for aggressive treatment of T2 penile cancer. It specifies that for T2 tumours on the glans penis, "Glansectomy, with or without distal urethrectomy, is the surgical treatment of choice." Furthermore, it notes that partial or total penectomy are valid alternatives when necessary. These surgical interventions, aimed at complete tumour removal, align with the concept of "aggressive treatment." |
| NCCN2025 | R | The NCCN guidelines align with the recommendation for aggressive treatment of Stage T2 penile cancer. The "Primary Treatment T1, T2 or Greater" algorithm (PN-7) specifies that for "T2 or greater" tumors, the primary treatment options are partial penectomy, total penectomy, radiotherapy (RT), or chemo/RT. The discussion section further supports this, stating that T≥2 lesions "typically require more extensive surgical intervention with partial or total penectomy" (MS-8). These surgical and radiotherapeutic options are considered aggressive forms of treatment. |

**15.19 For localized penile cancer in kidney transplant recipients, the surgical treatment should be consistent with established guidelines for the general population.**

[5]

| AUA2020 | NA | The guideline provides a comprehensive overview of surgical treatments for early-stage penile cancer, such as circumcision, tumor excision, Mohs surgery, and glansectomy. However, it does not specifically address the management of kidney transplant recipients or patients on immunosuppression in the context of surgical treatment. While the guideline briefly mentions transplant recipients when discussing the safety of topical agents, it makes no mention of this specific patient population in its discussion of surgical approaches. |
| --- | --- | --- |
| DGU2020 | NA | The guideline provides comprehensive surgical treatment recommendations for localized penile cancer based on tumor characteristics such as stage and location. However, the document does not address or contain any specific guidance for the treatment of special patient populations, such as kidney transplant recipients or other immunocompromised individuals. Since this specific patient group is not mentioned, the guideline does not take a stance on the proposed recommendation. |
| EAU-ASCO2025 | DR | The treatment recommendation suggests that surgical treatment for localized penile cancer in kidney transplant recipients should be the same as for the general population. The EAU-ASCO guideline, however, introduces a specific consideration for this patient group. In section 6.1.2.7 on Glansectomy, it states that patients with "immunosuppression... are less suitable for graft application due to higher failure rates." This implies that while the general treatment principles may apply, the choice of a specific surgical technique (like glansectomy with grafting) must be approached with more caution in immunosuppressed patients, such as transplant recipients, which is a different and more nuanced approach than a standard application of general guidelines. |
| ESMO2024 | NA | The ESMO-EURACAN guideline provides a comprehensive framework for the diagnosis, treatment, and follow-up of penile cancer, with recommendations stratified by disease stage (local, locoregional, advanced/metastatic). However, the guideline does not mention or discuss specific patient populations, such as kidney transplant recipients or other immunosuppressed individuals. Consequently, it offers no specific advice or modifications for managing penile cancer in this particular clinical context. |
| NCCN2025 | NA | The guideline provides a comprehensive framework for the surgical management of localized penile cancer based on tumor stage (T1, T2), grade, and other pathologic features. However, the document does not specifically mention kidney transplant recipients, organ transplant recipients, or immunosuppression as factors that would modify these surgical recommendations. Because the guideline does not provide any distinct advice or considerations for this specific patient population, the recommendation is not addressed. |

**16. Prognostic Assessment**

**16.1 Testing of the tumour tissue for p53 expression is suggested to be useful for clinical planning to stratify patients and help inform management and follow-up strategy.**

[28]; [21]

| AUA2020 | NA | The AUA Update Series guideline provides a comprehensive overview of the diagnosis and treatment of early-stage squamous cell carcinoma of the penis. It bases treatment recommendations on factors such as clinical stage (Tis, Ta, T1), tumor grade, location, and histopathological features like pattern of invasion. The document does not mention or discuss the use of p53 expression testing or any other molecular biomarkers for stratifying patients or informing clinical management strategies. |
| --- | --- | --- |
| DGU2020 | DR | The guideline explicitly states that there are currently no molecular biological markers that can be recommended for the routine diagnosis of penile carcinoma (Statement 4.9). Although the background text acknowledges that several studies have associated abnormal p53 expression with poorer overall survival and tumor relapses, this information does not culminate in a clinical recommendation. Therefore, the guideline's conclusion is contrary to the treatment recommendation, which suggests using p53 expression to inform management and follow-up strategies. |
| EAU-ASCO2025 | DR | The guideline does not recommend testing for p53 expression as part of the standard pathological workup for clinical planning. Instead, the guideline gives a strong recommendation that "The pathological evaluation of penile carcinoma specimens must include an assessment of p16 by immunohistochemistry" (page 13). While the guideline acknowledges that strong p53 immunoreactivity has been reported as a predictor of pelvic lymph node involvement (page 29), it is not included in the list of required information for pathology reports (Table 3.3) or in the formal recommendations for pathological assessment. |
| ESMO2024 | NA | The guideline provides detailed recommendations for the pathological assessment of penile cancer, including HPV status, histological grade, and tumour type. It also discusses molecular profiling for advanced disease, focusing on biomarkers like TMB, MSI-H, dMMR, and PD-L1 to guide immunotherapy. However, the guideline does not mention testing for p53 expression as a tool for stratifying patients or informing clinical management and follow-up strategies. |
| NCCN2025 | NA | The recommendation suggests testing for p53 expression to help stratify patients and plan treatment. The NCCN guideline outlines numerous prognostic factors for consideration, including histologic grade, depth of invasion, lymphovascular invasion, perineural invasion, and HPV/p16 status (PN-F, MS-4). However, the guideline makes no mention of testing for p53 expression as a prognostic marker or for guiding clinical management and follow-up strategies. |

**17. Reconstructive Surgery**

**17.1 For patients undergoing glansectomy who desire a neoglans, a split-thickness skin graft (SSG) can be used for reconstruction.**

[16]

| AUA2020 | R | The guideline supports the use of a split-thickness skin graft for reconstruction following glansectomy. It explicitly discusses "total and partial glansectomy" as a surgical approach that has "gained interest" for distally located penile cancers. Furthermore, the guideline includes a figure (Figure 1) titled "Partial glansectomy with concomitant partial thickness skin grafting," directly illustrating the recommended procedure as a valid treatment option. |
| --- | --- | --- |
| DGU2020 | R | The guideline aligns with the treatment recommendation for neoglans reconstruction. Section 7.1.3.3 on page 53 explicitly states that glans reconstruction can be performed with free skin grafts, "üblicherweise als 'split thickness'-Transplantat" (usually as a 'split thickness' transplant). This directly corresponds to the recommendation, confirming that a split-thickness skin graft is a standard and appropriate method for this procedure. |
| EAU-ASCO2025 | R | The guideline aligns with the treatment recommendation. Section 6.1.2.7, "Glansectomy," explicitly states that "A split-thickness skin graft is commonly used to reconstruct a neo-glans." This directly supports the recommendation's suggestion of using a split-thickness skin graft (SSG) for neoglans reconstruction in patients undergoing glansectomy. |
| ESMO2024 | R | The guideline supports the use of a split-thickness skin graft (SSG) for neoglans reconstruction following glansectomy. In the section on "T2 disease," the guideline states, "An SSG is recommended to reconstruct a neo-glans from the preserved distal corporal tips." Furthermore, in the formal "Recommendations" for "T2-4 disease," the guideline recommends "glansectomy with or without distal urethrectomy and SSG reconstruction." |
| NCCN2025 | R | The NCCN guideline recommends the use of a split-thickness skin graft (STSG) for neoglans reconstruction following a glansectomy. In the "Principles of Penile Organ-Sparing Approaches" section (PN-A), the guideline states that for glansectomy, "Treatment is followed in certain instances with an STSG or FTSG to create a neoglans." This statement directly aligns with the proposed treatment recommendation. |

**18. Salvage Treatment**

**18.1 Patients who experience a local recurrence or are found to have positive surgical margins after initial organ-sparing surgery can be offered a salvage penectomy.**

[4]

| AUA2020 | R | The guideline supports the recommendation to offer salvage penectomy for recurrence or positive margins after organ-sparing surgery. In the "SURGERY" section, the text explicitly states, "More aggressive surgical approaches such as partial and total penectomy are acceptable options for patients with unfavorable histopathology or recurrent disease." Furthermore, the treatment algorithm in Figure 4 lists "Partial penectomy" and "Radical penectomy" as valid treatment options for a "Recurrent lesion at the same site." |
| --- | --- | --- |
| DGU2020 | DR | The guideline recommends a different approach than the one proposed. For local recurrence after organ-sparing surgery, the guideline states it should be treated according to the same principles as a primary tumor, meaning organ-sparing strategies can and should be considered again (Empfehlung 7.25, p. 61). Similarly, for positive surgical margins, the guideline recommends a "secondary operation" (Empfehlung 7.18, p. 54), which implies a re-excision to achieve clear margins rather than an immediate penectomy. While salvage penectomy is an option for treatment failure, the guideline prioritizes attempting less radical, organ-sparing approaches first, in contrast to the recommendation's direct suggestion of penectomy. |
| EAU-ASCO2025 | R | The guideline supports the recommendation to offer salvage penectomy for local recurrence after initial organ-sparing surgery. Section 6.1.4, "Local recurrence after organ-sparing surgery," explicitly states that for "large or high-stage recurrence, partial or total amputation is required" (page 24). This is further reinforced in the recommendations table on page 25, which advises to "Offer amputative surgery to patients with large local recurrences or corpora cavernosa involvement." Although the guideline also suggests salvage organ-sparing surgery for smaller recurrences, it clearly recommends penectomy (amputation) as a valid and necessary salvage option in specific scenarios, aligning with the provided recommendation. |
| ESMO2024 | R | The guideline supports the recommendation to offer a salvage penectomy for local recurrence or positive surgical margins. In the section on "T2 disease," the guideline notes that the incidence of "salvage penectomy for positive margins and/or recurrence is 1.2%-8.3%" and that partial or total penectomy are valid alternatives. Furthermore, under "Recurrent disease," the guideline explicitly states that recurrent disease involving the corpus cavernosum warrants a partial or total penectomy. |
| NCCN2025 | R | The guideline supports the recommendation. The flowchart for "Management of Recurrent Disease" (page PN-9) directs that a "Recurrence of penile lesion after initial penile sparing treatment" should be treated according to the recurrence stage, as detailed on pages PN-1 and PN-2. The treatment algorithm on page PN-2 lists partial and total penectomy as standard options for tumors T1b or greater. Furthermore, the discussion section on glansectomy outcomes explicitly mentions the incidence of "salvage penectomy due to positive margins and/or recurrence" (page MS-7). |

**19. Screening**

**19.1 General screening protocols for penile cancer are not recommended in kidney transplant recipients.**

[5]

| AUA2020 | NA | The AUA guideline focuses on the epidemiology, diagnosis, and treatment options for existing early-stage penile cancer. The treatment recommendation, however, addresses the specific topic of general screening protocols for penile cancer in kidney transplant recipients. The provided guideline does not contain any information or recommendations about screening for penile cancer in any patient population. |
| --- | --- | --- |
| DGU2020 | R | The guideline explicitly states that there is no special screening examination ("Vorsorgeuntersuchung") for penile cancer in general. This aligns with the treatment recommendation, which advises against general screening protocols for a specific patient population (kidney transplant recipients). Since the guideline does not recommend general screening for any group, it supports the recommendation to not screen this specific sub-group. |
| EAU-ASCO2025 | NA | The EAU-ASCO guideline does not address the topic of general screening protocols for penile cancer. The document makes no mention of screening asymptomatic individuals in any population, nor does it specifically discuss kidney transplant recipients or immunosuppressed individuals as a high-risk group that would warrant a screening program. The guideline's focus is on risk factors for disease development (e.g., HPV, phimosis), and the diagnosis and staging of patients who already present with a lesion. |
| ESMO2024 | NA | The guideline does not address the topic of general screening protocols for penile cancer in any population. The document focuses on the incidence, risk factors, and the diagnostic work-up for suspected cases, as well as treatment and follow-up for confirmed penile cancer. There is no mention of screening asymptomatic individuals, including high-risk groups like kidney transplant recipients. |
| NCCN2025 | NA | The NCCN guideline provides comprehensive recommendations for the diagnosis, staging, and treatment of patients with suspected or confirmed penile cancer. The document details risk factors such as HPV, HIV, phimosis, and tobacco use, but it does not mention kidney transplant recipients as a specific risk group. The guideline does not address the topic of screening for penile cancer in either the general population or in specific high-risk cohorts, and therefore does not offer a stance on screening protocols for kidney transplant recipients. |

**20. Surgical Margins**

**20.1 For penile-preserving surgery, conservative surgical margins of 3-5 mm (or a few millimeters) are considered acceptable to achieve oncological control, a reduction from historical recommendations.**

[41]; [23]; [4]

| AUA2020 | R | The guideline supports the recommendation for smaller surgical margins. It explicitly states that "the previous goal of a 2 cm surgical margin is no longer applicable" and that a "margin of 5 mm is considered oncologically safe." This aligns directly with the recommendation's proposed 3-5 mm margin. The guideline further reinforces this by citing evidence that the maximum tumor spread was 5 mm for grade 1 and 2 tumors. |
| --- | --- | --- |
| DGU2020 | R | The guideline aligns with the treatment recommendation by advising a shift away from historical 2 cm surgical margins. Specifically, Recommendation 7.17 states that for organ-preserving surgery, a tumor-free margin of "at least one to a few millimeters" is sufficient. The background text further reinforces this, noting that a few millimeters are now considered oncologically safe, and cites studies [135, 174] concluding that margins as small as 5 mm are adequate. |
| EAU-ASCO2025 | R | The guideline supports the recommendation for conservative surgical margins. In section 6.1.2.1, "Width of negative surgical margins," the guideline discusses evidence supporting smaller margins, citing reports that an excision margin between 5 mm and 10 mm leads to low recurrence rates. It also notes another study where recurrence rates only increased when the margin was less than 1 mm. This evidence aligns with the recommendation that a conservative margin of 3-5 mm is acceptable for achieving oncological control in penile-preserving surgery. |
| ESMO2024 | DR | The treatment recommendation suggests conservative surgical margins of 3–5 mm for penile-preserving surgery. However, the ESMO-EURACAN guideline proposes an even more minimal margin. Specifically, in the section on "T2 disease," it states, "Surgical margins of >1 mm are now accepted, with the risk of local recurrence being low." Although both advocate for reduced margins compared to historical norms, the guideline recommends a different, smaller acceptable margin. |
| NCCN2025 | DR | The treatment recommendation suggests conservative surgical margins of 3-5 mm. The NCCN guideline, in the discussion section for Wide Local Excision (page MS-7), cites studies showing that surgical margins of 5 to 10 mm are as safe as historical 2-cm margins. Because the guideline's stated safe range of 5-10 mm differs from the 3-5 mm proposed in the recommendation, it supports a different, slightly larger margin. |

**20.2 When performing Wide Local Excision (WLE) for penile SCC, a 1 cm margin is often suggested to reduce recurrence.**

[42]

| AUA2020 | DR | The guideline suggests a different and more nuanced approach to surgical margins than the single 1 cm recommendation. It states that the previous goal of a 2 cm margin is no longer applicable and instead recommends margins based on tumor grade. Specifically, the guideline indicates a 5 mm (0.5 cm) margin for grade 1 and 2 tumors and a 10 mm (1 cm) margin for grade 3 tumors, making its recommendation different for lower-grade cancers. |
| --- | --- | --- |
| DGU2020 | DR | The guideline recommends a different surgical margin than the one proposed. While the recommendation suggests a 1 cm margin for Wide Local Excision, the guideline explicitly states that a margin of "at least one to a few millimeters" is sufficient (Recommendation 7.17). The background text reinforces this, noting that the previously propagated 2 cm margin is outdated and that multiple systematic reviews support the use of smaller, tumor-free margins of just a few millimeters to achieve oncological safety while preserving organ function. |
| EAU-ASCO2025 | R | The guideline supports the recommendation for surgical margins in wide local excision (WLE). Section 6.1.2.1, "Width of negative surgical margins," states that while there is no definitive consensus, reports show that an excision margin between 5 mm and 10 mm results in acceptably low recurrence rates. Since the recommendation suggests a 1 cm (10 mm) margin, it falls within the range considered effective by the guideline for achieving oncological control. |
| ESMO2024 | DR | The guideline recommends a different surgical margin than the proposed treatment. The recommendation suggests a 1 cm margin for Wide Local Excision (WLE). However, the guideline, in the section discussing T2 disease, states that "Surgical margins of >1 mm are now accepted, with the risk of local recurrence being low." This indicates a significant discrepancy, with the guideline supporting a much smaller margin to facilitate organ-sparing surgery. |
| NCCN2025 | R | The guideline supports the recommendation for a 1 cm surgical margin during Wide Local Excision (WLE). The Discussion section on page MS-7, under "Wide Local Excision," explicitly states that studies have demonstrated surgical margins of 5 to 10 mm (0.5 to 1 cm) are as safe as 2 cm margins. This aligns with the recommendation that a 1 cm (10 mm) margin is appropriate for reducing recurrence. |

**21. Surgical Technique**

**21.1 If tumors extend into or macroscopically involve the distal corporal tips during glansectomy, a distal corporectomy should be performed.**

[16]; [4]

| AUA2020 | NA | The guideline provides a general overview of surgical treatments for early-stage penile cancer, mentioning glansectomy as an option for tumors on the glans. It also states that more aggressive approaches like partial or total penectomy are acceptable for unfavorable or recurrent disease. However, the guideline does not address the specific intraoperative scenario described in the recommendation, where a tumor involves the distal corporal tips during glansectomy. Furthermore, the specific procedure of "distal corporectomy" is not mentioned in the text. |
| --- | --- | --- |
| DGU2020 | R | The treatment recommendation advises performing a distal corporectomy if the tumor extends into the distal corporal tips during a glansectomy. The guideline aligns with this by classifying tumors with invasion of the corpora cavernosa as T3 stage. For T3 tumors, the guideline recommends partial penile amputation as the therapy of choice (Recommendation 7.22, page 59). The background information on the same page explicitly mentions "distale Corporektomie" (distal corporectomy) as a surgical procedure for this stage, thus supporting the recommended surgical escalation. |
| EAU-ASCO2025 | R | The guideline supports the treatment recommendation. In Section 6.1.3.1, "Resectable disease," the guideline discusses the surgical approach for cT2 disease where there is doubt about corporeal invasion. It explicitly describes the procedure of "glansectomy combined with distal corporectomy" as a management option in this scenario. This directly aligns with the recommendation to perform a distal corporectomy if tumors involve the distal corporal tips during a glansectomy. |
| ESMO2024 | R | The guideline's proposed algorithm for the management of primary penile tumors (Figure 2) explicitly lists "Glansectomy with distal corporectomy [III, A]" as a recommended surgical option for T2-T4 tumors, which are defined by tunical or cavernosal involvement. The treatment recommendation describes this exact clinical scenario: performing a distal corporectomy when the tumor extends to involve the distal corporal tips. Therefore, the guideline directly supports the recommendation as a standard of care. |
| NCCN2025 | DR | The guideline does not mention "distal corporectomy" specifically. Instead, when there is tumor invasion into the corpora cavernosum, the NCCN guideline recommends a more extensive procedure. On page PN-B, under "Penectomy," it explicitly states, "Partial or total penectomy is necessary when there is invasion into the corpora cavernosum in order to achieve a negative margin." This indicates a shift from an organ-sparing approach (glansectomy) to a more formal amputative surgery (penectomy), which is different from the recommendation of adding a distal corporectomy to an ongoing glansectomy. |

**22. Systemic Therapy**

**22.1 A multimodal therapeutic approach is typically warranted for patients with lymph node involvement (locally advanced and advanced PSCC).**

[12]

| AUA2020 | NA | The AUA guideline focuses exclusively on the "Treatment of Early Stage Squamous Cell Carcinoma of the Penis," as stated in its title and learning objective. The document details therapies such as topical agents, laser ablation, and various organ-preserving surgeries for non-invasive and early-stage (Tis, Ta, T1) disease. The treatment recommendation, however, pertains to a different patient population: those with locally advanced and advanced disease with lymph node involvement. Since the guideline's scope is strictly limited to early-stage cancer, it does not address the multimodal therapeutic approaches required for advanced disease. |
| --- | --- | --- |
| DGU2020 | R | The guideline strongly supports the recommendation for a multimodal approach in patients with lymph node involvement. Section 7.2.3, specifically the background information for recommendations 7.43-7.45 on page 73, explicitly states that for metastatic penile cancer, "a curative therapy can only be achieved in a multimodal setting." The guideline further details this approach by recommending neoadjuvant chemotherapy for patients with fixed inguinal lymph nodes (Recommendation 7.44), which is then followed by surgery, confirming the multimodal strategy. |
| EAU-ASCO2025 | R | The guideline strongly aligns with the recommendation for a multimodal approach in patients with lymph node involvement. Section 6.4 is entirely dedicated to the "Role of multimodal chemotherapy/radiotherapy in the management of (regional) lymph nodes." The guideline specifically recommends neoadjuvant chemotherapy (NAC) followed by consolidative surgery for patients with extensive inguinal or pelvic lymph node involvement (cN3 disease), as stated in sections 6.3.2 and 6.4.1.1. This combination of systemic therapy and surgery exemplifies the multimodal strategy advocated in the recommendation. |
| ESMO2024 | R | The guideline supports the recommendation for a multimodal therapeutic approach for patients with lymph node involvement. The guideline explicitly states that for bulky or ulcerated nodal disease (cN3), "multimodal treatment" is required, recommending neoadjuvant chemotherapy (ChT) followed by surgery. It also advises that salvage inguinal lymph node dissection for recurrent disease should be part of a "multimodal treatment" including ChT. Furthermore, for high-risk node-positive disease (pN3), the guideline suggests considering adjuvant radiotherapy in combination with adjuvant ChT, reinforcing its support for using multiple treatment modalities. |
| NCCN2025 | R | The NCCN guideline consistently recommends a multimodal approach for penile cancer with lymph node involvement, which aligns with the treatment recommendation. For example, in the management of palpable bulky inguinal lymph nodes (PN-5), the guideline advises neoadjuvant chemotherapy followed by surgery (ILND/PLND), with potential for adjuvant chemotherapy or radiotherapy. The discussion section further supports this, stating that a patient with resectable bulky disease "will rarely be cured with a single treatment modality" and details various perioperative chemotherapy and radiotherapy options (MS-13). |

**22.2 For unresectable tumours or disseminated metastatic disease, cytotoxic chemotherapy is employed, which can be used in a neoadjuvant or adjuvant context.**

[38]

| AUA2020 | NA | The treatment recommendation addresses the use of cytotoxic chemotherapy for unresectable or disseminated metastatic penile cancer. The provided AUA guideline, however, is explicitly focused on the "Treatment of Early Stage Squamous Cell Carcinoma of the Penis," as stated in its title and learning objective. The guideline details topical therapies, laser therapy, and organ-preserving surgery for non-invasive and early-stage (Tis, Ta, T1) disease. While it briefly mentions systemic chemotherapy in a neoadjuvant context for one specific therapy, it does not provide any recommendations or discussion for the management of advanced, unresectable, or metastatic disease. |
| --- | --- | --- |
| DGU2020 | R | The guideline aligns with the recommendation to use chemotherapy for unresectable or metastatic disease. Specifically, it recommends neoadjuvant chemotherapy for patients with unresectable, fixed inguinal lymph nodes to improve outcomes (Recommendation 7.44). For disseminated metastatic disease, it recommends palliative combination chemotherapy to prolong life and control symptoms in patients with good general condition (Recommendation 7.53). The guideline also discusses the use of adjuvant chemotherapy in high-risk scenarios, thus supporting all aspects of the proposed treatment. |
| EAU-ASCO2025 | R | The guideline aligns with the recommendation to use cytotoxic chemotherapy for unresectable and metastatic penile cancer. Section 6.1.3.2 specifically discusses using induction chemotherapy for non-resectable disease to downstage tumors and enable surgical resection. Furthermore, Section 6.5 recommends platinum-based chemotherapy as the preferred first-line approach for patients with distant metastatic disease. The guideline also provides detailed recommendations for using chemotherapy in both neoadjuvant (Section 6.4.1.1) and adjuvant (Section 6.4.1.2) contexts, fully supporting all aspects of the proposed treatment. |
| ESMO2024 | R | The guideline aligns with the recommendation, stating that cytotoxic chemotherapy (ChT) is used for patients with metastatic, inoperable penile cancer. The document explicitly discusses and recommends neoadjuvant ChT for locally advanced, inoperable primary tumors or fixed regional nodes (cN3). It also recommends adjuvant ChT for patients with high-risk node-positive disease following surgery and palliative ChT for metastatic disease. |
| NCCN2025 | R | The NCCN guideline aligns with the recommendation by advocating for systemic chemotherapy in cases of metastatic disease and for unresectable tumors. Page PN-10, "Management of Metastatic Disease," clearly indicates "Systemic chemotherapy" as the initial step for metastatic penile cancer. Furthermore, the guideline details the use of chemotherapy in both the neoadjuvant and adjuvant settings for advanced disease (Page PN-D, 1 of 5), including the use of neoadjuvant chemotherapy to downstage initially unresectable (T4) primary tumors. |

**23. Systemic Therapy (Adjuvant)**

**23.1 Adjuvant chemotherapy (ACT) should be considered for patients with pathologically confirmed pN2-3 disease, as it may be of benefit, particularly for those with pN3 or pelvic lymph node metastases.**

[1]; [44]

| AUA2020 | NA | The AUA Update Series guideline focuses explicitly on the "Treatment of Early Stage Squamous Cell Carcinoma of the Penis," detailing management for non-invasive and early-stage disease (carcinoma in situ, Ta, and T1). The treatment recommendation, however, pertains to adjuvant chemotherapy for patients with advanced nodal disease (pN2-3), a stage beyond the scope of this guideline. While the document briefly mentions systemic chemotherapy in the context of neoadjuvant therapy combined with laser treatment, it does not address the use of adjuvant chemotherapy following lymph node dissection for pathologically confirmed pN2-3 disease. |
| --- | --- | --- |
| DGU2020 | R | The guideline aligns with the treatment recommendation. Recommendation 7.37 on page 69 explicitly states that adjuvant chemotherapy can be administered to patients with pN2/pN3 disease. The accompanying background information on page 71 further elaborates that while randomized controlled trials are lacking, the existing data supports a recommendation for adjuvant chemotherapy in patients with a pN2/pN3 status. This is consistent with the proposal to consider ACT for this specific patient group. |
| EAU-ASCO2025 | R | The guideline aligns with the recommendation to consider adjuvant chemotherapy (ACT) for high-risk patients. It explicitly states, "the benefit from adjuvant chemotherapy is expected to be highest in the population of patients with pathological N3 stage, in particular for those patients with pelvic nodal involvement" (page 32). Although noting the limited data for improving overall survival, the guideline recommends a "balanced discussion of risks and benefits of adjuvant chemotherapy with high-risk patients...in particular, with those with pathological pelvic LN involvement (pN3)" (page 33). This supports the recommendation's stance on considering ACT for pN2-3 disease, with a particular focus on pN3. |
| ESMO2024 | R | The guideline explicitly recommends that "Patients with pN2 and pN3 disease following LND should be considered for adjuvant ChT following surgery [III, B]." This statement is directly aligned with the treatment recommendation to consider adjuvant chemotherapy for patients with pathologically confirmed pN2-3 disease. Furthermore, the guideline text discusses that while evidence is limited, studies have shown overall survival improvements in the highest-risk patients, including those with pelvic lymph node involvement, which supports the recommendation's specific mention of pN3 and pelvic LN metastases. |
| NCCN2025 | R | The NCCN guideline aligns with the recommendation to consider adjuvant chemotherapy for pN2-3 disease. The guideline algorithm for "Management of Palpable Non-Bulky Inguinal Lymph Nodes" (PN-4) lists adjuvant chemotherapy as a treatment option for patients with pN2-3 disease. Furthermore, the "Principles of Systemic Therapy" (PN-D, 1 of 5) states that it is reasonable to administer adjuvant chemotherapy when pathology shows high-risk features, which include pelvic lymph node metastases (part of the pN3 definition). The discussion section (MS-14) also explicitly states that treatment for patients with pN2 or pN3 disease may entail adjuvant chemotherapy. |

**23.2 Adjuvant radiotherapy (ART) should be considered after lymph node dissection for patients with pN2-3 disease, as it may reduce recurrence and improve survival.**

[1]; [29]

| AUA2020 | NA | The guideline focuses exclusively on the "Treatment of Early Stage Squamous Cell Carcinoma of the Penis," as stated in its title and learning objective. The provided text details management for localized tumors (Tis, Ta, T1) using topical therapies, laser ablation, and various surgical techniques. The recommendation, however, addresses adjuvant radiotherapy for patients with advanced nodal disease (pN2-3) after lymph node dissection, a clinical scenario that falls outside the scope of this early-stage disease guideline. |
| --- | --- | --- |
| DGU2020 | DR | The guideline does not generally recommend adjuvant radiotherapy after lymph node dissection due to contradictory and insufficient evidence. Recommendation 7.42 states that adjuvant radiotherapy "cannot be generally recommended" but "can be considered" for high-risk patients, which includes those with pN2-3 disease. However, the guideline's primary recommendation for this patient group is adjuvant chemotherapy (Recommendations 7.37 and 7.39). Therefore, the guideline suggests a different primary adjuvant treatment approach than the one proposed in the recommendation. |
| EAU-ASCO2025 | R | The guideline recommends offering adjuvant radiotherapy to patients with pN2/N3 disease, which aligns directly with the treatment recommendation. Specifically, in the recommendations table on page 35, the guideline states, "Offer adjuvant radiotherapy (with or without chemo sensitisation) to patients with pN2/N3 disease...". Although the strength of this recommendation is rated as "Weak," the guideline's discussion in section 6.4.2.2 reviews evidence suggesting that adjuvant radiotherapy can improve overall and disease-specific survival in this patient population, supporting the rationale presented. |
| ESMO2024 | R | The guideline supports the consideration of adjuvant radiotherapy (ART) for patients with pN2-3 disease, aligning with the treatment recommendation. The text notes that while the role of ART is controversial, there is "positive evidence reported supporting the use of adjuvant RT in high-risk node-positive disease." The guideline specifically cites studies demonstrating an overall survival benefit driven by patients with N2 disease and improved survival with adjuvant chemoradiotherapy in pN3 disease. The final recommendation is for clinicians to "consider the use of adjuvant RT... in combination with adjuvant ChT in patients with pN3 disease." |
| NCCN2025 | R | The guideline aligns with the recommendation to consider adjuvant radiotherapy (ART) for pN2-3 disease. In the "Principles of Radiotherapy" section (PN-C), ART is listed as a consideration for "palpable non-bulky inguinal lymph nodes pN2–3 disease (category 2B)." Similarly, the treatment algorithm for palpable non-bulky inguinal lymph nodes (PN-4) includes pathways for pN2-3 disease that involve adjuvant RT, either alone or with chemotherapy. The discussion section further reinforces this, stating for pN2-3 disease, "a PLND with or without adjuvant RT... is recommended" (MS-15). |

**23.3 Adjuvant Chemoradiotherapy (ACRT) may provide a modest additional benefit in reducing locoregional recurrence and improving survival in pN3 disease, especially with extranodal extension (ENE) or HPV positivity.**

[1]

| AUA2020 | NA | The AUA guideline focuses exclusively on the "Treatment of Early Stage Squamous Cell Carcinoma of the Penis," covering stages like carcinoma in situ, Ta, and T1. The treatment recommendation, however, pertains to adjuvant chemoradiotherapy for pN3 disease, which signifies advanced regional lymph node metastasis and falls outside the scope of early-stage cancer. The guideline does not discuss the management of advanced nodal disease or the role of adjuvant therapies in that specific clinical context. |
| --- | --- | --- |
| DGU2020 | DR | The guideline recommends adjuvant chemotherapy for patients with pN2/pN3 disease (Recommendation 7.37). However, for adjuvant radiotherapy, it explicitly states that it cannot be generally recommended due to insufficient and contradictory evidence (Recommendation 7.42, Statement 7.41). While radiotherapy can be considered in high-risk situations like extranodal extension, the guideline discusses these treatments as separate modalities and does not recommend their combination as a standard Adjuvant Chemoradiotherapy (ACRT) regimen. Therefore, the guideline's primary recommendation is for chemotherapy alone, differing from the proposed combined treatment. |
| EAU-ASCO2025 | R | The guideline supports the use of adjuvant chemoradiotherapy for high-risk disease. It explicitly recommends to "Offer adjuvant radiotherapy (with or without chemo sensitisation) to patients with pN2/N3 disease" (page 35). The document also discusses evidence suggesting that HPV status could be a selection factor for adjuvant radiotherapy, noting that HPV-positive patients appeared to have increased overall survival (page 34). Furthermore, it reviews data showing improved outcomes with adjuvant chemoradiotherapy for patients with high-risk features like extranodal extension (ENE) and pN3 disease. |
| ESMO2024 | R | The guideline supports the use of adjuvant chemoradiotherapy (ACRT) for pN3 disease. It specifically recommends that clinicians "may consider the use of adjuvant RT... in combination with adjuvant ChT in patients with pN3 disease" [III, B]. The guideline also discusses evidence showing that adjuvant CRT significantly improved survival in patients with pN3 disease and extracapsular extension, and that HPV-positive patients receiving post-operative RT had a significantly longer overall survival, aligning with all key aspects of the recommendation. |
| NCCN2025 | R | The NCCN guideline supports the use of adjuvant chemoradiotherapy (ACRT) for patients with pN3 disease. On multiple algorithmic pages (eg, PN-4, PN-9), "Chemo/RT" is listed as a category 2B option for patients with pN2-3 disease after inguinal lymph node dissection (ILND). The discussion (MS-14) further clarifies that "Chemoradiotherapy can be considered for patients with high-risk features including...extranodal extension" and that for pN2 or pN3 disease, treatment may entail adjuvant chemoradiotherapy. This directly aligns with the recommendation to consider ACRT for pN3 disease, especially with extranodal extension (ENE). |

**23.4 (Neo)adjuvant treatment (chemotherapy or radiotherapy) is not recommended for pN1 disease as it has no proven survival benefit.**

[1]

| AUA2020 | NA | The guideline, "Treatment of Early Stage Squamous Cell Carcinoma of the Penis," focuses exclusively on the management of primary tumors, specifically non-invasive and early-stage malignancies (carcinoma in situ, Ta, and T1). It details topical therapies, laser ablation, and various surgical options for the primary lesion. The document does not address the management of pathologically-confirmed lymph node metastasis (pN1 disease), which represents a more advanced stage of cancer beyond the scope of this guideline. Therefore, the recommendation against using (neo)adjuvant chemotherapy or radiotherapy for pN1 disease is not mentioned or discussed. |
| --- | --- | --- |
| DGU2020 | DR | The treatment recommendation states that (neo)adjuvant treatment is not recommended for any pN1 disease. In contrast, the guideline offers a more nuanced approach. Recommendation 7.38 on page 69 specifies that adjuvant chemotherapy can be considered ("kann ... in Erwägung gezogen werden") for pN1 patients who have high-risk features, such as an aggressive tumor (≥ G2) and/or a large lymph node metastasis (≥ 3cm). Therefore, while the guideline does not recommend adjuvant therapy for all pN1 cases, it explicitly identifies a subgroup for whom it is an option, which differs from the blanket non-recommendation. |
| EAU-ASCO2025 | R | The guideline aligns with the recommendation by consistently reserving neoadjuvant and adjuvant therapies for higher-stage nodal disease and not for pN1. Section 6.2.2.1 of the guideline states that radical inguinal lymph node dissection (rILND) is curative for low-volume disease (pN1), implying that additional treatment is not required. Furthermore, the recommendations for neoadjuvant chemotherapy, adjuvant chemotherapy, and adjuvant radiotherapy are explicitly for patients with pN2 or pN3 disease (Sections 6.4.1.3 and 6.4.2.3). The absence of a recommendation for (neo)adjuvant therapy in pN1 patients supports the provided statement. |
| ESMO2024 | R | The guideline aligns with the recommendation to not use (neo)adjuvant therapy for pN1 disease. In its discussion of adjuvant radiotherapy, the guideline cites a study from the US National Cancer Database which demonstrated an overall survival benefit for patients with N2 disease, but this benefit "was absent in patients presenting with N1 disease." The guideline's specific recommendations for adjuvant chemotherapy and radiotherapy are for higher-risk disease stages (pN2 and pN3), implicitly excluding pN1. |
| NCCN2025 | R | The NCCN guideline aligns with the recommendation to not use adjuvant therapy for pN1 disease. The treatment algorithm for palpable, non-bulky inguinal lymph nodes (PN-4) shows that after a positive biopsy leading to a pN1 finding, the recommended treatment is bilateral inguinal lymph node dissection (ILND), followed by surveillance. Adjuvant therapies (chemotherapy, RT, or chemo/RT) are only recommended for more advanced pN2-3 disease or in the presence of high-risk features, which are not characteristic of pN1 disease. |

**23.5 Adjuvant Radiotherapy (RT) following Pelvic Lymph Node Dissection (PLND) is not recommended as a routine practice and should be restricted to prospective controlled clinical trials.**

[34]

| AUA2020 | NA | The guideline focuses exclusively on the treatment of "early stage squamous cell carcinoma of the penis," covering management of the primary tumor through topical therapies, laser ablation, and organ-preserving surgery. The recommendation, however, pertains to adjuvant radiotherapy following Pelvic Lymph Node Dissection (PLND), which is a treatment for more advanced, node-positive disease. As the provided guideline does not discuss the management of advanced nodal disease, it does not address this specific recommendation. |
| --- | --- | --- |
| DGU2020 | R | The guideline aligns with the treatment recommendation by stating that adjuvant radiotherapy after lymph node dissection cannot be generally recommended (Empfehlung 7.42). The rationale provided is the lack of sufficient evidence, with Statement 7.41 noting that the value of postoperative radiotherapy has not been adequately investigated in studies. While the guideline suggests that radiotherapy can be considered for high-risk patients, this nuance does not contradict the core position that it is not a routine practice, thus supporting the recommendation's cautious stance. |
| EAU-ASCO2025 | DR | The treatment recommendation states that adjuvant radiotherapy (RT) following Pelvic Lymph Node Dissection (PLND) is not recommended for routine practice and should be restricted to clinical trials. The guideline acknowledges that adjuvant RT is "controversial" and was "not recommended in prior guidelines" due to a lack of level 1 evidence (p. 33). However, it proceeds to cite evidence suggesting a benefit, such as prolonging Disease-Specific Survival (DSS) in patients with positive pelvic lymph nodes (p. 34). Ultimately, the guideline makes a direct recommendation to "Offer adjuvant radiotherapy... to patients with pN2/N3 disease" (p. 35), thus proposing its use in specific clinical scenarios rather than restricting it solely to trials. |
| ESMO2024 | DR | The treatment recommendation advises against the routine use of adjuvant RT following PLND. The guideline, however, presents a more nuanced view. While acknowledging that the role of adjuvant RT is controversial and lacks prospective data, it cites studies showing an overall survival benefit for patients with positive pelvic LNs after PLND. Based on this evidence, the guideline concludes that adjuvant RT "remains a reasonable approach" for high-risk, node-positive patients and specifically recommends that clinicians "may consider the use of adjuvant RT" for patients with pN3 disease. |
| NCCN2025 | DR | The guideline recommends a different approach than the one proposed. The NCCN guideline explicitly states that "adjuvant RT is recommended following a PLND after a positive result on ILND" (MS-14). This is further supported by the treatment algorithm for palpable non-bulky inguinal lymph nodes (PN-4), which includes "adjuvant RT" as a treatment option following pelvic lymph node dissection (PLND) if pelvic nodes are positive. This directly contradicts the recommendation that adjuvant RT following PLND should not be a routine practice. |

**24. Systemic Therapy (First-Line Advanced/Metastatic)**

**24.1 For advanced, locally advanced, or metastatic disease, first-line systemic therapy typically involves platinum-based chemotherapy, such as the TIP regimen (paclitaxel, ifosfamide, and cisplatin).**

[45]; [3]

| AUA2020 | NA | The treatment recommendation specifies a systemic chemotherapy regimen (TIP) for advanced, locally advanced, or metastatic penile cancer. The provided AUA guideline, however, explicitly focuses on the "Treatment of Early Stage Squamous Cell Carcinoma of the Penis," as stated in its title and learning objective. The document details management for non-invasive and early-stage disease (Tis, Ta, T1) using topical therapies, laser, and organ-preserving surgery, but it does not address treatment strategies for advanced or metastatic disease. |
| --- | --- | --- |
| DGU2020 | R | The guideline recommends platinum-based chemotherapy for advanced or metastatic penile cancer. Specifically, recommendation 7.54 states that chemotherapy should consist of cisplatin, ifosfamide, and paclitaxel, which is the TIP regimen mentioned in the treatment recommendation. Furthermore, recommendation 7.47 also advises neoadjuvant polychemotherapy with paclitaxel, ifosfamide, and cisplatin for locally advanced disease, confirming a direct alignment. |
| EAU-ASCO2025 | R | The guideline aligns with the recommendation by identifying platinum-based chemotherapy as the preferred first-line approach for advanced disease. Section 6.5.2 explicitly supports "platinum-based chemotherapy as the preferred approach to first-line palliative systemic therapy" and lists triplet regimens like TIP (paclitaxel, ifosfamide, and cisplatin) as a choice. Furthermore, Section 6.4.1.1 discusses a phase II trial of neoadjuvant TIP for locally advanced (N2 or N3) disease, noting an objective response rate of 50%. The formal recommendations also advise offering platinum-based chemotherapy for patients with distant metastatic disease. |
| ESMO2024 | R | The guideline recommends first-line, cisplatin-based combination chemotherapy for the treatment of metastatic penile cancer. It explicitly states that "TIP [paclitaxel, ifosfamide, and cisplatin] and TPF are reasonable choices for first-line systemic therapy in patients considered fit enough." Furthermore, for locally advanced disease with fixed lymph nodes (cN3), the guideline recommends considering neoadjuvant chemotherapy with triplet regimens such as TIP or TPF. This aligns directly with the treatment recommendation. |
| NCCN2025 | R | The NCCN guideline aligns directly with the treatment recommendation. In the "Principles of Systemic Therapy" section, under "First-Line Systemic Therapy for Metastatic/Recurrent Disease," the guideline lists the TIP regimen (paclitaxel, ifosfamide, and cisplatin) as the "Preferred Regimen" (PN-D 2 of 5). The discussion section further reinforces this, stating that "Cisplatin-based regimens...[TIP]...are the most active first-line systemic chemotherapy regimens" and that the panel "recommends first-line chemotherapy using TIP (preferred)" for metastatic penile cancer (MS-17). |

**25. Systemic Therapy (Investigational)**

**25.1 The combination of Nivolumab and Ipilimumab appears unpromising for patients with PeCa.**

[38]

| AUA2020 | NA | , laser therapy, surgical excision, and radiation. The document makes no mention of checkpoint inhibitors like Nivolumab or Ipilimumab, which are typically considered for more advanced or metastatic disease. |
| --- | --- | --- |
| DGU2020 | NA | , it states that for therapy with checkpoint inhibitors ("Checkpointinhibitoren"), there are no study results available yet ("noch keine Studienergebnisse"). While it mentions the possibility of immunotherapy based on microsatellite status, it does not mention specific drugs like Nivolumab or Ipilimumab, nor does it comment on whether they are promising or unpromising. |
| EAU-ASCO2025 | R | The guideline addresses the use of combination immunotherapy in penile cancer. Specifically, in section 6.5.2.1, it cites a study testing the combination of ipilimumab and nivolumab where, of the five penile SCC patients included, only two achieved stable disease (SD). These modest results, with no reported partial or complete responses, align with the recommendation's assessment that this combination appears "unpromising" for this patient population. |
| ESMO2024 | NA | The guideline discusses immunotherapy and various immune checkpoint inhibitors (ICIs) for penile cancer, including cemiplimab, atezolizumab, and pembrolizumab, particularly for biomarker-selected patients (e.g., high TMB, MSI-H). However, the guideline does not specifically mention or evaluate the combination of nivolumab and ipilimumab. Therefore, it does not offer a stance on this particular treatment combination. |
| NCCN2025 | NA | The NCCN guideline for penile cancer provides recommendations for systemic therapy, including the use of the immune checkpoint inhibitor pembrolizumab in first-line and subsequent-line settings. However, the guideline does not mention or evaluate the specific combination of Nivolumab and Ipilimumab. Therefore, it takes no stance on whether this particular dual-immunotherapy regimen is promising or unpromising for treating penile cancer. |

**26. Systemic Therapy (Neoadjuvant)**

**26.1 Neoadjuvant chemotherapy (NACT) is recommended for patients with fixed, bulky, or advanced nodal disease (cN2-3), particularly when not immediately resectable, with the goal of downstaging to enable subsequent surgical consolidation.**

[1]; [44]; [29]

| AUA2020 | NA | The treatment recommendation addresses the use of neoadjuvant chemotherapy for patients with advanced nodal disease (cN2-3). The provided AUA guideline, however, explicitly states its focus is on the "Treatment of Early Stage Squamous Cell Carcinoma of the Penis." The document's content, including discussions on topical therapies, laser treatments, and organ-sparing surgeries, is consistently limited to early/low-stage disease (Tis, Ta, T1). Consequently, the management of advanced, bulky nodal disease as described in the recommendation is outside the scope of this guideline and is not addressed. |
| --- | --- | --- |
| DGU2020 | R | The guideline strongly aligns with the treatment recommendation. Recommendation 7.44 states that neoadjuvant chemotherapy "soll" (should) be performed for patients with fixed inguinal lymph nodes and good general condition. The background information on page 73 further specifies that neoadjuvant chemotherapy is indicated for patients with large, fixed, or non-resectable lymph node metastases to enable subsequent curative surgery. This directly corresponds to the recommendation's indication for fixed, bulky, or advanced nodal disease (cN2-3) with the goal of downstaging for surgical consolidation. |
| EAU-ASCO2025 | R | The guideline recommends neoadjuvant chemotherapy (NACT) for patients with advanced nodal disease. Section 6.3.2 identifies NACT as a "reasonable strategy among cN3 patients" to enable subsequent surgical resection. Furthermore, the recommendations table on page 33 explicitly states to "Offer neoadjuvant chemotherapy... to... patients with ... extensive inguinal involvement (cN3), in preference to up-front surgery" and as an alternative for "bulky mobile inguinal nodes or bilateral disease (cN2)". This aligns perfectly with the proposed recommendation to use NACT for cN2-3 disease with the goal of downstaging for surgery. |
| ESMO2024 | R | The guideline strongly supports the use of neoadjuvant chemotherapy (ChT) for advanced nodal disease. In the "Management of cN3 disease" section, it states that "Neoadjuvant chemotherapy (ChT) followed by ipsilateral radical inguinal and pelvic lymphadenectomy in responders is recommended." Furthermore, under the recommendations for systemic therapy, it specifies that "Patients with cN3 fixed nodes should be considered for neoadjuvant ChT with triplet regimens such as TIP or TPF [III, B]," which aligns directly with the proposed treatment for fixed, bulky nodal disease to enable surgical consolidation. |
| NCCN2025 | R | The NCCN guideline strongly supports the use of neoadjuvant chemotherapy for patients with advanced nodal disease. The "Management of Palpable Bulky Inguinal Lymph Nodes" algorithms (PN-5, PN-6) and the "Principles of Systemic Therapy" (PN-D, 1 of 5) explicitly recommend neoadjuvant TIP chemotherapy for patients with fixed, bilateral, or ≥4 cm inguinal lymph nodes (which aligns with cN2-3 disease). The guideline specifies that this treatment should be followed by consolidative surgery, such as ILND and/or PLND, which directly corresponds to the recommendation's goal of downstaging to enable surgical consolidation. |

**26.2 Neoadjuvant Chemotherapy (NC) can be utilized when initial local control is desired, with the specific goal of enabling a subsequent, less aggressive radical local surgery.**

[44]

| AUA2020 | DR | The guideline, which focuses on early-stage penile cancer, does not endorse the recommendation's strategy of using neoadjuvant chemotherapy to enable less aggressive surgery. While neoadjuvant chemotherapy is mentioned, it is only in the context of laser therapy for select cases. The guideline explicitly states that "a clear conclusion regarding the efficacy of neoadjuvant chemotherapy... cannot be drawn" and that "more data are required," characterizing its use as rare and its role as unelucidated. This contrasts with the recommendation, which presents it as a viable option for downstaging before surgery. |
| --- | --- | --- |
| DGU2020 | R | The guideline aligns with the recommendation, stating that for locally advanced tumors (T4), neoadjuvant chemotherapy should be considered (p. 73, Empfehlung 7.46). The background information further supports this, noting that for a non-resectable primary tumor, neoadjuvant chemotherapy is indicated and can improve local resectability (p. 60, p. 73). This directly supports the recommendation's goal of using chemotherapy for initial local control to enable a less aggressive subsequent surgery. |
| EAU-ASCO2025 | R | The guideline aligns with the treatment recommendation by supporting the use of induction (neoadjuvant) chemotherapy for specific cases of advanced penile cancer. Section 6.1.3.2 on "Non-resectable disease" explicitly states that "induction chemotherapy offers the ability to downstage disease and thereby enable surgical resection among responders" (page 23). This principle is further solidified in the recommendations table (page 25), which advises to "Offer induction chemotherapy followed by surgery to responders... for non-resectable advanced primary lesions." This directly corresponds to the recommendation's goal of using chemotherapy to achieve local control and enable subsequent surgery. |
| ESMO2024 | R | The guideline recommends discussing neoadjuvant chemotherapy (ChT) for patients with locally advanced (T4), inoperable primary penile squamous cell carcinoma (SCC). It cites studies showing that neoadjuvant ChT can lead to "surgical downstaging" and enable patients to undergo radical surgery. This directly aligns with the recommendation's goal of using neoadjuvant chemotherapy to achieve local control and facilitate a subsequent, less aggressive surgery. |
| NCCN2025 | R | The NCCN guideline supports the use of neoadjuvant chemotherapy for the primary tumor in specific situations. On page PN-D (1 of 5), under "Neoadjuvant Chemotherapy Prior to ILND or PLND," it explicitly states, "Patients with initially unresectable (T4) primary tumors may be downstaged by response to chemotherapy." This aligns with the recommendation's goal of using neoadjuvant chemotherapy to achieve initial local control and enable subsequent surgery. While the guideline focuses on unresectable T4 tumors, the principle of downstaging to facilitate surgical intervention is clearly recommended. |

**27. Systemic Therapy (Radiotherapy for Penile Metastasis)**

**27.1 Radiotherapy for symptomatic Penile Metastasis (PM) is primarily palliative to manage pain, priapism, bleeding, and urinary dysfunction, with dosages from 25 Gy to 60 Gy depending on goals.**

[2]

| AUA2020 | NA | The treatment recommendation specifies the use of palliative radiotherapy for symptomatic metastatic penile cancer. The AUA guideline, however, is explicitly titled "Treatment of Early Stage Squamous Cell Carcinoma of the Penis" and its content focuses exclusively on management options for non-invasive and early-stage disease (Tis, Ta, T1). While the guideline does have a section on radiation, it discusses its use for treating the primary, early-stage tumor, not for palliative management of metastatic disease. |
| --- | --- | --- |
| DGU2020 | R | The guideline supports the use of palliative radiotherapy for symptomatic metastatic penile cancer. Section 7.3.2 ("Palliative Strahlentherapie") states that this treatment is used when curative therapy is not possible, with the goal of improving symptoms and preventing complications. This aligns with the recommendation's aim to manage pain, bleeding, and other dysfunctions. The guideline suggests a hypofractionated dosage schedule (e.g., 12 x 3 Gy, totaling 36 Gy), which is consistent with the 25 Gy to 60 Gy range proposed in the recommendation. |
| EAU-ASCO2025 | R | The guideline strongly supports the use of radiotherapy for palliative purposes in advanced penile cancer. Section 6.5.3, "Role of radiotherapy in palliation," explicitly states that "Radiotherapy is frequently necessary for palliation of penile cancer" and should be customized for unique presentations. This aligns with the recommendation's core message of using radiotherapy for symptomatic relief. Furthermore, the recommendation table in section 6.5.4 gives a "Strong" strength rating to the statement, "Offer radiotherapy for symptom control (palliation) in advanced disease," directly endorsing the proposed treatment's palliative intent. |
| ESMO2024 | R | The guideline recommends considering palliative radiotherapy (RT) or RT with concurrent chemotherapy for sites requiring local control in metastatic penile cancer. This directly aligns with the treatment recommendation, which proposes using palliative RT to manage local symptoms like pain, bleeding, and urinary dysfunction. The guideline states, "Palliative RT or RT with concurrent ChT for sites requiring local control should be considered [V, C]." Although the guideline does not specify dosages, it confirms that palliative RT is an appropriate treatment approach. |
| NCCN2025 | R | The NCCN guideline supports the use of palliative radiotherapy for metastatic penile cancer. The "Principles of Radiotherapy" page (PN-C) includes a section for "Palliative RT," which recommends a palliative dose of 30 Gy in 10 fractions. This specific dose falls within the 25 Gy to 60 Gy range mentioned in the recommendation. Furthermore, the algorithm for metastatic disease (PN-10) and the discussion section (MS-17) both endorse considering radiotherapy for local control, palliative benefit, and best supportive care. |

**27.2 Stereotactic body radiotherapy (SBRT), e.g., 25 Gy in 5 fractions, is a valuable approach for Penile Metastasis (PM) that can provide good symptomatic relief and local control, especially in oligometastatic disease.**

[2]

| AUA2020 | NA | The AUA Update Series guideline focuses exclusively on the "Treatment of Early Stage Squamous Cell Carcinoma of the Penis," as stated in its title and learning objectives. It details management for non-invasive and early invasive disease (Tis, Ta, T1) using topical therapies, laser ablation, and organ-preserving surgeries. The treatment recommendation, however, pertains to stereotactic body radiotherapy (SBRT) for Penile Metastasis (PM), which represents advanced or metastatic disease. The guideline's scope does not extend to metastatic disease, and as such, it does not address SBRT or other treatments for this advanced stage. |
| --- | --- | --- |
| DGU2020 | R | The guideline addresses palliative radiotherapy for metastatic penile cancer in section 7.3.2. It explicitly states that palliative radiotherapy is typically delivered in a hypofractionated manner, with single doses ranging from 3 to 5 Gy per day (p. 80). The treatment recommendation for Stereotactic Body Radiotherapy (SBRT) with a regimen of 25 Gy in 5 fractions (5 Gy per fraction) aligns perfectly with this principle and falls within the specified dose-per-fraction range, thus representing a supported treatment approach. |
| EAU-ASCO2025 | R | The guideline strongly recommends palliative radiotherapy for symptom control in advanced penile cancer. In Section 6.5.3, "Role of radiotherapy in palliation," it states, "Radiotherapy is frequently necessary for palliation of penile cancer and should be customised for unique presentations," and advises that "standard palliative regimens should be readily employed." Although the guideline does not specify stereotactic body radiotherapy (SBRT) or the exact 25 Gy in 5 fractions dosage, this approach falls under the umbrella of a standard palliative regimen for symptomatic relief, aligning with the guideline's strong recommendation to "Offer radiotherapy for symptom control (palliation) in advanced disease." |
| ESMO2024 | NA | for local control in patients with metastatic penile cancer. However, it does not specify the radiotherapy technique or provide details on dosing schedules for this setting. The treatment recommendation suggests a specific advanced modality, Stereotactic Body Radiotherapy (SBRT), with a particular dose and fractionation (25 Gy in 5 fractions), which is not mentioned or discussed anywhere in the provided guideline. |
| NCCN2025 | DR | The guideline recommends radiotherapy (RT) for local control and palliative benefit in metastatic penile cancer. However, under "Palliative RT," it specifically suggests a dose of 30 Gy in 10 fractions (PN-C). The guideline does not mention Stereotactic Body Radiotherapy (SBRT) or the different hypofractionated regimen of 25 Gy in 5 fractions proposed by the recommendation, thereby suggesting a different radiotherapy approach. |

**27.3 Adjuvant radiotherapy (e.g., 30 Gy in 10 fractions) can be considered after surgery for Penile Metastasis (PM) to help prevent recurrence.**

[2]

| AUA2020 | NA | The treatment recommendation specifies the use of adjuvant radiotherapy for penile metastasis (PM). The provided AUA guideline, however, explicitly focuses on the "Treatment of Early Stage Squamous Cell Carcinoma of the Penis," covering non-invasive and localized disease (e.g., Tis, Ta, T1). The document details therapies such as topical treatments, laser ablation, and organ-preserving surgery but does not address the management of metastatic disease. Therefore, the guideline's scope does not include the clinical scenario described in the recommendation. |
| --- | --- | --- |
| DGU2020 | R | The guideline does not recommend adjuvant radiotherapy for all patients after lymphadenectomy due to insufficient evidence. However, Recommendation 7.42 specifies that adjuvant radiotherapy "can be considered" (`kann... erwogen werden`) for patients at high risk of lymph node recurrence, such as those with a high number of metastases or extracapsular growth. This aligns with the treatment recommendation's suggestion that adjuvant radiotherapy "can be considered" to help prevent recurrence in patients with penile metastasis. |
| EAU-ASCO2025 | NA | The guideline is focused on the diagnosis and management of primary penile squamous cell carcinoma (SCC) and its subsequent spread. While it briefly defines penile metastases as secondary tumors originating from other cancers (e.g., prostatic, bladder, or colorectal) on page 10, it does not provide any treatment recommendations for this specific clinical situation. All radiotherapy sections discuss its use for primary tumors or regional lymph node metastases from primary penile cancer, not for treating metastases to the penis from another primary site. |
| ESMO2024 | DR | The guideline recommends a different radiotherapy dosage and fractionation schedule compared to the treatment recommendation. While the guideline does consider adjuvant radiotherapy for regional lymph node metastases in high-risk (pN3) patients, it specifies a dose of "at least 50 Gy in 2 Gy fractions or a biological equivalent dose in 1.8 Gy fractions." Conversely, the treatment recommendation suggests a hypofractionated regimen (30 Gy in 10 fractions), which the guideline explicitly argues against, stating, "There are no arguments to support hypofractionation." |
| NCCN2025 | DR | The NCCN guideline recommends radiotherapy in the context of metastatic disease for palliative purposes or local control, not as an adjuvant therapy to prevent recurrence. The specific dose mentioned in the recommendation, "30 Gy in 10 fractions," is explicitly listed under "Palliative RT" in the guideline's Principles of Radiotherapy (PN-C). The discussion section on metastatic disease further clarifies that the role of RT is considered "mostly palliative" (MS-17), which represents a different clinical intent than the adjuvant approach proposed in the recommendation. |

**28. Systemic Therapy (for Refractory Disease)**

**28.1 For patients with advanced or metastatic disease refractory to first-line chemotherapy, immunotherapy with immune checkpoint inhibitors (e.g., Avelumab, Nivolumab/Ipilimumab) may be considered, particularly for those with Microsatellite Instability-High (MSI-H) or high Tumor Mutational Burden (TMB-H).**

[45]; [3]

| AUA2020 | NA | The guideline exclusively focuses on the "Treatment of Early Stage Squamous Cell Carcinoma of the Penis," as stated in its title and learning objective. It details various local therapies like topical treatments, laser therapy, and organ-preserving surgeries for non-invasive and early-stage disease (Tis, Ta, T1). The treatment recommendation, however, pertains to a different patient population: those with "advanced or metastatic disease" refractory to chemotherapy, for whom it suggests immunotherapy. The guideline does not discuss the management of advanced or metastatic penile cancer, nor does it mention immunotherapy or immune checkpoint inhibitors. |
| --- | --- | --- |
| DGU2020 | R | The guideline aligns with the recommendation. In the section on palliative systemic therapy, the guideline discusses second-line treatment options for refractory metastatic disease. It specifically states that for therapy with checkpoint inhibitors, there are no study results yet, but it can be useful to determine the microsatellite status to then begin immunotherapy, although it notes that microsatellite instability is rare in penile cancer (p. 79). This aligns with the recommendation to consider immunotherapy with checkpoint inhibitors for refractory patients, particularly those with MSI-H status. |
| EAU-ASCO2025 | R | The guideline supports the use of immunotherapy for patients with advanced penile cancer who have progressed on first-line chemotherapy, which aligns with the treatment recommendation. Specifically, Section 6.5.2.1 discusses immune-checkpoint blockade, including nivolumab and ipilimumab, as emerging potential treatment options with early evidence of activity from basket trials. While the guideline notes a lack of definitive data, its formal recommendation in Section 6.5.4 for patients with progressive disease is to "Offer patients...the opportunity to enroll in clinical trials, including experimental therapies within phase I or basket trials," which is a strong endorsement for this approach in a controlled setting. The guideline does not, however, mention MSI-H or TMB-H as specific biomarkers for patient selection. |
| ESMO2024 | R | The guideline supports the use of immunotherapy for specific patient populations with advanced or metastatic penile cancer. The "Molecular profiling and immunotherapy" section states that immunotherapy may be a useful strategy in chemo-resistant disease for patients with high Tumor Mutational Burden (TMB), Microsatellite Instability-High (MSI-H), or dMMR tumors. The formal recommendations further state that "In selected patients whose tumours have a high TMB, MSI-H or dMMR, the use of anti-PD-L1 immunotherapy can be considered [V, C]," which directly aligns with the proposed treatment recommendation. |
| NCCN2025 | R | The NCCN guidelines support the use of immune checkpoint inhibitors for patients with metastatic penile cancer refractory to first-line chemotherapy. In the section "Subsequent-Line Systemic Therapy for Metastatic/Recurrent Disease," the guideline lists Pembrolizumab as a preferred regimen for patients whose tumors are microsatellite instability-high (MSI-H), mismatch repair-deficient (dMMR), or tumor mutational burden-high (TMB-H). This directly aligns with the treatment recommendation to consider immunotherapy for this specific patient population based on the same biomarkers. |

**29. Systemic Therapy (Targeted / Immunotherapy - Investigational)**

**29.1 Based on molecular profiles, the following are proposed: PARP inhibitors for defective DNA repair/MSI; mTOR inhibitors for TP53-mutated tumors; EGFR/COX2 inhibitors for tumors with alterations in ADAM6/EGFR/COX2; and imatinib for PDGFA-mutated tumors.**

[46]

| AUA2020 | NA | The AUA guideline focuses exclusively on the treatment of early-stage (Tis, Ta, T1) squamous cell carcinoma of the penis. It details local therapies such as topical treatments (5-FU, imiquimod), laser therapy, and surgical options (e.g., circumcision, glansectomy). The recommendation, however, proposes the use of systemic, molecularly targeted therapies like PARP inhibitors and mTOR inhibitors based on specific genetic mutations. As the guideline's scope is limited to early-stage disease and its associated local treatments, it does not address these advanced systemic or targeted therapies at all. |
| --- | --- | --- |
| DGU2020 | DR | The guideline recommends standard, Cisplatin-based polychemotherapy for metastatic penile cancer (Empfehlung 7.52, 7.54). It explicitly states that there are currently no molecular markers that can be recommended for routine diagnostics to guide treatment (Statement 4.9). While the guideline acknowledges research into targets like EGFR and MSI, it concludes there is no approved targeted therapy and the data is insufficient for specific recommendations. The proposed molecularly-profiled treatments, such as PARP inhibitors or mTOR inhibitors, are not mentioned or endorsed. |
| EAU-ASCO2025 | DR | The guideline suggests a different, more cautious approach to targeted therapies. While it acknowledges that EGFR inhibitors have been tested with sporadic activity reported, it emphasizes that currently, limited additional systemic options exist and specific actionable genetic alterations are rare. The guideline explicitly states that for the most frequently identified actionable alteration (PIK3CA), "no data currently support the use of selected targeted agents" (page 36, section 6.5.2.2). It does not mention or endorse the other specific molecularly-profiled treatments proposed in the recommendation, such as PARP inhibitors, mTOR inhibitors, or imatinib. |
| ESMO2024 | DR | The guideline recommends a different approach based on molecular profiling than the one proposed. The guideline's recommendations focus on using biomarkers like TMB, MSI-H, or dMMR to identify candidates for anti-PD-L1 immunotherapy. It also generally advises considering biomarker-selected clinical trials. In contrast, the treatment recommendation suggests a specific list of targeted therapies (e.g., PARP inhibitors, mTOR inhibitors, imatinib) based on various genetic alterations, none of which are specifically endorsed or mentioned in the guideline. |
| NCCN2025 | DR | The guideline supports the general principle of using molecular profiling for metastatic disease to identify "rare and actionable mutations and fusions" (PN-10) and using tumor-agnostic therapies based on NGS results (PN-D 3 of 5). However, it does not recommend the specific drug-mutation pairings listed in the recommendation. For example, for MSI-H/dMMR tumors, the guideline recommends the immune checkpoint inhibitor pembrolizumab, not PARP inhibitors as proposed (PN-D 3 of 5). While cetuximab (an EGFR inhibitor) is mentioned as a subsequent-line option, it is not specifically linked to EGFR/COX2 alterations, and the other proposed targeted therapies (mTOR inhibitors, imatinib) are not mentioned at all. |

**29.2 For patients with NOTCH1-mutated tumors, enrollment in clinical trials focused on PI3K/mTOR inhibitors is suggested.**

[46]

| AUA2020 | NA | The AUA Update Series guideline focuses on the treatment of early-stage squamous cell carcinoma of the penis. Its recommendations are based on clinical stage, grade, and lesion location, covering topical therapies, laser ablation, various surgical approaches, and radiation. The document does not address the molecular or genetic basis of penile cancer, and therefore makes no mention of specific mutations like NOTCH1, targeted therapies such as PI3K/mTOR inhibitors, or enrollment in clinical trials based on these factors. |
| --- | --- | --- |
| DGU2020 | NA | The guideline does not address the use of PI3K/mTOR inhibitors for patients with NOTCH1-mutated penile cancer. The section on molecular biology (4.4.4) states that no molecular markers are currently recommended for routine diagnostics. While the section on palliative systemic therapy (7.3.1) discusses various targeted therapies (e.g., EGFR inhibitors, checkpoint inhibitors), it makes no mention of NOTCH1 mutations or the PI3K/mTOR pathway as a potential therapeutic target. Therefore, the specific recommendation is not addressed in the guideline. |
| EAU-ASCO2025 | NA | The EAU-ASCO guideline does not mention NOTCH1 mutations as a target for therapy in penile cancer. In the section on "Targeted therapies" (6.5.2.2), the guideline states that specific actionable genetic alterations are rare. While it does mention PIK3CA gene alterations (related to the PI3K pathway), it explicitly concludes that "no data currently support the use of selected targeted agents in this disease." Therefore, the specific recommendation to enroll patients with NOTCH1 mutations in trials for PI3K/mTOR inhibitors is not addressed. |
| ESMO2024 | NA | The guideline discusses molecular profiling for metastatic penile cancer, recommending biomarker-selected clinical trials where available. It specifically mentions considering anti-PD-L1 immunotherapy for patients with tumors that have a high tumor mutational burden (TMB), are microsatellite instability-high (MSI-H), or are mismatch repair deficient (dMMR). However, the guideline does not mention NOTCH1 mutations or the use of PI3K/mTOR inhibitors as a potential therapeutic strategy for this specific patient subgroup. |
| NCCN2025 | NA | The guideline does not mention NOTCH1 mutations or the use of PI3K/mTOR inhibitors for treating penile cancer. While the guideline does recommend considering broad molecular/genomic testing to identify "rare and actionable mutations and fusions" for metastatic disease (PN-10) and strongly encourages participation in clinical trials (PN-10, MS-18), it does not specify any particular mutations (like NOTCH1) or targeted therapies (like PI3K/mTOR inhibitors). Therefore, this specific recommendation is not addressed. |

# Section 8: Assessment of Stance Classification of Guidelines Toward Literature Recommendations

## Prompt

You are given the following components:

- Guideline - a comprehensive clinical guideline on penile cancer treatment.
- Treatment Recommendation - a proposed approach or method for treating penile cancer.
- Extracted Stance - a previously determined stance that classifies the guideline's position toward the recommendation. This stance is one of the following: Recommended, Different Recommendation, or Not Addressed. As well as a concise justification.

* Correction Suggestions – an evaluative assessment of the guideline's position on the recommended treatment.

Based on the suggestions on correction, formulate a corrected version of the guideline's stance on the recommended treatment.

\# Treatment Recommendation:

RECOMMENDATION_TEXT

\# Extracted Stance

GUIDELINE_SNIPPET_TEXT

\# Guideline (context):

GUIDELINE_TEXT

\# Correction Suggestions

SUGGESTION_TEXT

\# Formatting Instructions:

Provide an updated classification code that can be one out of [R (Recommended); DR (Different Recommendation); NA (Not Addressed)] and an updated summary that justifies that classification referencing key elements from both the recommendation and the guideline.

Output Table: white_areas_consensus_table_panel_progress.tsv

# Section 9: External Expert Appraisal of Delphi-Ready Candidate Items

## Table of Initial Candidate Items for the Delphi Process Prior to External Expert Appraisal, Derived From Divergent Guideline Recommendations (G01–G53) and the Supplementary Literature Review (W01–W09)

| **Item** | **Origin** | **Category** | **Candidate Item** |
| --- | --- | --- | --- |
| G01 | Guideline-derived item | Diagnosis & Staging | Perform magnetic resonance imaging (MRI) or penile ultrasound (US) of the primary tumor when there is uncertainty regarding corporal invasion (T3) and/or the feasibility of organ-sparing surgery. |
| G02 | Guideline-derived item | Diagnosis & Staging | Computerized tomography (CT) of the chest, abdomen, and pelvis is performed to evaluate for distant metastatic spread. For high-risk or node-positive disease, ¹⁸F-FDG-PET/CT can also be used. |
| G03 | Guideline-derived item | Diagnosis & Staging | Cross-sectional imaging (CT, PET/CT) for primary staging should generally be reserved for T3/T4 tumors or in cases of positive lymph node status. |
| G04 | Guideline-derived item | Diagnosis & Staging | For superficial, non-invasive penile carcinomas (Carcinoma in situ), a fluorescence-diagnostic technique can be used to better determine the extent of the lesion. |
| G05 | Guideline-derived item | Diagnosis & Staging | For clinically node-negative (cN0) low-risk tumors (PeIN, pTa, pT1a G1), surveillance of the inguinal lymph nodes is recommended instead of operative staging. |
| G06 | Guideline-derived item | Diagnosis & Staging | For clinically node-negative (cN0) intermediate-risk tumors (T1a G2), surveillance may be discussed as an alternative to surgical staging with patients who are willing to comply with strict follow-up. |
| G07 | Guideline-derived item | Diagnosis & Staging | The preferred method for surgical staging in cN0 patients is dynamic sentinel node biopsy (DSNB). If DSNB is unavailable or not feasible, modified inguinal lymph node dissection (ILND) is an alternative. If DSNB is positive, a completion ILND is recommended. |
| G08 | Guideline-derived item | Diagnosis & Staging | If DSNB is planned, perform inguinal ultrasound (US) first, with fine needle aspiration cytology (FNAC) of any sonographically abnormal lymph nodes. |
| G09 | Guideline-derived item | Diagnosis & Staging | In cN0 patients undergoing staging, frozen section analysis should not be performed on tissue from a modified inguinal lymphadenectomy or DSNB. |
| G10 | Guideline-derived item | Diagnosis & Staging | For clinically node-positive (cN+) disease, an (image-guided) biopsy of the suspicious node is required to confirm metastasis before initiating treatment. |
| G11 | Guideline-derived item | Primary Treatment (General Principles) | The goal of surgical treatment is complete excision with negative margins (R0). A margin of 5 mm for G1/G2 tumors and 10 mm for G3 tumors is suggested. In case of a positive surgical margin, a re-excision should be performed. |
| G12 | Guideline-derived item | Primary Treatment (Topical Therapy) | If topical treatment fails, it should not be repeated. |
| G13 | Guideline-derived item | Primary Treatment (Topical Therapy) | Topical therapy is ineffective for more advanced stages of penile cancer. |
| G14 | Guideline-derived item | Primary Treatment (Laser Therapy) | A surgical margin of 5 mm is considered oncologically safe for laser therapy. |
| G15 | Guideline-derived item | Primary Treatment (Post-Therapy Biopsy) | After topical or laser therapy, a control biopsy should be performed to verify local tumor control, especially in cases of clinical doubt. |
| G16 | Guideline-derived item | Primary Treatment (Surgical) | Organ-sparing surgery options (circumcision, wide local excision, glansectomy, Mohs) are offered for lesions confined to the glans and prepuce (PeIN, Ta, T1-T2) in patients willing to comply with strict follow-up. |
| G17 | Guideline-derived item | Primary Treatment (Surgical) | Intra-operative frozen section analysis of resection margins should be performed to confirm negative margins, especially in cases of doubt. |
| G18 | Guideline-derived item | Primary Treatment (Surgical) | When using radiotherapy or topical therapy for a preputial lesion, a circumcision should be performed first. |
| G19 | Guideline-derived item | Primary Treatment (Surgical) | Wide local excision (WLE) is an option for early-stage tumors (PeIN, Ta, T1). Complete excision with wide negative margins is required. |
| G20 | Guideline-derived item | Primary Treatment (Surgical) | Mohs micrographic surgery can be considered as an alternative to WLE for select, low-grade, superficial lesions, particularly on the glans or distal penis, if the appropriate expertise is available. |
| G21 | Guideline-derived item | Primary Treatment (Surgical) | Glansectomy (partial or total) or glans resurfacing are options for tumors located on the glans penis (PeIN, Ta, T1-T2). Reconstruction may be performed with a split-thickness skin graft. |
| G22 | Guideline-derived item | Primary Treatment (Radiotherapy) | For T3–4 or N+ surgically unresectable disease, consider EBRT with concurrent chemotherapy to the penis and regional lymph nodes. |
| G23 | Guideline-derived item | Primary Treatment (Radiotherapy) | Specific radiotherapy doses may include: • EBRT: 66-74 Gy in 2 Gy fractions. • Brachytherapy (HDR): 42-45 Gy in 12-14 fractions. • Brachytherapy (LDR/PDR): 60-65 Gy delivered continuously over ~5 days. |
| G24 | Guideline-derived item | Primary Treatment (Multimodal) | For non-resectable, locally advanced primary tumors (T4), consider induction/neoadjuvant chemotherapy followed by surgery for responders, or chemo-radiotherapy. |
| G25 | Guideline-derived item | Recurrent Disease | After radiotherapy, local recurrence should be treated with surgical salvage therapy. |
| G26 | Guideline-derived item | Recurrent Disease | After topical or laser therapy, local recurrence should be treated with a different treatment modality. |
| G27 | Guideline-derived item | Recurrent Disease | Regional lymph node recurrence after initial surveillance should be managed according to the same principles as primary lymph node metastases (i.e., with ILND +/- systemic therapy). |
| G28 | Guideline-derived item | Recurrent Disease | Regional lymph node recurrence after a prior radical lymphadenectomy should be managed with a multimodal approach, including neoadjuvant chemotherapy, surgery, and/or radiotherapy. |
| G29 | Guideline-derived item | Lymph Node Management | Following circumcision for a primary tumor, patients with moderately or poorly differentiated tumors (grades 2 and 3) will require inguinal node dissection. |
| G30 | Guideline-derived item | Lymph Node Management | For palpable, mobile inguinal nodes (cN1-cN2), inguinal lymph node dissection (ILND) is recommended. The dissection may be fascial-sparing or radical, with preservation of the saphenous vein where possible. |
| G31 | Guideline-derived item | Lymph Node Management | If intraoperatively suspicious nodes are removed during surgery for cN+ disease, frozen section analysis should be performed. If positive, an ipsilateral radical lymphadenectomy should be completed in the same session. |
| G32 | Guideline-derived item | Lymph Node Management | For bulky, fixed, or extensive inguinal nodes (cN3), neoadjuvant chemotherapy (NAC) with a cisplatin- and taxane-based regimen is preferred over up-front surgery. |
| G33 | Guideline-derived item | Lymph Node Management | Minimally-invasive ILND for cN1–2 disease should only be offered as part of a clinical trial. It is contraindicated for cN3 disease. |
| G34 | Guideline-derived item | Lymph Node Management | Following a response to neoadjuvant chemotherapy, patients should undergo consolidative surgery, including radical inguinal lymphadenectomy and possible pelvic lymphadenectomy. |
| G35 | Guideline-derived item | Lymph Node Management | Ipsilateral pelvic lymph node dissection (PLND) is recommended for patients with ≥2-3 positive inguinal nodes on one side or with extranodal extension. |
| G36 | Guideline-derived item | Systemic Therapy | Recommended neoadjuvant chemotherapy regimens are cisplatin- and taxane-based triplets, such as TIP (Paclitaxel/Ifosfamide/Cisplatin) or TPF (Cisplatin/Paclitaxel/5-Fluorouracil). |
| G37 | Guideline-derived item | Systemic Therapy | Adjuvant chemotherapy should be considered for patients with pN2 or pN3 disease following lymph node dissection. |
| G38 | Guideline-derived item | Systemic Therapy | Adjuvant radiotherapy (with or without chemo-sensitization) should be offered or considered for patients with high-risk pathological findings such as pN2/N3 disease or extranodal extension. |
| G39 | Guideline-derived item | Systemic Therapy | Definitive radiotherapy (with or without chemo sensitization) can be offered to patients who are unwilling or unable to undergo surgery. |
| G40 | Guideline-derived item | Systemic Therapy | For first-line treatment of metastatic disease, platinum-based combination chemotherapy is the preferred approach. Recommended regimens include TIP, TPF, or cisplatin + 5-FU. |
| G41 | Guideline-derived item | Systemic Therapy | Bleomycin-containing chemotherapy regimens should not be used due to the risk of pulmonary toxicity. |
| G42 | Guideline-derived item | Systemic Therapy | For patients with progressive disease, enrollment in a clinical trial is the preferred option for subsequent-line therapy. |
| G43 | Guideline-derived item | Systemic Therapy | Subsequent-line therapy options may include paclitaxel monotherapy or, for biomarker-selected tumors (MSI-H, dMMR, TMB-H), immunotherapy such as pembrolizumab. |
| G44 | Guideline-derived item | Palliative & Supportive Care | Patients with metastatic disease or requiring multimodal therapy should be discussed in an interdisciplinary tumor board. |
| G45 | Guideline-derived item | Palliative & Supportive Care | Palliative radiotherapy can be offered for symptom control in advanced disease (e.g., 30 Gy in 10 fractions). |
| G46 | Guideline-derived item | Palliative & Supportive Care | For chronic lymphedema resulting from treatment, a complex decongestive therapy (lymphatic drainage, compression therapy, and skin care) should be performed. |
| G47 | Guideline-derived item | Follow-up & Survivorship | A general follow-up schedule is every 3-4 months for the first 2 years, then less frequently. Follow-up should extend for at least 5 years. |
| G48 | Guideline-derived item | Follow-up & Survivorship | Specific follow-up schedules after laser therapy are: • CO2 Laser: After 2 months, then every 3 months for 3 years, then biannually. • Nd:YAG Laser: Every 3 months for 2 years, then biannually. |
| G49 | Guideline-derived item | Follow-up & Survivorship | Specific follow-up schedules based on treatment: • Organ Preservation: Every 3 mo (Yrs 1-2), every 6 mo (Yrs 3-5). • Total Penectomy: Every 3 mo (Yrs 1-2), annually (Yrs 3-5). |
| G50 | Guideline-derived item | Follow-up & Survivorship | Patients should be taught self-examination for early detection of recurrence and given easy access back to the clinic if concerns arise. |
| G51 | Guideline-derived item | Follow-up & Survivorship | Penile cancer care should be delivered by an extended multi-disciplinary team including specialists in urology, oncology, nursing, pathology, radiology, psychology, and lymphedema therapy. |
| G52 | Guideline-derived item | Follow-up & Survivorship | Patients should be offered psychological support and counseling, and physicians should discuss the psychological and functional (urinary, sexual) impact of the disease and its treatments. |
| G53 | Guideline-derived item | Follow-up & Survivorship | Patients should be assessed for lymphedema at follow-up and referred to lymphedema therapists early if it develops. |
| W01 | Literature-derived true white-area item | General Principles / Management | For patients with locally advanced disease who are kidney transplant recipients, treatment should be individualized to minimize graft injury, preferring non-cytotoxic options over radiotherapy and cytotoxic treatments. |
| W02 | Literature-derived true white-area item | Cancer Prevention | The use of male condoms is recommended as the most effective method for reducing the transmission of Sexually Transmitted Infections (STIs) like HPV and HIV, which are established risk factors for penile cancer. |
| W03 | Literature-derived true white-area item | Complication Management | Primary Penile Surgery Complications: • Surgical Site Infections (SSIs): Manage with empirical antibiotics, transitioning to culture-specific therapy, along with regular wet-to-dry dressing changes. • Meatal Stenosis: Manage with self-catheterization or formal surgical revision.• Partial Skin Graft Loss: Manage expectantly with observation. • Total Skin Graft Loss: Manage with wet-to-dry dressing changes followed by delayed regrafting. |
| W04 | Literature-derived true white-area item | Complication Management | Inguinal Surgery Complications - Wound Issues: • Infected Inguinal Wounds: Manage with meticulous wound care, daily inspection, and culture-specific antibiotics. • Wound Dehiscence / Flap Necrosis: Manage with wet-to-dry dressings or vacuum-assisted closure (VAC) dressings, followed by delayed closure. • Patients with Large Body Habitus: Place dry gauze ("Interdry") in groin folds to prevent moisture buildup. |
| W05 | Literature-derived true white-area item | Lymph Node Management | The management of inguinal nodal disease in transplanted patients should be consistent with standard oncological guidelines. |
| W06 | Literature-derived true white-area item | Perioperative Care | Strict diabetes control is crucial for avoiding wound infection-related adverse events in patients undergoing ILND. |
| W07 | Literature-derived true white-area item | Postoperative Care | Primary Penile Surgery - Wound and Catheter Management: • Dressing: Use compressive surgical dressings for 2-7 days. • Foley Catheter: Typically remove around 7 days post-surgery for patients who undergo neomeatus creation or STSG application. |
| W08 | Literature-derived true white-area item | Screening | General screening protocols for penile cancer are not recommended in kidney transplant recipients. |
| W09 | Literature-derived true white-area item | Systemic Therapy (Targeted / Immunotherapy - Investigational) | For patients with NOTCH1-mutated tumors, enrollment in clinical trials focused on PI3K/mTOR inhibitors is suggested. |

## Characteristics of the Four External Expert Raters

| **Expert** | **Expert 1** | **Expert 2** | **Expert 3** | **Expert 4** |
| --- | --- | --- | --- | --- |
| **Specialty** | Urology and uro-oncology; particular expertise in psycho-oncology, quality of life, and penile cancer | Urology, surgical uro-oncology, and translational penile-cancer research; head of the penile-cancer research group | Urology and surgical uro-oncology, with clinical and translational expertise in penile cancer | Urology and uro-oncology, including penile-cancer care, outcomes research, and health-services research |
| **Country / region** | Rostock, Germany | Mainz, Germany | Tübingen, Germany | Greifswald, Germany |
| **Criterion 1**  **Publication record** | ≥14 PubMed-indexed penile-cancer publications | ≥11 PubMed-indexed penile-cancer publications | Met: ≥12 PubMed-indexed penile-cancer publications | ≥6 PubMed-indexed penile-cancer publications |
| **Criterion 2**  **Guideline, consensus, or systematic-review work** | Lead of the German S3 penile-cancer guideline update | EAU-YAU Penile and Testis Cancer Working Group; co-author of a Delphi consensus study | International collaborative reviews and consensus-oriented work in penile cancer | Guideline-adherence, centralization, and governance studies in penile cancer |
| **Criterion 3**  **Referral-center or high-volume activity** | Executive senior physician at the certified penile-cancer center, University Medical Center Rostock | Senior physician and principal surgical lead at a certified supraregional penile-cancer center, University Medical Center Mainz | Medical Director and Chair at University Hospital for Urology, Tübingen, with a dedicated penile-tumor program and referral pathways | Main surgeon within the recognized university-based Uro-Oncological Center at University Medicine Greifswald, providing multidisciplinary penile-cancer care |
| **Criterion 4**  **Study, registry, or multicenter leadership** | Participation in penile-cancer cohorts and studies without a documented PI, co-PI, or steering-group role | Translational penile-cancer research and eUROGEN collaborations | Principal investigator/clinical lead of DEPECA-1 and DEPECA-2 | E-PROPS working-group participation and investigator-level involvement in multicenter penile-cancer initiatives |
| **Eligibility criteria fulfilled**  **(minimum: 2 of 4)** | **Criteria 1, 2, and 3 (3/4)** | **Criteria 1, 2, and 3 (3/4)** | **Criteria 1, 2, 3, and 4 (4/4)** | **Criteria 3 and 4 (2/4)** |
| **Declared conflicts of interest** | No declared conflicts of interest | No declared conflicts of interest | No declared conflicts of interest | No declared conflicts of interest |
| * Eligibility required clinical or academic activity in penile cancer and fulfillment of at least 2 of criteria 1–4. Publication counts are conservative minima identified through targeted PubMed author-topic searches performed on 14 July 2026. | | | | |

## Rating Framework and Assessment Criteria for Delphi-Ready Candidate Items

| **Purpose** | | Assess whether each candidate item is suitable for inclusion in a subsequent Delphi consensus survey. This is not a clinical vote and does not establish disease-specific consensus. |
| --- | --- | --- |
| **Question to be answered for each item** | | *Is this item appropriate for inclusion in a subsequent Delphi consensus survey?* |
| **Likert Scale** | | |
| **Code** | **Meaning** | **Interpretation** |
| 1 | Strongly disagree | The item is not appropriate for inclusion in a subsequent Delphi consensus survey. |
| 2 | Disagree | The item is probably not appropriate for inclusion without substantial revision. |
| 3 | Uncertain | The item may be appropriate but requires clarification, revision, merging, or further adjudication. |
| 4 | Agree | The item is appropriate for inclusion in a subsequent Delphi consensus survey. |
| 5 | Strongly agree | The item is clearly appropriate for inclusion in a subsequent Delphi consensus survey. |
| **Reason Codes for Ratings 1-3** | | |
| **Code** | **Reason** | **Use When** |
| 1 | Unclear wording | The item is difficult to interpret or not phrased clearly enough for later voting. |
| 2 | Limited clinical relevance | The item does not appear sufficiently relevant for penile cancer consensus work. |
| 3 | Redundant or overlapping with another item | The item substantially duplicates another item. |
| 4 | Insufficiently derived from guideline divergence or white-area evidence | The item does not appear adequately supported as a guideline-divergent, incompletely addressed, or white-area candidate. |
| 5 | Should be merged with another item | The item should not stand alone and is better combined with another item. |
| 6 | Other reason / unable to judge | Use this if none of the above categories apply. |

## External Assessment of the Delphi-Ready Candidate Items by Four Independent Expert Raters, Including the Median and Interquartile Range.

| **Item** | **Expert 1** | **Expert 2** | **Expert 3** | **Expert 4** | **Median** | **IQR** |
| --- | --- | --- | --- | --- | --- | --- |
| G01 | 5 | 5 | 4 | 5 | 5 | 4.75-5 |
| G02 | 5 | 5 | 4 | 4 | 4.5 | 4-5 |
| G03 | 4 | 4 | 4 | 5 | 4 | 4-4.25 |
| G04 | 4 | 4 | 4 | 5 | 4 | 4-4.25 |
| G05 | 5 | 5 | 4 | 5 | 5 | 4.75-5 |
| G06 | 4 | 5 | 4 | 5 | 4.5 | 4-5 |
| G07 | 5 | 5 | 4 | 4 | 4.5 | 4-5 |
| G08 | 5 | 5 | 4 | 4 | 4.5 | 4-5 |
| G09 | 5 | 4 | 4 | 4 | 4 | 4-4.25 |
| G10 | 5 | 5 | 4 | 5 | 5 | 4.75-5 |
| G11 | 5 | 5 | 4 | 5 | 5 | 4.75-5 |
| G12 | 5 | 4 | 4 | 5 | 4.5 | 4-5 |
| G13 | 5 | 3 | 3 | 4 | 3.5 | 3-4.25 |
| G14 | 5 | 4 | 4 | 5 | 4.5 | 4-5 |
| G15 | 5 | 5 | 4 | 4 | 4.5 | 4-5 |
| G16 | 5 | 5 | 4 | 5 | 5 | 4.75-5 |
| G17 | 5 | 5 | 4 | 5 | 5 | 4.75-5 |
| G18 | 5 | 5 | 4 | 5 | 5 | 4.75-5 |
| G19 | 5 | 5 | 3 | 5 | 5 | 4.5-5 |
| G20 | 5 | 5 | 4 | 5 | 5 | 4.75-5 |
| G21 | 5 | 5 | 4 | 5 | 5 | 4.75-5 |
| G22 | 5 | 5 | 4 | 4 | 4.5 | 4-5 |
| G23 | 5 | 3 | 4 | 4 | 4 | 3.75-4.25 |
| G24 | 5 | 5 | 4 | 5 | 5 | 4.75-5 |
| G25 | 5 | 5 | 4 | 5 | 5 | 4.75-5 |
| G26 | 5 | 4 | 4 | 4 | 4 | 4-4.25 |
| G27 | 5 | 5 | 4 | 5 | 5 | 4.75-5 |
| G28 | 5 | 5 | 4 | 5 | 5 | 4.75-5 |
| G29 | 5 | 2 | 4 | 5 | 4.5 | 3.5-5 |
| G30 | 5 | 5 | 4 | 5 | 5 | 4.75-5 |
| G31 | 5 | 5 | 4 | 5 | 5 | 4.75-5 |
| G32 | 5 | 5 | 4 | 5 | 5 | 4.75-5 |
| G33 | 5 | 4 | 4 | 5 | 4.5 | 4-5 |
| G34 | 5 | 5 | 4 | 5 | 5 | 4.75-5 |
| G35 | 5 | 5 | 4 | 5 | 5 | 4.75-5 |
| G36 | 5 | 5 | 4 | 5 | 5 | 4.75-5 |
| G37 | 5 | 5 | 4 | 5 | 5 | 4.75-5 |
| G38 | 5 | 5 | 4 | 5 | 5 | 4.75-5 |
| G39 | 5 | 4 | 4 | 5 | 4.5 | 4-5 |
| G40 | 5 | 5 | 4 | 5 | 5 | 4.75-5 |
| G41 | 5 | 5 | 4 | 4 | 4.5 | 4-5 |
| G42 | 5 | 5 | 4 | 5 | 5 | 4.75-5 |
| G43 | 5 | 4 | 4 | 4 | 4 | 4-4.25 |
| G44 | 5 | 5 | 4 | 5 | 5 | 4.75-5 |
| G45 | 5 | 5 | 4 | 5 | 5 | 4.75-5 |
| G46 | 5 | 5 | 4 | 5 | 5 | 4.75-5 |
| G47 | 5 | 3 | 4 | 4 | 4 | 3.75-4.25 |
| G48 | 5 | 4 | 4 | 4 | 4 | 4-4.25 |
| G49 | 5 | 4 | 4 | 5 | 4.5 | 4-5 |
| G50 | 5 | 5 | 4 | 5 | 5 | 4.75-5 |
| G51 | 5 | 5 | 4 | 5 | 5 | 4.75-5 |
| G52 | 5 | 5 | 4 | 5 | 5 | 4.75-5 |
| G53 | 5 | 5 | 4 | 4 | 4.5 | 4-5 |
| W01 | 5 | 4 | 4 | 5 | 4.5 | 4-5 |
| W02 | 5 | 3 | 4 | 5 | 4.5 | 3.75-5 |
| W03 | 5 | 4 | 4 | 4 | 4 | 4-4.25 |
| W04 | 5 | 4 | 4 | 4 | 4 | 4-4.25 |
| W05 | 5 | 3 | 4 | 4 | 4 | 3.75-4.25 |
| W06 | 5 | 5 | 4 | 4 | 4.5 | 4-5 |
| W07 | 5 | 5 | 4 | 4 | 4.5 | 4-5 |
| W08 | 5 | 5 | 4 | 4 | 4.5 | 4-5 |
| W09 | 5 | 4 | 4 | 5 | 4.5 | 4-5 |

## Delphi-Ready Candidate Items Rated ≤3 By At Least One Expert Rater Regarding Their Suitability For Inclusion In The Delphi Process, Together With The Corresponding Reason For The Rating

| **Item** | **Expert 1**  **Rating** | **Expert 2**  **Rating** | **Expert 2**  **Reason Rating** $\boldsymbol{\leq}$**3** | **Expert 3**  **Rating** | **Expert 3**  **Reason Rating** $\boldsymbol{\leq}$**3** | **Expert 4**  **Rating** |
| --- | --- | --- | --- | --- | --- | --- |
| **G13** | 5 | 3 | Unclear wording | 3 | Unclear wording | 4 |
| **G19** | 5 | 5 | - | 3 | Unclear wording | 5 |
| **G23** | 5 | 3 | Unclear wording | 4 | - | 4 |
| **G29** | 5 | 2 | Unclear wording | 4 | - | 5 |
| **G47** | 5 | 3 | Unclear wording | 4 | - | 4 |
| **W02** | 5 | 3 | Insufficiently derived from white-area evidence | 4 | - | 5 |
| **W05** | 5 | 3 | Unclear wording | 4 | - | 4 |

## Substantive Appraisal and Resulting Actions for Candidate Items Receiving Ratings ≤3

| **Item** | **Critical rating(s)** | **Stated reason** | **Substantive response** | **Decision and action** |
| --- | --- | --- | --- | --- |
| G13 | Expert 2 = 3;  Expert 3 = 3 | Unclear wording | G13 is the only item that met the a priori threshold for mandatory revision: two independent raters assigned a score of 3 and both identified unclear wording. The concern cannot be dismissed as isolated inter-rater variation, because the absolute term “ineffective” and the undefined phrase “more advanced stages” leave the intended disease stage, treatment setting, and comparator insufficiently specified. The underlying topic remains relevant, but the current wording requires a complete reassessment rather than a merely editorial defence. | Complete work-up of G13, including a structured reassessment of the item and the concerns raised by the two raters in discussion with the AI model; subsequent revision and optimisation of the wording;  targeted resubmission of the revised item to all experts. |
| G19 | Expert 3 = 3 | Unclear wording | Only one rater assigned a score of 3, whereas the other three raters assigned scores of 5. Under the predefined rule, this isolated concern does not outweigh the strong concordant assessment of suitability or the structured derivation of the item from guideline divergence. The criticism most plausibly reflects the combination of eligibility and margin status in one statement, not a lack of relevance or Delphi suitability. | Retain the item without alteration. |
| G23 | Expert 2 = 3 | Unclear wording | Only one rater assigned a score of 3. The remaining ratings were 5, 4, and 4. The item was generated to capture clinically relevant variation in radiotherapy dose and fractionation, and its technical specificity is an advantage for later Delphi voting. The isolated uncertainty may relate to the presentation of several modalities in one item, but it does not undermine the evidence-based rationale or the appropriateness of the topic. Extensive reformulation could remove precisely the variation the Delphi process is intended to adjudicate. | Retain the item without alteration. |
| G29 | Expert 2 = 2 | Unclear wording | One rater assigned a score of 2, while the other three assigned scores of 5, 4, and 5. The strong majority assessment and the structured guideline-based derivation support retention. The isolated objection appears to concern the categorical terminology and the distinction between invasive nodal staging and therapeutic lymph-node dissection, rather than the relevance of inguinal nodal management itself. This terminology can be acknowledged without reopening or materially redesigning the candidate item. | Retain the item without alteration. |
| G47 | Expert 2 = 3 | Unclear wording | A single rater assigned a score of 3, whereas the remaining ratings were 5, 4, and 4. The broad terms “general” and “less frequently” identify the very uncertainty in surveillance intensity and duration that the Delphi process is intended to resolve. Adding extensive risk stratification at this stage could prematurely narrow the question and pre-empt the consensus process. The item-generation and external-rating procedures therefore provide sufficient support for retention despite one uncertain rating. | Retain the item without alteration. |
| W02 | Expert 2 = 3 | Insufficiently derived from white-area evidence | Only one rater assigned a score of 3; the other ratings were 5, 4, and 5. The stated objection seems to concern provenance rather than wording or clinical comprehensibility. As explained in Section 2.3 of the Output Table on Stance Classification in Supplementary Material 7, none of the guidelines addresses this topic. The item therefore represents a true white area. | Retain the item without alteration. |
| W05 | Expert 2 = 3 | Unclear wording | Only one rater assigned a score of 3; the remaining ratings were 5, 4, and 4. The intentionally broad formulation reflects a genuine white-area question in an underrepresented transplant population: whether standard oncological principles should remain applicable or require adaptation. The absence of detailed qualifiers is not necessarily a flaw, because the Delphi process is intended to determine the degree of agreement and the need for exceptions. The structured literature-based generation and the positive majority assessment support retention. | Retain the item without alteration. |

# Section 10: AI-Assisted Revision of Failed Items

## Prompt

The following prompt was used to instruct the large language model (LLM) Google’s Gemini-2.5-Pro to revise the failed items according to the experts reviewers’ critique:

You are given an extracted treatment recommendation from a clinical guideline that has been identified as requiring human review before inclusion in a Delphi process.

Your task is to revise the wording of the extracted recommendation according to the human reviewer's critique while remaining faithful to the source guideline from which the recommendation was extracted.

**Inputs**

- Original recommendation
- Aspect of Care
- Source guideline
- Guideline stances
- Human reviewer critique

**Task** Revise the original recommendation by addressing the reviewer's critique.

When revising:

- Use the source guideline as the authoritative source.
- Preserve the original clinical intent unless the reviewer identifies that the extracted recommendation does not accurately represent the guideline.
- Do not introduce information that is not supported by the source guideline.
- Make the recommendation as clear, specific, and self-contained as possible.
- Preserve clinically relevant qualifiers whenever they are supported by the guideline, including patient population, disease stage, treatment setting, timing, line of therapy, exceptions, contraindications, and recommendation strength.
- Do not rewrite the recommendation to better match other guidelines.
- If the reviewer requests a change that is not supported by the source guideline, explain this and retain a recommendation that faithfully reflects the guideline.

**Output**

Return only the following JSON object.

- revised_recommendation
- rationale: brief explanation of how the reviewer critique was addressed and why the revision reflects the source guideline
- meaning changed: true / false
- reviewer request supported by guideline: true / false

Where:

- revised recommendation is the revised recommendation.
- rationale briefly explains the revision (1–3 sentences).
- Meaning changed is true only if the clinical meaning of the recommendation had to be changed to better reflect the source guideline; otherwise false.
- Reviewer request supported by guideline indicates whether the requested revision was supported by the source guideline.

Do not include any text outside the JSON object.

## Output Table Containing the Revised Failed Items

| **Original candidate item** | **Revised candidate item** | **Rationale** | **Meaning changed** | **Reviewer request supported by guideline** |
| --- | --- | --- | --- | --- |
| G13 original: Topical therapy is ineffective for more advanced stages of penile cancer. | G13 revised: For patients with penile cancer, topical therapy is an ineffective treatment for advanced stages of the disease (i.e., beyond T1). | The revision clarifies the ambiguous term 'more advanced stages' by defining it based on the guideline's description of appropriate candidates for topical therapy (carcinoma in situ, Ta, and T1). This addresses the reviewer's critique of unclear wording by making the recommendation more specific, while remaining faithful to the source text's assertion of ineffectiveness for advanced disease. | False | True |

# Section 11: Repeat Expert Ratings of Failed Items and Final Delphi-Ready Candidate Item List

## Repeat Expert Ratings of Failed Items

| **Item** | **Expert 1** | **Expert 2** | **Expert 3** | **Expert 4** | **Median** | **IQR** |
| --- | --- | --- | --- | --- | --- | --- |
| **G13 original** | 5 | 3 | 3 | 4 | 3.5 | 3-4.25 |
| **G13 revised** | 5 | 5 | 4 | 5 | 5 | 4.5-5 |

## Final Delphi-Ready Candidate Item List After External Expert Appraisal and AI-Assisted Revision of Failed Items

| **Item** | **Origin** | **Category** | **Candidate Item** |
| --- | --- | --- | --- |
| G01 | Guideline-derived item | Diagnosis & Staging | Perform magnetic resonance imaging (MRI) or penile ultrasound (US) of the primary tumor when there is uncertainty regarding corporal invasion (T3) and/or the feasibility of organ-sparing surgery. |
| G02 | Guideline-derived item | Diagnosis & Staging | Computerized tomography (CT) of the chest, abdomen, and pelvis is performed to evaluate for distant metastatic spread. For high-risk or node-positive disease, ¹⁸F-FDG-PET/CT can also be used. |
| G03 | Guideline-derived item | Diagnosis & Staging | Cross-sectional imaging (CT, PET/CT) for primary staging should generally be reserved for T3/T4 tumors or in cases of positive lymph node status. |
| G04 | Guideline-derived item | Diagnosis & Staging | For superficial, non-invasive penile carcinomas (Carcinoma in situ), a fluorescence-diagnostic technique can be used to better determine the extent of the lesion. |
| G05 | Guideline-derived item | Diagnosis & Staging | For clinically node-negative (cN0) low-risk tumors (PeIN, pTa, pT1a G1), surveillance of the inguinal lymph nodes is recommended instead of operative staging. |
| G06 | Guideline-derived item | Diagnosis & Staging | For clinically node-negative (cN0) intermediate-risk tumors (T1a G2), surveillance may be discussed as an alternative to surgical staging with patients who are willing to comply with strict follow-up. |
| G07 | Guideline-derived item | Diagnosis & Staging | The preferred method for surgical staging in cN0 patients is dynamic sentinel node biopsy (DSNB). If DSNB is unavailable or not feasible, modified inguinal lymph node dissection (ILND) is an alternative. If DSNB is positive, a completion ILND is recommended. |
| G08 | Guideline-derived item | Diagnosis & Staging | If DSNB is planned, perform inguinal ultrasound (US) first, with fine needle aspiration cytology (FNAC) of any sonographically abnormal lymph nodes. |
| G09 | Guideline-derived item | Diagnosis & Staging | In cN0 patients undergoing staging, frozen section analysis should not be performed on tissue from a modified inguinal lymphadenectomy or DSNB. |
| G10 | Guideline-derived item | Diagnosis & Staging | For clinically node-positive (cN+) disease, an (image-guided) biopsy of the suspicious node is required to confirm metastasis before initiating treatment. |
| G11 | Guideline-derived item | Primary Treatment (General Principles) | The goal of surgical treatment is complete excision with negative margins (R0). A margin of 5 mm for G1/G2 tumors and 10 mm for G3 tumors is suggested. In case of a positive surgical margin, a re-excision should be performed. |
| G12 | Guideline-derived item | Primary Treatment (Topical Therapy) | If topical treatment fails, it should not be repeated. |
| G13 | Guideline-derived item | Primary Treatment (Topical Therapy) | For patients with penile cancer, topical therapy is an ineffective treatment for advanced stages of the disease (i.e., beyond T1). |
| G14 | Guideline-derived item | Primary Treatment (Laser Therapy) | A surgical margin of 5 mm is considered oncologically safe for laser therapy. |
| G15 | Guideline-derived item | Primary Treatment (Post-Therapy Biopsy) | After topical or laser therapy, a control biopsy should be performed to verify local tumor control, especially in cases of clinical doubt. |
| G16 | Guideline-derived item | Primary Treatment (Surgical) | Organ-sparing surgery options (circumcision, wide local excision, glansectomy, Mohs) are offered for lesions confined to the glans and prepuce (PeIN, Ta, T1-T2) in patients willing to comply with strict follow-up. |
| G17 | Guideline-derived item | Primary Treatment (Surgical) | Intra-operative frozen section analysis of resection margins should be performed to confirm negative margins, especially in cases of doubt. |
| G18 | Guideline-derived item | Primary Treatment (Surgical) | When using radiotherapy or topical therapy for a preputial lesion, a circumcision should be performed first. |
| G19 | Guideline-derived item | Primary Treatment (Surgical) | Wide local excision (WLE) is an option for early-stage tumors (PeIN, Ta, T1). Complete excision with wide negative margins is required. |
| G20 | Guideline-derived item | Primary Treatment (Surgical) | Mohs micrographic surgery can be considered as an alternative to WLE for select, low-grade, superficial lesions, particularly on the glans or distal penis, if the appropriate expertise is available. |
| G21 | Guideline-derived item | Primary Treatment (Surgical) | Glansectomy (partial or total) or glans resurfacing are options for tumors located on the glans penis (PeIN, Ta, T1-T2). Reconstruction may be performed with a split-thickness skin graft. |
| G22 | Guideline-derived item | Primary Treatment (Radiotherapy) | For T3–4 or N+ surgically unresectable disease, consider EBRT with concurrent chemotherapy to the penis and regional lymph nodes. |
| G23 | Guideline-derived item | Primary Treatment (Radiotherapy) | Specific radiotherapy doses may include: • EBRT: 66-74 Gy in 2 Gy fractions. • Brachytherapy (HDR): 42-45 Gy in 12-14 fractions. • Brachytherapy (LDR/PDR): 60-65 Gy delivered continuously over ~5 days. |
| G24 | Guideline-derived item | Primary Treatment (Multimodal) | For non-resectable, locally advanced primary tumors (T4), consider induction/neoadjuvant chemotherapy followed by surgery for responders, or chemo-radiotherapy. |
| G25 | Guideline-derived item | Recurrent Disease | After radiotherapy, local recurrence should be treated with surgical salvage therapy. |
| G26 | Guideline-derived item | Recurrent Disease | After topical or laser therapy, local recurrence should be treated with a different treatment modality. |
| G27 | Guideline-derived item | Recurrent Disease | Regional lymph node recurrence after initial surveillance should be managed according to the same principles as primary lymph node metastases (i.e., with ILND +/- systemic therapy). |
| G28 | Guideline-derived item | Recurrent Disease | Regional lymph node recurrence after a prior radical lymphadenectomy should be managed with a multimodal approach, including neoadjuvant chemotherapy, surgery, and/or radiotherapy. |
| G29 | Guideline-derived item | Lymph Node Management | Following circumcision for a primary tumor, patients with moderately or poorly differentiated tumors (grades 2 and 3) will require inguinal node dissection. |
| G30 | Guideline-derived item | Lymph Node Management | For palpable, mobile inguinal nodes (cN1-cN2), inguinal lymph node dissection (ILND) is recommended. The dissection may be fascial-sparing or radical, with preservation of the saphenous vein where possible. |
| G31 | Guideline-derived item | Lymph Node Management | If intraoperatively suspicious nodes are removed during surgery for cN+ disease, frozen section analysis should be performed. If positive, an ipsilateral radical lymphadenectomy should be completed in the same session. |
| G32 | Guideline-derived item | Lymph Node Management | For bulky, fixed, or extensive inguinal nodes (cN3), neoadjuvant chemotherapy (NAC) with a cisplatin- and taxane-based regimen is preferred over up-front surgery. |
| G33 | Guideline-derived item | Lymph Node Management | Minimally-invasive ILND for cN1–2 disease should only be offered as part of a clinical trial. It is contraindicated for cN3 disease. |
| G34 | Guideline-derived item | Lymph Node Management | Following a response to neoadjuvant chemotherapy, patients should undergo consolidative surgery, including radical inguinal lymphadenectomy and possible pelvic lymphadenectomy. |
| G35 | Guideline-derived item | Lymph Node Management | Ipsilateral pelvic lymph node dissection (PLND) is recommended for patients with ≥2-3 positive inguinal nodes on one side or with extranodal extension. |
| G36 | Guideline-derived item | Systemic Therapy | Recommended neoadjuvant chemotherapy regimens are cisplatin- and taxane-based triplets, such as TIP (Paclitaxel/Ifosfamide/Cisplatin) or TPF (Cisplatin/Paclitaxel/5-Fluorouracil). |
| G37 | Guideline-derived item | Systemic Therapy | Adjuvant chemotherapy should be considered for patients with pN2 or pN3 disease following lymph node dissection. |
| G38 | Guideline-derived item | Systemic Therapy | Adjuvant radiotherapy (with or without chemo-sensitization) should be offered or considered for patients with high-risk pathological findings such as pN2/N3 disease or extranodal extension. |
| G39 | Guideline-derived item | Systemic Therapy | Definitive radiotherapy (with or without chemo sensitization) can be offered to patients who are unwilling or unable to undergo surgery. |
| G40 | Guideline-derived item | Systemic Therapy | For first-line treatment of metastatic disease, platinum-based combination chemotherapy is the preferred approach. Recommended regimens include TIP, TPF, or cisplatin + 5-FU. |
| G41 | Guideline-derived item | Systemic Therapy | Bleomycin-containing chemotherapy regimens should not be used due to the risk of pulmonary toxicity. |
| G42 | Guideline-derived item | Systemic Therapy | For patients with progressive disease, enrollment in a clinical trial is the preferred option for subsequent-line therapy. |
| G43 | Guideline-derived item | Systemic Therapy | Subsequent-line therapy options may include paclitaxel monotherapy or, for biomarker-selected tumors (MSI-H, dMMR, TMB-H), immunotherapy such as pembrolizumab. |
| G44 | Guideline-derived item | Palliative & Supportive Care | Patients with metastatic disease or requiring multimodal therapy should be discussed in an interdisciplinary tumor board. |
| G45 | Guideline-derived item | Palliative & Supportive Care | Palliative radiotherapy can be offered for symptom control in advanced disease (e.g., 30 Gy in 10 fractions). |
| G46 | Guideline-derived item | Palliative & Supportive Care | For chronic lymphedema resulting from treatment, a complex decongestive therapy (lymphatic drainage, compression therapy, and skin care) should be performed. |
| G47 | Guideline-derived item | Follow-up & Survivorship | A general follow-up schedule is every 3-4 months for the first 2 years, then less frequently. Follow-up should extend for at least 5 years. |
| G48 | Guideline-derived item | Follow-up & Survivorship | Specific follow-up schedules after laser therapy are: • CO2 Laser: After 2 months, then every 3 months for 3 years, then biannually. • Nd:YAG Laser: Every 3 months for 2 years, then biannually. |
| G49 | Guideline-derived item | Follow-up & Survivorship | Specific follow-up schedules based on treatment: • Organ Preservation: Every 3 mo (Yrs 1-2), every 6 mo (Yrs 3-5). • Total Penectomy: Every 3 mo (Yrs 1-2), annually (Yrs 3-5). |
| G50 | Guideline-derived item | Follow-up & Survivorship | Patients should be taught self-examination for early detection of recurrence and given easy access back to the clinic if concerns arise. |
| G51 | Guideline-derived item | Follow-up & Survivorship | Penile cancer care should be delivered by an extended multi-disciplinary team including specialists in urology, oncology, nursing, pathology, radiology, psychology, and lymphedema therapy. |
| G52 | Guideline-derived item | Follow-up & Survivorship | Patients should be offered psychological support and counseling, and physicians should discuss the psychological and functional (urinary, sexual) impact of the disease and its treatments. |
| G53 | Guideline-derived item | Follow-up & Survivorship | Patients should be assessed for lymphedema at follow-up and referred to lymphedema therapists early if it develops. |
| W01 | Literature-derived true white-area item | General Principles / Management | For patients with locally advanced disease who are kidney transplant recipients, treatment should be individualized to minimize graft injury, preferring non-cytotoxic options over radiotherapy and cytotoxic treatments. |
| W02 | Literature-derived true white-area item | Cancer Prevention | The use of male condoms is recommended as the most effective method for reducing the transmission of Sexually Transmitted Infections (STIs) like HPV and HIV, which are established risk factors for penile cancer. |
| W03 | Literature-derived true white-area item | Complication Management | Primary Penile Surgery Complications: • Surgical Site Infections (SSIs): Manage with empirical antibiotics, transitioning to culture-specific therapy, along with regular wet-to-dry dressing changes. • Meatal Stenosis: Manage with self-catheterization or formal surgical revision.• Partial Skin Graft Loss: Manage expectantly with observation. • Total Skin Graft Loss: Manage with wet-to-dry dressing changes followed by delayed regrafting. |
| W04 | Literature-derived true white-area item | Complication Management | Inguinal Surgery Complications - Wound Issues: • Infected Inguinal Wounds: Manage with meticulous wound care, daily inspection, and culture-specific antibiotics. • Wound Dehiscence / Flap Necrosis: Manage with wet-to-dry dressings or vacuum-assisted closure (VAC) dressings, followed by delayed closure. • Patients with Large Body Habitus: Place dry gauze ("Interdry") in groin folds to prevent moisture buildup. |
| W05 | Literature-derived true white-area item | Lymph Node Management | The management of inguinal nodal disease in transplanted patients should be consistent with standard oncological guidelines. |
| W06 | Literature-derived true white-area item | Perioperative Care | Strict diabetes control is crucial for avoiding wound infection-related adverse events in patients undergoing ILND. |
| W07 | Literature-derived true white-area item | Postoperative Care | Primary Penile Surgery - Wound and Catheter Management: • Dressing: Use compressive surgical dressings for 2-7 days. • Foley Catheter: Typically remove around 7 days post-surgery for patients who undergo neomeatus creation or STSG application. |
| W08 | Literature-derived true white-area item | Screening | General screening protocols for penile cancer are not recommended in kidney transplant recipients. |
| W09 | Literature-derived true white-area item | Systemic Therapy (Targeted / Immunotherapy - Investigational) | For patients with NOTCH1-mutated tumors, enrollment in clinical trials focused on PI3K/mTOR inhibitors is suggested. |

# Section 12: Planned Downstream Delphi Voting Procedure

The present Tutorial concludes with the generation and independent external appraisal of Delphi-ready candidate items. Any subsequent Delphi consensus process should be conducted as a separate, prospectively specified study. Before voting begins, investigators should define panel eligibility criteria, required expertise, disciplinary representation, panel size, recruitment procedures, measures to ensure independence, and conflict-of-interest management. The involvement and roles of any additional stakeholder groups, including whether they participate in voting or serve in an advisory capacity, should likewise be specified in advance.

Voting should be anonymous and conducted using a secure survey platform, with additional rounds performed as required. Panelists may rate each item on a nine-point Likert scale, with scores of 1–3 indicating disagreement, 4–6 uncertainty or partial agreement, and 7–9 agreement (Supplementary Table 1). Consensus criteria should be predefined and may include the proportion of ratings within a specified zone, the median, and the interquartile range. Items not meeting the threshold may proceed to subsequent rounds accompanied by anonymized group-level feedback. Any clarification or reformulation of items between rounds should be documented and preserve their intended clinical meaning.

Stopping rules should also be specified prospectively and may include achievement of consensus, response stability, completion of a predefined maximum number of rounds, or lack of further convergence. The subsequent Delphi study should be reported separately, including its panel composition, voting procedures, consensus criteria, feedback process, stopping rules, and handling of item modifications. Any resulting consensus should be interpreted as the outcome of that downstream clinical adjudication process and not as a finding of the present item-preparation study.

# Table S1

Nine-Point Likert scale

| **Category** | **Score** | **Definition** |
| --- | --- | --- |
| **Disagree** | **1** | **Strongly disagree:**  I completely reject this statement.  The recommendation is clearly inappropriate, unsupported by evidence, or clinically misleading. |
|  | **2** | **Disagree:**  I mostly disagree with this statement.  There are major concerns regarding its validity, relevance, or practical applicability. |
|  | **3** | **Slightly disagree:**  I tend to disagree with this statement.  While some aspects may be acceptable, overall the recommendation is not convincing. |
| **Neutral / indifferent** | **4** | **Leaning toward disagreement:**  I have reservations and am slightly inclined to disagree.  The evidence or rationale appears weak or inconsistent. |
|  | **5** | **Neutral:**  I am undecided.  The available information is insufficient or conflicting to form a clear judgment. |
|  | **6** | **Leaning toward agreement:**  I have minor concerns but generally tend to agree.  The recommendation is plausible but not fully convincing. |
| **Agree** | **7** | **Slightly agree:**  I generally agree with this statement.  The recommendation is reasonable, though some minor issues remain. |
|  | **8** | **Agree:**  I agree with this statement.  The recommendation is well supported by evidence and clinically appropriate. |
|  | **9** | **Strongly agree:**  I fully endorse this statement.  The recommendation is highly relevant, evidence-based, and clearly applicable in clinical practice. |

# Table S2

CHART Checklist: Chatbot Assessment Reporting Tool

| **CHART Section** | **Item** | **CHART Item description** | **Reported (Yes / Partial / No / N/A)** | **Location in Manuscript (Section)** |
| --- | --- | --- | --- | --- |
| Title / Abstract | 1a | Identification of the study as involving a chatbot or generative AI system | Yes | Title; Abstract |
| Title / Abstract | 1b | Description of the chatbot/AI purpose in the abstract | Yes | Abstract – Methods |
| Background | 2a | Rationale for using a chatbot or generative AI system | Yes | Introduction |
| Background | 2b | Intended clinical or research context of use | Yes | Introduction; Materials and Methods – **Tutorial Aim, Scope, and Intended Use** |
| AI System Description | 3a | Name and version of the chatbot or generative AI model | Yes | Materials and Methods – LLM Selection |
| AI System Description | 3b | Model developer and access modality | Yes | Materials and Methods – LLM Selection |
| Model Characteristics | 4a | Description of model characteristics and capabilities | Yes | Materials and Methods – LLM Selection; Limitations |
| Model Characteristics | 4b | Description of training data or known training limitations | Partial | Materials and Methods – LLM Selection;  Model training data are proprietary; model-specific limitations and the benchmark-based selection rationale are reported. |
| Model Characteristics | 4c | Model update or version control | Yes | Materials and Methods – LLM Selection |
| Prompting and Inputs | 5a | Description of prompt structure and design | Yes | Materials and Methods – Multimodal Preparation and AI-Based Content Interpretation; Matrix Construction and Guideline Stance Classification;  Supplementary Materials and public repository |
| Prompting and Inputs | 5b | Changes to prompts during the study | N/A | Tutorial – no iterative prompt optimisation reported |
| Query Strategy | 6a | Description of query strategy | Yes | Materials and Methods – Multimodal Preparation and AI-Based Content Interpretation; Matrix Construction and Guideline Stance Classification; LLM ensemble consistency check |
| Query Strategy | 6b | Number and frequency of queries | Yes | Materials and Methods – LLM Selection; Matrix Construction and Guideline Stance Classification; LLM ensemble consistency check; public repository. |
| Query Strategy | 6c | Handling of repeated or conflicting outputs | Yes | Materials and Methods – LLM ensemble consistency check |
| Query Strategy | 6d | Human involvement in query handling | No | No human query handling was used or reported. The consistency check was performed by an ensemble of three LLMs; independent experts appraised the final candidate items downstream rather than handling model queries. |
| Performance Evaluation | 7a | Metrics used to assess chatbot/AI performance | Partial | Results – Ensemble Consistency Check of Classification and Model Concordance; External Expert Appraisal of Candidate-Item Suitability; Limitations. Inter-model concordance and item-suitability ratings were reported, but no classification-accuracy metric against an independent reference standard was calculated. |
| Performance Evaluation | 7b | Description of evaluation process | Partial | Materials and Methods – LLM ensemble consistency check; External Expert Appraisal of Delphi-Ready Candidate Items; Limitations. These procedures assessed model consistency and downstream item suitability, not formal AI performance against an independent reference standard. |
| Sample Size | 8 | Number of evaluated samples or outputs | Yes | Results – Overview of Included Guidelines and Extracted Content; Identification of White Areas; Finalization of the Delphi Item Set; External Expert Appraisal of Candidate-Item Suitability |
| Data Analysis | 9a | Description of analytical methods | Yes | Materials and Methods – Harmonization and Semantic Consolidation of Recommendations; Matrix Construction and Guideline Stance Classification; LLM ensemble consistency check; Identification and Processing of White Areas; External Expert Appraisal of Delphi-Ready Candidate Items. |
| Results | 10a | Primary results related to AI outputs | Yes | Results – Overview of Included Guidelines and Extracted Content; Thematic Harmonization and Construction of the Recommendation Matrix; Ensemble Consistency Check of Classification and Model Concordance; Identification of White Areas; Finalization of the Delphi Item Set; External Expert Appraisal of Candidate-Item Suitability |
| Results | 10b | Secondary or subgroup analyses | N/A | No subgroup analysis of AI outputs |
| Results | 10c | Error analysis or failure cases | Partial | Results – Ensemble Consistency Check of Classification and Model Concordance; External Expert Appraisal of Candidate-Item Suitability; Limitations; Supplementary Materials 4-5 and 10-11. Model disagreements, corrected classifications, and the failed/revised item were documented, but no formal error analysis against an independent human reference standard was performed. |
| Discussion | 11a | Interpretation of findings related to AI use | Yes | Discussion |
| Discussion | 11b | AI-specific limitations | Yes | Limitations |
| Discussion | 11c | Implications for future research or practice | Yes | Discussion; Conclusion |
| Other Information | 12a | Ethics approval and consent | Yes | Materials and Methods – Ethics Approval |
| Other Information | 12b | Protocol availability | No | No prospectively registered protocol existed. |
| Other Information | 12c | Data availability | Yes | Data availability |
| Other Information | 12d | Funding sources | Yes | Funding |
| Other Information | 12e | Competing interests | Yes | Competing interests |

# References

[1]	A. Sachdeva *et al.*, “Management of Lymph Node–positive Penile Cancer: A Systematic Review,” Mar. 01, 2024, *Elsevier B.V.* doi: 10.1016/j.eururo.2023.04.018.

[2]	A. Piras *et al.*, “Stereotactic Radiotherapy for Penile Metastasis: Case Report and Systematic Literature Review,” *Oncol. Res. Treat.*, vol. 47, no. 11, pp. 565–572, Nov. 2024, doi: 10.1159/000539275.

[3]	H. Taghizadeh and H. Fajkovic, “Immunotherapy in the Management of Penile Cancer—A Systematic Review,” Mar. 01, 2025, *Multidisciplinary Digital Publishing Institute (MDPI)*. doi: 10.3390/cancers17050883.

[4]	K. H. Pang, A. Muneer, and H. M. Alnajjar, “Glansectomy and Reconstruction for Penile Cancer: A Systematic Review,” Sep. 01, 2022, *Elsevier B.V.* doi: 10.1016/j.euf.2021.11.008.

[5]	G. Basile *et al.*, “Testis and penile cancers in kidney transplant recipients: A systematic review of epidemiology, treatment options and oncological outcomes by the EAU-YAU Penile and Testis Cancer Working Group,” Apr. 01, 2025, *Elsevier Ltd*. doi: 10.1016/j.acuro.2025.501683.

[6]	J. S. Smith *et al.*, “Male circumcision reduces penile hpv incidence and persistence: A randomized controlled trial in Kenya,” *Cancer Epidemiology Biomarkers and Prevention*, vol. 30, no. 6, pp. 1139–1148, Jun. 2021, doi: 10.1158/1055-9965.EPI-20-1272.

[7]	S. B. Shapiro, C. Laurie, M. El-Zein, and E. L. Franco, “Association between male circumcision and human papillomavirus infection in males and females: a systematic review, meta-analysis, and meta-regression,” Aug. 08, 2022. doi: 10.1101/2022.08.08.22278357.

[8]	F. Crocetto *et al.*, “Impact of sexual activity on the risk of male genital tumors: A systematic review of the literature,” Aug. 02, 2021, *MDPI AG*. doi: 10.3390/ijerph18168500.

[9]	L. L. da Silva *et al.*, “Malignancy Associated with Low-Risk HPV6 and HPV11: A Systematic Review and Implications for Cancer Prevention,” Aug. 01, 2023, *Multidisciplinary Digital Publishing Institute (MDPI)*. doi: 10.3390/cancers15164068.

[10]	A. Sood, J. K. Rudzinski, P. E. Spiess, and C. A. Pettaway, “The Acute Complications After Surgery for Penile Carcinoma and Strategies for Their Management: A Systematic Review of the Literature,” *Semin. Oncol. Nurs.*, vol. 38, no. 3, Jun. 2022, doi: 10.1016/j.soncn.2022.151285.

[11]	A. F. Dagi, N. E. Jones, and J. T. Bogue, “The Role of Plastic Surgery in Urologic Cancer and Trauma Reconstruction: A Systematic Review,” *Ann. Plast. Surg.*, vol. 95, no. 5, pp. 590–591, Nov. 2025, doi: 10.1097/SAP.0000000000004500.

[12]	K. Parza *et al.*, “The Prognostic Role of Human Papillomavirus and p16 Status in Penile Squamous Cell Carcinoma—A Systematic Review,” Jul. 01, 2023, *Multidisciplinary Digital Publishing Institute (MDPI)*. doi: 10.3390/cancers15143713.

[13]	A. Mustasam *et al.*, “The Prognostic Role of HPV or p16INK4a Status in Penile Squamous Cell Carcinoma: A Meta-Analysis,” *JNCCN Journal of the National Comprehensive Cancer Network*, vol. 23, no. 2, Feb. 2025, doi: 10.6004/jnccn.2024.7078.

[14]	D. I. Jo, S. H. Han, S. H. Kim, H. Y. Kim, H. Chung, and H. S. Kim, “Optimal treatment for penile verrucous carcinoma: a systematic literature review,” *BMC Urol.*, vol. 21, no. 1, Dec. 2021, doi: 10.1186/s12894-020-00777-1.

[15]	S. Krishna, N. Schieda, G. S. Kulkarni, K. Shanbhogue, R. H. Baroni, and S. Woo, “Diagnostic Accuracy of MRI in Local Staging (T Category) of Penile Cancer and the Value of Artificial Erection: A Systematic Review and Meta-Analysis,” Jul. 01, 2022, *American Roentgen Ray Society*. doi: 10.2214/AJR.21.27063.

[16]	K. H. Pang, H. M. Alnajjar, and A. Muneer, “Functional outcomes of glansectomy to treat localised penile cancer: a systematic review,” 2025, *Springer Nature*. doi: 10.1038/s41443-025-01062-1.

[17]	D. S. Zekan, A. Dahman, A. J. Hajiran, A. M. Luchey, J. Chahoud, and P. E. Spiess, “Prognostic predictors of lymph node metastasis in penile cancer: a systematic review,” *International Braz J Urol*, vol. 47, no. 5, pp. 943–956, Sep. 2021, doi: 10.1590/S1677-5538.IBJU.2020.0959.

[18]	A. Issa *et al.*, “Treatment Options and Outcomes for Men with Penile Intraepithelial Neoplasia: A Systematic Review,” May 01, 2022, *Elsevier B.V.* doi: 10.1016/j.euf.2021.04.026.

[19]	G. Fallara *et al.*, “Diagnostic Accuracy of Dynamic Sentinel Lymph Node Biopsy for Penile Cancer: A Systematic Review and Meta-analysis,” May 01, 2023, *Elsevier B.V.* doi: 10.1016/j.euf.2022.11.018.

[20]	M. Z. Yunis, K. H. Pang, A. Muneer, and H. M. Alnajjar, “Intraoperative frozen section examination for penile cancer surgery: a systematic review,” Sep. 01, 2025, *Springer Nature*. doi: 10.1038/s41443-025-01024-7.

[21]	F. L. Sand, S. Lindquist, G. L. Aalborg, and S. K. Kjaer, “The prognostic value of p53 and Ki-67 expression status in penile cancer: a systematic review and meta-analysis,” Apr. 01, 2025, *Elsevier B.V.* doi: 10.1016/j.pathol.2024.11.006.

[22]	A. Soikkeli-Jalonen, S. Vierelä, A. Kaipia, E. Harju, and E. Haavisto, “Psychometric properties and content of instruments for assessing penile cancer patients’ quality of life: A systematic review,” Dec. 01, 2025, *Elsevier B.V.* doi: 10.1016/j.ijnsa.2025.100412.

[23]	E. Whyte *et al.*, “Effects of partial penectomy for penile cancer on sexual function: A systematic review,” Sep. 01, 2022, *Public Library of Science*. doi: 10.1371/journal.pone.0274914.

[24]	X. Yan *et al.*, “Near-infrared fluorescence-assisted superficial inguinal lymph-node excision for low-risk penile cancer,” *World J. Urol.*, vol. 42, no. 1, Dec. 2024, doi: 10.1007/s00345-024-04877-7.

[25]	K. N. Patel, A. Salunke, G. Bakshi, D. Jayaprakash, and S. J. Pandya, “Robotic-Assisted Video-Endoscopic Inguinal Lymphadenectomy (RAVEIL) and Video-Endoscopic Inguinal Lymphadenectomy (VEIL) versus Open Inguinal Lymph-Node Dissection (OILND) in carcinoma of penis: Comparison of perioperative outcomes, complications and oncological outcomes. A systematic review and meta-analysis,” *Urologic Oncology: Seminars and Original Investigations*, vol. 40, no. 3, pp. 112.e11-112.e22, Mar. 2022, doi: 10.1016/j.urolonc.2021.11.010.

[26]	G. E. Cacciamani *et al.*, “Assessment and Reporting of Perioperative Adverse Events and Complications in Patients Undergoing Inguinal Lymphadenectomy for Melanoma, Vulvar Cancer, and Penile Cancer: A Systematic Review and Meta-analysis,” Apr. 01, 2023, *Springer Science and Business Media Deutschland GmbH*. doi: 10.1007/s00268-022-06882-6.

[27]	S. C. Schmid *et al.*, “Final results of the PräVAC trial: prevention of wound complications following inguinal lymph node dissection in patients with penile cancer using epidermal vacuum-assisted wound closure,” *World J. Urol.*, vol. 39, no. 2, pp. 613–620, Feb. 2021, doi: 10.1007/s00345-020-03221-z.

[28]	J. Bowie *et al.*, “A systematic review of non-hpv prognostic biomarkers used in penile squamous cell carcinoma,” Sep. 01, 2021, *AVES*. doi: 10.5152/tud.2021.21199.

[29]	S. Ge *et al.*, “Comparing the safety and effectiveness of minimally invasive surgery and open inguinal lymph node dissection in penile cancer: A systematic review and meta-analysis,” *European Journal of Surgical Oncology*, vol. 50, no. 10, Oct. 2024, doi: 10.1016/j.ejso.2024.108553.

[30]	P. Yuan *et al.*, “Efficacy of indocyanine green fluorescence-guided inguinal lymph node dissection for penile cancer: a randomised trial,” *BJU Int.*, vol. 133, no. 4, pp. 442–450, Apr. 2024, doi: 10.1111/bju.16231.

[31]	I. Greco *et al.*, “Systematic Review and Meta-analysis of Minimally Invasive Procedures for Surgical Inguinal Nodal Staging in Penile Carcinoma,” Jul. 01, 2024, *Elsevier B.V.* doi: 10.1016/j.euf.2023.11.010.

[32]	S. W. Lee and S. J. Kim, “Diagnostic Performance of 18F-FDG PET/CT for Lymph Node Staging in Penile Cancer,” *Clin. Nucl. Med.*, vol. 47, no. 5, pp. 402–408, May 2022, doi: 10.1097/RLU.0000000000004081.

[33]	C. V. Suartz *et al.*, “Comparing open and video endoscopic lymphadenectomy for penile cancer: a systematic review and meta-analysis of prospective studies,” Apr. 01, 2025, *John Wiley and Sons Inc*. doi: 10.1111/bju.16661.

[34]	N. Naushad, A. A. Deb, A. A. Agag, H. A. Serag, and V. K. Sangar, “Pelvic lymph node dissection alone versus adjuvant radiotherapy in node positive penile cancer: A systematic review,” Apr. 01, 2022, *Wolters Kluwer Medknow Publications*. doi: 10.4103/iju.iju_453_21.

[35]	Y. G. Tan *et al.*, “Comparing the Perioperative and Oncological Outcomes of Open Versus Minimally Invasive Inguinal Lymphadenectomy in Penile Cancer: A Systematic Review and Meta-Analysis,” Sep. 01, 2025, *Multidisciplinary Digital Publishing Institute (MDPI)*. doi: 10.3390/cancers17183035.

[36]	R. Mehta, S. Kumar, and R. Sah, “A comment on: ‘Comparing open and video endoscopic lymphadenectomy for penile cancer: a systematic review and meta-analysis of prospective studies,’” Aug. 01, 2025, *John Wiley and Sons Inc*. doi: 10.1111/bju.16792.

[37]	P. Ravisankar, K. Malik, A. Raja, and K. Narayanaswamy, “Clipping inguinal lymphatics decreases lymphorrhoea after lymphadenectomy following cancer treatment: results from a randomized clinical trial,” *Scand. J. Urol.*, vol. 55, no. 6, pp. 480–485, 2021, doi: 10.1080/21681805.2021.1980096.

[38]	F. Fadigas, D. Martins, and F. Mendes, “Immunotherapy in Penile Cancer: A Systematic Review,” Dec. 28, 2024, *Iniestares, S.A.* doi: 10.56434/j.arch.esp.urol.20247710.154.

[39]	K. Vandermaesen *et al.*, “The prognostic role of HPV status in penile squamous cell carcinoma: a systematic review and meta-analysis,” Sep. 01, 2025, *Springer Nature*. doi: 10.1038/s41443-025-01145-z.

[40]	V. I. Sakalis *et al.*, “What Is the Most Effective Management of the Primary Tumor in Men with Invasive Penile Cancer: A Systematic Review of the Available Treatment Options and Their Outcomes,” Jun. 01, 2022, *Elsevier B.V.* doi: 10.1016/j.euros.2022.04.002.

[41]	S. M. Croghan, I. M. Cullen, and O. Raheem, “Functional outcomes and health-related quality of life following penile cancer surgery: a comprehensive review,” Oct. 01, 2023, *Oxford University Press*. doi: 10.1093/sxmrev/qead021.

[42]	S. Yadlapati, P. M. Rosa-Nieves, K. C. Lauck, and S. N. Tolkachjov, “Mohs Micrographic Surgery Versus Wide Local Excision in the Treatment of Anogenital Squamous Cell Carcinoma: A Systematic Review,” Jun. 01, 2025, *John Wiley and Sons Inc*. doi: 10.1111/ijd.17689.

[43]	G. Fallara *et al.*, “Oncological and Functional Outcomes of Penile Shaft Sparing Surgery for Localised Penile Cancer: A Systematic Review,” Jan. 01, 2025, *Elsevier B.V.* doi: 10.1016/j.euf.2024.05.004.

[44]	J. F. Paz Rojas, C. A. Ballestas Almario, and H. A. García-Perdomo, “Effectiveness and safety of adjuvant chemotherapy compared to neoadjuvant chemotherapy in patients with penile cancer and positive lymph nodes regarding overall survival and free disease survival: a systematic review and meta-analysis,” *Urologic Oncology: Seminars and Original Investigations*, vol. 40, no. 5, pp. 200.e11-200.e18, May 2022, doi: 10.1016/j.urolonc.2022.02.014.

[45]	Y. Lu, Y. Wang, H. Su, and H. Li, “PD-L1 is associated with the prognosis of penile cancer: A systematic review and meta-analysis,” Nov. 30, 2022, *Frontiers Media S.A.* doi: 10.3389/fonc.2022.1013806.

[46]	I. Ribera-Cortada *et al.*, “Pathogenesis of penile squamous cell carcinoma: Molecular update and systematic review,” Jan. 01, 2022, *MDPI*. doi: 10.3390/ijms23010251.

[47]	S. Babadi *et al.*, “Relationship between NLR and penile squamous cell carcinoma: a systematic review and meta-analysis,” *BMC Urol.*, vol. 24, no. 1, Dec. 2024, doi: 10.1186/s12894-024-01569-7.

[48]	J. G. Porto *et al.*, “The Influence of the Microbiome on Urological Malignancies: A Systematic Review,” Oct. 01, 2023, *Multidisciplinary Digital Publishing Institute (MDPI)*. doi: 10.3390/cancers15204984.

[49]	C. J. Catalfamo, H. E. Brown, and L. K. Dennis, “Evaluating the Strength of Association of Human Papillomavirus Infection With Penile Carcinoma: A Meta-Analysis,” *Sex. Transm. Dis.*, vol. 49, no. 5, pp. 368–376, May 2022, doi: 10.1097/OLQ.0000000000001606.

[50]	E. Crupi *et al.*, “Nectin-4 Positivity in Genitourinary Malignancies: A Systematic Review,” *JCO Precis. Oncol.*, no. 8, Dec. 2024, doi: 10.1200/po-24-00470.

[51]	P. Maisch, F. Koll, C. Bolenz, F. K. H. Chun, J. E. Gschwend, and S. C. Schmid, “Combination of radiation and immunotherapy in the treatment of genitourinary malignancies: A systematic review and meta-analysis,” May 01, 2023, *Elsevier Inc.* doi: 10.1016/j.urolonc.2022.10.009.

[52]	H. M. Saputra, F. Hidayatullah, Y. P. Kloping, J. Renaldo, E. Chung, and L. Hakim, “Prognostic value of neutrophil-to-lymphocyte ratio (NLR) in penile cancer: A systematic review and meta-analysis,” Sep. 01, 2022, *Elsevier Ltd*. doi: 10.1016/j.amsu.2022.104335.

[53]	S. Sethi, “Defining the Molecular Intricacies of Human Papillomavirus-Associated Tonsillar Carcinoma,” Jan. 01, 2025, *SAGE Publications Ltd*. doi: 10.1177/10732748241310932.

[54]	C. Rosado, Â. R. Fernandes, A. G. Rodrigues, and C. Lisboa, “Impact of Human Papillomavirus Vaccination on Male Disease: A Systematic Review,” Jun. 01, 2023, *MDPI*. doi: 10.3390/vaccines11061083.

[55]	P. J. Lou *et al.*, “Systematic literature review on the clinical and economic burden of human papillomavirus–related diseases in select areas in the Asia-Pacific region,” *Hum. Vaccin. Immunother.*, vol. 20, no. 1, 2024, doi: 10.1080/21645515.2024.2425535.

[56]	A. A. Dinesh, S. Helena Pagani Soares Pinto, O. Brunckhorst, P. Dasgupta, and K. Ahmed, “Anxiety, depression and urological cancer outcomes: A systematic review,” Dec. 01, 2021, *Elsevier Inc.* doi: 10.1016/j.urolonc.2021.08.003.

[57]	R. Linertová, C. Guirado-Fuentes, J. Mar Medina, I. Imaz-Iglesia, L. Rodríguez-Rodríguez, and M. Carmona-Rodríguez, “Cost-effectiveness of extending the HPV vaccination to boys: A systematic review,” Sep. 01, 2021, *BMJ Publishing Group*. doi: 10.1136/jech-2020-216305.

[58]	S. Balachandra *et al.*, “Blood-based biomarkers of human papillomavirus–associated cancers: A systematic review and meta-analysis,” *Cancer*, vol. 127, no. 6, pp. 850–864, Mar. 2021, doi: 10.1002/cncr.33221.

[59]	R. Zhang, Y. Zhang, S. Dong, K. Pang, X. Yang, and X. Wei, “Performance of indocyanine green in sentinel lymph node mapping and lymph node metastasis in penile cancer: systematic review, meta-analysis, and single-center experience,” *World J. Urol.*, vol. 41, no. 9, pp. 2319–2326, Sep. 2023, doi: 10.1007/s00345-023-04485-x.

[60]	S. Sethi *et al.*, “A systematic review and meta-analysis of the prevalence of human papillomavirus infection in Indigenous populations – A Global Picture,” Oct. 01, 2021, *John Wiley and Sons Inc*. doi: 10.1111/jop.13201.

[61]	W. Wang *et al.*, “Real-world impact and effectiveness assessment of the quadrivalent HPV vaccine: a systematic review of study designs and data sources,” 2022, *Taylor and Francis Ltd.* doi: 10.1080/14760584.2022.2008243.

[62]	Z. Tao *et al.*, “Prevalence of HPV Infection Among Chinese Males With HPV-Related Diseases: A Systematic Review and Meta-Analysis,” Jul. 01, 2025, *John Wiley and Sons Inc*. doi: 10.1002/jmv.70469.

[63]	J. Pow, L. Clarke, S. McHale, and C. Gray-Brunton, “A systematic review and thematic synthesis exploring how gay, bisexual and other men who have sex with men (GBMSM) experience HPV and HPV vaccination,” 2025, *Taylor and Francis Ltd.* doi: 10.1080/21645515.2025.2490440.

[64]	S. Sasidharanpillai, N. Ravishankar, V. Kamath, P. V. Bhat, P. Bhatt, and G. Arunkumar, “Prevalence of Human Papillomavirus (HPV) DNA among Men with Oropharyngeal and Anogenital Cancers: A Systematic Review and Meta-Analysis,” *Asian Pacific Journal of Cancer Prevention*, vol. 22, no. 5, pp. 1351–1364, May 2021, doi: 10.31557/APJCP.2021.22.5.1351.

[65]	C. Bennett *et al.*, “Which interventions improve HPV vaccination uptake and intention in children, adolescents and young adults? An umbrella review,” Sep. 05, 2022, *BMJ Publishing Group*. doi: 10.1136/sextrans-2022-055504.

[66]	N. Pyrgidis *et al.*, “Synchronous Surgical Management of Erectile Dysfunction and Stress Urinary Incontinence: A Systematic Review and Meta-Analysis of Reoperation Rates,” Oct. 01, 2022, *Elsevier B.V.* doi: 10.1016/j.sxmr.2022.08.003.

[67]	B. A. Hahn, M. C. Richir, A. J. Witkamp, T. de Jong, and D. D. Krijgh, “Prevalence of lower extremity edema following inguinal lymphadenectomy: A systematic review and meta-analysis,” Mar. 01, 2025, *Elsevier Ltd*. doi: 10.1016/j.jpra.2024.11.001.

[68]	N. Wongkalasin *et al.*, “Global Estimates of the HPV-related, Noncervical Cancer Burden in People With HIV and AIDS and the Potential Effect of Improved HPV Vaccine Coverage in This Population: A Systematic Review, Meta-analysis and Modeling Study,” *Open Forum Infect. Dis.*, vol. 12, no. 10, Oct. 2025, doi: 10.1093/ofid/ofaf577.

[69]	A. R. Giuliano *et al.*, “High Risk of New HPV Infection Acquisition Among Unvaccinated Young Men,” *Journal of Infectious Diseases*, vol. 229, no. 3, pp. 707–718, Mar. 2024, doi: 10.1093/infdis/jiad485.

[70]	Z. Li *et al.*, “Corpora Cavernos invasion vs. Corpus Spongiosum invasion in Penile Cancer: A systematic review and meta-analysis,” *J. Cancer*, vol. 12, no. 7, pp. 1960–1966, Jan. 2021, doi: 10.7150/jca.56504.
